# Enantioselective Molecular Recognition in a Flexible Self-Folding Cavitand

**DOI:** 10.1021/acs.orglett.3c00463

**Published:** 2023-04-11

**Authors:** Rubén Álvarez-Yebra, Ricard López-Coll, Pere Galán-Masferrer, Agustí Lledó

**Affiliations:** Institut de Química Computacional i Catàlisi (IQCC), Universitat de Girona, Maria Aurèlia Capmany 69, 17003 Girona, Spain

## Abstract

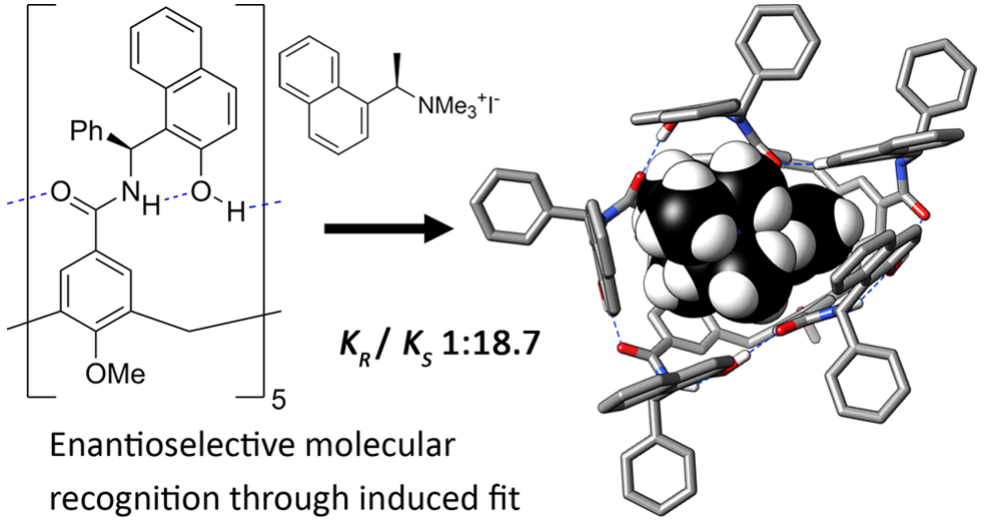

We report a chiral deep cavitand receptor based on calix[5]arene stabilized by a cooperative network of hydrogen bonds and having a highly flexible structure. The cavitand displays enantioselective molecular recognition with a series of chiral quaternary ammonium salts, providing unprecedented stability ratios between the corresponding diastereomeric host–guest complexes. Molecular dynamics simulations corroborate the higher flexibility of the new host and the emergence of superior induced-fit behavior with regards to resorcin[4]arene derived self-folding cavitands.

Taking inspiration from the molecular recognition phenomena occurring in Nature, a myriad of synthetic host–guest systems have been developed throughout the years, targeting applications such as catalysis,^1,2^ sensing,^3^ or transport.^4^ For all of these applications, the emergence of enantioselective molecular recognition has been a long sought objective.^5−9^ A universal design principle of synthetic hosts is that a receptor must be preorganized to develop significant and efficient molecular recognition behavior, although this typically comes at the expense of reducing the flexibility and adaptability of a host. In practice, the majority of synthetic host–guests systems falls short of reproducing the rich induced-fit behavior and conformational heterogeneity of proteinogenic receptors that are key to their exceptional properties.^10,11^

Self-folding cavitands are a subset of aromatic hosts that are stabilized in their closed, binding competent conformers by a hydrogen bond array akin to the networks stabilizing secondary structure in proteins. The archetypal example is the resorcin[4]arene derived octaamide cavitand (**1**, Figure 1).^12,13^ Receptors of this family have been claimed to feature induced-fit behavior, although they favor highly rigid and symmetrical cylindrical cavities.^14,15^ We recently introduced an alternative self-folding cavitand scaffold based on calix[5]arene (**2**) that displays enhanced flexibility.^16^ Cavitand **2** exists in solution as an ensemble of low symmetry cone-like conformers, which we identified as a promising platform for developing chiral and adaptable confined spaces that surpass the capabilities of current systems.

**Figure 1:**
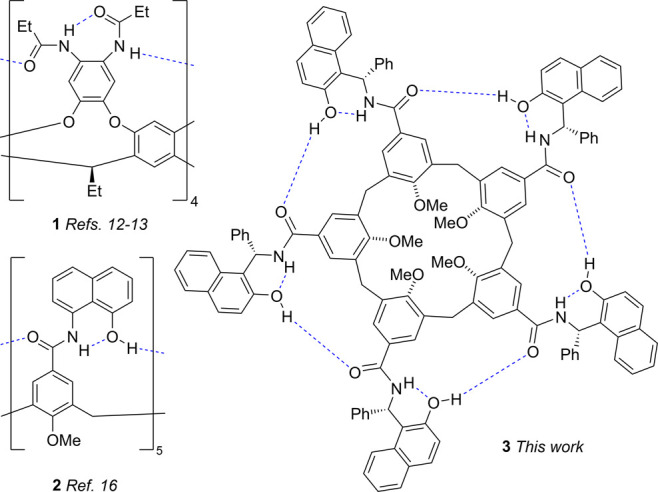
Structures of resorcin[4]arene derived (**1**) and calix[5]arene derived (**2** and **3**) self-folding cavitands.

Herein, we report a new chiral self-folding cavitand receptor (**3**), based on calix[5]arene, and discuss its unprecedented induced-fit behavior and enantioselective molecular recognition properties.^17^ Following our seminal discovery of receptor **2**,^16^ we envisaged that substitution of the 1,8-aminonaphthol panels by an α-substituted 2-(aminomethyl)phenol would be a suitable approach for introducing chirality. We rationalized that a proper choice of substituents at the chiral center could favor the formation of one of the possible *cyclodiastereomers* arising from the directional arrangement of the hydrogen bond seam, relaying the chiral information onto the macrocyclic structure of the host. A similar approach was previously used to construct a chiral resorcin[4]arene derived cavitand.^18^ However, the resulting binding space was highly symmetrical, severely limiting the emergence of enantioselective molecular recognition in such systems. We identified the so-called Betti base (**4**, Scheme 1) as a convenient building block for our approach since it is readily available in multigram quantities in both enantiomeric forms.^19,20^ In addition, preliminary modeling studies suggested that this scaffold would favor a unidirectional arrangement of the hydrogen bond network with the phenyl rings facing outward from the cavity. The opposite directional arrangement with the phenyl groups facing inward provides a structure that is too sterically congested. Receptors (*R*,*R*,*R*,*R*,*R*)-**3** and (*S*,*S*,*S*,*S*,*S*)-**3** (*R***-3** and *S***-3** for short), were synthesized in good yield by condensation of the key calix[5]arene pentaacid **5** with either enantiomer of **4**, under conventional amide coupling conditions (Scheme 1). The ease of access to either enantiomer of **3** is one of the most remarkable advantages of our system in comparison to recently reported chiral hosts, which typically require resolution by chiral stationary phase HPLC.^5−9^

**Scheme 1:**
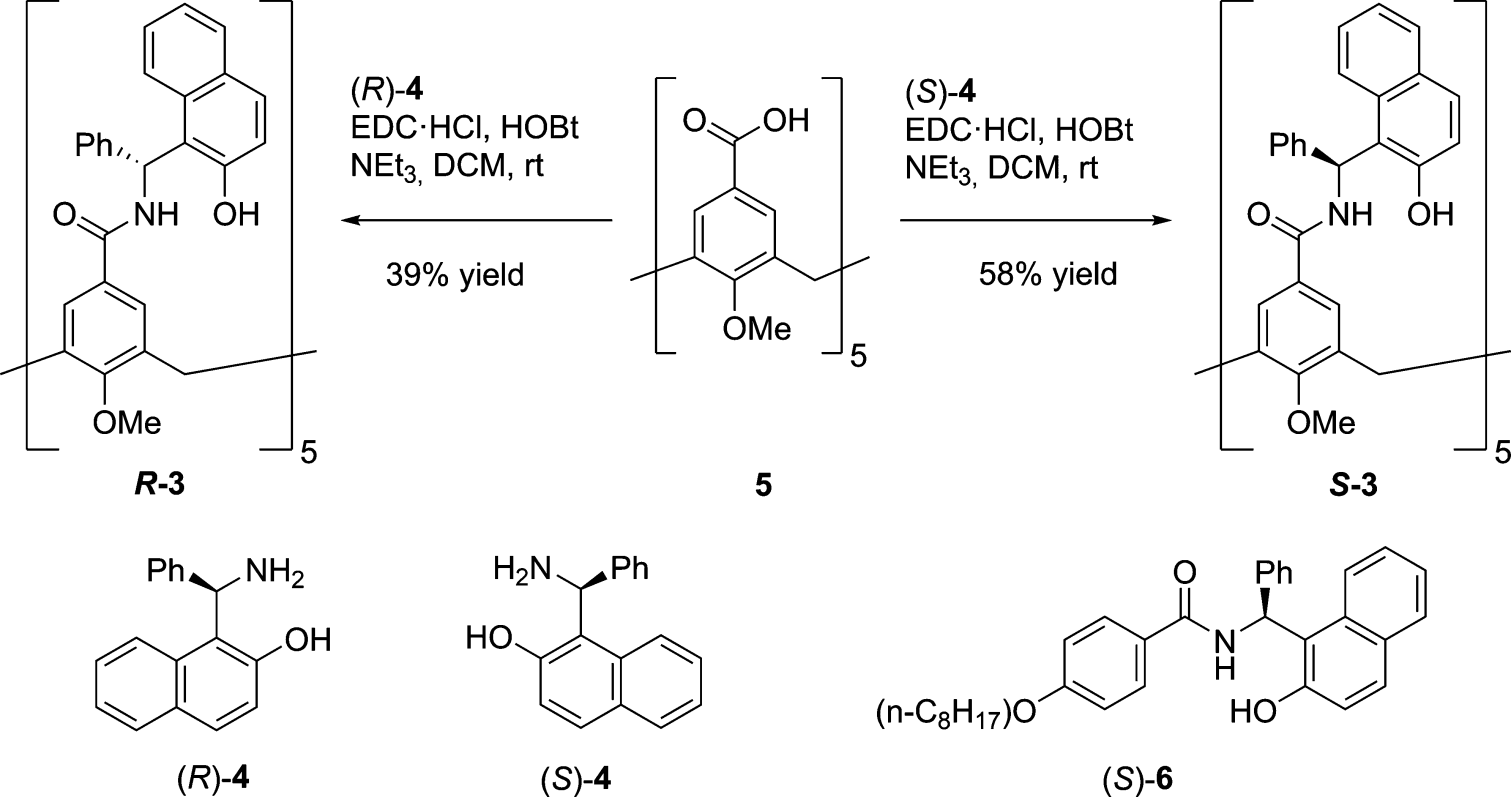
Synthesis of the Chiral Self-Folding Cavitands *S***-3** and *R***-3** Also shown are the Betti base building blocks ((*R*)-**4**/(*S*)-**4**)) and model compound (*S*)-**6**.

With **3** in hand, we first analyzed its conformational and dynamic features in solution by ^1^H NMR spectroscopy (Figure 2). In CDCl_3_ and C_6_D_6_, both the methylene and aromatic protons of the calix[5]arene core (H_a_/H_b_, H_c_/H_d_) appear as separate resonances of two AB systems, indicating restricted rotation about both the aryl–CH_2_ and the aryl–CONH bonds. The far downfield shifts of both the OH and NH protons are indicative of a cyclic intramolecular network of cooperative hydrogen bonds (Δδ_OH_ = 2.7 ppm, Δδ_NH_ = 1.8 ppm, with respect to acyclic reference (6) (*c* = 2.0 mM in C_6_D_6_)).^21^ This hypothesis is corroborated by the small temperature coefficients (Δδ/Δ*T*) observed for both OH and NH resonances of **3** and the fact that the OH and NH of **6** appear as much broader resonances. Additionally, the OH and NH resonances of **3** remain unaltered at various concentrations, ruling out the formation of hydrogen-bonded aggregates (see Supporting Information). Cavitand **3** thus exists in solution as a monomeric structure of time averaged *C*_5_ symmetry. As anticipated, the introduced stereogenic elements fix the directionality of the hydrogen bond seam since only one set of signals is observed. If this were not the case, two sets of signals corresponding to unevenly populated cyclodiastereomers would be expected. The 2D NOESY spectra of **3** in CDCl_3_ and C_6_D_6_ display cross peaks between H_a_/H_b_ and between H_c_/H_d_, indicating slow chemical exchange between two equivalent closed forms of the cavitand. This bowl inversion motion involves concerted rotation about the aryl–CH_2_ and aryl–CONH bonds and temporary disruption of the hydrogen bond seam (Figure 2). By means of EXSY experiments,^22,23^ a barrier (Δ*G*^‡^) of 17.7 kcal mol^–1^ was calculated for this process in CDCl_3_ at 298 K. Remarkably, the stabilizing effect provided by the hydrogen bond seam in **3** is comparable to that of **2** (Δ*G*^‡^_298 K_ = 17.4 kcal mol^–1^ in CDCl_3_),^16^ despite the fact that **3** has five additional bonds with free rotation involved (one NPhCH–aryl bond per aromatic panel). These barriers are also comparable to that of the unfolding motion for **1**, despite the much higher degree of covalent restriction present in resorcin[4]derived cavitands.^12−15,24^ In DMSO-*d*_6_, both the methylene and aromatic protons of **3** (H_a_/H_b_, H_c_/H_d_) appear as a single resonance peak, indicating that disruption of the stabilizing hydrogen bonds by the solvent induces rapid interconversion of bowl conformers in the NMR time scale.

**Figure 2:**
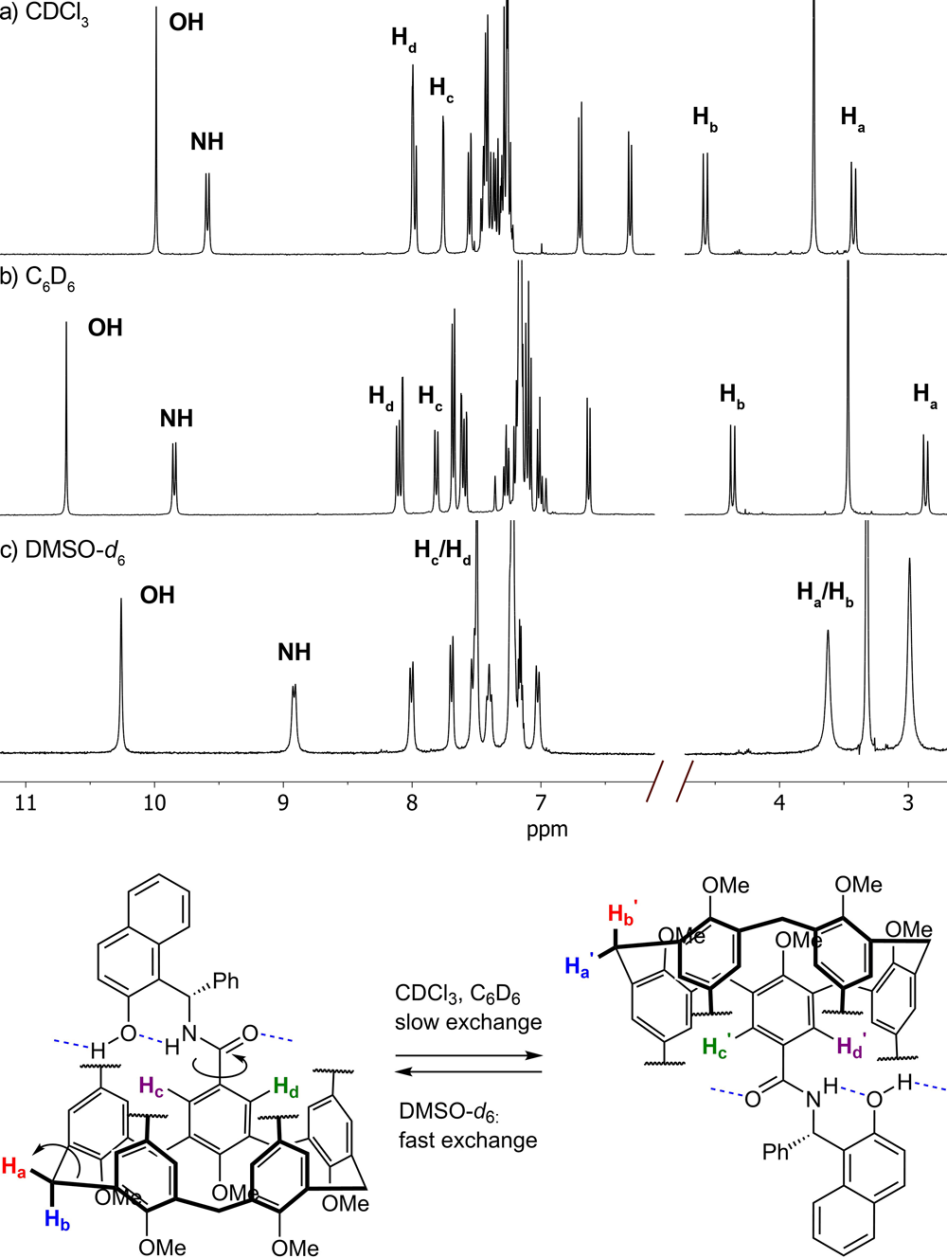
^1^H NMR spectra (400 MHz, 298 K) of **3** in (a) CDCl_3_, (b) C_6_D_6_, and (c) DMSO-*d*_6_. Bottom: scheme of the bowl inversion motion for **3**.

After characterizing the dynamic behavior of **3** in solution, we sought to demonstrate the potential of this flexible scaffold to discriminate between two enantiomers through induced-fit effects. We chose quaternary ammonium salt guests as ideal candidates to prove our hypothesis, because of their well-established affinity for the electron-rich π surfaces of calixarene receptors^25^ and because they are readily prepared from a plethora of substituted ethylamines that are commercially available in enantiopure form (see Supporting Information). Guided by preliminary modeling studies, we tested a selection of ammonium salts with diverse shapes and volumes (**G1**–**G10**, Figure 3).^26^ An interesting feature of both cavitands **2** and **3** is that their complexes display fast exchange in the NMR time scale (only one set of host signals is observed upon addition of suitable guests), despite the observed kinetic stability of the closed bowl conformer. This points at a guest exchange mechanism that, unlike that operating in resorcin[4]arene derived cavitands (**1** and its congeners), does not require unfolding of the cavitand structure.^27^ In other words, guests trapped in **2** or **3** can escape the cavity without complete disruption of the hydrogen bond seam, as a result of the cavitand’s enhanced flexibility. We determined the association constants with each enantiomer of **3** by means of ^1^H NMR titration experiments and nonlinear fit analysis (Table 1). In addition to the binding constant ratios (*K*_*R*_/*K*_*S*_), Table 1 displays the difference in the binding free energy associated to the resulting diastereomeric host–guest complexes, to better gauge the selectivity of the molecular recognition event. Remarkably, cavitand **3** is able to exert enantioselective molecular recognition for most of the guests tested and provides a notable *K*_*R*_/*K*_*S*_ ratio of 1:18.7 for (*R*)-**G3** (Table 1, entry 3). This is the highest ratio so far reported for enantioselective molecular recognition of quaternary ammonium salts, to the best of our knowledge.^6^ Remarkably, cavitand **3** provides similar association constants with guests of differing sizes, an intriguing observation that is likely the consequence of the receptor’s adaptable nature. This flexibility makes it difficult to rationalize host–guest phenomena based on static molecular models and size considerations alone. Therefore, we carried out molecular dynamics (MD) simulations of cavitands **1** and **3** and selected complexes thereof, to better understand their dynamic and molecular recognition features (Supporting Information). A 500 ns simulation of *S***-3** alone in CHCl_3_ reveals a structure that fluctuates among nonsymmetrical conformers of diverse shapes and volumes. Approximately four solvent molecules are buried in the cavity for most of the trajectory, although the host is flexible enough to adapt to lower occupancies. The bowl inversion motion of the cavitand is not observed throughout the simulation, in good agreement with the calculated exchange barrier,^27^ and the integrity of the hydrogen bond seam is maintained throughout the trajectory. The upper naphthalene panels are the part that displays wider spatial fluctuation, but the intrinsic flexibility of the calix[5]arene core can also be appreciated. For comparison, an analogous MD trajectory of **1** (1 μs) illustrates the much narrower conformational space of resorcin[4]arene derived cavitands. Interestingly, upon incorporation of (*R*)-**G3** into the cavity of *S***-3**, the populated conformational space is reduced to adapt to the shape and size of the bound guest. The guest is oriented with the naphthalene moiety buried deep in the cavity and the trimethylammonium knob near the rim of the cavity where it can establish close ion pairing with the iodide anion through the opening. At any given time, the methyl groups establish CH−π interactions at a close range with one or two of the upper naphthalene panels of **3**. This is in good agreement with the moderate shifts observed for the Me_3_N moiety of the guests (Figure 3), which would be far more pronounced if this portion of the guest was positioned in the deep narrow section of the calix[5]arene core. Finally, we computed a 500 ns trajectory for complex (*S*)-**G10**⊂*S***-3** to investigate the effect of guest size. While an energy minimum for the complex can be located at a semiempirical level, the MD simulation reveals that two CHCl_3_ molecules enter the cavity sequentially. Co-encapsulation of chloroform molecules to provide optimal occupancy has been previously observed^28,29^ and explains the fact that guests of disparate volumes can be efficiently encapsulated within **3**, thanks to the adaptable nature of the receptor. A simple way to quantify the enhanced flexibility of **3** with respect to **1** is to assess the buried volume along the trajectories in each case (see Supporting Information).^30^ These calculations clearly reveal larger volume fluctuation values for **3**.

**Table 1:** Association Constants Obtained for the Binding of Guests **G1**–**G10** in *R***-3** and/or *S***-3** in CDCl_3_^b^^a^

	Guest	*K*_a_ (M^–1^) *R*-**3**^b^	*K*_a_ (M^–1^) *S*-**3**^b^	*K*_R_/*K*_S_	|ΔΔ*G*| kcal mol^–1^
1	(1*R*,2*R*)-**G1**	129 ± 18	99 ± 6	1.3:1	0.16
2	(*R*)-**G2**	192 ± 12	42 ± 3	4.5:1	0.90
					
3	(*R*)-**G3**	31 ± 3	587 ± 73	1:18.7	1.73
					
4	(*S*)-**G4**	106 ± 15	32 ± 3	3.3:1	0.71
5	(*S*)-**G5**	414 ± 21	200 ± 21	2.1:1	0.43
6	(*S*)-**G6**	890 ± 48	1128 ± 100	1:1.3	0.14
7	(*R*)-**G7**	607 ± 88	1220 ± 118	1:2.0	0.41
8	(1*S*,2*R*)-**G8**	555 ± 68	417 ± 27	1.3:1	0.17
9	(*R*)-**G9**		147 ± 3	1:2.3	0.49
10	(*S*)-**G9**		335 ± 10		
11	(*S*)-**G10**	187 ± 11	1416 ± 92	1:7.6	1.20

a The resulting constant ratios and equivalent free energy difference between diastereomeric complexes are shown.

b Obtained from ^1^H NMR titration experiments at 298 K.

**Figure 3:**
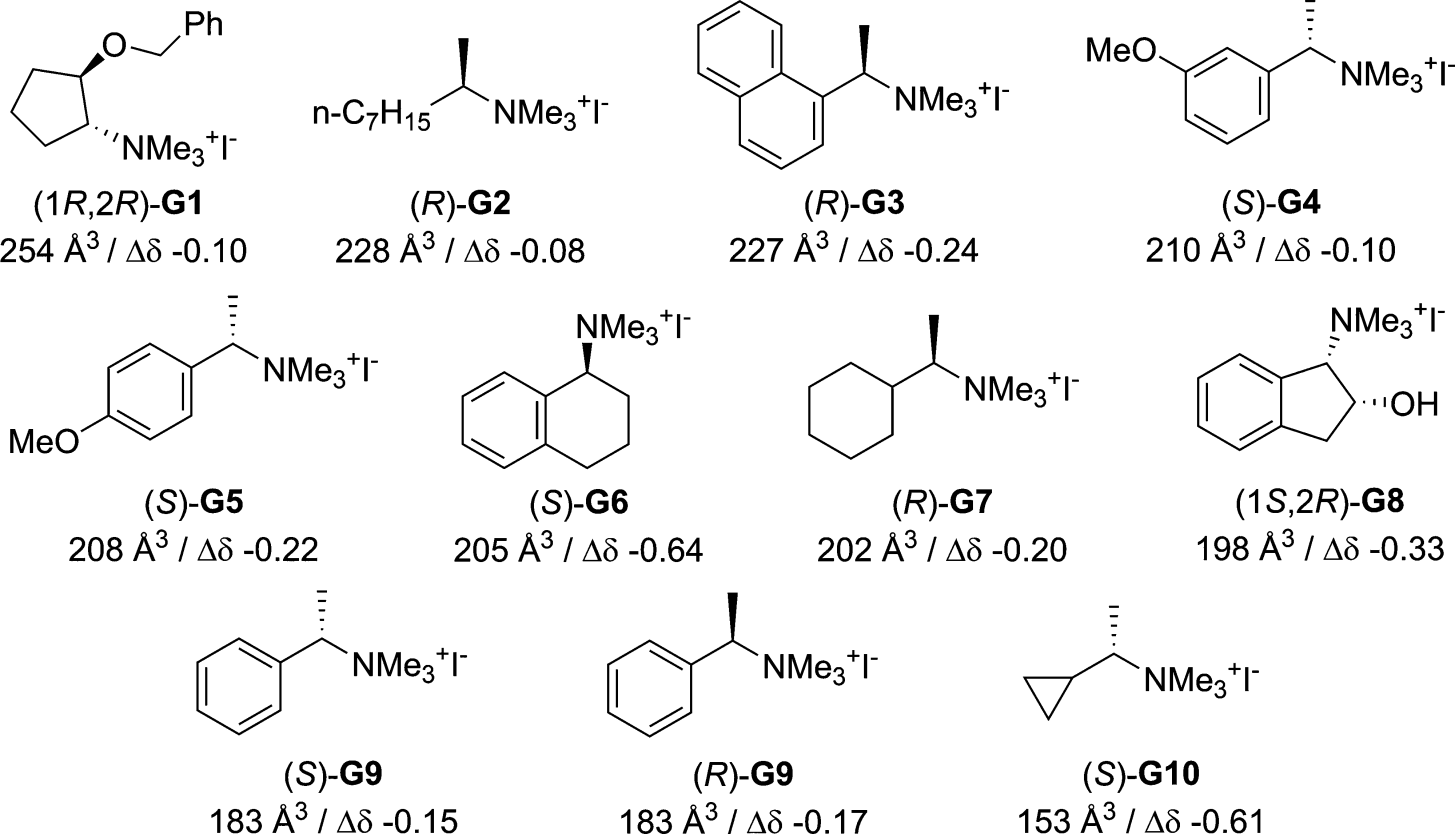
Structures of guests **G1**–**G10**, the corresponding solvent accessible volumes of the cationic portion, and the maximum shift observed (Δδ/ppm) for the Me_3_N resonance upon binding to *R***-3** or *S***-3**.

In conclusion, we have developed a new chiral self-folding cavitand receptor based on calix[5]arene that allows efficient enantioselective molecular recognition of chiral quaternary ammonium guests. This is facilitated by the flexible nature of the host, leading to superior induced-fit and conformational selection phenomena in comparison with previously reported receptors. The new design allows efficient relay of chiral information from the stereogenic elements to the cavity by means of noncovalent interactions and is a step forward from chiral hosts which are highly symmetrical^5−7,9^ and/or do not allow efficient transfer of chirality to the molecular recognition units.^18,31^ By screening a relatively reduced set of chiral quaternary ammonium ions, we have readily identified structures that provide good diastereomeric ratios. The corresponding ΔΔ*G* values would yield useful enantioselectivity levels if they corresponded to hypothetical diastereomeric transition states, giving good prospects for the use of **3** or variants thereof in supramolecular enantioselective catalysis.^5^ The modular nature of cavitand **3** and the straightforward access to either enantiomer of the host without the need of chromatographic resolution should allow facile diversification and further research in this direction.

## Data Availability

The data underlying this study are available in the published article and its Supporting Information. Spectroscopic data are available from the CORA repository, 10.34810/data571. Computational data are available from the ioChem-BD repository, 10.19061/iochem-bd-4-48.

## Supporting Information Available

Experimental procedures and characterization for compounds *R***-3**, *S***-3**, (*S*)-**6**, and **G1**–**G10**. EXSY ^1^H NMR experiments for **3** and exchange rate calculations. ^1^H NMR titration experiments. Details for MD simulations, analysis of MD trajectories, and volume calculations. Copies of NMR (^1^H, ^13^C, 2D) and HRMS spectra of new compounds (PDF)
1 μs trajectory of cavitand **1** in chloroform. The CHCl_3_ bound in the cavity is shown in CPK representation (MP4)
500 ns trajectory of cavitand *S*-**3** in chloroform. Selected CHCl_3_ molecules that occupy the cavity at some point of the trajectory are shown in CPK representation (MP4)
500 ns trajectory of cavitand *S*-**3** in chloroform with bound (*R*)-**G3**. The guest is shown in CPK representation. The iodide counter ion is shown in pink (MP4)
500 ns trajectory of cavitand *S*-**3** in chloroform with bound (*S*)-**G10**. The guest and CHCl_3_ molecules that occupy the cavity at some point of the trajectory are shown in CPK representation. The iodide counter ion is shown in pink (MP4)
