# Supplementary material for: Enantioselective Molecular Recognition in a Flexible Self-Folding Cavitand
Source: Org Lett. 2023 Apr 11;25(18):3190–4. doi: 10.1021/acs.orglett.3c00463 (PMC10186369; doi:10.1021/acs.orglett.3c00463)
Supplement: Supplementary file 1 — ol3c00463_si_001.pdf [file ol3c00463_si_001.pdf]

Supporting Information for

Enantioselective Molecular Recognition in a Flexible Self-Folding Cavitand

Rubén Álvarez-Yebra, Ricard López-Coll, Pere Galán-Masferrer, and Agustí Lledó*

Institut de Química Computacional i Catàlisi (IQCC), Universitat de Girona, Maria Aurèlia Capmany 69, 17003, Girona, Spain.

Table of contents

Table of contents **S1**

1 Materials and methods **S2**

2 Open data access **S2**

3 Synthetic procedures **S2**

(*S*) and (*R*) Betti base S2

Cavitand (+)-(*S,S,S,S,S*)-**3** (***S*-3**) S4

Cavitand (-)-(*R,R,R,R,R*)-**3** (***R*-3**) S4

Model compound (-)-(*S*)-**6** S5

3.1 Synthesis of guest compounds S6

(1*R*,2*R*)-2-(Benzyloxy)-*N*,*N*-dimethylcyclopentan-1-amine, (1*R*,2*R*)-**A1** S6

(*R*)-*N*,*N*-Dimethylnonan-2-amine, (*R*)-**A2**[4] S6

(*R*)-*N*,*N*-Dimethyl-1-(naphthalen-1-yl)ethan-1-amine, (*R*)-**A3**[5-6] S7

(*S*)-1-(3-Methoxyphenyl)-*N*,*N*-dimethylethan-1-amine, (*S*)-**A4**[7] S7

(*S*)-1-(4-Methoxyphenyl)-*N*,*N*-dimethylethan-1-amine, (*S*)-**A5**[8] S7

(*S*)-*N*,*N*-Dimethyl-1,2,3,4-tetrahydronaphthalen-1-amine, (*S*)-**A6**[9] S7

(*R*)-1-Cyclohexyl-*N*,*N*-dimethylethan-1-amine, (*R*)-**A7**[10] S7

(1*S*,2*R*)-1-(Dimethylamino)-indan-2-ol, (1*S*,2*R*)-**A8**[11] S8

(*S*)-1-Cyclopropyl-*N*,*N*-dimethylethan-1-amine, (*S*)-**A10** S8

[(1*R*,2*R*)-2-(Benzyloxy)-cyclopentan-1-yl]-trimethylammonium iodide, (1*R*,2*R*)-**G1** S8

(*R*)-(Nonan-2-yl)trimethylammonium iodide (*R*)-**G2**[12] S8

(*R*)-1-(Naphthalen-1-yl)ethyl-trimethylammonium iodide, (*R*)-**G3**[13] S9

(*S*)-Trimethyl-(1-(3-methoxyphenyl)-ethyl)ammonium iodide, (*S*)-**G4** S9

(*S*)-Trimethyl-(1-(4-methoxyphenyl)-ethyl)ammonium iodide, (*S*)-**G5**[12] S9

(*S*)-Trimethyl-(1,2,3,4-tetrahydronaphthalen-1-yl)ammonium iodide, (*S*)-**G6** S9

(*R*)-(1-Cyclohexylethyl)-trimethylammonium iodide, (*R*)-**G7**[12] S10

[(1*S*,2*R*)-2-hydroxy-indan-1-yl]trimethylammonium iodide, (1*S*,2*R*)-**G8** S10

(*S*)-1-Cyclopropylethyl-trimethylammonium iodide, (*S*)-**G10** S10

4 ^{1}H NMR characterization of 3 .... **S11**

4.1 2D NMR EXSY and exchange rate calculations S13

5 ^{1}H NMR titration experiments. **S15**

6 MD Simulations **S35**

7 References **S38**

8 NMR Spectra of new compounds and known compounds without previously reported data. **S39**

9 HRMS spectra of new compounds **S102**

1 Materials and methods

Unless otherwise noted, materials were obtained from commercial suppliers and used without further purification, and synthesis grade solvents were used. Reaction progress was monitored using thin layer chromatography (TLC) on Macherey-Nagel Xtra SIL G/UV254 silica gel plates. Flash column chromatography was performed on silica gel 60 (40-60 μm SiO_2). ^{1}H and ^{13}C NMR spectra were acquired at 298 K unless otherwise stated, at 400 MHz and 101 MHz respectively. A Bruker Ultrashield AVANCE III 400 spectrometer equipped with a 5 mm BBI probe and a Bruker ASCEND 400 spectrometer equipped with a 5 mm BBFO probe were used. NMR spectra were internally referenced to tetramethylsilane (TMS) for ^{1}H, and to the solvent signal for ^{13}C. The NMR data are reported as follows: chemical shift (δ) in ppm from TMS, multiplicity (bs = broad singlet, s = singlet, d= doublet, t = triplet, q = quartet, m = multiplet), coupling constants (Hz), integration (^{1}H) and assignment. IR spectra were recorded on an Agilent Cary 630 FT-IR spectrometer equipped with an ATR sampling accessory. High resolution mass spectra (HRMS) were acquired on a Bruker micrOTOF-QII instrument with an ESI source. Samples were introduced into the mass spectrometer ion source by direct infusion through a syringe pump and were externally calibrated using sodium formate. Optical rotation was measured on a Jasco P-2000 iRM Polarimeter equipped with an OSRAM sodium lamp (λ = 589 nm).

2 Open data access

Links for available raw data

Spectroscopic data (CORA): https://doi.org/10.34810/data571

Computational data (ioChem-BD): http://dx.doi.org/10.19061/iochem-bd-4-48

3 Synthetic procedures

(*S*) and (*R*) Betti base

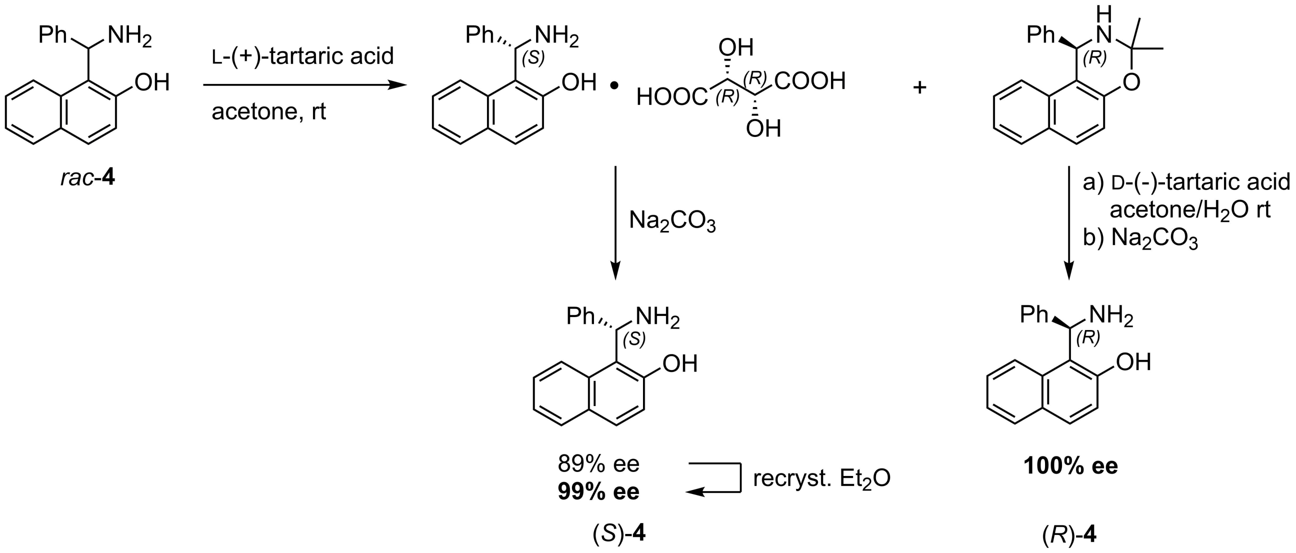

Scheme S1. Resolution of the Betti base racemate *rac*-**4**.

Racemic 1-(amino(phenyl)methyl)naphthalen-2-ol (*rac*-**4)** was synthesized and resolved according to previous reports (Scheme S1).[1-2] From the racemic amine and L-(+)-tartaric acid, the (*S*) enantiomer was obtained in 89% ee (HPLC) following the reported procedure. Recrystallization of the free base from ether[2] yielded (*S*)-**4** in 99% ee. The (*R*) ketal obtained in the resolution was treated with D-(-)-tartaric acid, and after neutralization (*R*)-**4** was obtained in 100% ee.

HPLC analysis: Chiralpak IC (4.6x250 mm, particle size 5 μm), *n*-hexane/DCM/*i*-PrOH (HPLC grade) 84.5:15:1.5 isocratic, 1 mL/min, 5 μL injection of 1 mg/mL solution in *n*-hexane/DCM 85:15. t_r (*R*)-**4** 11 min. t_r (*S*)-**4** 15 min.

HPLC chromatograms

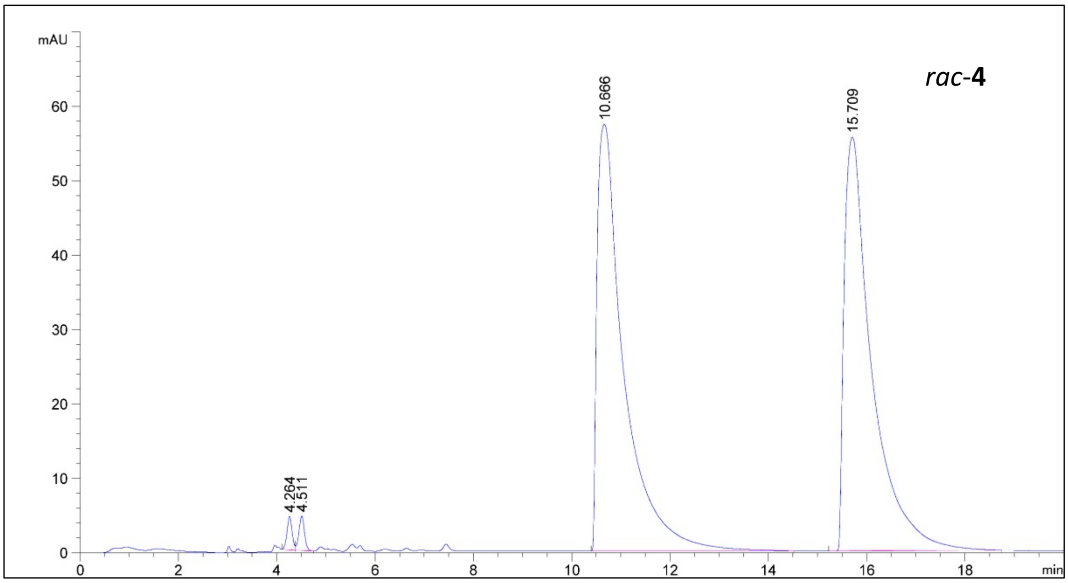

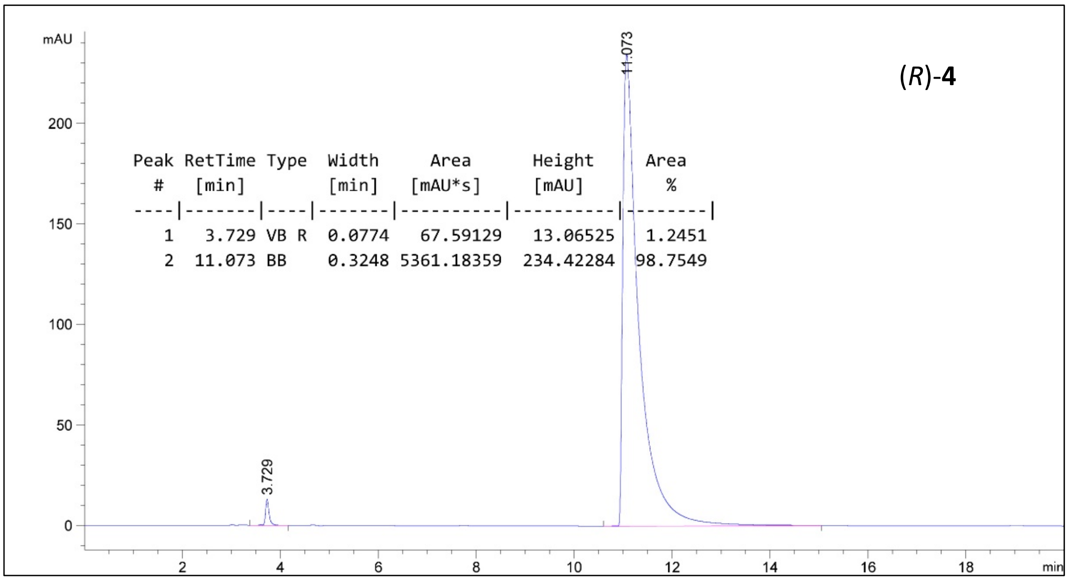

Peak #	RetTime [min]	Type	Width [min]	Area [mAU*s]	Height [mAU]	Area %
1	3.729	VB R	0.0774	67.59129	13.06525	1.2451
2	11.073	BB	0.3248	5361.18359	234.42284	98.7549

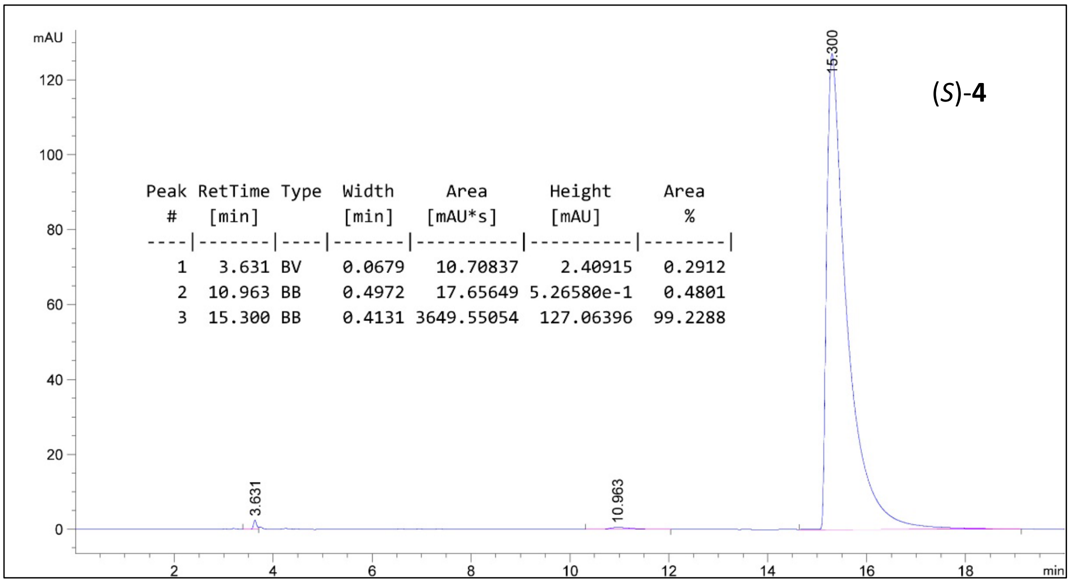

Peak #	RetTime [min]	Type	Width [min]	Area [mAU*s]	Height [mAU]	Area %
1	3.631	BV	0.0679	10.70837	2.40915	0.2912
2	10.963	BB	0.4972	17.65649	5.26580e-1	0.4801
3	15.300	BB	0.4131	3649.55054	127.06396	99.2288

Cavitand (+)-(*S,S,S,S,S*)-3 (*S*-3)

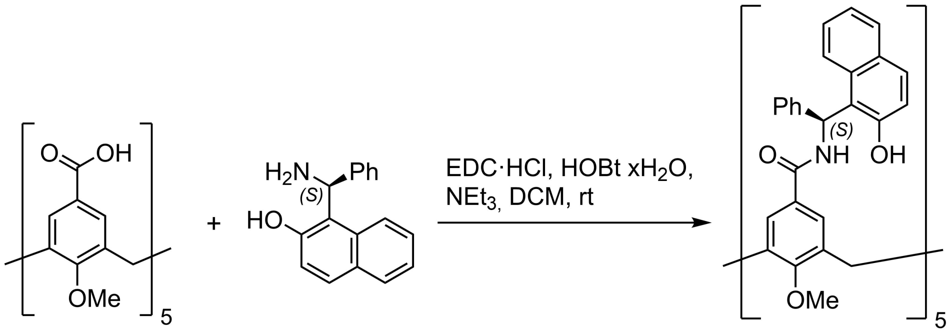

5 (*S*)-**4** ***S*-3**

In a 10 mL round bottom flask, calix[5]arene pentaacid **5**[3] (101 mg, 0.123 mmol), amine (*S*)-**4** (182 mg, 0.730 mmol, 5.9 eq.), and 1-hydroxybenzotriazole hydrate (102 mg, 0.664 mmol, 5.4 eq.) were mixed in 2.4 mL of dichloromethane. The mixture was stirred at rt for 5 min and then cooled in an ice bath. EDC·HCl (148 mg, 0.772 mmol, 6.3 eq.) was then added in one portion, followed by dropwise addition of trimethylamine (102 μL, 0.732 mmol, 5.95 eq.). The mixture was stirred at 0 °C for 30 min. and then the ice bath was removed. Stirring was continued at rt for 14 h. After this time the resulting yellow solution is diluted with 10 mL of dichloromethane and extracted twice with 10 mL of water and then with 10 mL of brine. The organic layer is dried over Na_2SO_4, filtered, and concentrated to dryness under reduced pressure. The crude product was purified by flash column chromatography (SiO_2, hexane/dichloromethane 1:9 to 100% dichloromethane). The resulting solid was triturated in methanol, filtered and dried under high vacuum to yield 140 mg (58%) of the title compound as an off-white solid. Rf 0.44 (DCM/AcOEt 98:2).

^{1}H NMR (400 MHz, $CDCl_3$) δ 9.99 (s, 1H, OH), 9.59 (d, *J* = 9.3 Hz, 1H, OH), 8.02 – 7.95 (m, 2H, CH+CH), 7.76 (d, *J* = 2.3 Hz, 1H, CH), 7.55 (dd, *J* = 8.2, 1.4 Hz, 1H, CH), 7.48 – 7.40 (m, 3H), 7.38 (d, *J* = 9.4 Hz, 1H, CH-N), 7.36 – 7.20 (m, 4H, overlap $CHCl_3$), 6.70 (d, *J* = 8.9 Hz, 1H, CH), 6.31 (d, *J* = 8.9 Hz, 1H, CH), 4.58 (d, *J* = 13.0 Hz, 1H, CH_2), 3.73 (s, 3H, CH_3-O), 3.43 (d, *J* = 13.1 Hz, 1H, CH_2) ppm.

^{13}C NMR (101 MHz, $CDCl_3$) δ 164.9 (CO), 159.3 (Cq), 153.1 (Cq), 141.1 (Cq), 134.6 (Cq), 134.0 (Cq), 132.4 (Cq), 129.3 (CH), 128.9 (CH), 128.8 (CH), 128.6 (CH), 128.2 (Cq), 127.8 (CH), 127.5(CH), 127.0 (CH), 126.8 (CH), 122.8 (CH), 122.0 (CH), 119.1 (CH), 115.6 (Cq), 62.1 (CH_3), 51.8 (CH), 27.9 (CH_2) ppm.

IR ν 3420, 3162, 3059, 2926, 1628, 1512, 1469, 1435, 1344, 1211, 1002, 813, 741, 696 cm^{-1}

HRMS (ESI-) m/z calcd. for $C_{130}H_{104}N_5O_{15}^-$ ($[M-H]^-$): 1975.7556; found: 1975.7556. m/z calcd. for $C_{130}H_{103}N_5O_{15}^{2-}$ ($[M-2H]^{2-}$) 987.3736; found: 987.3746.

Optical rotation $[\alpha]_D$ +428.3±0.6 (*c* 0.5, $CHCl_3$).

Cavitand (-)-(*R,R,R,R,R*)-3 (*R*-3)

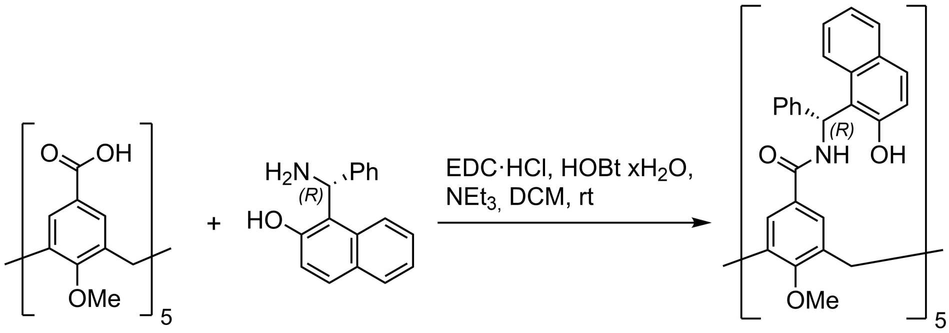

5 (*R*)-**4** ***R*-3**

R-**3** was obtained in 39% yield from 101 mg of **5** and 185 mg of (*R*)-**4** following the previous procedure.

Optical rotation $[\alpha]_D$ -416.2±0.2 (*c* 0.5, $CHCl_3$).

Model compound (-)-(*S*)-6

(-)-(*S*)-*N*-((2-hydroxynaphthalen-1-yl)(phenyl)methyl)-4-(octyloxy)benzamide, (-)-(*S*)-**6**

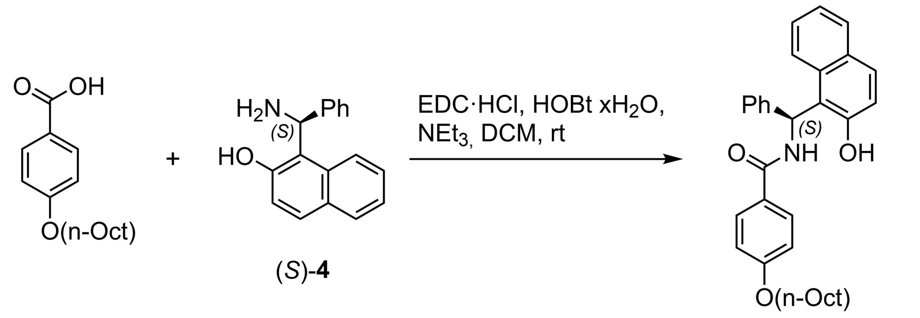

(*S*)-**6**

4-(Octyloxy)benzoic acid (49.9 mg, 0.199 mmol), 1-hydroxybenzotriazole hydrate (32.5 mg, 0.209 mmol) and (*S*)-**4** (75.2 mg, 0.299 mmol) are weighed in 10 mL round bottom flask. Dichloromehtane (1 mL) is then added, and the mixture is cooled in an ice bath under magnetic stirring. *N*-(3-Dimethylaminopropyl)-*N*'-ethylcarbodiimide hydrochloride (57.6 mg, 0.299 mmol) is added in one portion. Finally, trimethylamine is added dropwise. The mixture is stirred at 0 °C for 1 h and then the ice bath is removed. After an additional 1.5 h, the mixture is diluted with dichloromethane and treated with 10% $NaHCO_3$ aqueous solution. The resulting two phases are separated and the organic layer is washed with an additional portion of 10% $NaHCO_3$ and then with brine. The organic layer is dried over anh. Na_2SO_4, filtered and concentrated under reduced pressure. The crude product is subjected to flash column chromatography (SiO_2, hexane/ethyl acetate 90:10 to 85:15), and the title product is obtained as a white solid (88.6 mg, 92% yield).

^{1}H NMR (400 MHz, $CDCl_3$) δ 8.28 (bs, 2H, NH+OH), 7.93 (d, *J* = 8.2 Hz, 1H, CH), 7.79 – 7.71 (m, 3H, CH), 7.65 (d, *J* = 8.8 Hz, 1H, CH), 7.44 (d, *J* = 9.1 Hz, 1H, CH-N), 7.36 – 7.14 (m, 8H, CH), 6.80 (d, *J* = 8.8 Hz, 1H, CH, 2H), 3.90 (t, *J* = 6.6 Hz, 2H, CH_2), 1.74 (m, 2H, CH_2), 1.41 (m, 2H, CH_2), 1.37 – 1.20 (m, 8H, CH_2) 0.88 (t, *J* = 7.0 Hz, 3H, CH_3) ppm.

^{13}C NMR (101 MHz, $CDCl_3$) δ 167.5 (CO), 162.1 (Cq), 153.0 (Cq), 141.2 (Cq), 132.4 (Cq), 129.9 (CH), 129.5 (Cq), 129.0 (CH), 128.7 (CH), 128.5 (CH), 127.0 (CH), 126.4 (CH), 125.8 (Cq), 123.3 (CH), 123.0 (CH), 119.3 (CH, 119.1 (Cq), 114.3 (CH), 68.2 (CH_2), 49.9 (CH), 31.8 (CH_2), 29.3 (CH_2), 29.2 (CH_2), 29.1 (CH_2), 26.0 (CH_2), 22.6 (CH_2), 14.1 (CH_3) ppm.

IR ν 3382, 3125, 3065, 2919, 2849, 1631, 1542, 1500, 1252, 1180, 747, 695 cm^{-1}

HRMS (ESI-) m/z calcd. for $C_{32}H_{34}NO_3^-$ ([M-H]$^-$): 480.2533; found: 480.2515. m/z calcd. for $C_{64}H_{69}N_2O_6^-$ ([2M-H]$^-$) 961.5161; found: 961.5125. (ESI+) m/z calcd. for $C_{32}H_{35}NNaO_3^+$ ([M+Na]$^+$): 504.2509; found: 504.2512.

Optical rotation $[\alpha]_D$ -43.0±0.2 (*c* 0.5, $CHCl_3$).

3.1 Synthesis of guest compounds

Guests **G1**-**G10** were prepared by Eschweiler–Clarke methylation of the corresponding commercially available homochiral primary amines, followed by quaternarization with methyl iodide (Scheme S2).

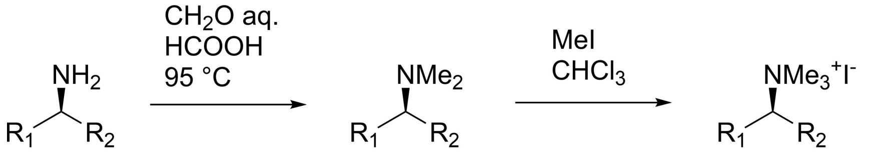

A1-**A10** **G1**-**G10**

Scheme S2. General synthetic scheme for the synthesis of guests **G1**-**G10**.

General procedure for the preparation of chiral tertiary amines A1-**A10** (adapted from ref. 9): the amine (3.5 mmol) is weighed in a 25 mL pressure tube equipped with a Teflon screw cap and a magnetic stir bar. The tube is purged with nitrogen and then 1.55 mL of aqueous formaldehyde solution (37%, 20.8 mmol) and 1.32 mL of formic acid (35 mmol) are added under nitrogen. The tube is sealed and after stirring for a few minutes an homogeneous solution is obtained. The sealed tube is heated in an oil bath at 95 °C under vigorous stirring for 6 h. After this time, the solution is cooled down to room temperature and the pressure that has built in the tube is released with caution. The solution is diluted with 30 mL of water and extracted with two 15 mL portions of dichloromethane, which are discarded. The resulting aqueous layer is basified with 3M NaOH to pH 10-11, causing the precipitation of the amine. The resulting aqueous suspension is extracted with 3 25 mL portions of dichloromethane. The combined dichloromethane layers are dried with Na_2SO_4, filtered, and concentrated under reduced pressure (250 mbar, bath temperature 20 °C). The resulting oil is purified by bulb to bulb distillation in a Kugelrohr apparatus.

(1*R*,2*R*)-2-(Benzyloxy)-*N*,*N*-dimethylcyclopentan-1-amine, (1*R*,2*R*)-A1

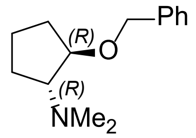

From 414 mg (2.16 mmol) of (1R,2R)-2-(benzyloxy)-cyclopentan-1-amine (+99%, 98% ee, Chipros/Thermo Scientific), 311 mg (65%) of (1R,2R)-A1 are obtained as a clear colorless oil after distillation (5 mbar, furnace T 170-175 °C).

^{1}H NMR (400 MHz, $CDCl_3$) δ 7.40 – 7.21 (m, 5H, CH), 4.55 (d, *J* = 11.6 Hz, 1H, CH_2), 4.44 (d, *J* = 11.6 Hz, 1H, CH_2), 3.85 (ddd, *J* = 6.9, 4.2, 4.2 Hz, 1H, CH), 2.57 (ddd, *J* = 8.7, 7.5, 4.6 Hz, 1H, CH), 2.27 (s, 6H, CH_3), 1.97 – 1.57 (m, 6H, CH_2), 1.44 (m, 1H, CH) ppm.

^{13}C NMR (101 MHz, $CDCl_3$) δ 138.6 (Cq), 128.3 (CH), 127.8 (CH), 127.5 (CH), 83.5 (CH), 73.4 (CH), 71.3 (CH_2), 44.1 (CH_3), 31.1 (CH_2), 29.6 (CH_2), 22.5 (CH_2) ppm.

HRMS (ESI+) m/z calcd. for $C_{14}H_{22}NO^+$ ($[M+H]^+$): 220.1696; found: 220.1701.

IR ν 3028, 2948, 2863, 2816, 2768, 1451, 1095, 1065, 732, 695 cm^{-1}

(*R*)-*N*,*N*-Dimethylnonan-2-amine, (*R*)-A2[4]

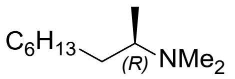

From 478 mg (3.34 mmol) of (*R*)-2-nonanamine (+99%, +98% ee, Chipros/Thermo Scientific), 420 mg (73%) of (*R*)-**A2** are obtained as a clear colorless oil are obtained after distillation (9 mbar, furnace T 110 °C).

^{1}H NMR (400 MHz, $CDCl_3$) δ 2.46 (m, 1H, CH), 2.22 (s, 6H, CH_3), 1.48 (m, 1H, CH_2), 1.28 (s, 11H, 6xCH_2), 0.94 (d, *J* = 6.5 Hz, 3H, CH_3), 0.88 (t, *J* = 7.0 Hz, 3H, CH_3) ppm.

^{13}C NMR (101 MHz, $CDCl_3$) δ 59.2 (CH), 40.7 (CH_3), 33.6 (CH_2), 31.9 (CH_2), 29.9 (CH_2), 29.3 (CH_2), 26.9 (CH_2), 22.7 (CH_2), 14.1 (CH_3), 13.6 (CH_3) ppm.

(*R*)-*N*,*N*-Dimethyl-1-(naphthalen-1-yl)ethan-1-amine, (*R*)-A3[5-6]

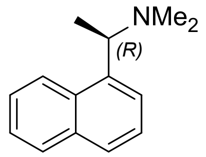

From 683 mg (3.99 mmol) of (*R*)-1-(naphthalen-1-yl)ethan-1-amine (+99%, +99% ee, Chipros/Thermo Scientific), 506 mg (64%) of (*R*)-**A3** are obtained as a clear colorless oil are obtained after distillation (6 mbar, furnace T 170 °C).

^{1}H NMR (400 MHz, $CDCl_3$) δ 8.38 (d, *J* = 8.3 Hz, 1H, CH), 7.84 (m, 1H, CH), 7.73 (d, *J* = 8.1 Hz, 1H, CH), 7.58 (d, *J* = 7.2 Hz, 1H, CH), 7.54 – 7.39 (m, 3H, 3x CH), 4.00 (q, *J* = 6.7 Hz, 1H, CH), 2.27 (s, 6H, CH_3), 1.47 (d, *J* = 6.7 Hz, 3H, CH_3) ppm.

^{13}C NMR (101 MHz, $CDCl_3$) δ 141.0 (Cq), 134.0 (Cq), 131.6 (Cq), 128.8 (CH), 127.2 (CH), 125.6 (CH), 125.4 (CH), 125.3 (CH), 124.4 (CH), 123.9 (CH), 62.3 (CH), 43.6 (CH_3), 19.1 (CH_3) ppm.

(*S*)-1-(3-Methoxyphenyl)-*N*,*N*-dimethylethan-1-amine, (*S*)-A4[7]

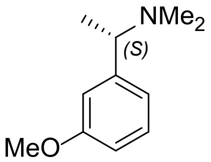

From 401 mg (2.65 mmol) of (*S*)-1-(3-methoxyphenyl)ethan-1-amine (97%, +99% ee, Thermo Scientific), 375 mg (79%) of (*S*)-**A4** are obtained as a clear colorless oil are obtained after distillation (6 mbar, furnace T 125-130 °C).

^{1}H NMR (400 MHz, $CDCl_3$) δ 7.22 (t, *J* = 7.8 Hz, 1H, CH), 6.92 – 6.84 (m, 2H, CH), 6.78 (ddd, *J* = 8.2, 2.6, 1.0 Hz, 1H, CH), 3.81 (s, 3H, CH_3), 3.20 (q, *J* = 6.7 Hz, 1H, CH), 2.20 (s, 6H, CH_3), 1.36 (d, *J* = 6.7 Hz, 3H, CH_3) ppm.

^{13}C NMR (101 MHz, $CDCl_3$) δ 159.6 (Cq), 146.0 (Cq), 129.1 (CH), 120.0 (CH), 113.0 (CH), 112.3 (CH), 66.1 (CH), 55.2 (CH_3), 43.4 (CH_3), 20.4 (CH_3) ppm.

(*S*)-1-(4-Methoxyphenyl)-*N*,*N*-dimethylethan-1-amine, (*S*)-A5[8]

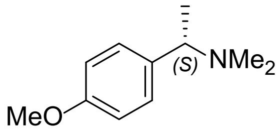

From 682 mg (4.51 mmol) of (*S*)-1-(4-methoxyphenyl)ethan-1-amine (+99%, 98% ee, Chipros/Thermo Scientific), 451 mg (56%) of (*S*)-**A5** are obtained as a clear colorless oil are obtained after distillation (6 mbar, furnace T 135-140 °C).

^{1}H and ^{13}C spectra match the previously reported data.

(*S*)-*N*,*N*-Dimethyl-1,2,3,4-tetrahydronaphthalen-1-amine, (*S*)-A6[9]

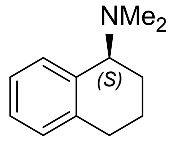

From 902 mg (6.13 mmol) of (*S*)-1,2,3,4-tetrahydronaphthalen-1-amine (+99%, 99% ee, Chipros/Thermo Scientific), 821 mg (76%) of (*S*)-**A6** are obtained as a clear colorless oil are obtained after distillation (6-7 mbar, furnace T 135 °C).

^{1}H NMR (400 MHz, $CDCl_3$) δ 7.59 (d, *J* = 7.4 Hz, 1H, CH), 7.13 (m, 2H, CH+CH), 7.05 (d, *J* = 7.1 Hz, 1H, CH), 3.77 (m, 1H, CH), 2.84 – 2.64 (m, 2H, CH_2), 2.27 (s, 6H, CH_3), 1.98 – 1.94 (m, 2H, CH_2), 1.71 (s, 2H. CH_2) ppm.

^{13}C NMR (101 MHz, $CDCl_3$) δ 138.6 (Cq), 138.2 (Cq), 128.7 (CH), 128.4 (CH), 126.2 (CH), 125.6 (CH), 62.7 (CH), 40.8 (CH_3), 29.7 (CH_2), 21.5 (CH_2), 20.2 (CH_2) ppm.

(*R*)-1-Cyclohexyl-*N*,*N*-dimethylethan-1-amine, (*R*)-A7[10]

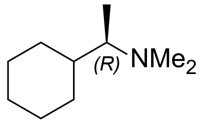

From 409 (3.21 mmol) of (*R*)-1-cyclohexylethan-1-amine (98%, 94% ee, Chipros/Thermo Scientific), 383 mg (77%) of (*R*)-**A7** are obtained as a clear colorless oil are obtained after distillation (10 mbar, furnace T 100 °C).

^{1}H NMR (400 MHz, $CDCl_3$) δ 2.19 (s, 6H, CH_3), 2.13 (m, 1H, CH), 1.88 (m, 1H, CH_2), 1.73 (m, 3H, CH_2+CH_2), 1.65 (m, 1H, CH_2), 1.38 – 1.05 (m, 4H), 0.98 – 0.82 (m, 2H, CH_2+CH_2), 0.86 (d, *J* = 6.5 Hz, 3H, CH_3) ppm.

^{13}C NMR (101 MHz, $CDCl_3$) δ 64.2 (CH), 41.1 (CH_3+CH), 31.1 (CH_2), 29.7 (CH_2), 26.8 (CH_2), 26.58 (CH_2), 26.55 (CH_2), 9.1 (CH_3) ppm.

(1*S*,2*R*)-1-(Dimethylamino)-indan-2-ol, (1*S*,2*R*)-A8[11]

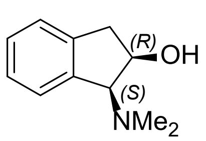

From 256 mg (1.72 mmol) of (1*S*,2*R*)-1-aminoindan-2-ol (99%, +98.5% ee, Sigma Aldrich), 285 mg (96%) of (1*S*,2*R*)-**A8** are obtained as a pale brown oil. The crude product was used in the next step without distillation.

^{1}H and ^{13}C spectra match the previously reported data.

(*S*)-1-Cyclopropyl-*N*,*N*-dimethylethan-1-amine, (*S*)-A10

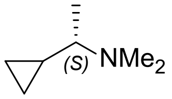

From 446 mg (5.24 mmol) of (*S*)-1-cyclopropylethan-1-amine (98%, +98% ee, Chip-ros/Thermo Scientific), 469 mg (79%) of (*S*)-**A10** are obtained as a clear colorless oil are obtained after distillation (140 mbar, furnace T 75 °C).

^{1}H NMR (400 MHz, $CDCl_3$) δ 2.33 (s, 6H, CH_3), 1.56 (dq, *J* = 8.8, 6.5 Hz, 1H, CH), 1.10 (d, *J* = 6.5 Hz, 3H, CH_3), 0.73 (m, 1H, CH), 0.57 (m, 1H, CH_2), 0.44 (m, 1H, CH_2), 0.28 (m, 1H, CH_2), 0.00 (m, 1H, CH_2) ppm.

^{13}C NMR (101 MHz, $CDCl_3$) δ 65.5 (CH), 42.4 (CH_3), 16.8 (CH_3), 14.8 (CH), 6.4 (CH_2), 2.1 (CH_2) ppm.

HRMS (ESI+) m/z calcd. for $C_7H_{16}N^+$ ($[M+H]^+$): 114.1277; found: 114.1278.

IR ν 3075, 2969, 2863, 2816, 2769, 1453, 1073, 1043 cm^{-1}

General procedure for the preparation of chiral trimethylammonium iodides G1-G10. In a typical run, the amine (1 mmol) was dissolved in 1 mL of $CHCl_3$ in a 25 mL round bottom flask. Methyl iodide (1.3 mmol) was then added dropwise, and the resulting solution was stirred at room temperature for 3h. After this time, the solvent was removed under reduced pressure, yielding an oil that was further dried under high vacuum until a foam or solid appeared. The solid was triturated in diethyl ether, filtered, washed with additional diethyl ether, and dried under high vacuum.

[(1*R*,2*R*)-2-(Benzyloxy)-cyclopentan-1-yl]-trimethylammonium iodide, (1*R*,2*R*)-G1

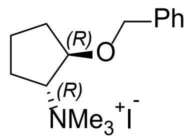

From 204 mg (0.928 mmol) of **A1** and 75 μL (1.21 mmol) of MeI, 315 mg (94%) of (1*R*,2*R*)-**G1** were obtained as a white powder following the general procedure.

^{1}H NMR (400 MHz, $CDCl_3$) δ 7.41 – 7.28 (m, 5H, CH), 4.65 (d, *J* = 11.4 Hz, 1H, CH_2), 4.57 (d, *J* = 11.4 Hz, 1H, CH_2), 4.42 (dd, *J* = 6.3, 6.3 Hz, 1H, CH), 3.72 (ddd, *J* = 8.8, 8,8, 6.2 Hz, 1H, CH), 3.41 (s, 9H, CH_3), 2.27 – 2.06 (m, 2H, CH_2), 1.99 (m, 1H, CH_2), 1.86 (m, 3H, CH_2) ppm.

^{13}C NMR (101 MHz, $CDCl_3$) δ 136.9 (Cq), 128.7 (CH), 128.3 (CH), 128.2 (CH), 81.7 (CH), 77.9 (CH), 71.5 (CH_2), 53.1 (CH_3), 30.1 (CH_2), 25.7 (CH_2), 21.0 (CH_2) ppm.

HRMS (ESI+) m/z calcd. for $C_{15}H_{24}NO^+$ ($[M-I]^+$): 234.1852; found: 234.1862.

IR ν 2999, 2964, 2939, 2863, 2769, 1475, 1451, 1088, 1060, 954, 784, 697 cm^{-1}

(*R*)-(Nonan-2-yl)trimethylammonium iodide (*R*)-G2[12]

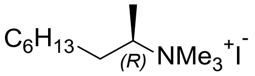

From 222 mg (1.296 mmol) of **A2** and 105 μL (1.684 mmol) of MeI, 371 mg (91%) of (*R*)-**G2** were obtained as a white powder following the general procedure.

^{1}H NMR (400 MHz, $CDCl_3$) δ 3.77 (m, 1H, CH), 3.40 (s, 9H, CH_3), 1.94 (m, 1H, CH_2), 1.56 – 1.18 (m, 11H, 6xCH_2), 1.46 (overlapping with previous multiplet, d, *J* = 6.6 Hz, 3H, CH_3), 0.88 (t, *J* = 6.9 Hz, 3H, CH_3).

^{13}C NMR (101 MHz, $CDCl_3$) δ 71.9 (CH), 51.5 (CH_3), 31.6 (CH_2), 30.3 (CH_2), 29.4 (CH_2), 29.1 (CH_2), 26.9 (CH_2), 22.6 (CH_2), 14.4 (CH_3), 14.1 (CH_3) ppm.

(*R*)-1-(Naphthalen-1-yl)ethyl-trimethylammonium iodide, (*R*)-G3[13]

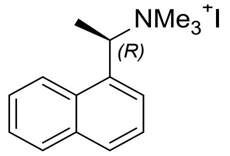

From 230 mg of **A3** (1.154 mmol) and 93 μL (1.50 mmol) of MeI, a thick viscous oil was obtained, which was subsequently purified by flash column chromatography (SiO_2, DCM/MeOH 95:5 to 90:10). The resulting product is hygroscopic, and was thoroughly dried under high vacuum overnight, in a desiccator containing P_2O_5. After drying, 377 mg (96%) (*R*)-**G3** were obtained as a yellowish foam.

^{1}H NMR (400 MHz, $CDCl_3$) δ 8.86 (d, *J* = 8.7 Hz, 1H, CH), 7.99 (d, *J* = 8.2 Hz, 1H, CH), 7.91 (d, *J* = 8.2 Hz, 1H, CH), 7.81 – 7.71 (m, 2H, CH), 7.63 – 7.51 (m, 2H, CH), 6.14 (q, *J* = 6.9 Hz, 1H, CH), 3.45 (s, 9H, CH_3), 2.02 (d, *J* = 6.8 Hz, 3H, CH_3) ppm.

^{13}C NMR (101 MHz, $CDCl_3$) δ 134.0 (Cq), 132.4 (Cq), 131.8 (CH), 129.3 (CH), 128.7 (Cq), 128.5 (CH), 127.9 (CH), 126.8 (CH), 124.8 (CH), 124.0 (CH), 67.8 (CH), 52.0 (CH_3), 16.9 (CH_3) ppm.

(*S*)-Trimethyl-(1-(3-methoxyphenyl)-ethyl)ammonium iodide, (*S*)-G4

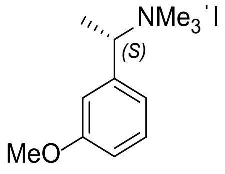

From 160 mg (0.893 mmol) of (*S*)-**A4** and 72 μL (1.16 mmol) of MeI, 263 mg (92%) of (*S*)-**G4** were obtained as a white powder following the general procedure.

^{1}H NMR (400 MHz, $CDCl_3$) δ 7.38 (dd, *J* = 8.0, 8.0 Hz, 1H, CH), 7.26 (bs, 1H, CH), 7.19 (d, *J* = 7.7 Hz, 1H, CH), 7.00 (dd, *J* = 8.3, 2.2 Hz, 1H, CH), 5.40 (q, *J* = 7.0 Hz, 1H, CH), 3.86 (s, 3H, CH_3), 3.40 (s, 9H, CH_3), 1.84 (d, *J* = 6.9 Hz, 3H, CH_3) ppm.

^{13}C NMR (101 MHz, $CDCl_3$) δ 160.0 (Cq), 133.9 (Cq), 130.4 (CH), 122.1 (broad, CH), 116.6 (broad, CH), 116.2 (CH), 73.3 (CH), 56.0 (CH_3), 51.7 (CH_3), 15.5 (CH_3) ppm.

HRMS (ESI+) m/z calcd. for $C_{12}H_{20}NO^+$ ($[M-I]^+$): 194.1539; found: 194.1542.

IR ν 2999, 2940, 2830, 2769, 1607, 1583, 1489, 1461, 1266, 1035, 834, 788, 726 cm^{-1}

(*S*)-Trimethyl-(1-(4-methoxyphenyl)-ethyl)ammonium iodide, (*S*)-G5[12]

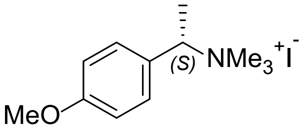

From 200 mg (1.116 mmol) of (*S*)-**A5** and 90 μL (1.45 mmol) of MeI, 341 mg (95%) of (*S*)-**G5** were obtained as a white powder following the general procedure.

^{1}H NMR (400 MHz, $CDCl_3$) δ 7.58 (d, *J* = 8.5 Hz, 1H, CH), 6.96 (d, *J* = 9.0 Hz, 1H, CH), 5.35 (q, *J* = 7.0 Hz, 1H, CH), 3.83 (s, 3H, CH_3), 3.35 (s, 9H, CH_3), 1.83 (d, *J* = 7.0 Hz, 3H, CH_3) ppm.

^{13}C NMR (101 MHz, $CDCl_3$) δ 161.3 (Cq), 132.0 (CH), 124.1 (Cq), 114.6 (CH), 73.2 (CH), 55.5 (CH_3), 51.4 (CH_3), 15.6 (CH_3) ppm.

(*S*)-Trimethyl-(1,2,3,4-tetrahydronaphthalen-1-yl)ammonium iodide, (*S*)-G6

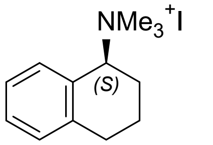

From 246 mg (1.403 mmol) of (*S*)-**A6** and 113 μL (1.824 mmol) of MeI, 272 mg (61%) of (*S*)-**G6** were obtained as a white powder following the general procedure.

^{1}H NMR (400 MHz, $CDCl_3$) δ 7.63 (d, *J* = 7.7 Hz, 1H, CH), 7.39 (td, *J* = 7.5, 1.4 Hz, 1H, CH), 7.31 (d, *J* = 7.6 Hz, 1H, CH), 7.25 (d, *J* = 7.6 Hz, 1H, CH), 5.47 (dd, *J* = 8.1, 4.3 Hz, 1H, CH), 3.38 (s, 9H, CH_3), 2.82 (dd, *J* = 7.8, 5.2 Hz, 2H, CH_2), 2.44 (m, 2H, CH_2), 2.16 (m, 1H, CH_2), 1.64 (m, 1H, CH_2) ppm.

^{13}C NMR (101 MHz, $CDCl_3$) δ 142.7 (Cq), 133.9 (CH), 130.7 (CH), 130.0 (CH), 126.6 (CH), 126.2 (Cq), 73.1 (CH), 51.7 (CH_3), 28.8 (CH_2), 24.2 (CH_2), 21.6 (CH_2) ppm.

HRMS (ESI+) m/z calcd. for $C_{13}H_{20}N^+$ ($[M-I]^+$): 190.1590; found: 190.1593.

IR ν 3006, 2929, 2862, 1481, 1449 1399, 951, 832, 746 cm^{-1}

(*R*)-(1-Cyclohexylethyl)-trimethylammonium iodide, (*R*)-G7[12]

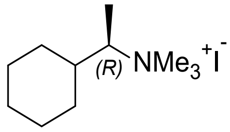

From 221 mg (1.423 mmol) of (*R*)-**A7** and 115 µL (1.851 mmol) of MeI, 407 mg (96%) of (*R*)-**G7** were obtained as a white powder following the general procedure.

^{1}H NMR (400 MHz, $CDCl_3$) δ 3.71 (q, *J* = 7.0 Hz, 1H, CH), 3.42 (s, 9H, CH_3), 2.05 (t, *J* = 12.1 Hz, 1H, CH), 1.88 – 1.64 (m, 5H, CH_2), 1.53 – 1.32 (m, 5H, CH_2+CH_3), 1.30 – 1.06 (m, 3H, CH_2) ppm.

^{13}C NMR (101 MHz, $CDCl_3$) δ 76.3 (CH), 52.2 (CH_3), 37.4 (CH), 33.0 (CH_2), 28.3 (CH_2), 26.6 (CH_2), 25.8 (CH_2), 25.7 (CH_2), 10.7 (CH_3) ppm.

[(1*S*,2*R*)-2-hydroxy-indan-1-yl]trimethylammonium iodide, (1*S*,2*R*)-G8

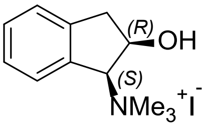

From 131 mg (0.741 mmol) of (1*S*,2*R*)-**A8** and 137 µL of MeI (0.963 mmol), a sticky foam was obtained after drying the crude mixture under high vacuum. The product was recrystallized as follows: the crude was dissolved in AcOEt/MeOH and concentrated under reduced pressure until a precipitate was observed, at which point the mixture was cooled in an iced bath. The formed precipitate was filtered and dried under high vacuum, yielding 123 mg (52%) of (1*S*,2*R*)-**G8** as a white powder.

^{1}H NMR (400 MHz, DMSO-D6) δ 7.64 (d, *J* = 7.7 Hz, 1H, CH), 7.48 (m, 1H, CH), 7.41 (d, *J* = 7.5 Hz, 1H, CH), 7.36 (t, dd = 7.5, 7.5 Hz, 1H, CH), 6.45 (d, *J* = 4.7 Hz, 1H, OH), 4.85 (m, 1H, CH), 4.79 (m, 1H, CH), 3.21 (s, 9H, CH_3), 3.10 (m, 2H, CH_2) ppm.

^{13}C NMR (101 MHz, DMSO) δ 143.0 (Cq), 132.5 (Cq), 130.7 (CH), 129.2 (CH), 127.0 (CH), 125.9 (CH), 75.9 (CH), 72.1 (CH), 52.1 (CH_3), 39.0 (CH_2, overlap DMSO) ppm.

HRMS (ESI+) m/z calcd. for $C_{12}H_{18}NO^+$ ($[M-I]^+$): 192.1383; found: 192.1386.

IR ν 3259, 3055, 2998, 2942, 1481, 1477, 1207, 1095, 753 cm^{-1}

(*S*)-1-Cyclopropylethyl-trimethylammonium iodide, (*S*)-G10

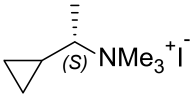

From 202 mg (1.784 mmol) of (*S*)-**A10** and 144 µL (2.32 mmol) of MeI, 352 mg (77%) of (*S*)-**G10** were obtained as a white powder following the general procedure.

^{1}H NMR (400 MHz, $CDCl_3$) δ 3.52 (dq, *J* = 10.0, 6.7 Hz, 1H, CH), 3.45 (s, 9H, CH_3), 1.55 (d, *J* = 6.6 Hz, 3H, CH_3), 1.11 (m, 1H, CH), 1.07 – 0.93 (m, 2H, CH_2), 0.83 (m, 1H, CH_2), 0.44 (m, 1H, CH_2) ppm.

^{13}C NMR (101 MHz, $CDCl_3$) δ 75.1 (CH), 51.8 (CH_3), 15.7 (CH_3), 12.2 (CH), 9.0 (CH_2), 3.4 (CH_2) ppm.

HRMS (ESI+) m/z calcd. for $C_{18}H_{18}N^+$ ($[M-I]^+$): 128.1434; found: 128.1432.

IR ν 3004, 1486, 1445, 1253, 1067, 958, 926, 854 cm^{-1}

4 ^{1}H NMR characterization of 3

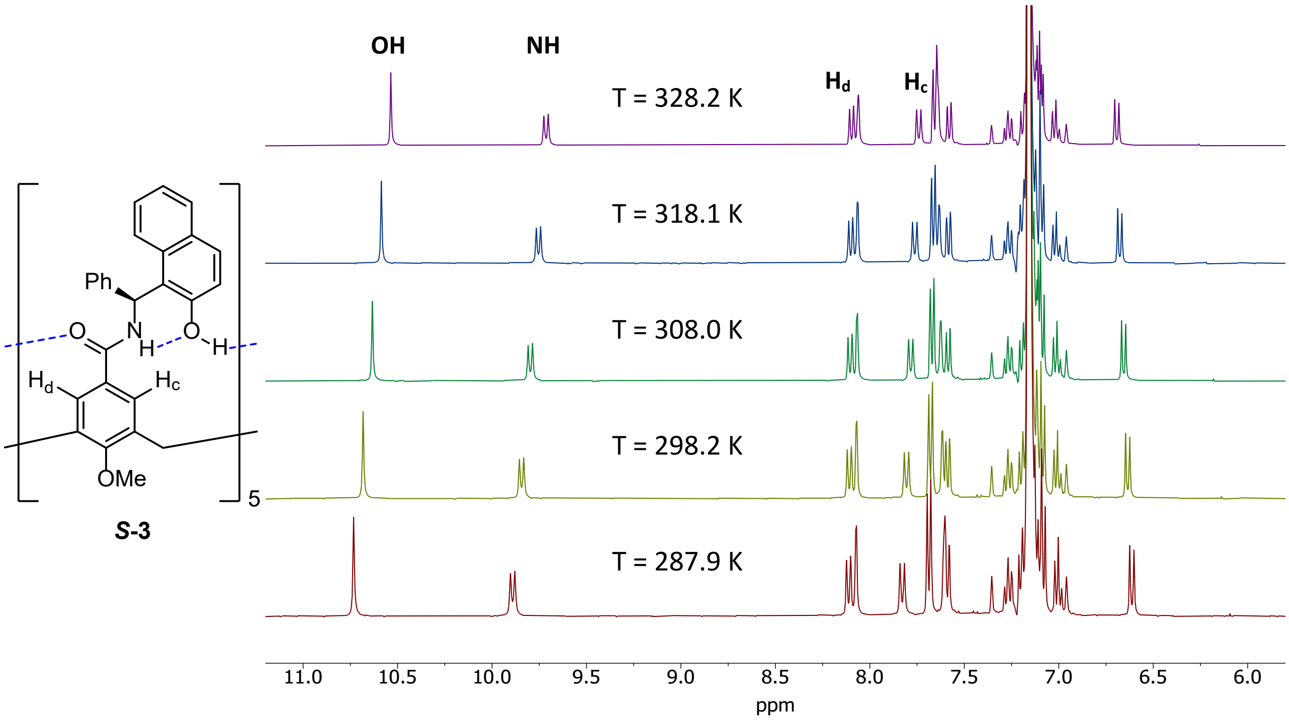

Figure S1. Downfield region of the ^{1}H NMR spectra of ***S*-3** in C_6D_6 at different temperatures ([**3**] = 1.0 mM).

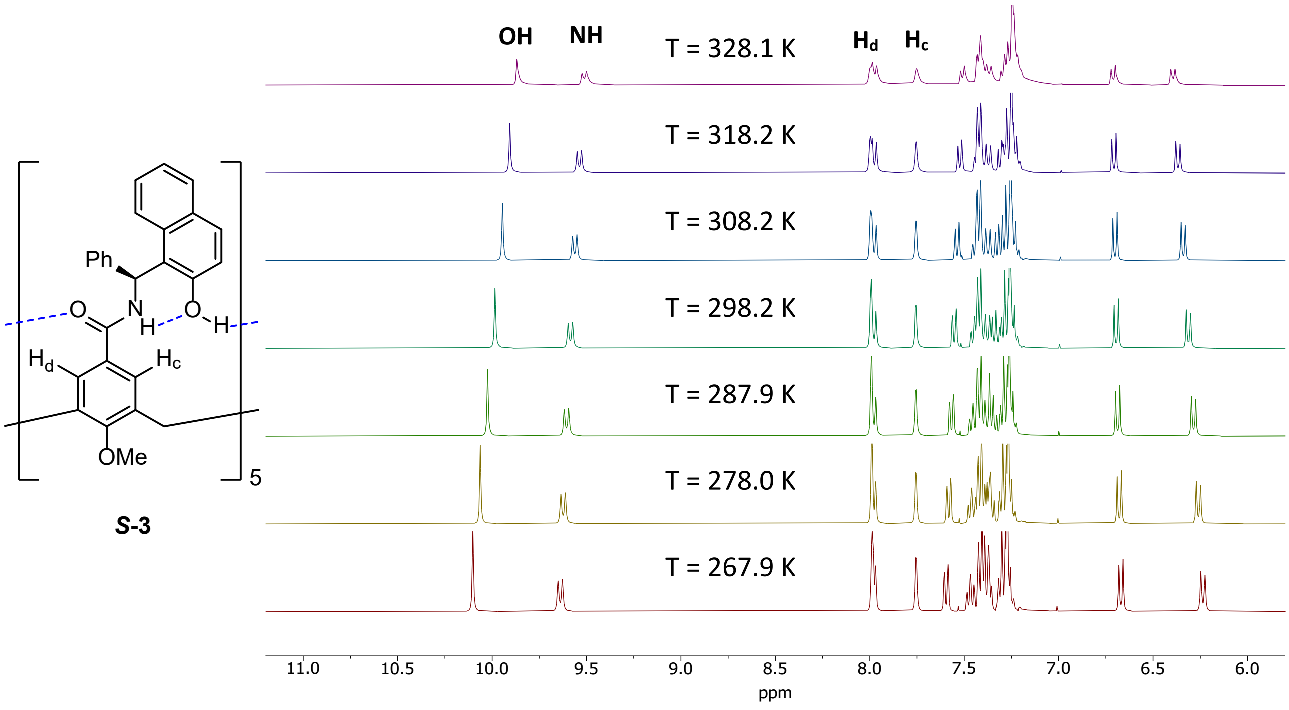

Figure S2. Downfield region of the ^{1}H NMR spectra of **3** in $CDCl_3$ at different temperatures ([**3**] = 1.0 mM).

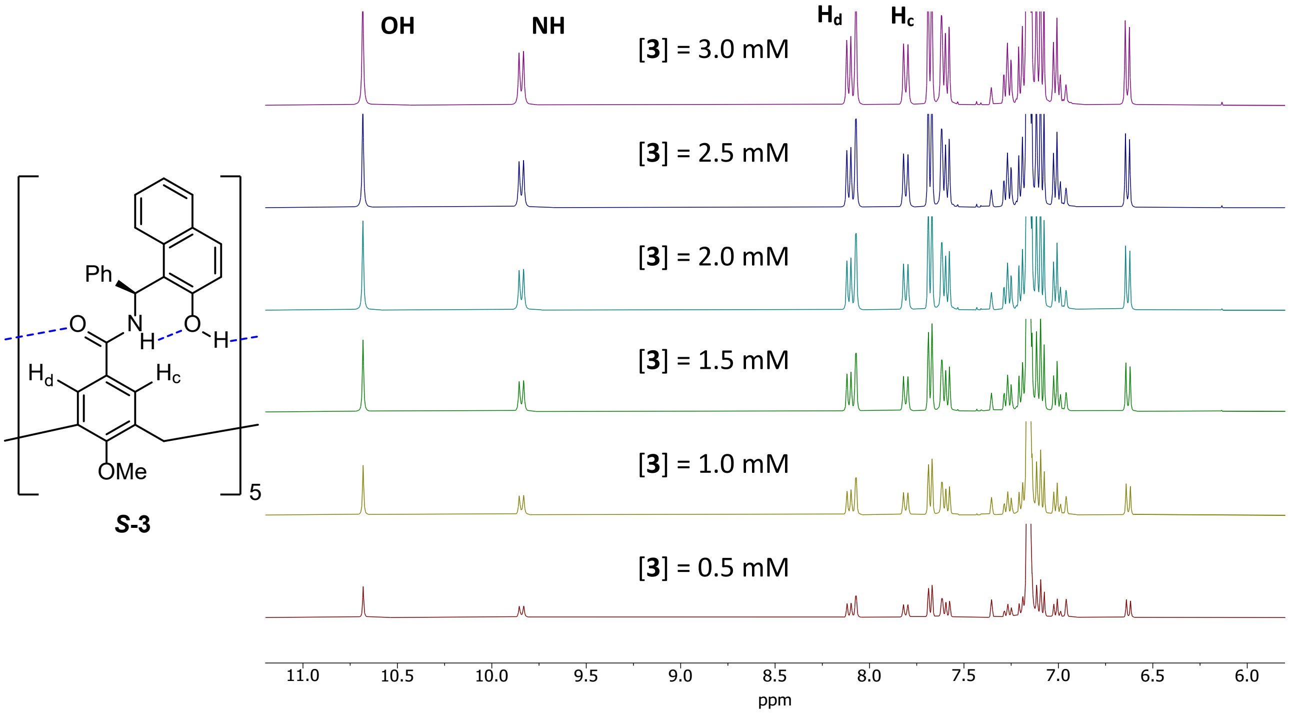

Figure S3. Downfield region of the ^{1}H NMR spectra of cavitand ***S*-3** in C_6D_6 at different concentrations (T = 298 K).

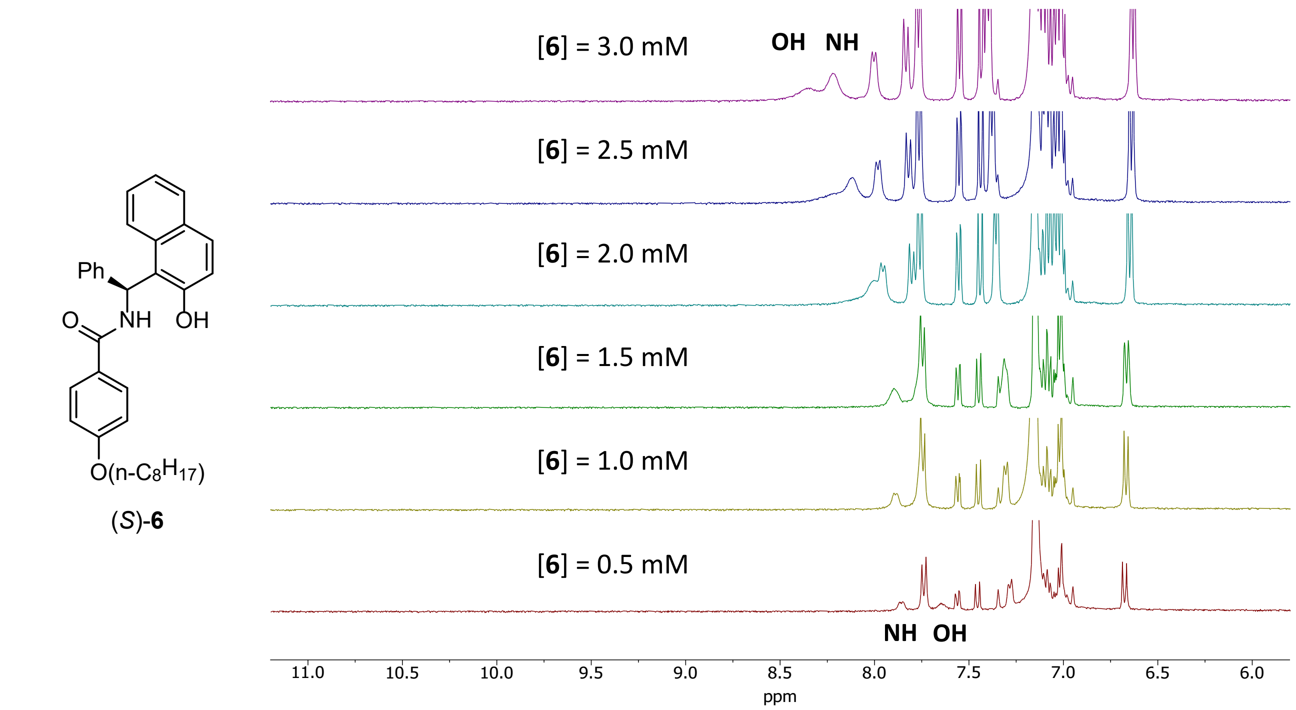

Figure S4. Downfield region of the ^{1}H NMR spectra of model compound (*S*)-**6** in C_6D_6 at different concentrations (T = 298 K).

4.1 2D NMR EXSY and exchange rate calculations

The EXSY experiment[4] was carried out at 298.0 K in $CDCl_3$ to calculate cone flip motion of cavitand **3** (Figure S5). The temperature was controlled by a Bruker BCU-X cooling unit, and was calibrated externally with a CD_3OD standard. Two ^{1}H 2D NOESY spectra were acquired sequentially on a sample of **3** (2.0 mM), one with the desired mixing time (τ_m) and a second reference spectrum at τ_m = 0. Spectra were recorded using the standard non-gradient pulsed, phase sensitive NOESY sequence from Bruker (*noesyph*). Each of the 256 F1 increments was the accumulation of 16 scans. The relaxation delay D1 was set to 4 s. Before Fourier transformation, the FIDs were multiplied by a $\pi/2$ sine square function in both the F2 and the F1 domains.

The rate constants k_1/k_{-1} for the forward and reverse chemical exchange processes were calculated from the integral values of the involved hydrogens (H_a/H_b, H_c/H_d) using the EXSYCalc program (Mestrelab Research)(Figure S6 and Table S1).[5] In a system with two exchanging equivalent sites the forward and reverse 1st order reaction rates are the same ($k_1 = k_{-1}$). The integrations of the diagonal peaks should be equal, as well as the two cross-peaks originating from magnetization exchange. The intrinsic uncertainty of the volume integral values obtained from NMR spectra results in slightly different measurements for k_1 and k_{-1}. Rather than artificially equating the integral values, we obtained an estimation of the rate constant k by averaging the measured k_1 and k_{-1} values. We then obtained the corresponding $\Delta G^‡$ through the Eyring equation (1). For our dataset, the same $\Delta G^‡$ values were obtained by averaging the diagonal and cross-peak integrals and feeding the three unique integration values (diagonal, cross-peaks, and reference diagonal) into EXSYCalc.

$$k = \frac{k_B T}{h} e^{(-\Delta G^‡/RT)} \qquad (1)$$

Of the two available sets of resonances available for the calculation, H_a/H_b and H_c/H_d (Figure S6), the latter overlap with other aromatic resonances and were discarded. For the neighboring methylene protons used for the calculation (H_a/H_b), chemical exchange evolution and through space cross-relaxation (NOE effect) add up. We demonstrated in our previous report that the cross-relaxation component is not very significant, and can be minimized by careful choice of the experiment conditions.[3] For cavitand **3**, we carried out the ESXY experiment in a range of mixing times (τ_m = 300/400/500 ms), obtaining the same barrier within the margin of error. Therefore, the error in the barrier induced by NOE effects is negligible. Because of its molecular size, cavitand **3** falls in the region of negative NOE effect, giving rise to diagonal and cross peaks of the same sign (this was corroborated by complementary ROESY experiments). Therefore, any residual NOE contribution will lead to an overestimation of the rate constant. The EXSY calculation will, in the worst case scenario, underestimate the exchange barrier.

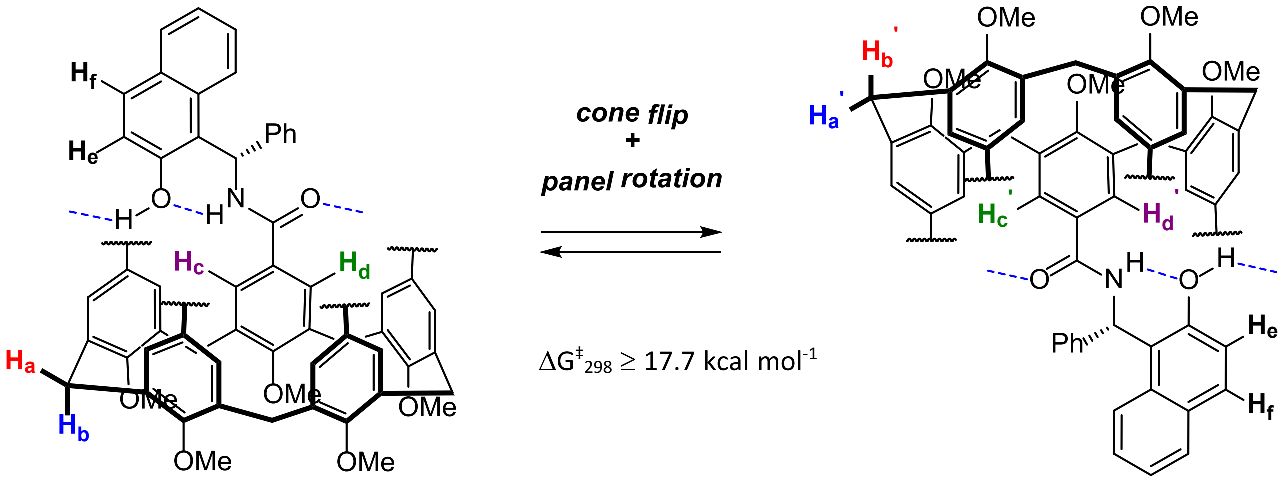

Figure S5. Representation of the bowl inversion motion in **3**, and the associated barrier in $CDCl_3$.

^{1}H 2D EXSY (400 MHz), $CDCl_3$, 298.0 K

τ_m = 500 ms τ_m = 0 ms

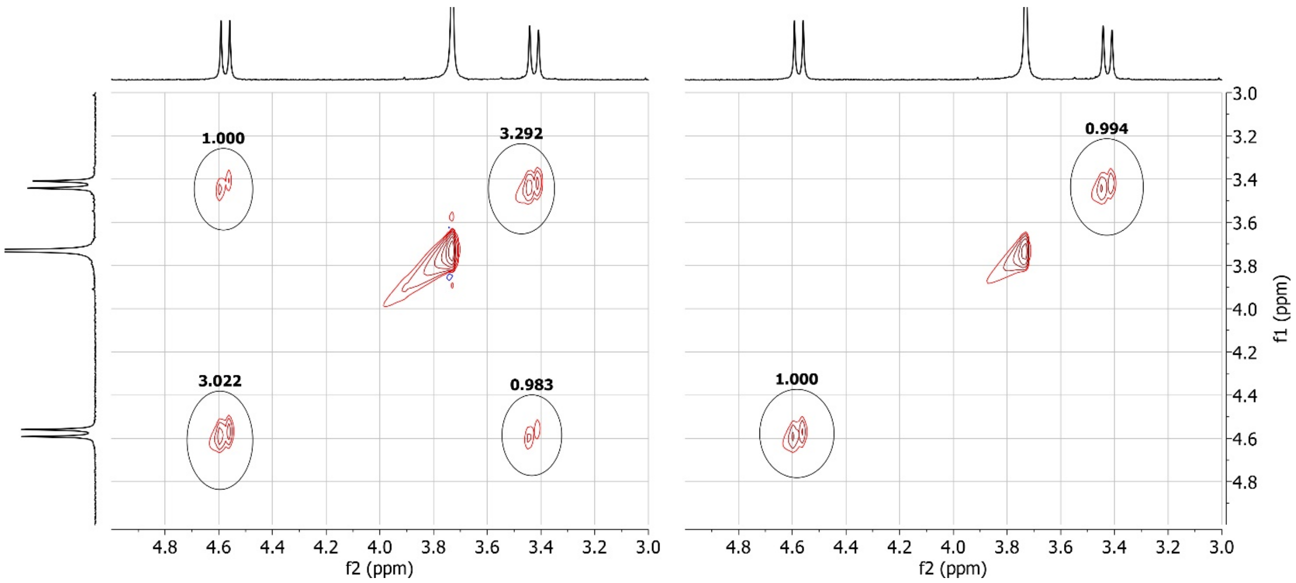

Figure S6. ^{1}H 2D EXSY spectra acquired to calculate the cone inversion exchange barriers of **3** in $CDCl_3$ ([**3**] = 2.0 mM).

Table S1. Integral values (*I*) and the resulting rate constants in $CDCl_3$ obtained with EXSYCalc.

I		I_{ref}	τ_m ms	T K	k_1 s^{-1}	k_{-1} s^{-1}	k_{mean} s^{-1}	$\Delta G^{\ddagger}$ kcal·mol^{-1}
3.022	0.983	1.000	500	298.0	0.643	0.658	0.651	17.7±0.2[a]
1.000	3.292	0.994						

[a] The errors are estimated by calculating the extreme $\Delta G^{\ddagger}$ values obtained with a 5% error bias in integration and 1% deviation in temperature.

5 ^{1}H NMR titration experiments.

For each host guest pair, a 1 mM stock solution of ***R*-3** or ***S*-3** in $CDCl_3$ was prepared (solution **A**). A solution of the appropriate guest (**G1-G10**) in **A** was then prepared (15-60 mM, solution **B**). In a 3 mm NMR tube, an initial volume of solution **A** (175 -200 μL) was titrated with increasing amounts of solution **B**. A ^{1}H NMR spectrum was recorded after each addition. After referencing each spectrum, the δ values for protons H_e and H_f (Figure S5) were extracted, and fit to a binding isotherm using Bindfit.[14,15] For each titration, stacked plots are include showing the evolution of the fitted host resonances (H_e, H_f) and the Me_3N resonance of the guest.

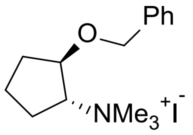

(1*R*,2*R*)-**G1**

***R*-3** / (1*R*,2*R*)-**G1**	K_a = 129±18 M^{-1}	***S*-3** / (1*R*,2*R*)-**G1**	K_a = 99±6 M^{-1}
	δ_{HG} = 6.713 / 6.337		δ_{HG} = 6.783 / 6.427

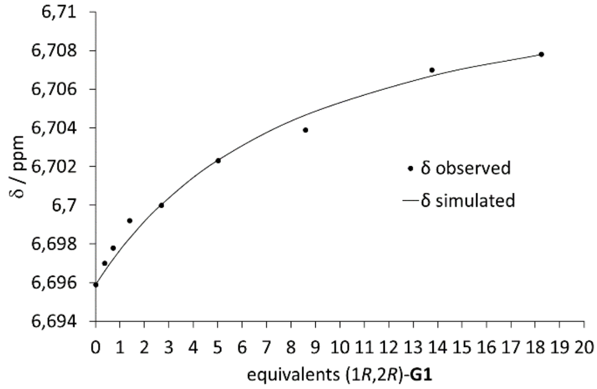

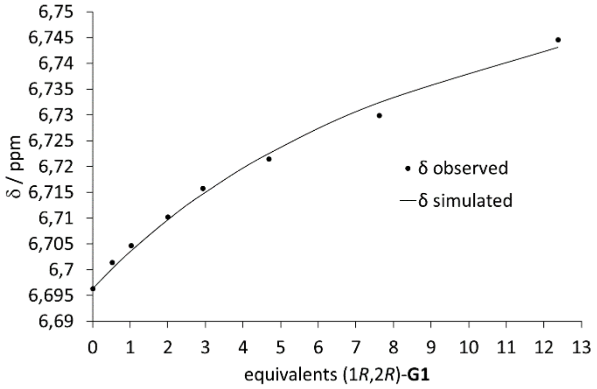

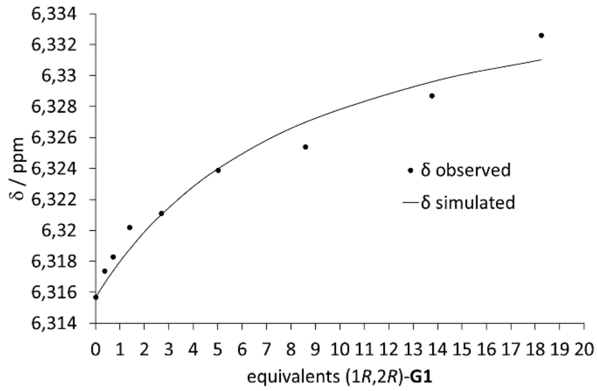

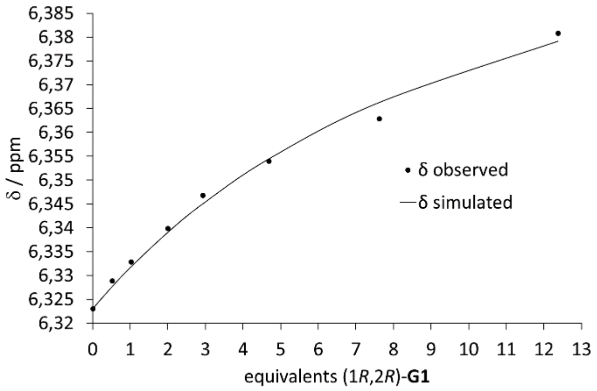

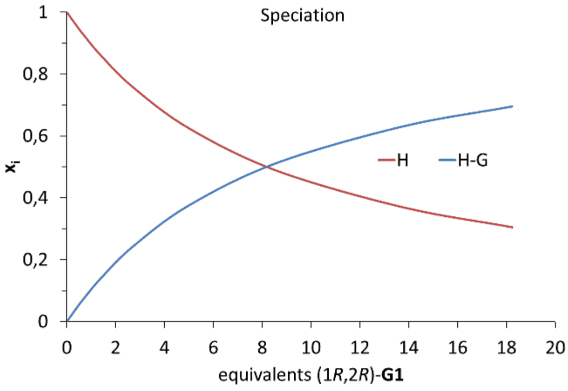

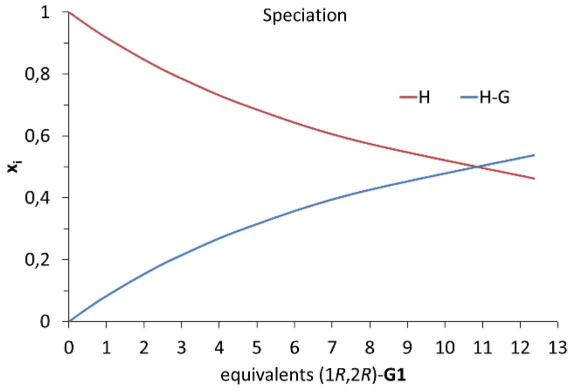

^{1}H NMR titration of ***R*-3** / (1*R*,2*R*)-**G1**

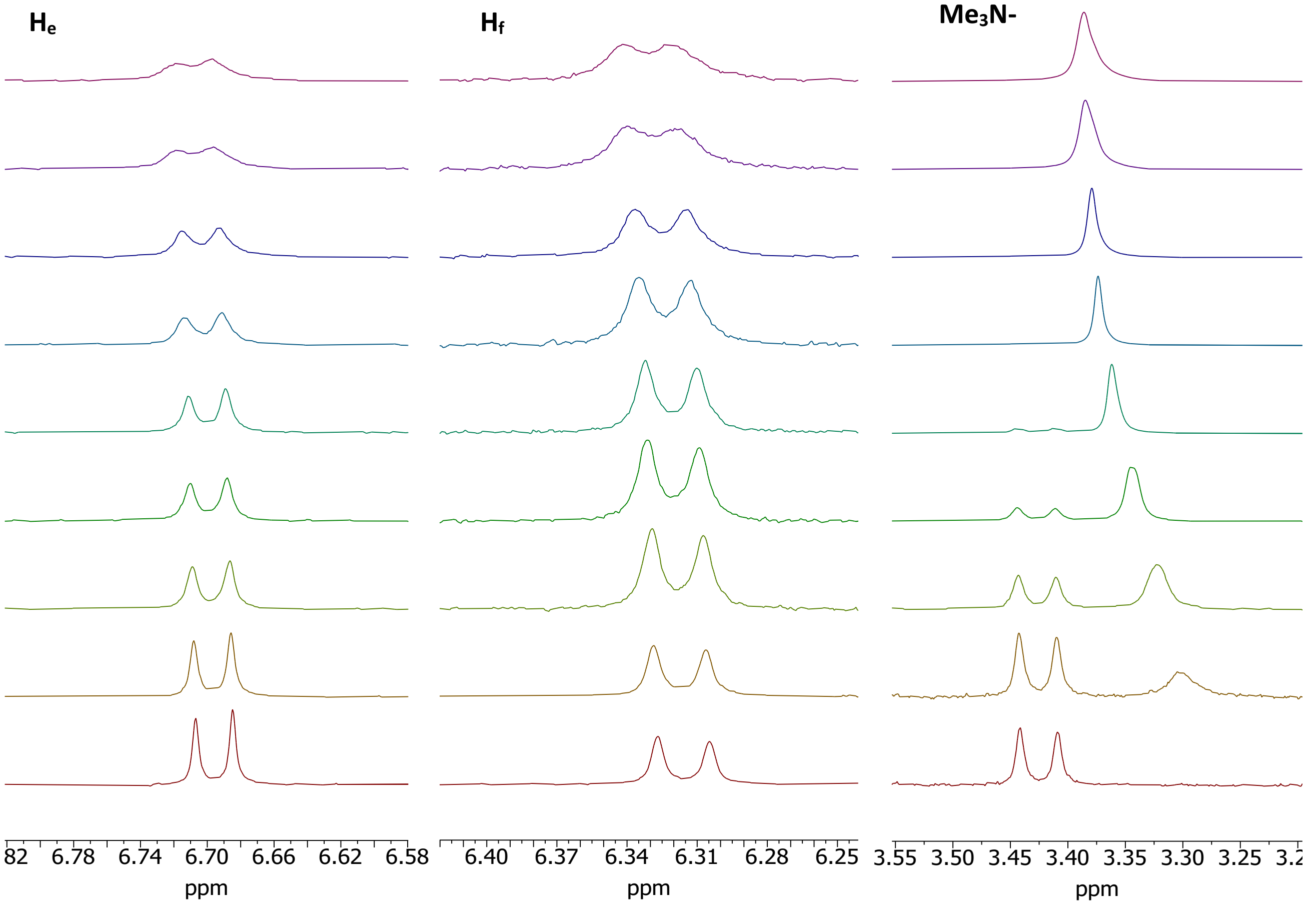

^{1}H NMR titration of ***S*-3** / (1*R*,2*R*)-**G1**

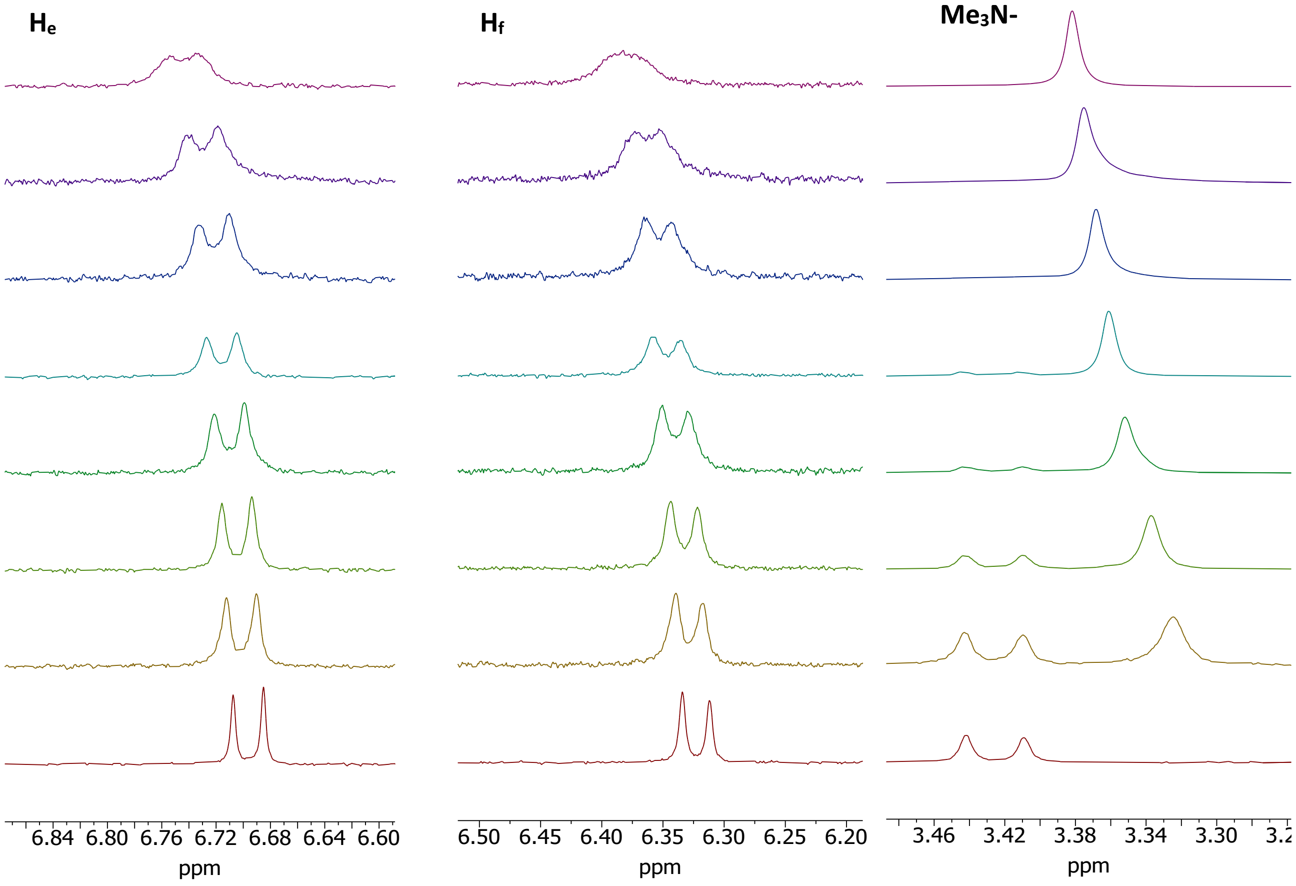

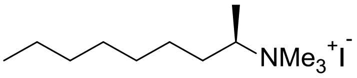

(*R*)-**G2**

***R*-3** / (*R*)-**G2**	K_a = 192±12 M^{-1}	***S*-3** / (*R*)-**G2**	K_a = 42±3 M^{-1}
	δ_{HG} = 6.715 / 6.350		δ_{HG} = 6.722 / 6.375

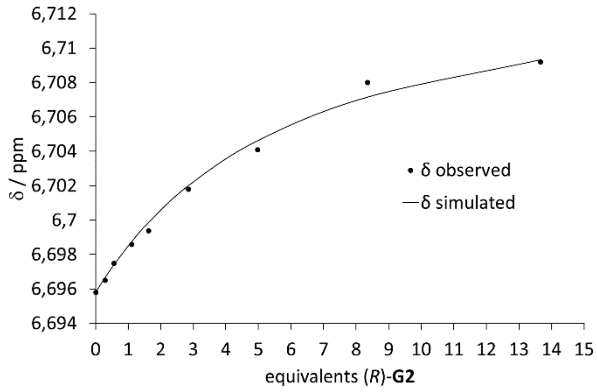

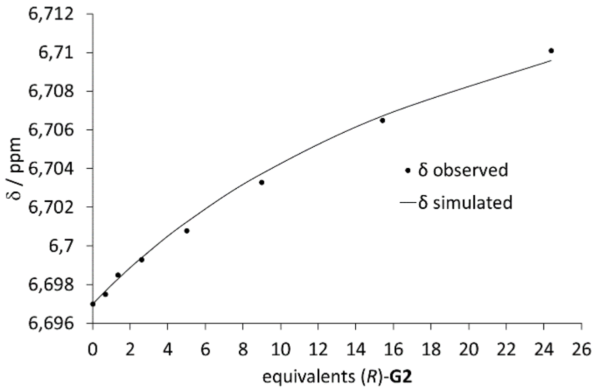

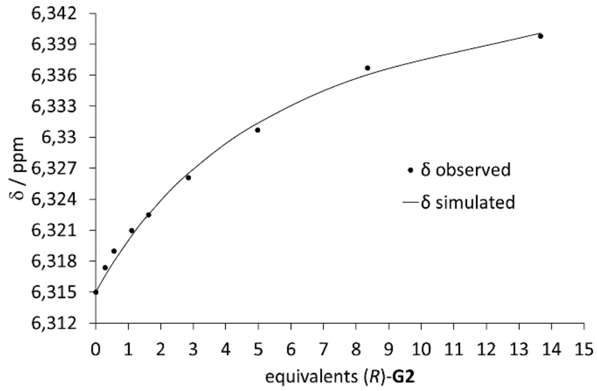

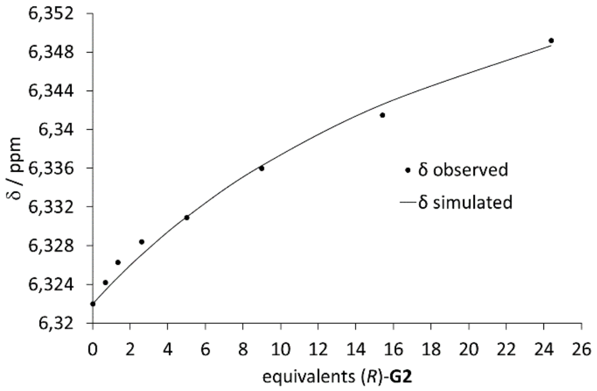

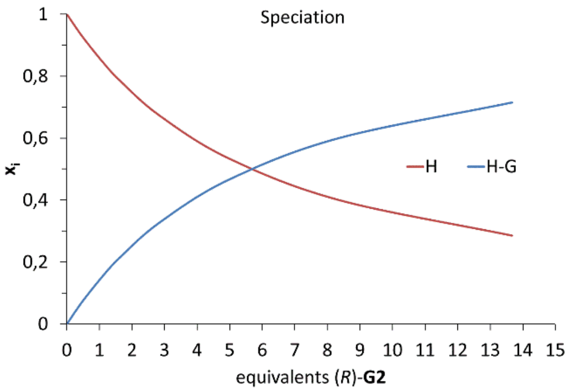

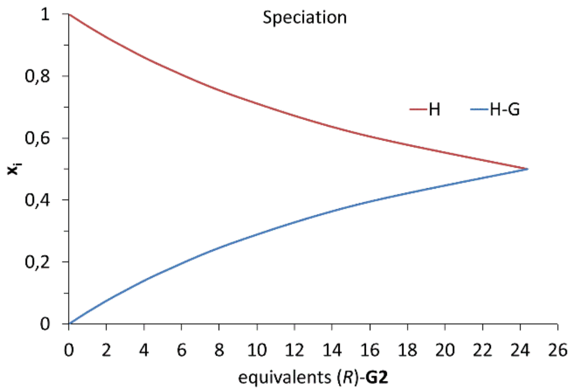

^{1}H NMR titration of ***R*-3** / (*R*)-**G2**

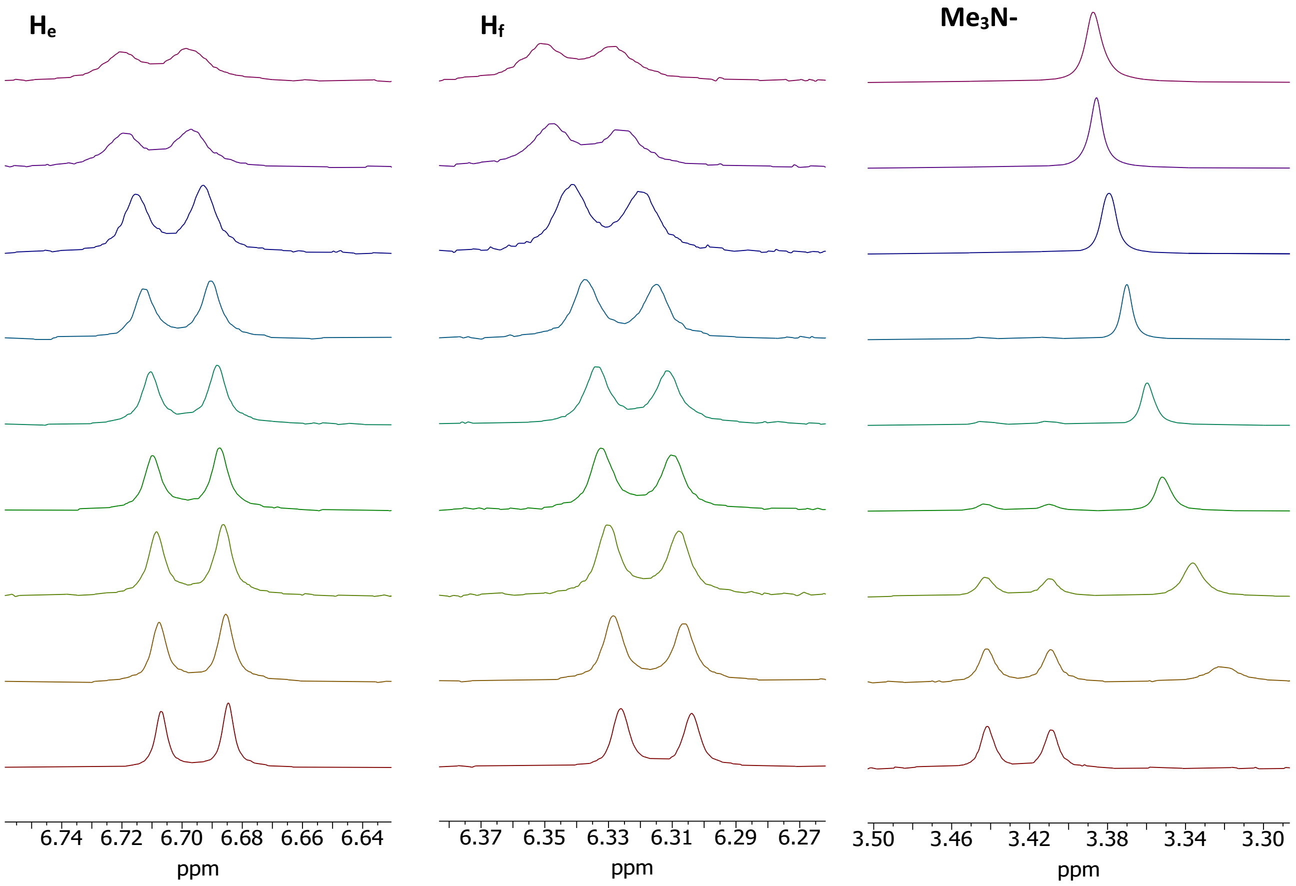

^{1}H NMR titration of ***S*-3** / (*R*)-**G2**

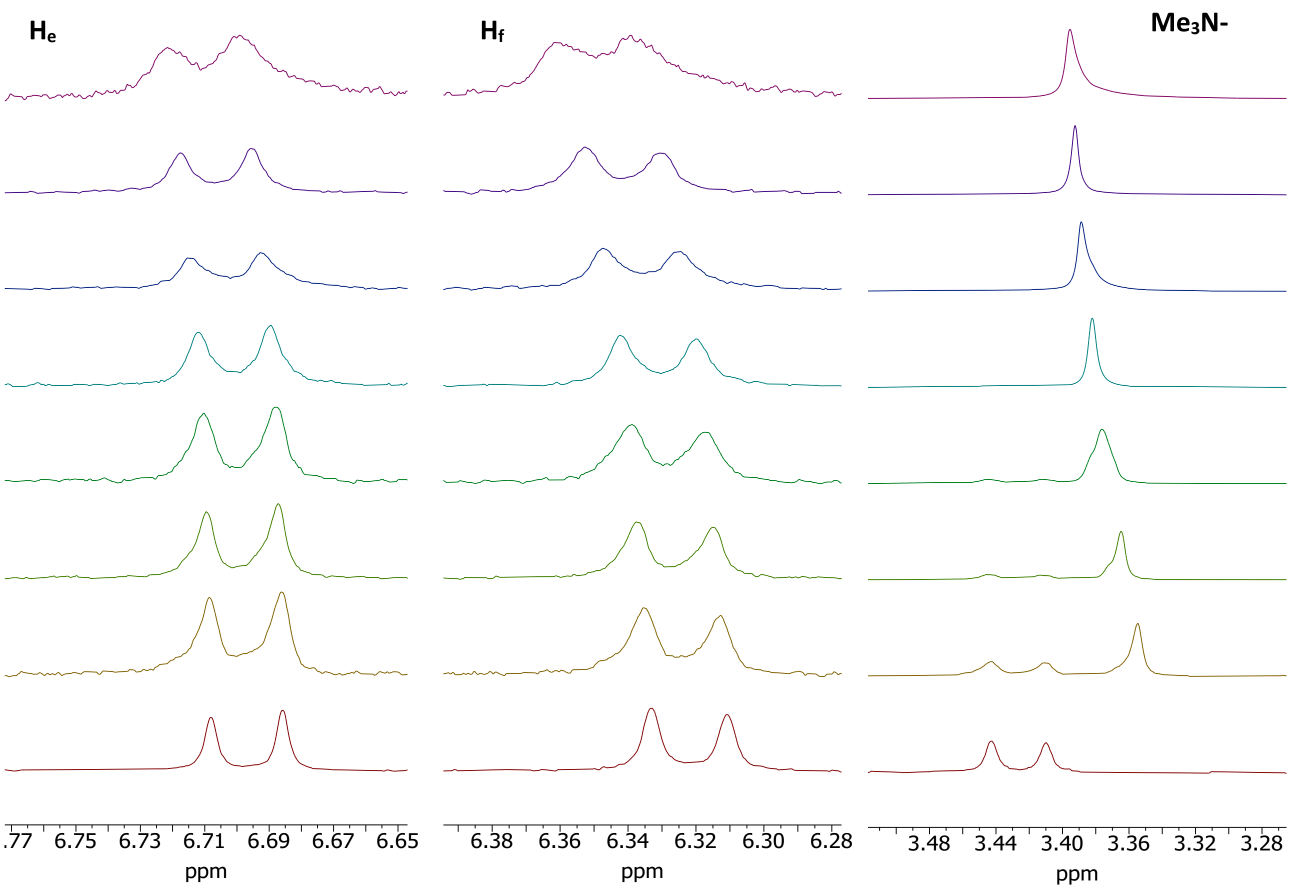

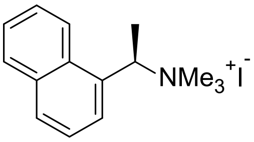

(*R*)-**G3**

***R*-3** / (*R*)-**G3**	K_a = 31±3 M^{-1}	***S*-3** / (*R*)-**G3**	K_a = 587±73 M^{-1}
	δ_{HG} = 6.748 / 6.402		δ_{HG} = 6.696 / 6.348

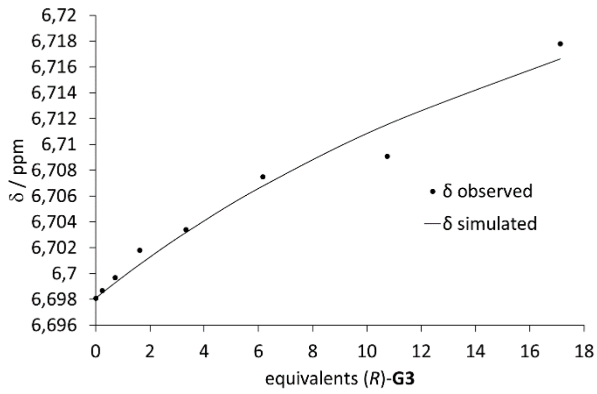

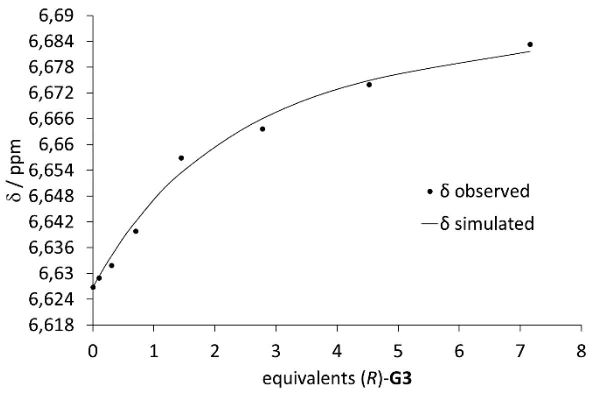

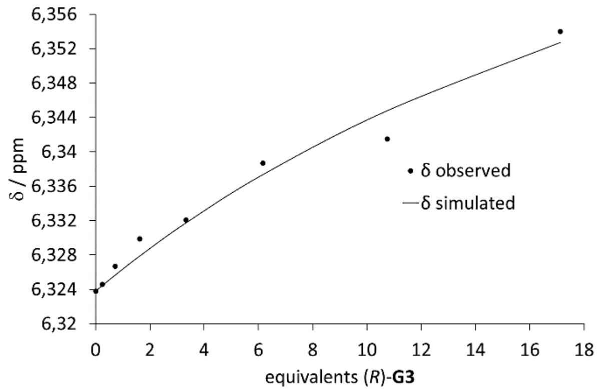

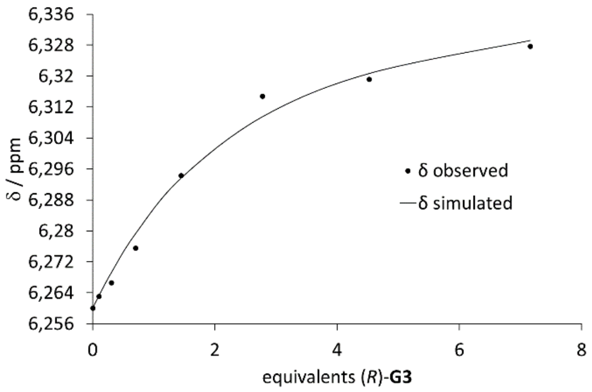

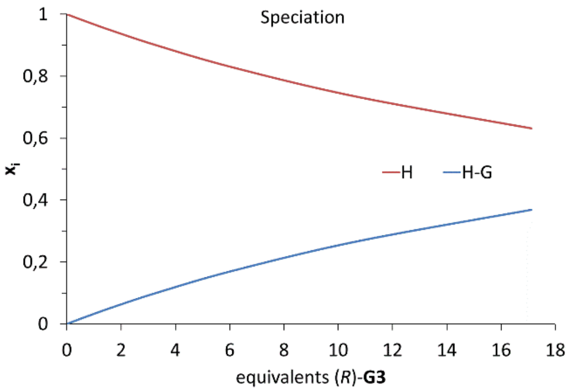

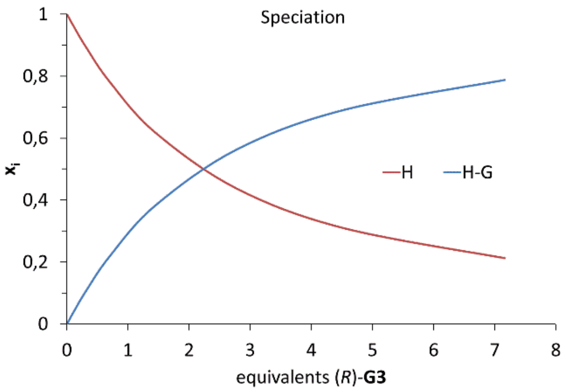

^{1}H NMR titration of ***R*-3** / (*R*)-**G3**

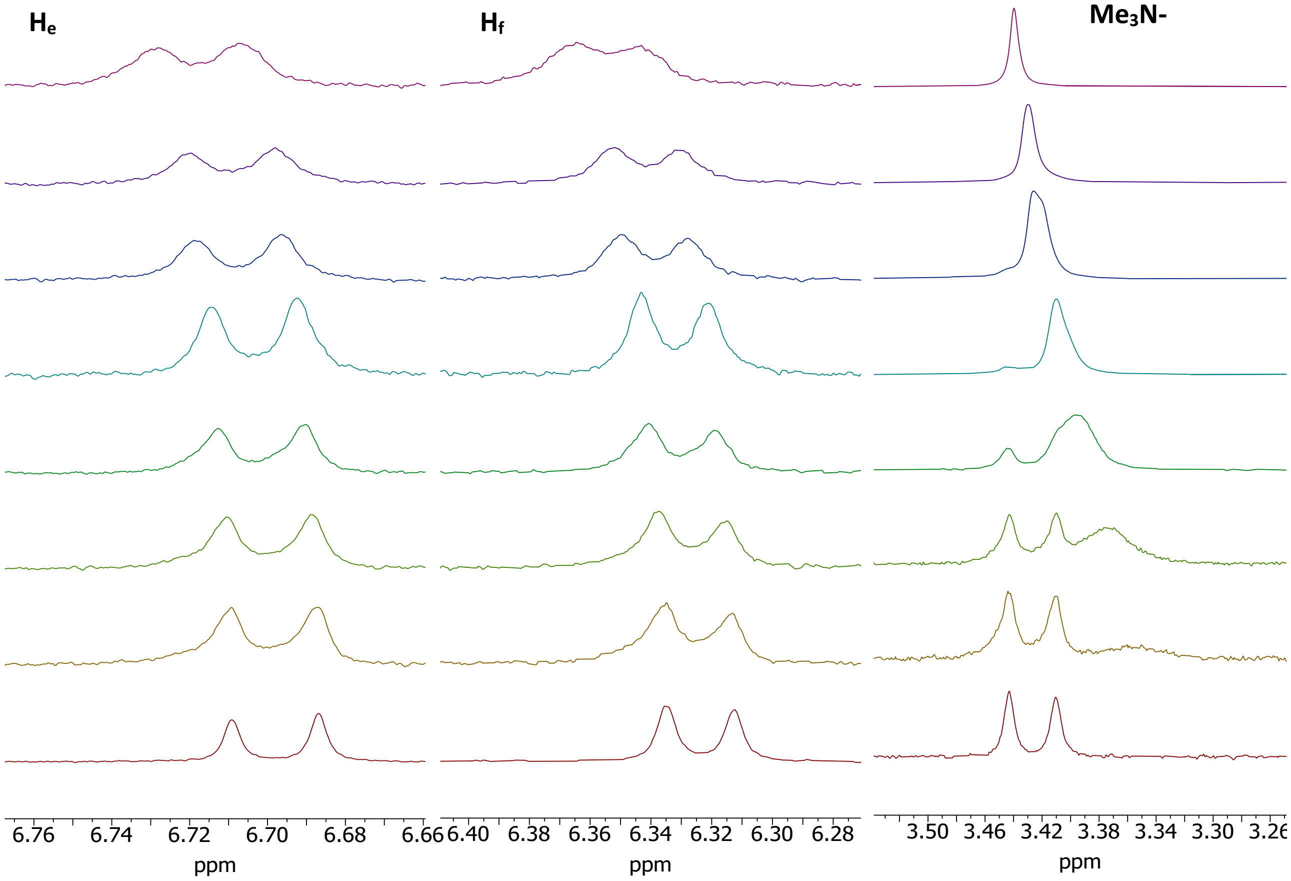

^{1}H NMR titration of ***S*-3** / (*R*)-**G3**

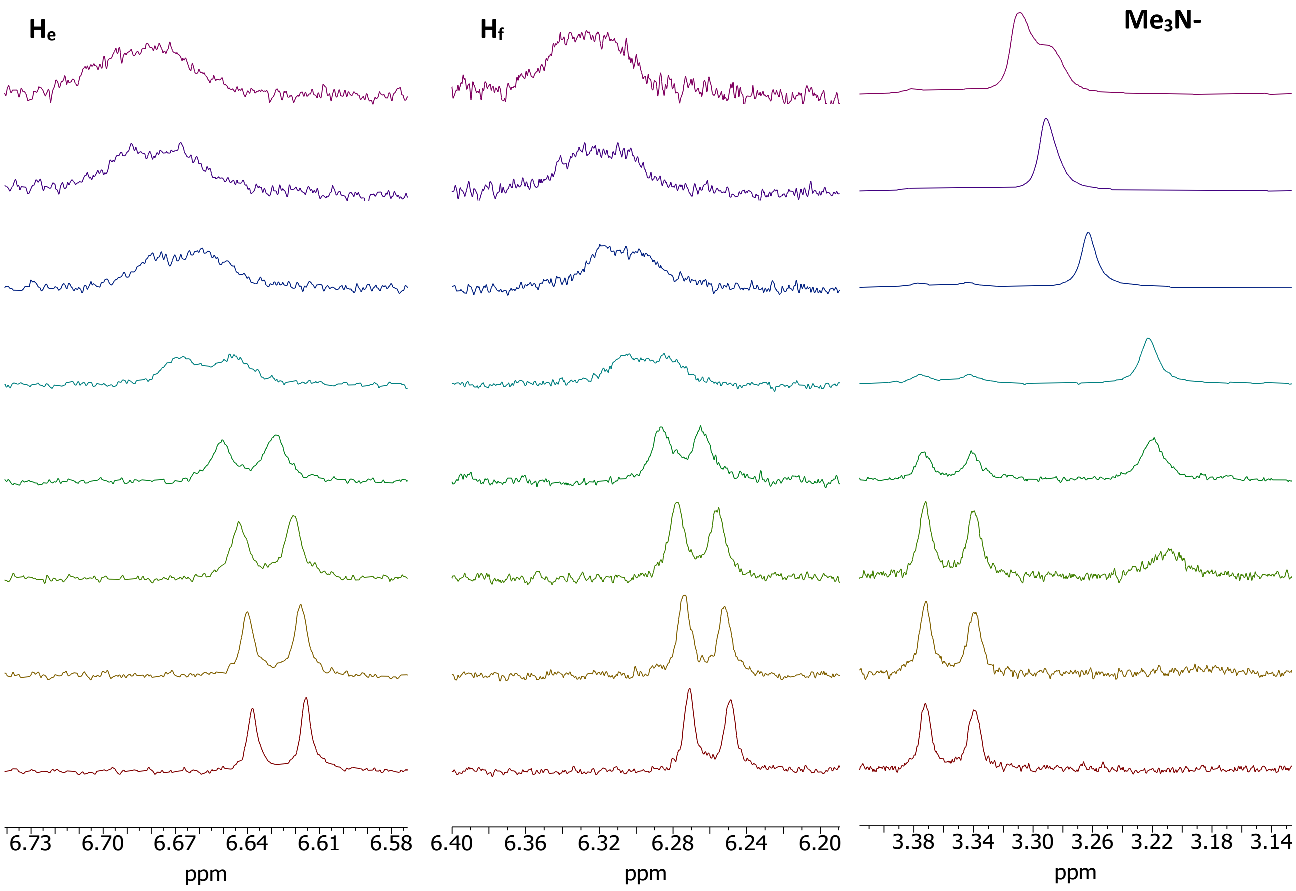

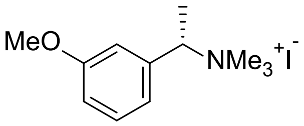

(*S*)-**G4**

***R*-3** / (*S*)-**G4**	K_a = 106±15 M^{-1}	***S*-3** / (*S*)-**G4**	K_a = 32±3 M^{-1}
	δ_{HG} = 6.720 / 6.354		δ_{HG} = 6.728 / 6.380

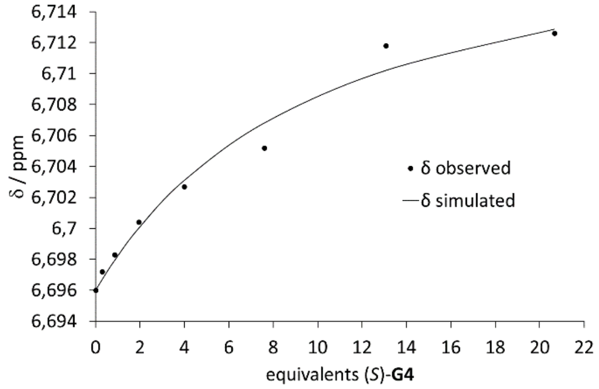

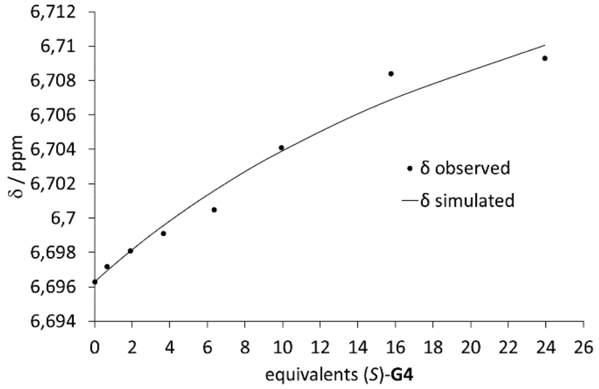

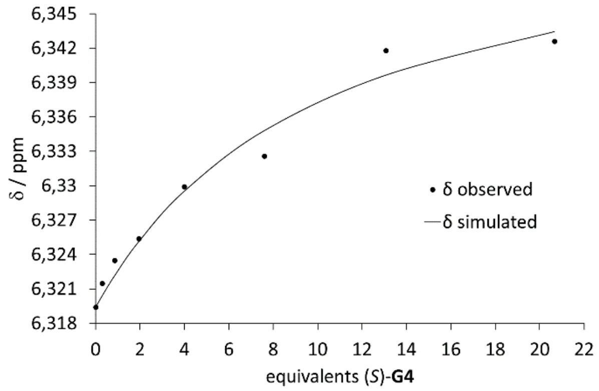

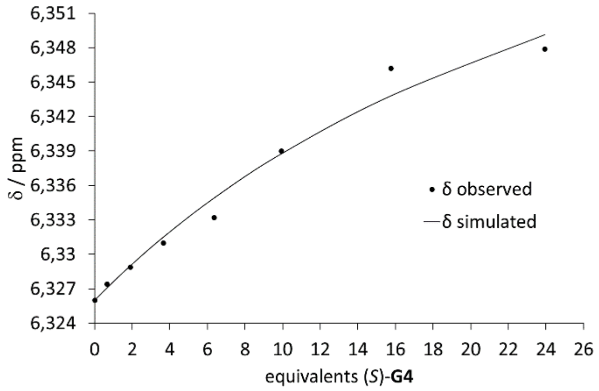

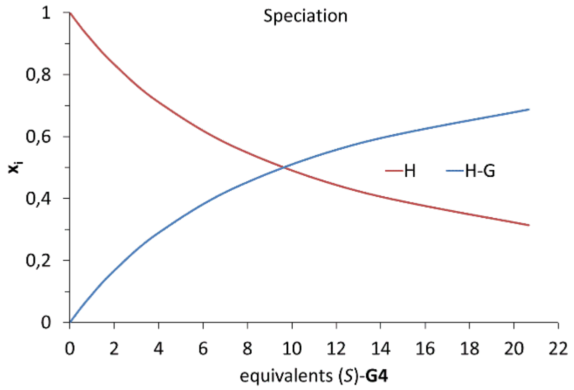

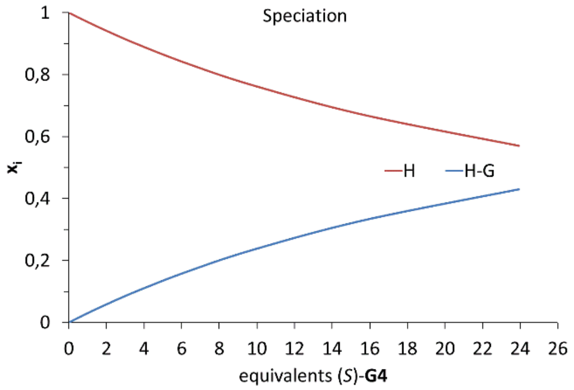

^{1}H NMR titration of ***R*-3** / (*S*)-**G4**

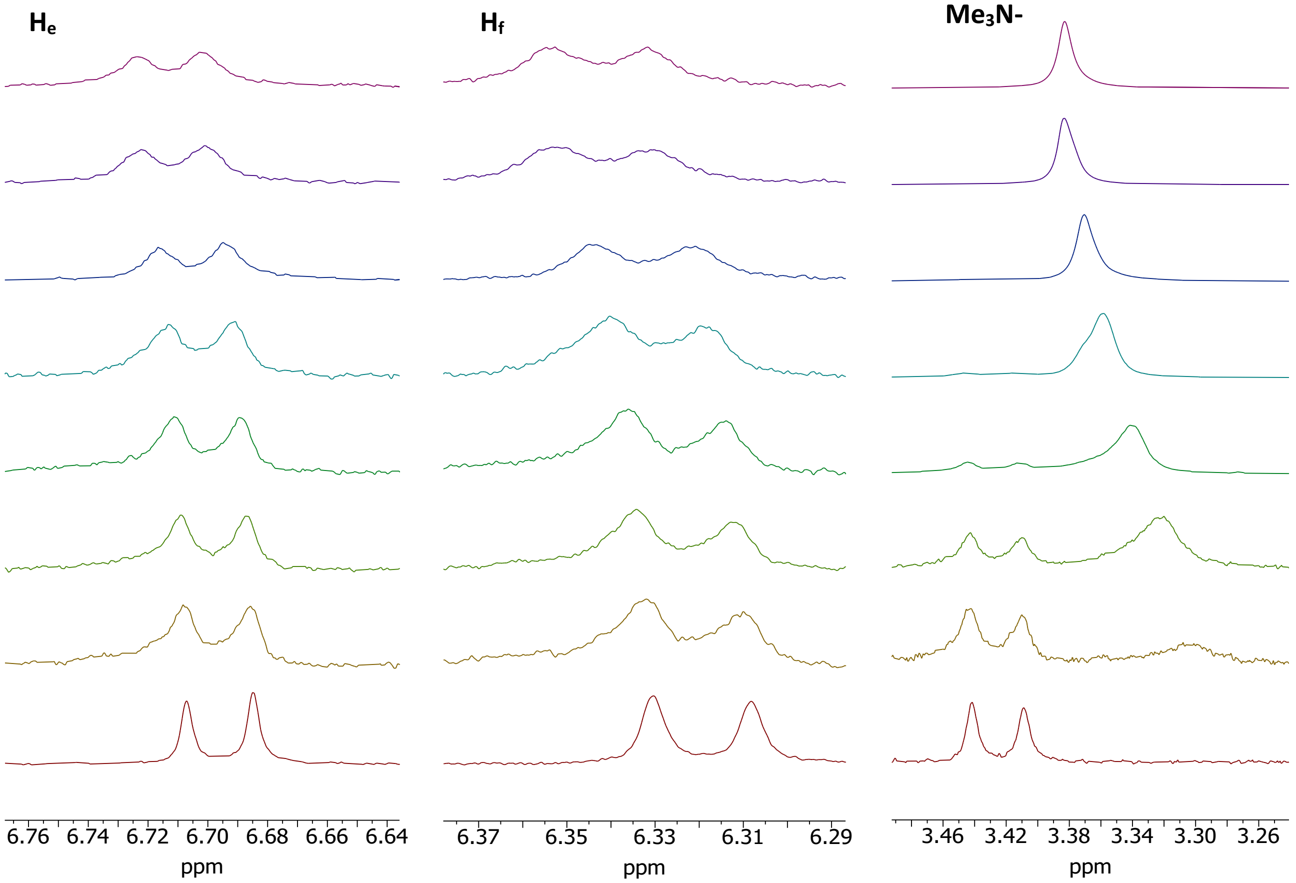

^{1}H NMR titration of ***S*-3** / (*S*)-**G4**

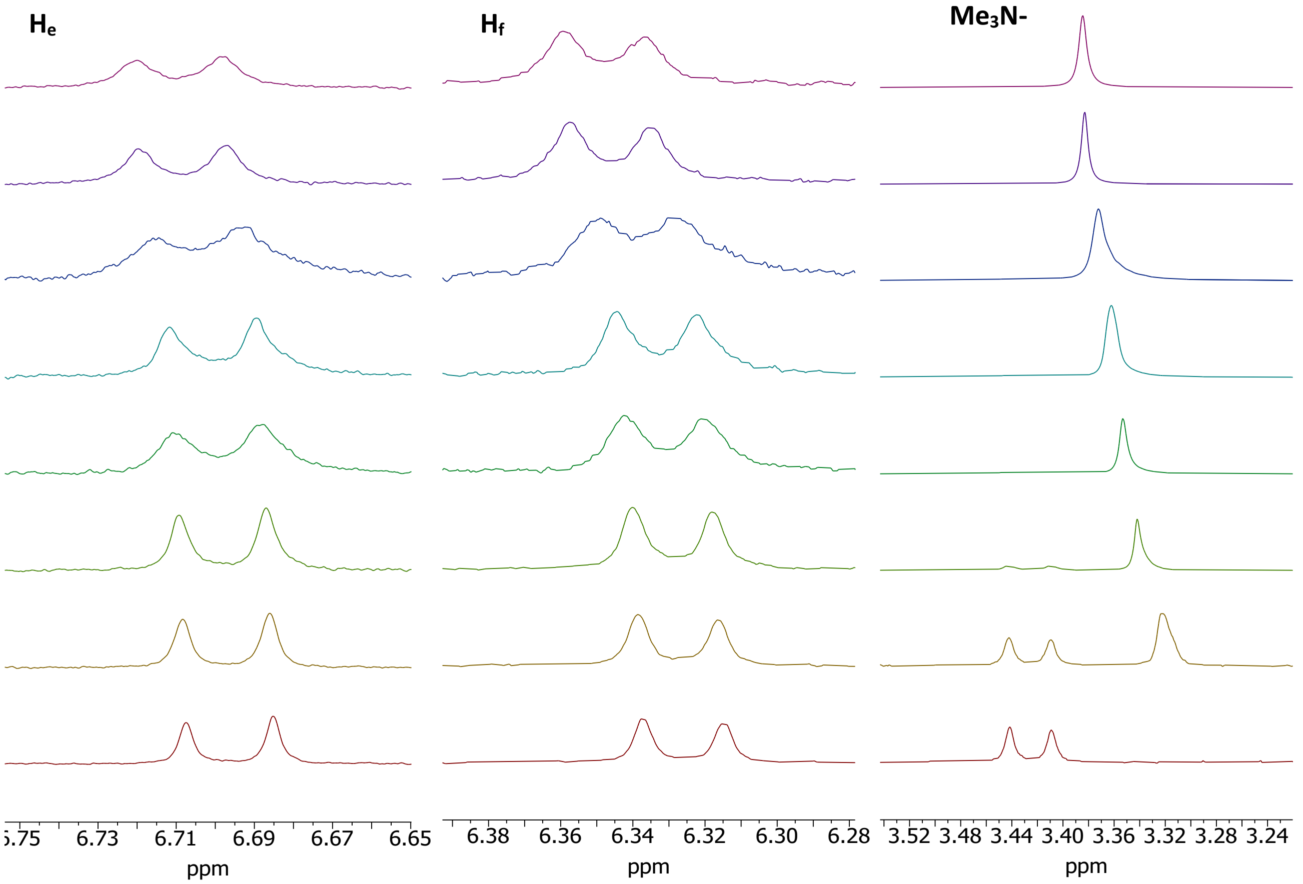

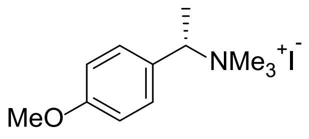

(*S*)-**G5**

***R*-3** / (*S*)-**G5** K_a = 414±21 M^{-1}	***S*-3** / (*S*)-**G5** K_a = 200±21 M^{-1}
δ_{HG} = 6.717 / 6.359	δ_{HG} = 6.764 / 6.414

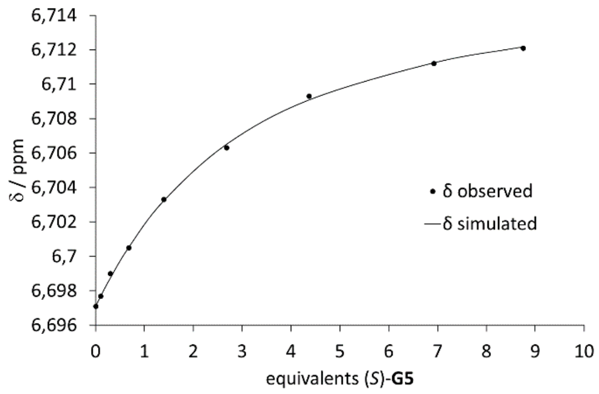

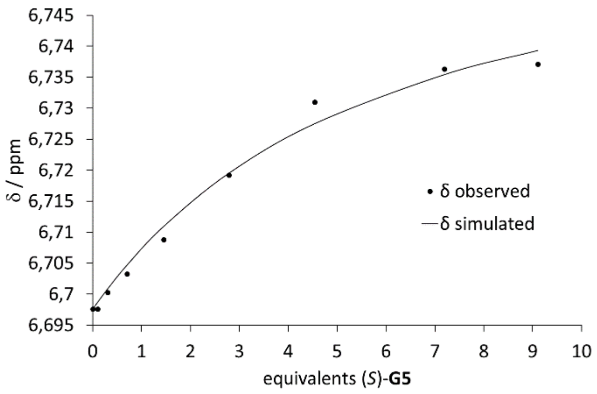

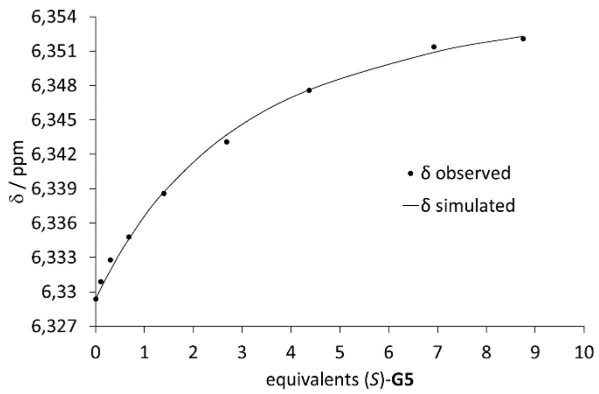

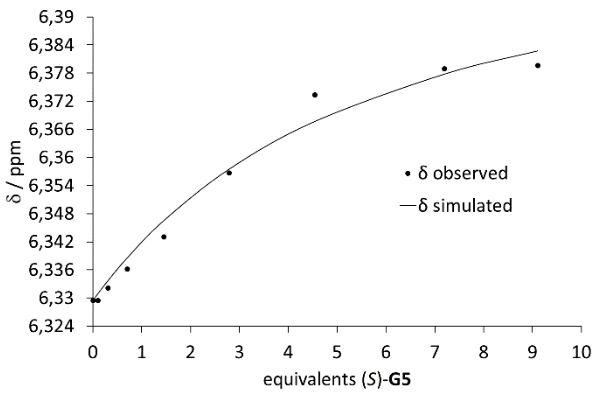

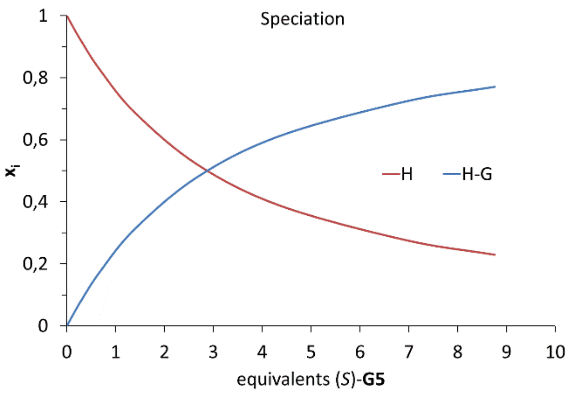

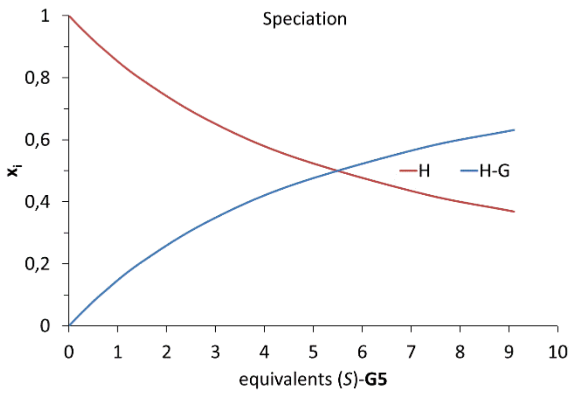

^{1}H NMR titration of ***R*-3** / (*S*)-**G5**

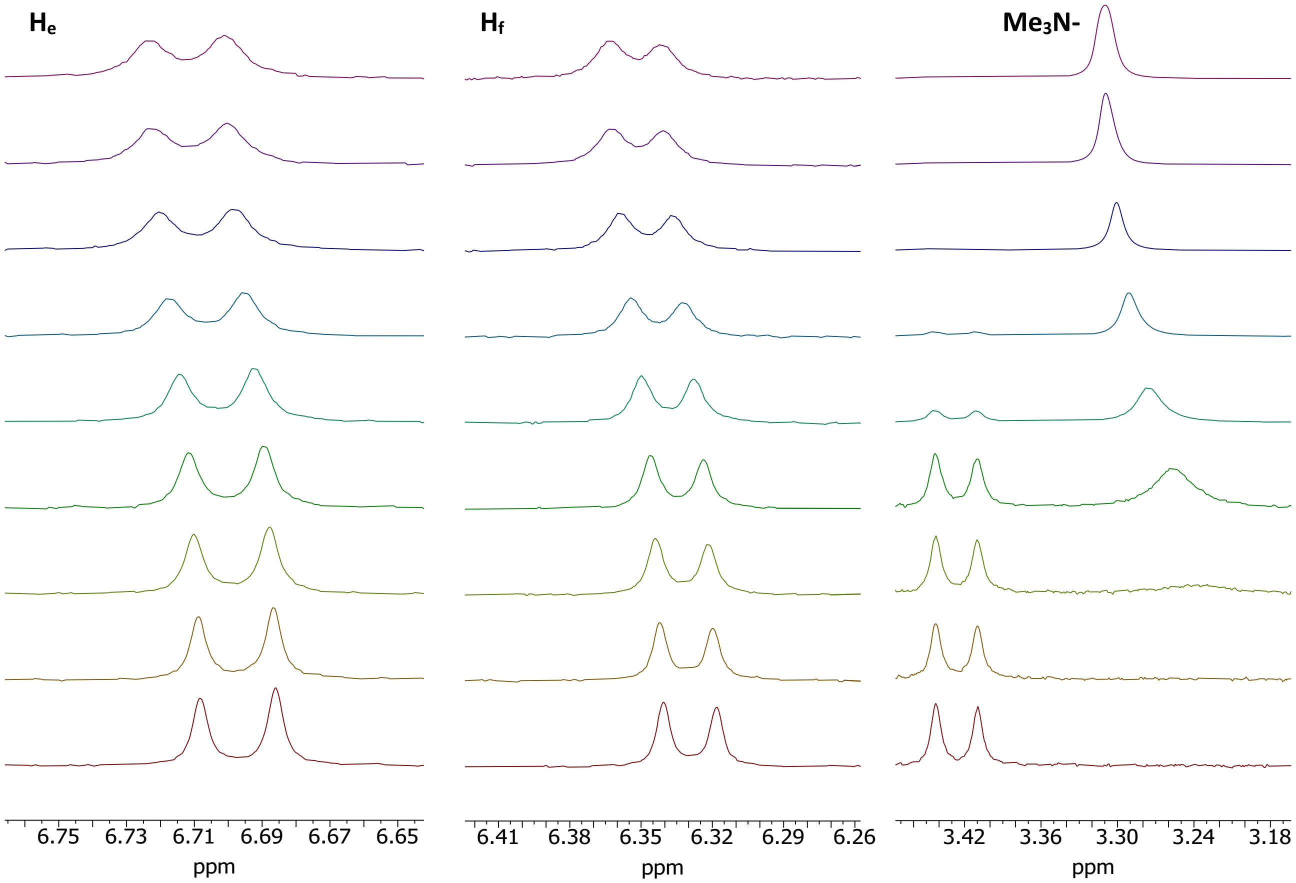

^{1}H NMR titration of ***S*-3** / (*S*)-**G5**

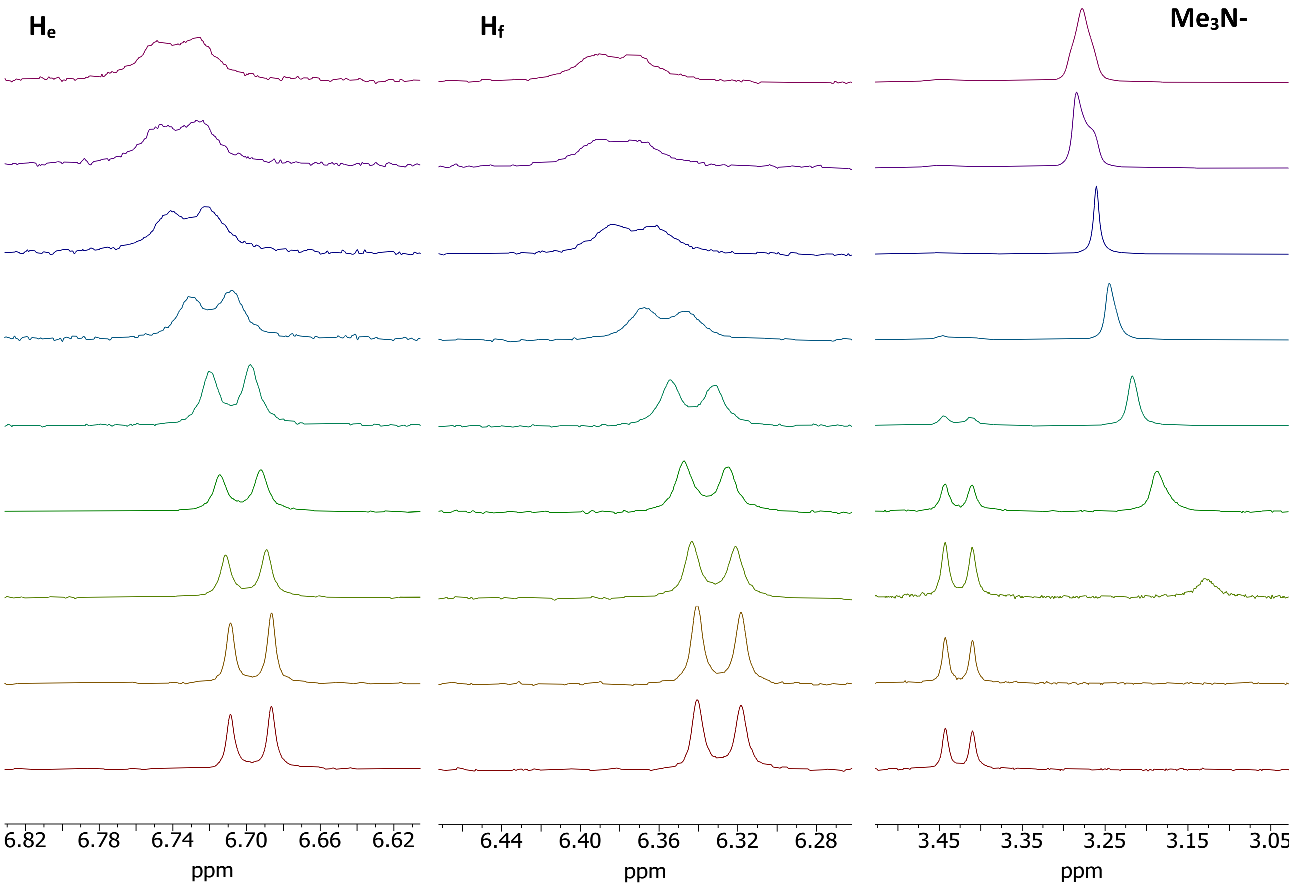

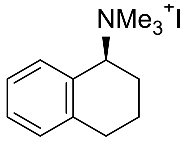

(*S*)-**G6**

***R*-3** / (*S*)-**G6**	K_a = 890±48 M^{-1}	***S*-3** / (*S*)-**G6**	K_a = 1128±100 M^{-1}
	δ_{HG} = 6.965 / 6.692		δ_{HG} = 6.738 / 6.405

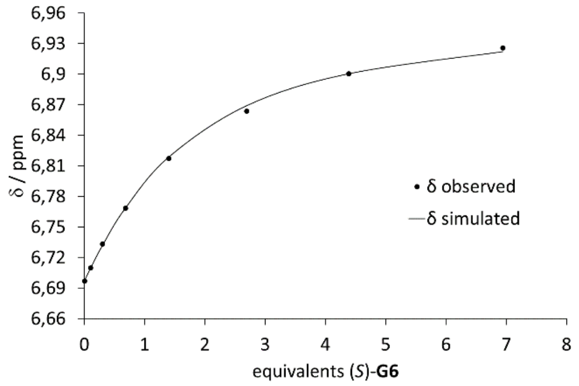

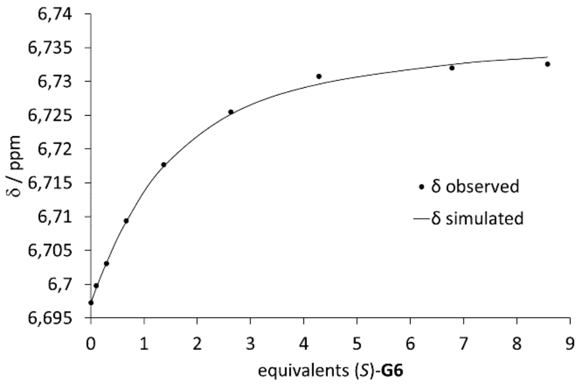

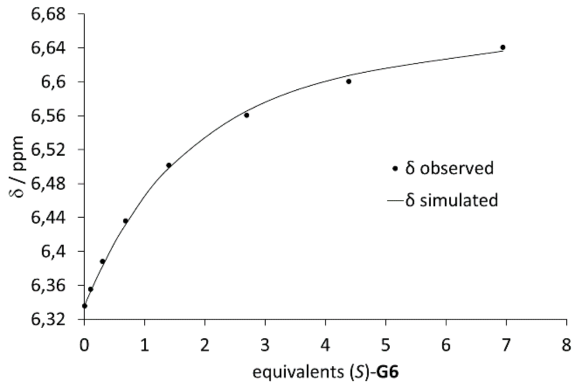

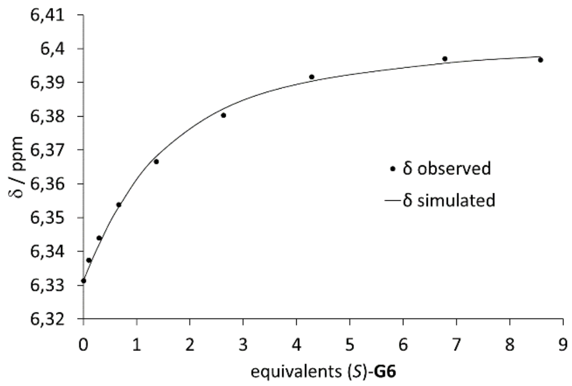

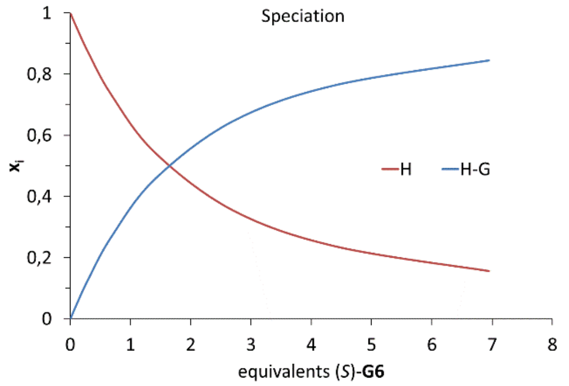

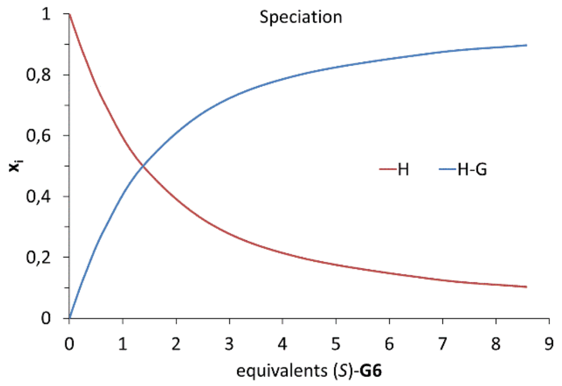

^{1}H NMR titration of ***R*-3** / (*S*)-**G6**

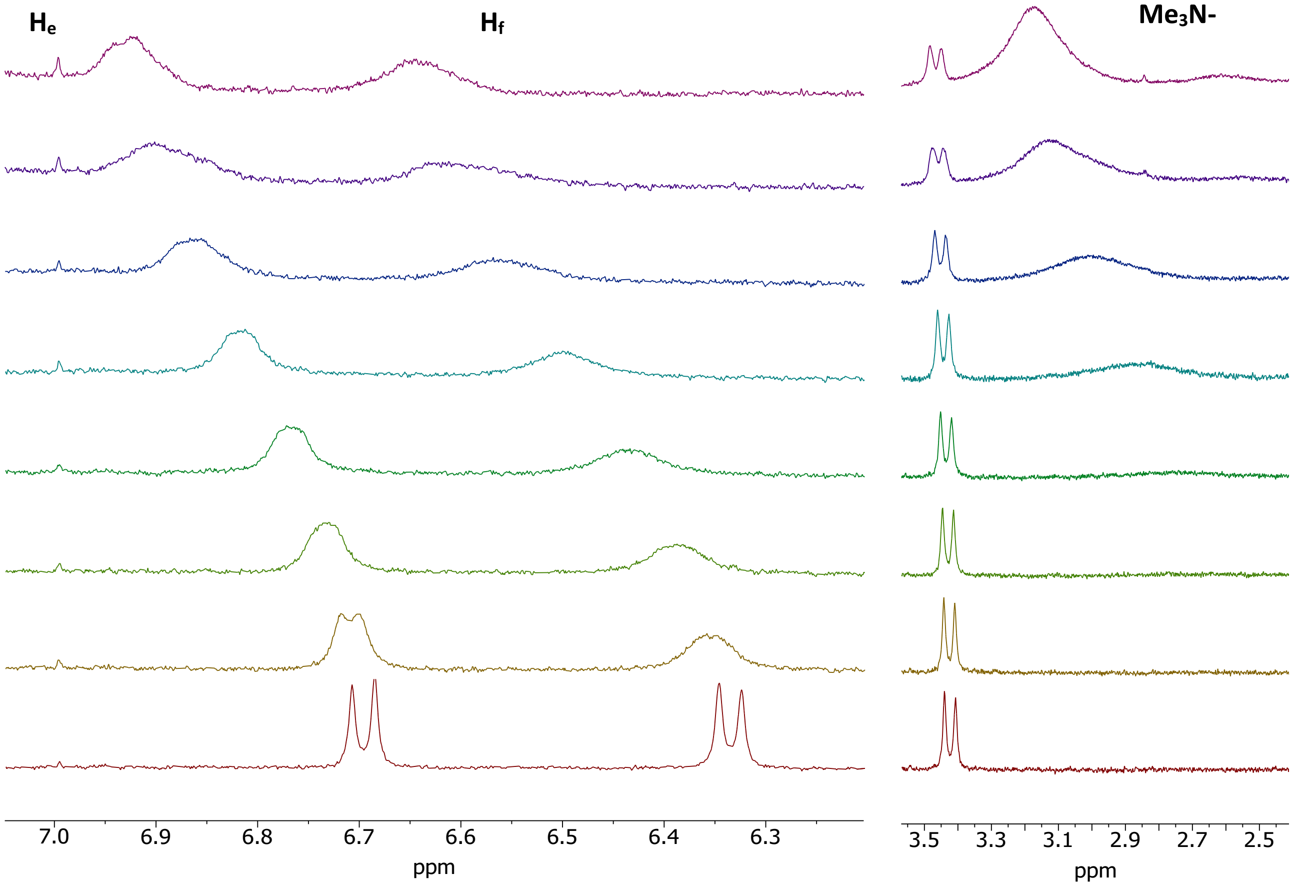

^{1}H NMR titration of ***S*-3** / (*S*)-**G6**

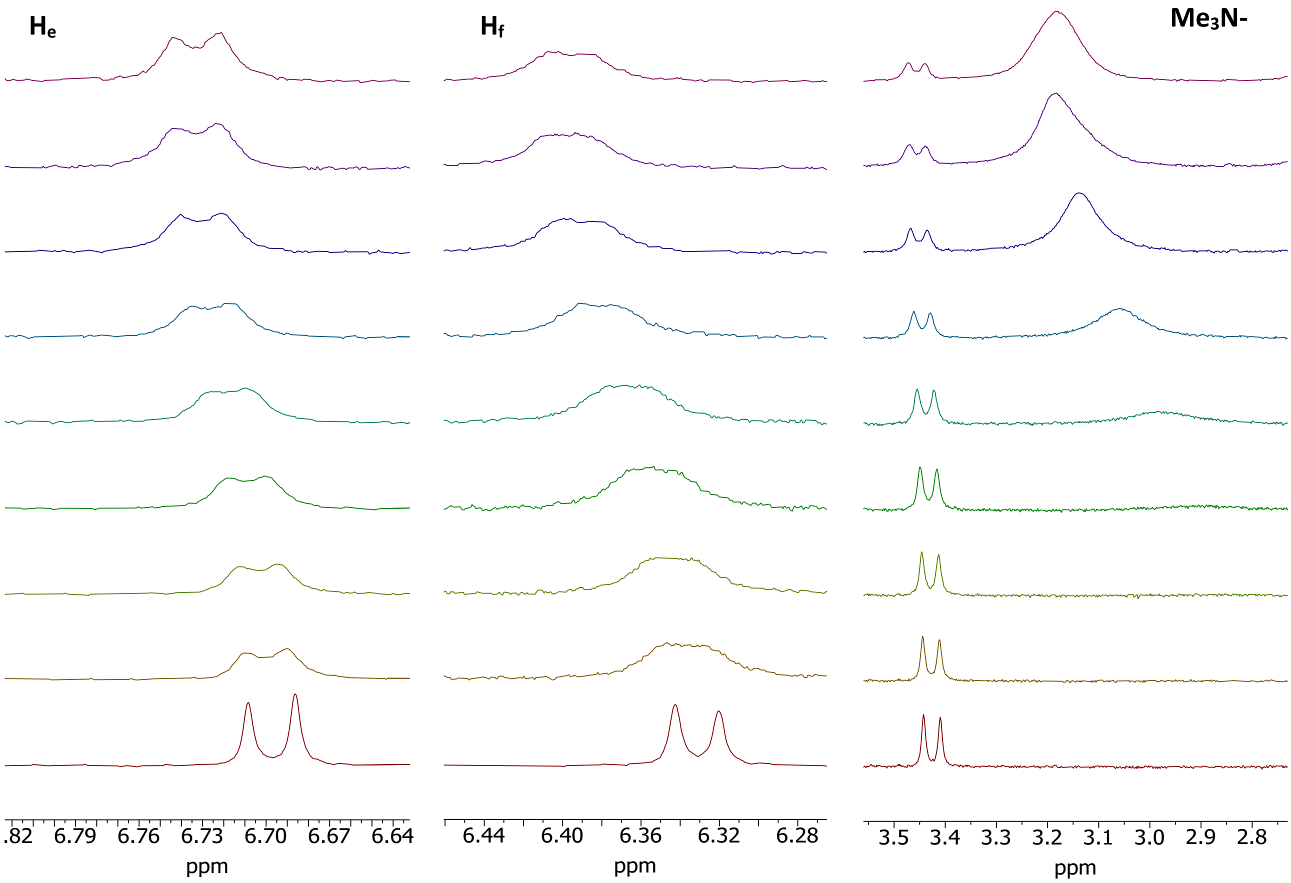

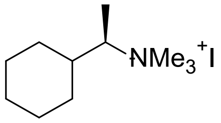

(*R*)-**G7**

***R*-3** / (*R*)-**G7**	K_a = 607±88 M^{-1}	***S*-3** / (*R*)-**G7**	K_a = 1220±118 M^{-1}
	δ_{HG} = 6.730 / 6.392		δ_{HG} = 6.728 / 6.371

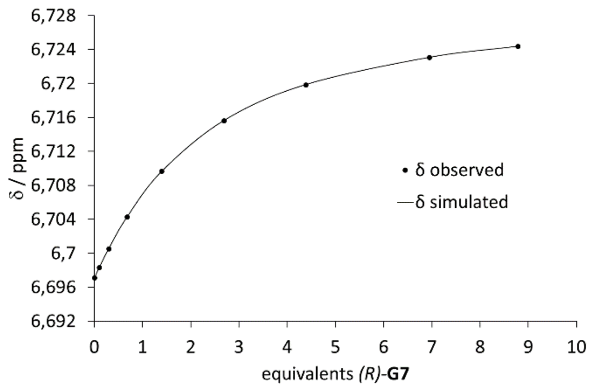

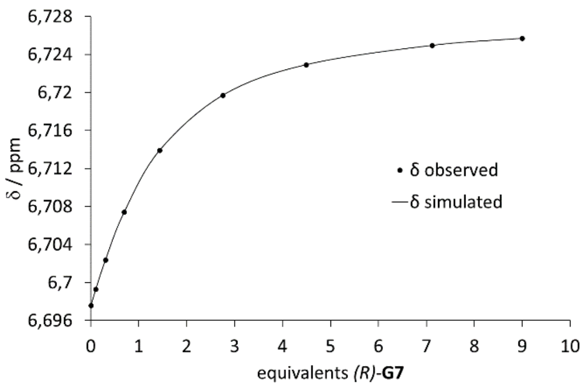

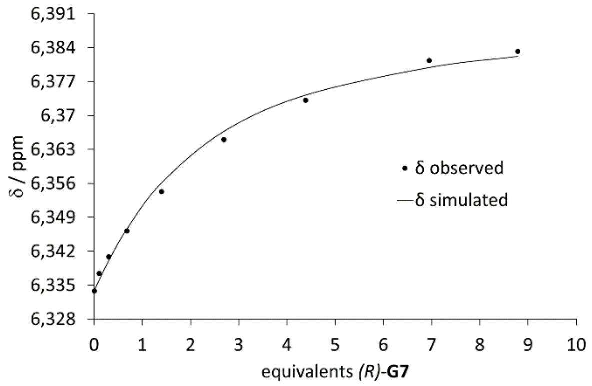

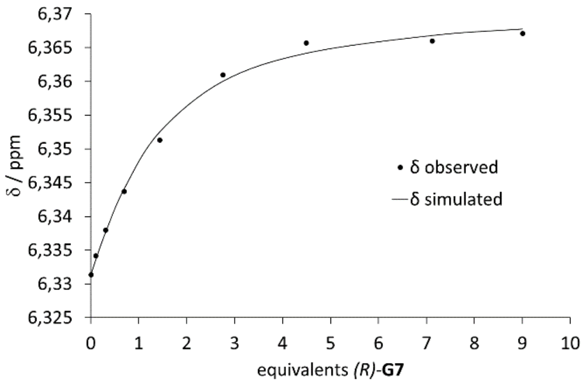

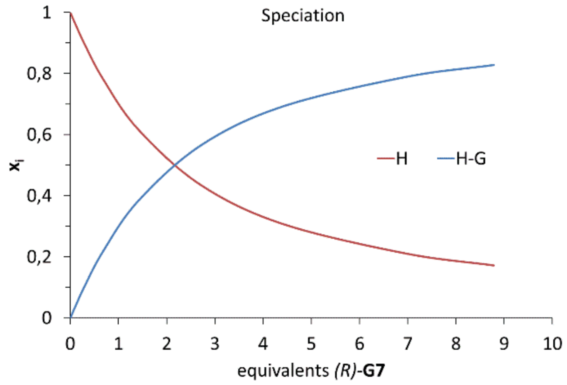

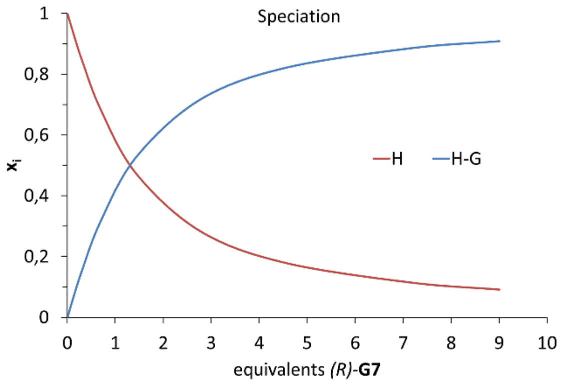

^{1}H NMR titration of ***R*-3** / (*R*)-**G7**

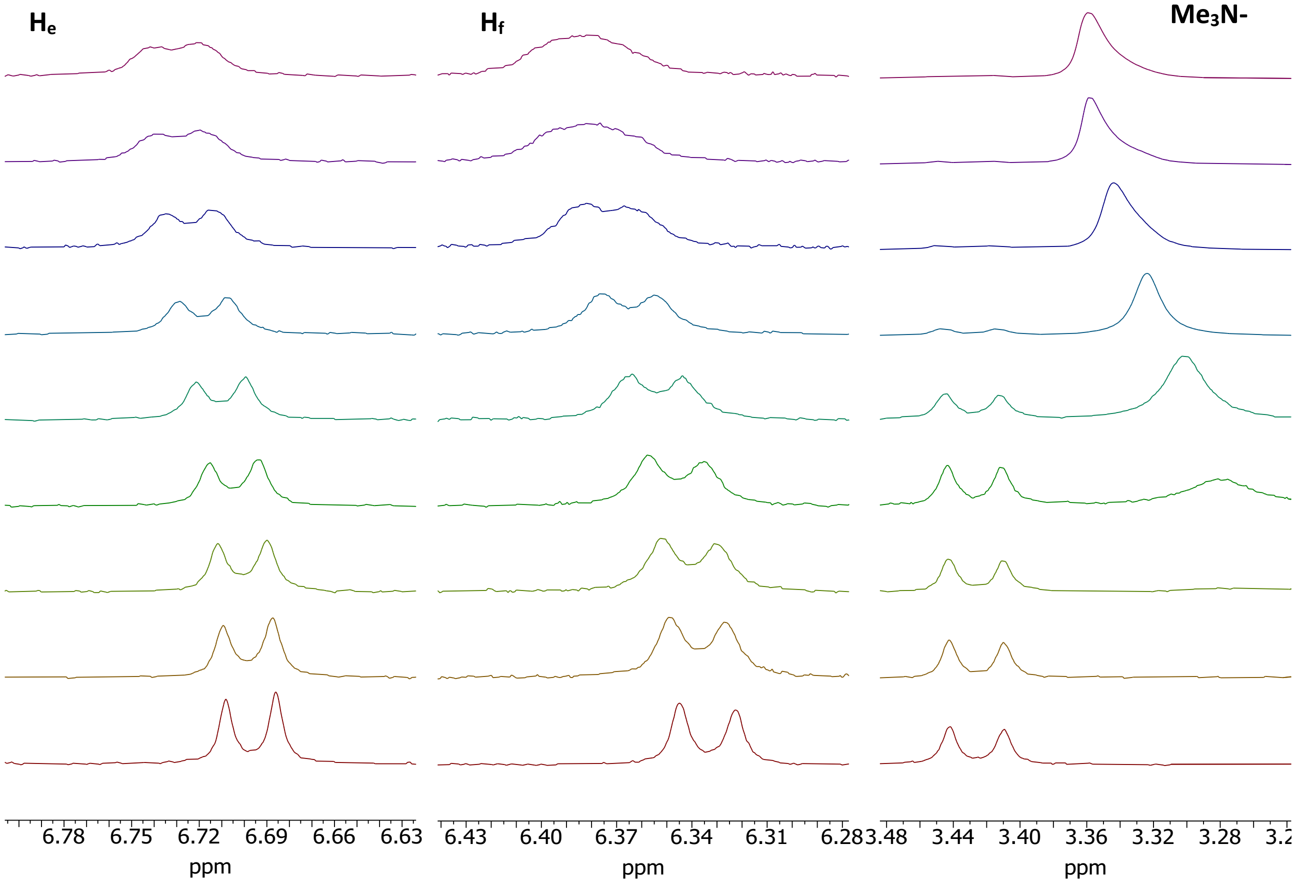

^{1}H NMR titration of ***S*-3** / (*R*)-**G7**

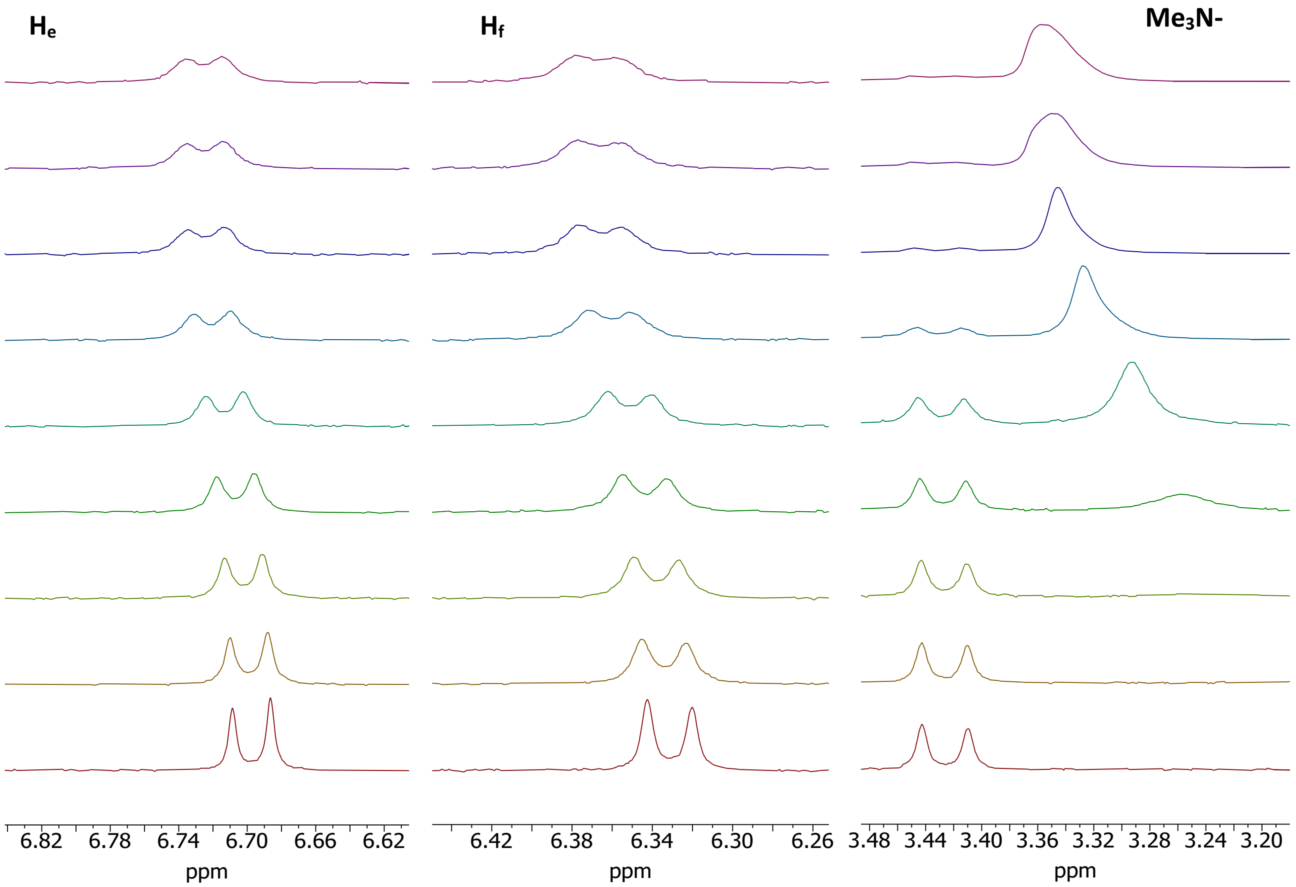

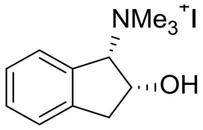

(1*S*,2*R*)-**G8**

***R*-3** / (1*S*,2*R*)-**G8**	K_a = 555±68 M^{-1}	***S*-3** / (1*S*,2*R*)-**G8**	K_a = 417±27 M^{-1}
	δ_{HG} = 6.708 / 6.358		δ_{HG} = 6.749 / 6.417

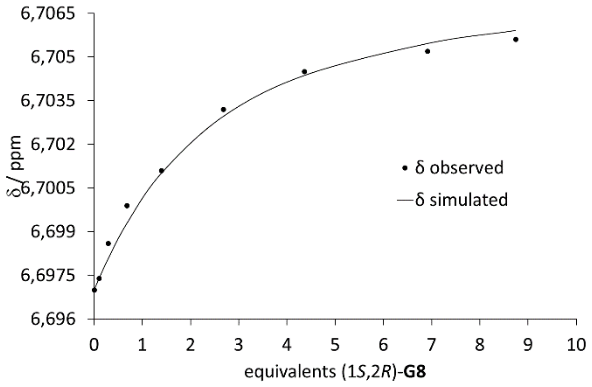

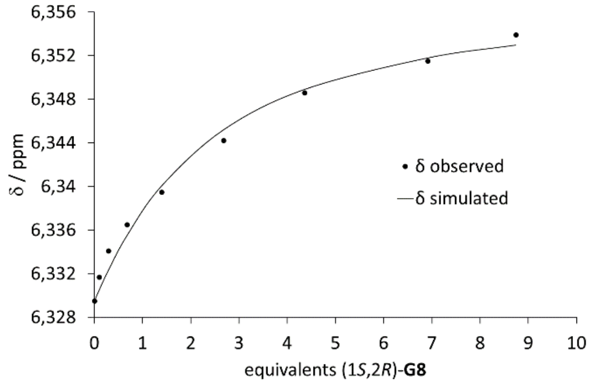

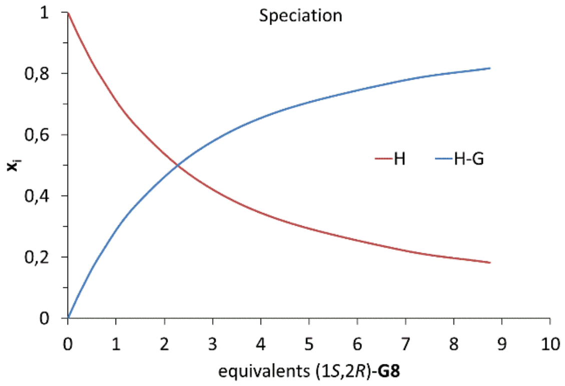

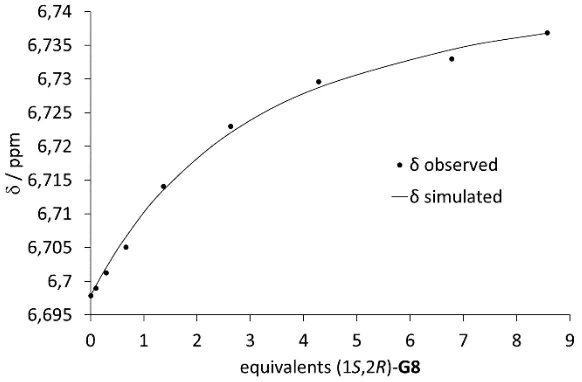

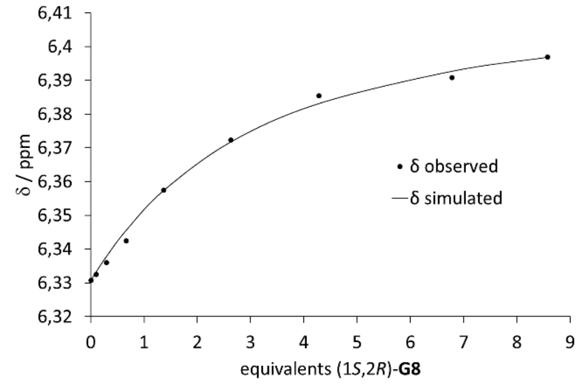

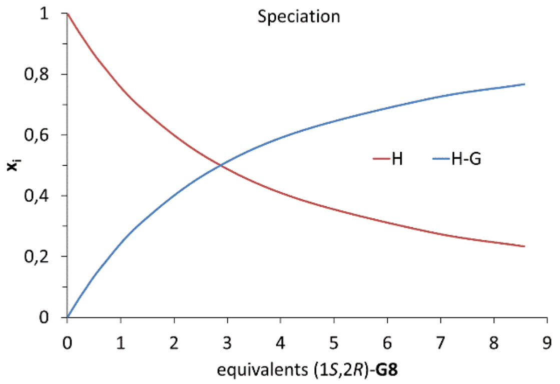

^{1}H NMR titration of ***R*-3** / (1*S*,2*R*)-**G8**

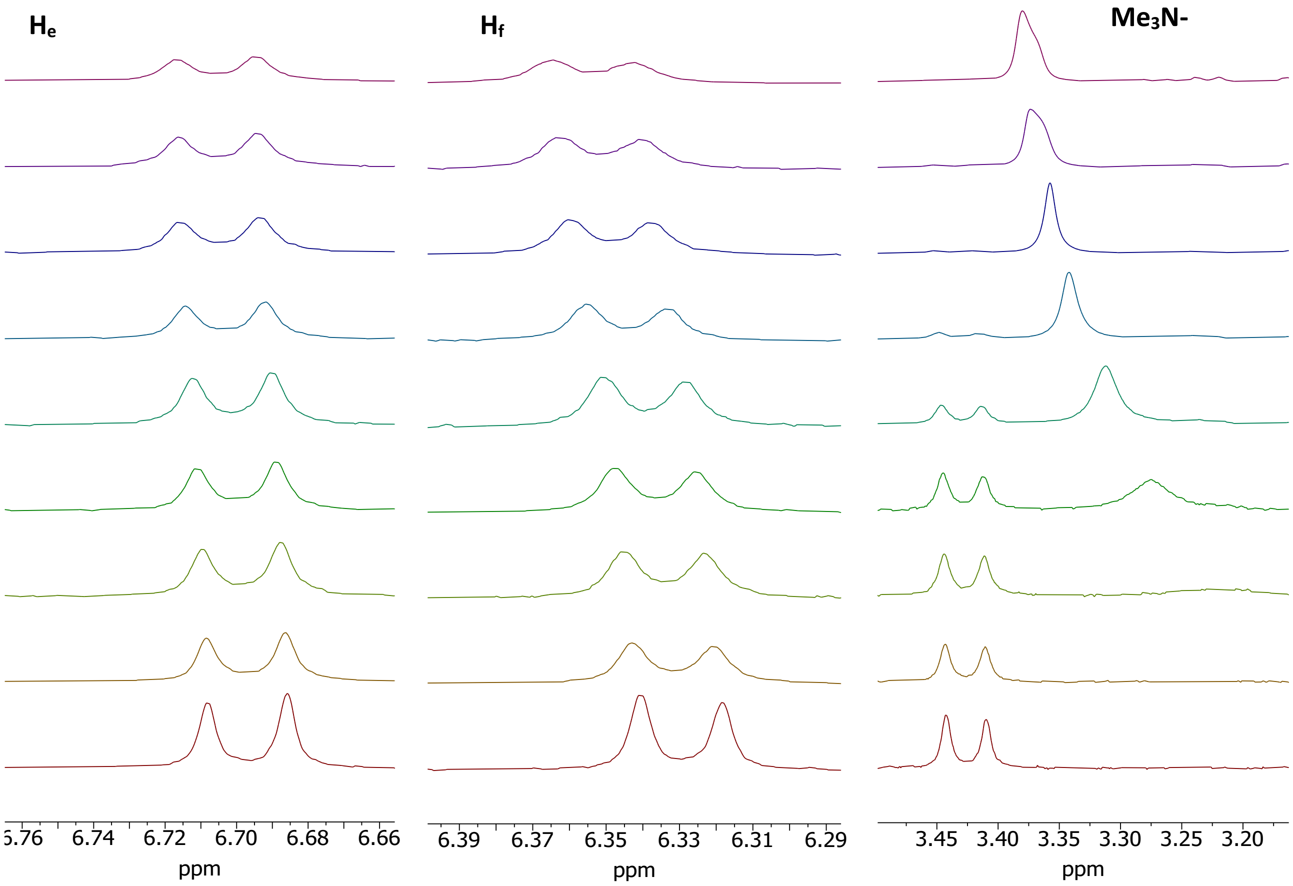

^{1}H NMR titration of ***S*-3** / (1*S*,2*R*)-**G8**

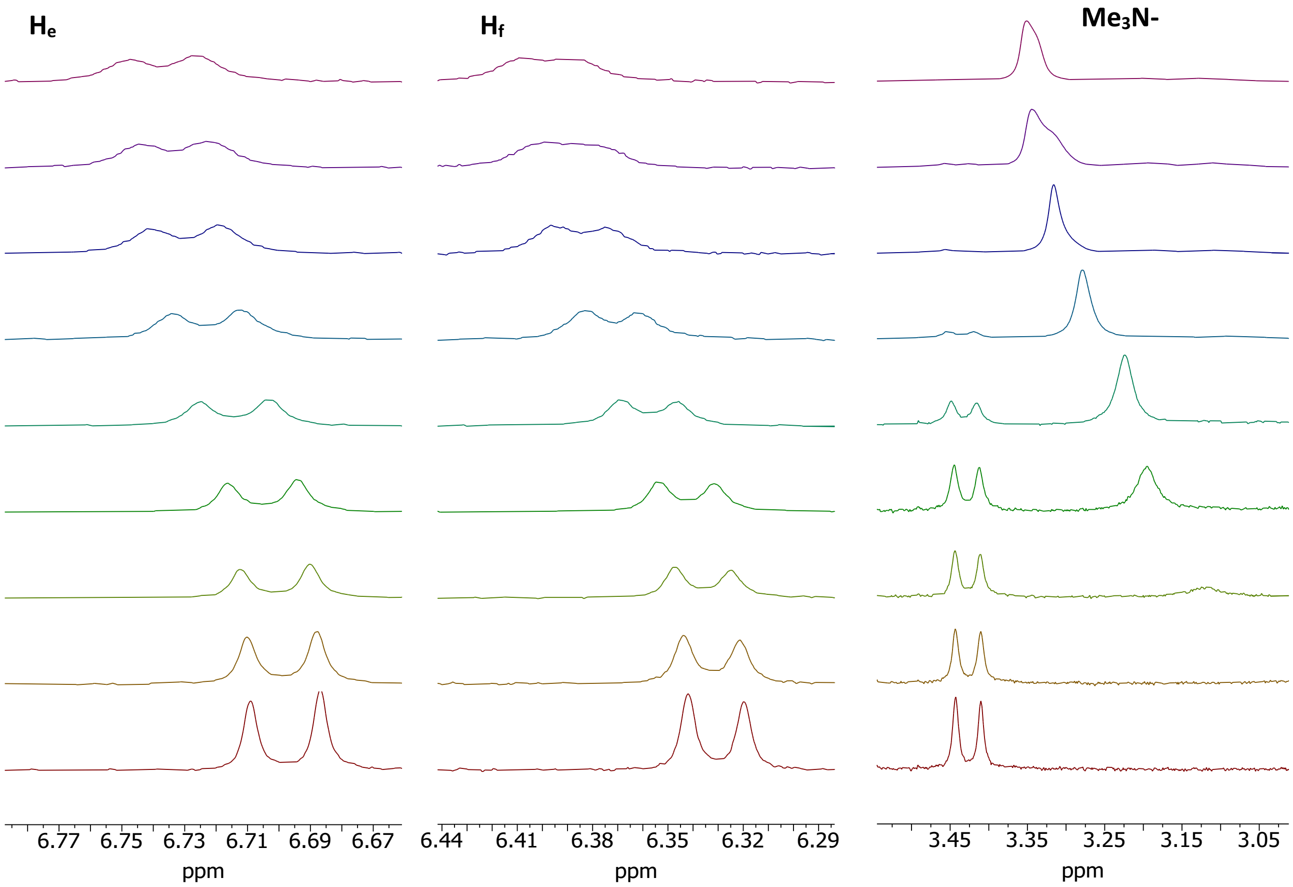

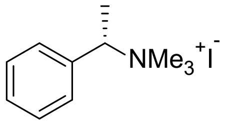

(*S*)-**G9**

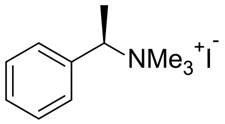

(*R*)-**G9**

***S*-3** / (*S*)-**G9**	K_a = 335±10 M^{-1}	***S*-3** / (*R*)-**G9**	K_a = 147±3 M^{-1}
	δ_{HG} = 6.715 / 6.366		δ_{HG} = 6.750 / 6.401

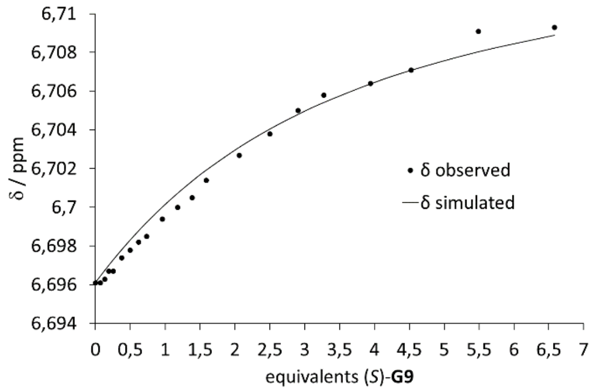

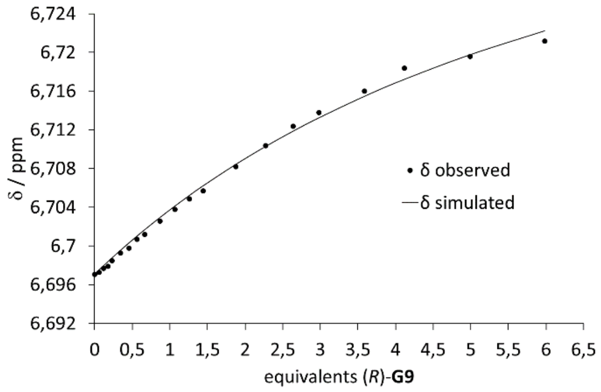

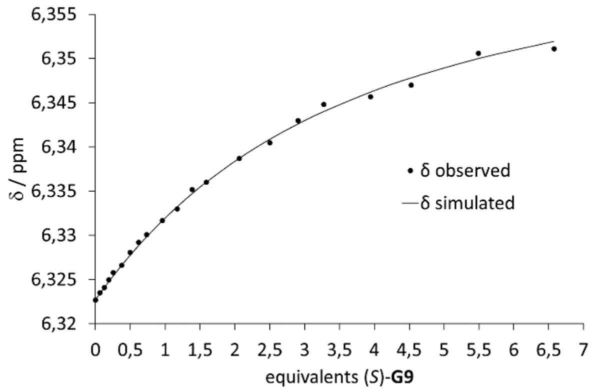

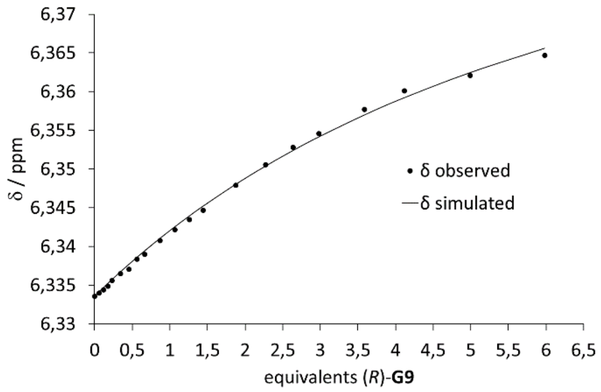

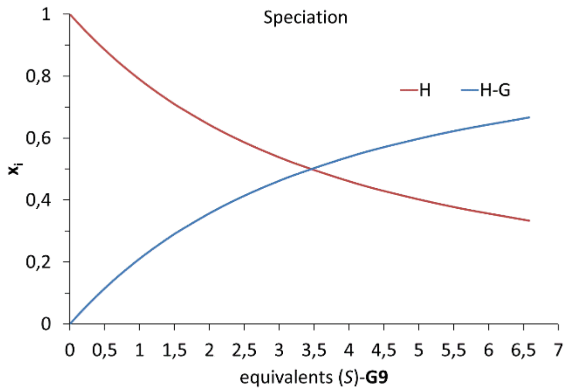

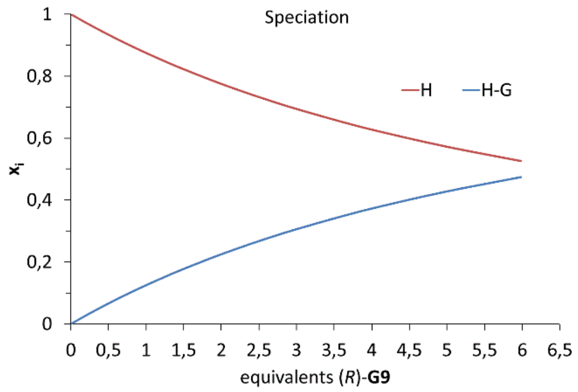

^{1}H NMR titration of ***S*-3** / (*S*)-**G9**

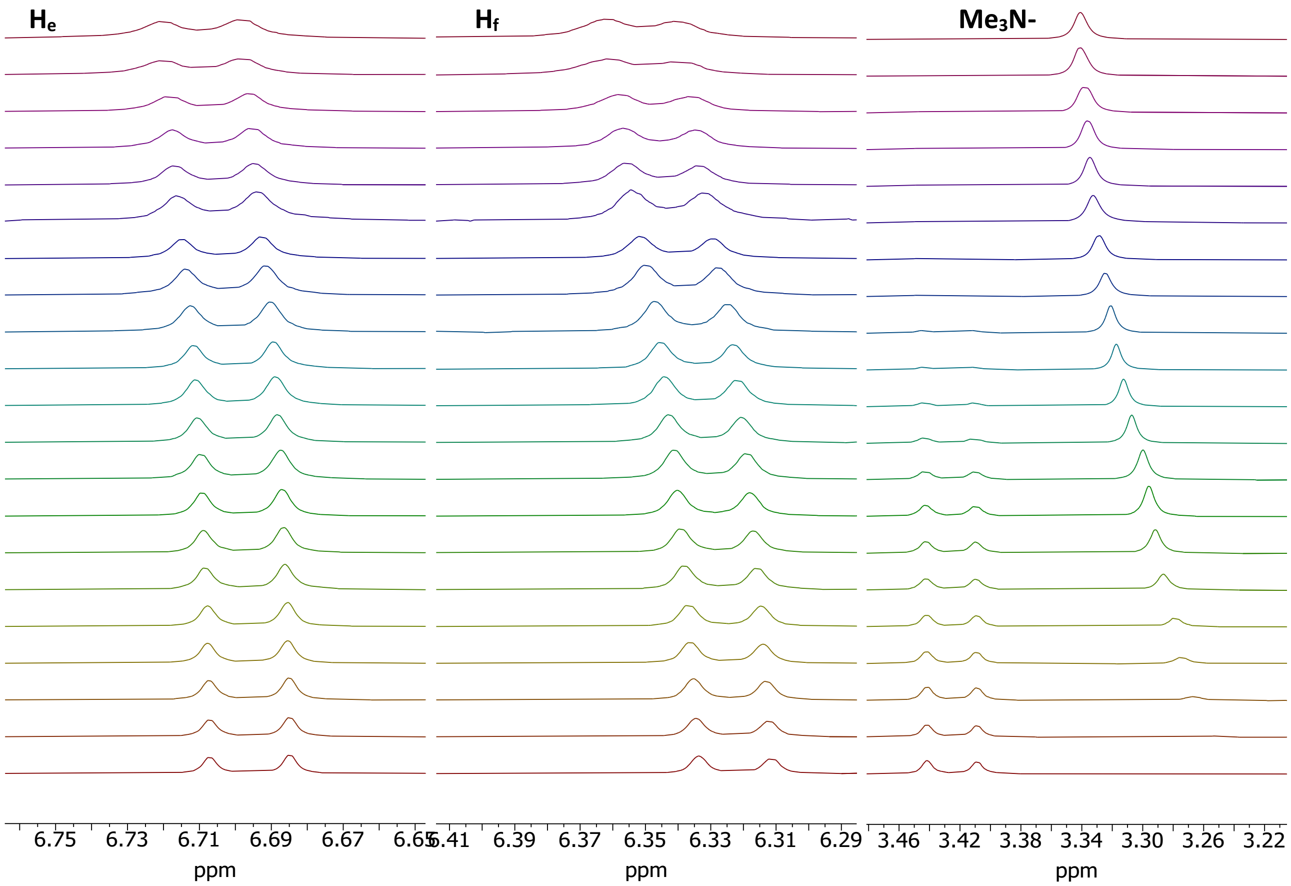

^{1}H NMR titration of ***S*-3** / (*R*)-**G9**

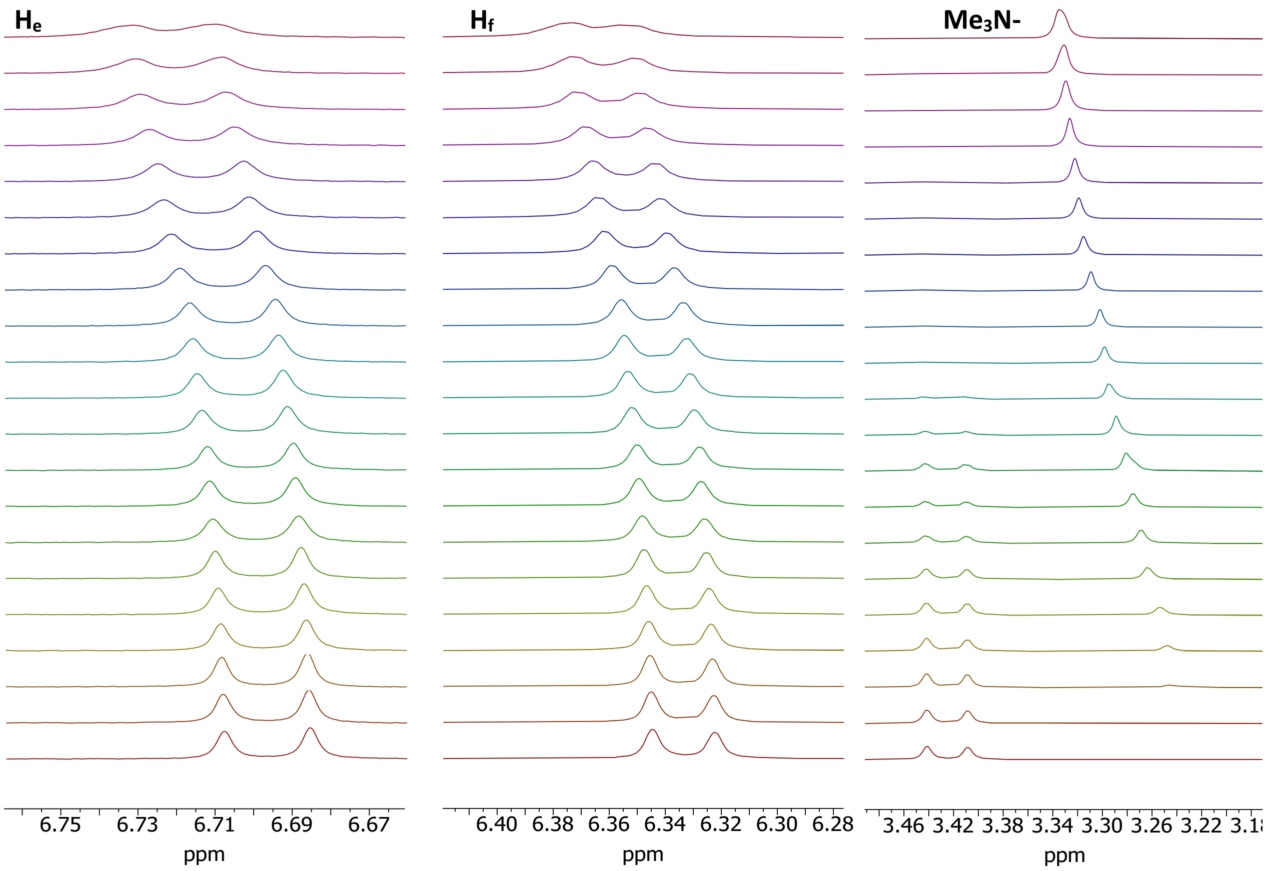

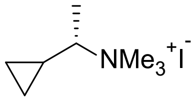

(*S*)-**G10**

***R*-3** / (*S*)-**G10** K_a = 187±11 M^{-1}

δ_{HG} = 6.750 / 6.440

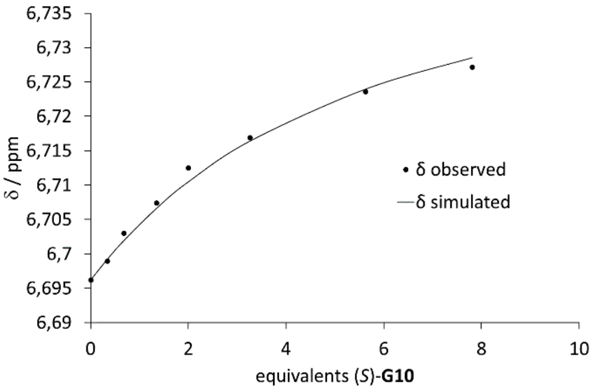

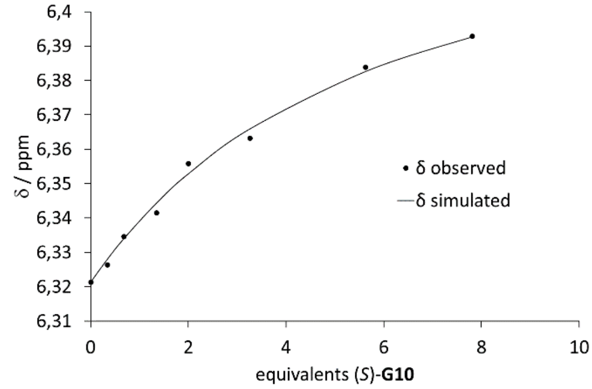

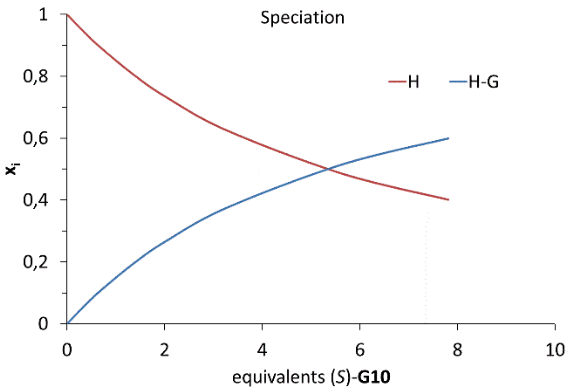

***S*-3** / (*S*)-**G10** K_a = 1416±92 M^{-1}

δ_{HG} = 6.702 / 6.402

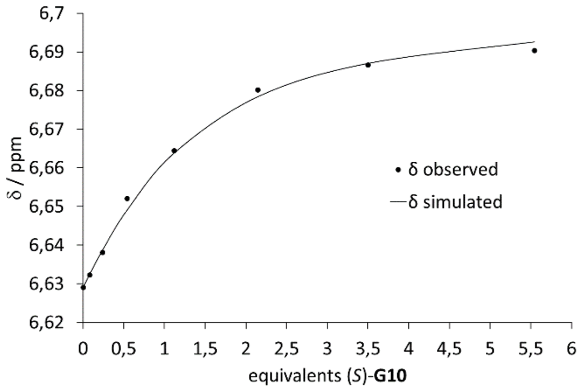

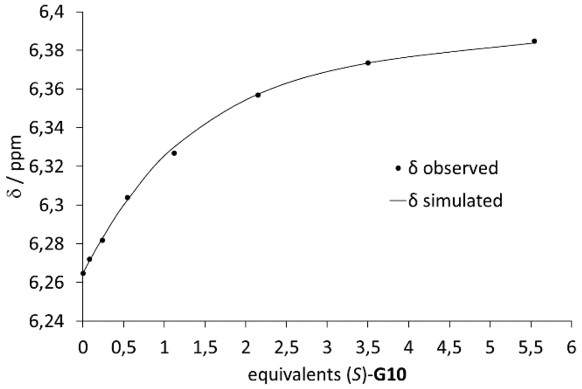

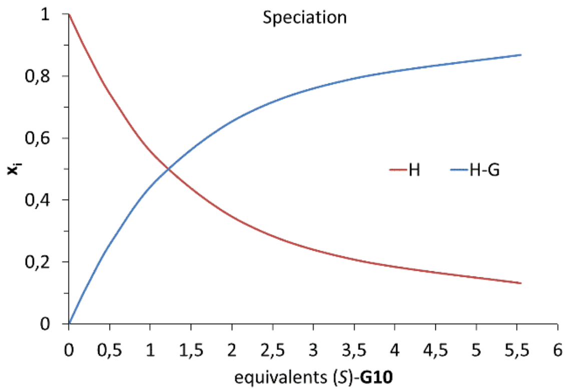

^{1}H NMR titration of ***R*-3** / (*S*)-**G10**

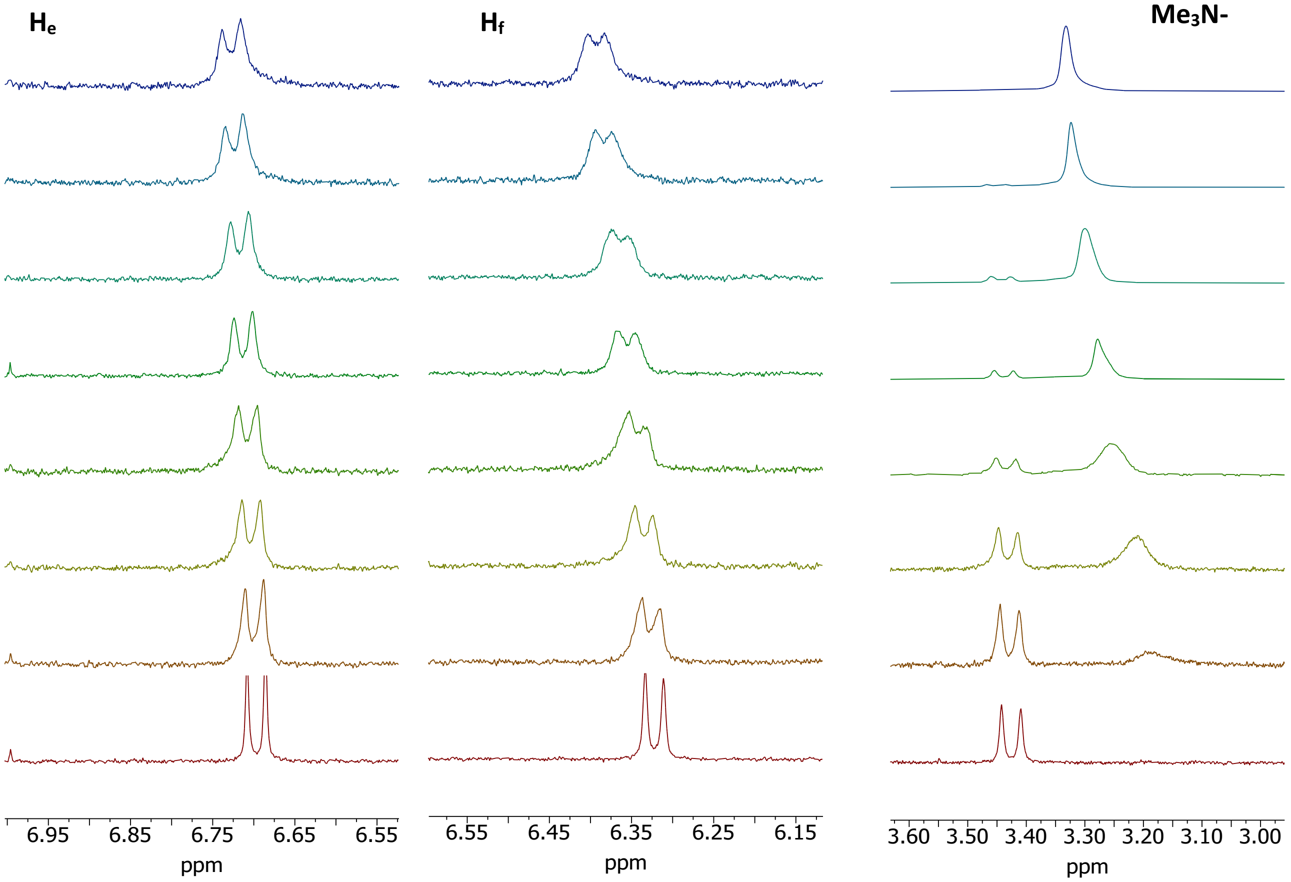

^{1}H NMR titration of ***S*-3** / (*S*)-**G10**

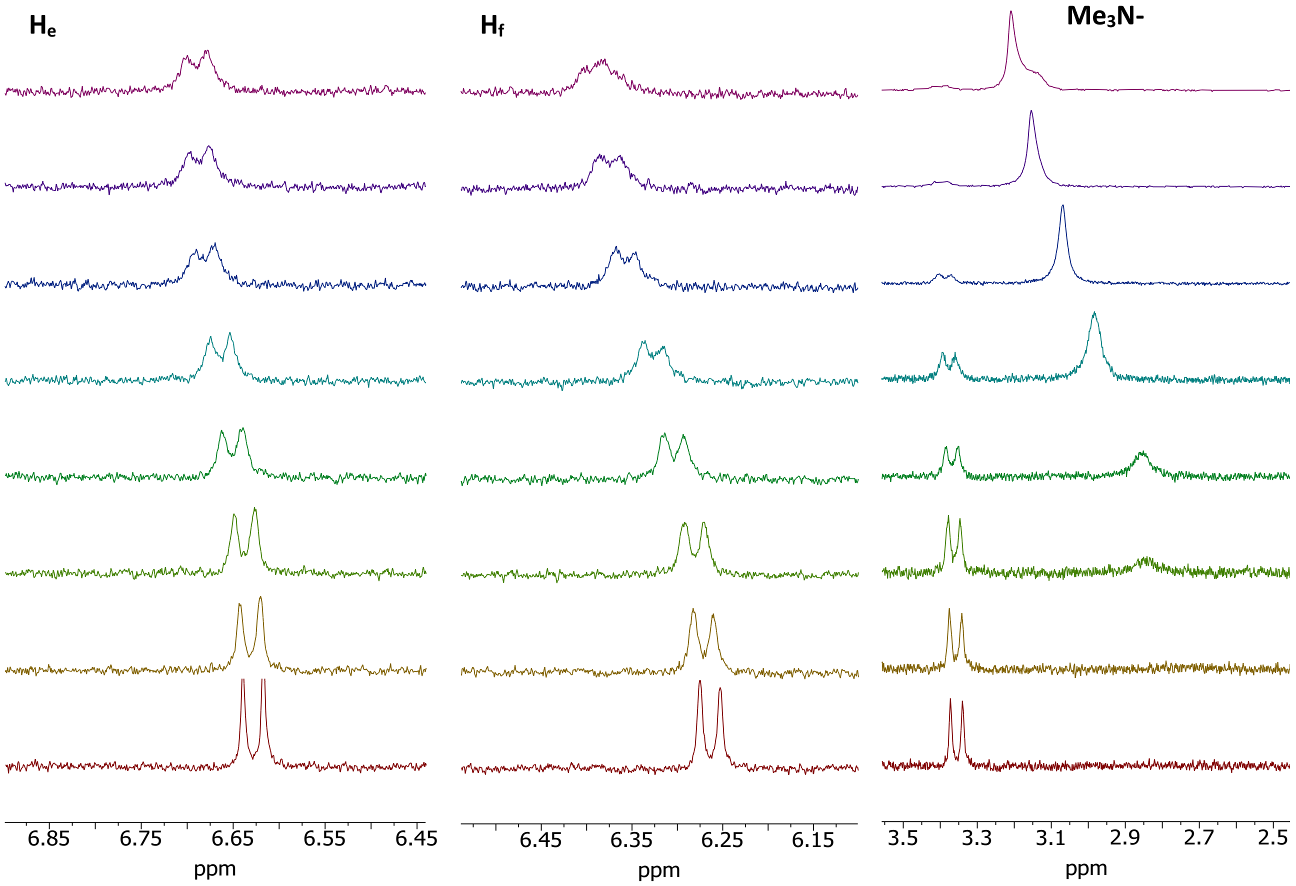

6 MD Simulations

Starting geometries for the MD simulations were obtained by minimization of the corresponding host-guest complexes, including the iodide counter ion. The minimizations were carried in the gas phase at the PM7 semi-empirical level, using the Gaussian 16 program.[16] The MD parameters for the cavitand ***S*-3** and the cationic portion of the guests ((*R*)-**G3**, (*S*)-**G10**) were generated within the Antechamber module of Amber 20 using the generalized Amber force field (GAFF2),[17] with partial charges calculated according to AM1-BCC scheme[18]. The standard AMBER parametrization for monovalent ions was used for the iodide counterion.[19] For the MD simulation of ***S*-3** in the absence of guests, the cavitand parameters were obtained from a PM7 optimized structure of the cavitand filled with 4 $CHCl_3$ molecules, which was the most stable arrangement observed in preliminary MD simulations. The MD trajectories for cavitand **1** have been previously reported, and were obtained analogously.[20]

Each system was immersed in a pre-equilibrated truncated cubic box of chloroform molecules with an internal offset distance of 12 Å, and the iodide counter ion was added, all using the Amber 20 LEaP module. All calculations were done using the GAFF force field. A two-stage geometry optimization approach was performed. First, a short minimization of the chloroform molecules positions, with positional restraints on solute by a harmonic potential with a force constant of 500 kcal mol^{-1} $Å^{-2}$ was done. The second stage was an unrestrained minimization of all the atoms in the simulation cell. Then, the systems were heated using six 50 ps steps, incrementing the temperature 50 K each step (0-300 K) under constant-volume, periodic-boundary conditions and the particle-mesh Ewald approach[21] to introduce long-range electrostatic effects. For these steps, a 10 Å cut-off was applied to Lennard-Jones and electrostatic interactions. Bonds involving hydrogen were constrained with the SHAKE algorithm. Harmonic restraints of 10 kcal mol^{-1} were applied to the solute, and the Langevin equilibration scheme was used to control and equalize the temperature. The time step was kept at 2 fs during the heating stages, allowing potential inhomogeneities to self-adjust. Each system was then equilibrated for 2 ns with a 2 fs time step at a constant pressure of 1 atm (NPT ensemble). Then MD simulations were performed under the NVT ensemble (constant volume) and periodic-boundary conditions. For each system, 500 ns of MD simulation were computed (1 μs for **1**).

The MD trajectories were obtained with the Cpptraj module of AMBER 20. For structure alignment (Figure S7), pdb files at 5 ns intervals were extracted (10 ns for **1**), removing solvent, bound guest, and iodide counter ion. The ensemble of 100 conformations of the cavitand was aligned to the first frame. Structure superposition was carried out with Pymol v. 2.5.0. From the same ensemble of pdb files, volumes of the cavitand's buried space were calculated at 20 ns intervals (40 ns for **1**) using the channel finder tool on the 3v Website (http://3vee.molmovdb.org/index.php)(Figure S8).[22] The calculations were carried out using a 30 Å outer probe radius, a 1.4 Å inner probe radius (water), and high grid resolution.

Video files were generated with Chimera v. 1.10.2 from *.crd trajectory coordinate files of reduced dimensionality obtained from Cpptraj. All videos show the cavitand in stick representation. Non-polar hydrogens of the cavitand and bulk solvent molecules are omitted for clarity. The following video files are provided:

File	Description
R4.mpg4	1 μs trajectory of cavitand **1** in chloroform. The $CHCl_3$ bound in the cavity is shown in CPK representation.
SC5B.mpg4	500 ns trajectory of cavitand ***S*-3** in chloroform. Selected $CHCl_3$ molecules that occupy the cavity at some point of the trajectory are shown in CPK representation.
SC5B_RG3.mpg4	500 ns trajectory of cavitand ***S*-3** in chloroform with bound (*R*)-**G3**. The guest is shown in CPK representation. The iodide counter ion is shown in pink
SC5B_SG10.mpg4	500 ns trajectory of cavitand ***S*-3** in chloroform with bound (*S*)-**G10**. The guest and $CHCl_3$ molecules that occupy the cavity at some point of the trajectory are shown in CPK representation. The iodide counter ion is shown in pink

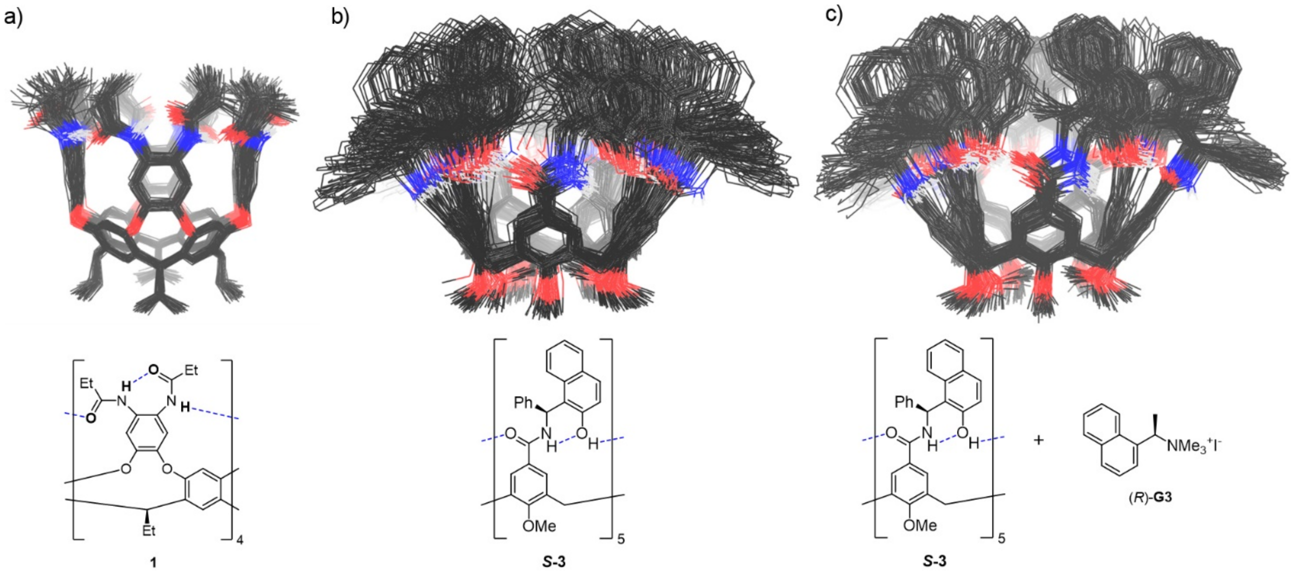

Figure S7. Overlaps of 100 aligned structures of MD simulations in $CHCl_3$ for a) resorcin[4]arene derived cavitand **1** alone, b) calix[5]arene derived cavitand ***S*-3** alone, and c) cavitand ***S*-3** with bound (*R*)-**G3**. Non-polar hydrogen atoms, solvent, and bound molecules (including $CHCl_3$) are omitted for clarity.

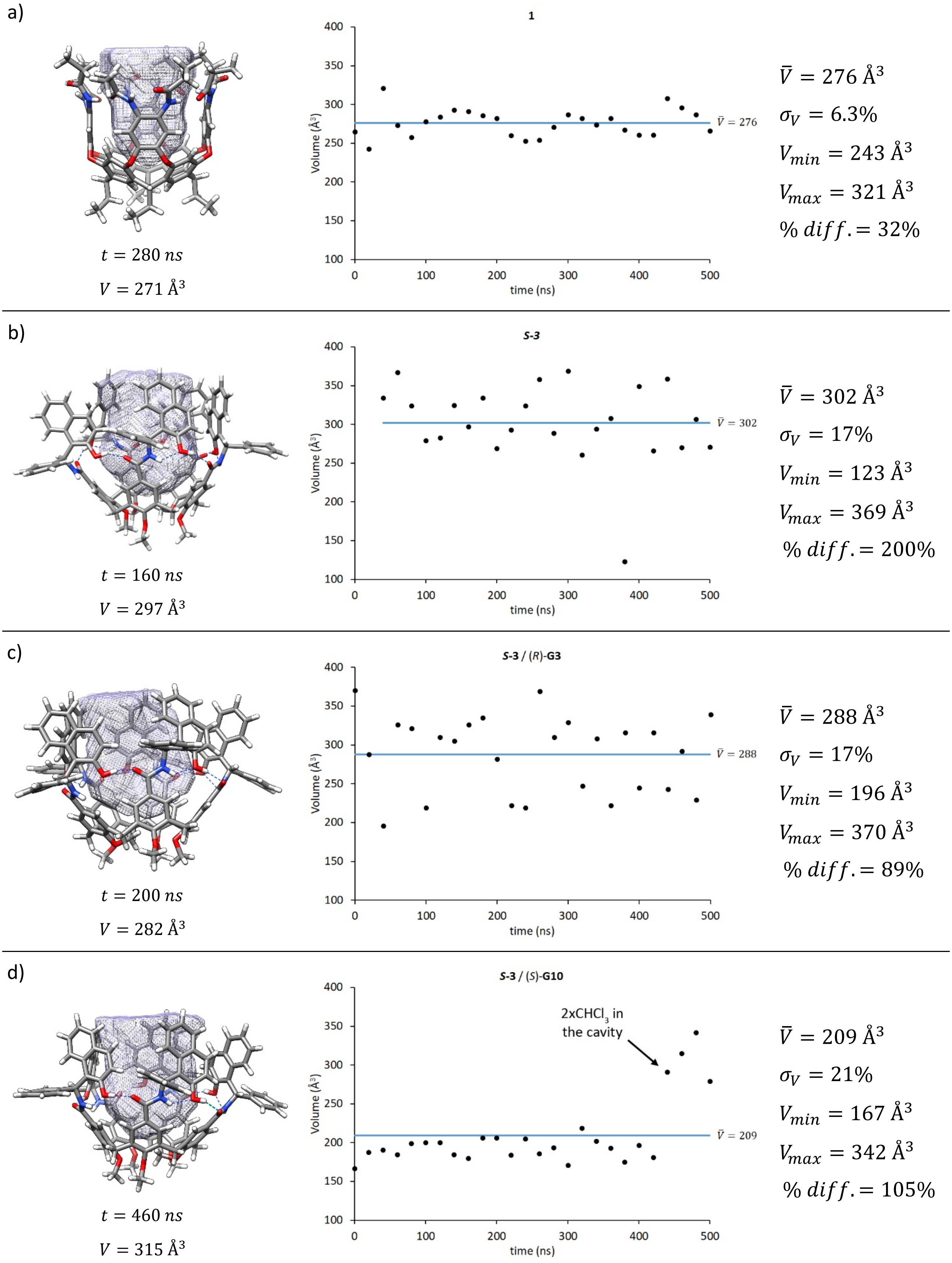

Figure S8. Variation of the buried solvent accessible volume at 20 ns intervals for a) cavitand **1**, b) cavitand ***S*-3**, c) cavitand ***S*-3** with bound (*R*)-**G3**, and d) cavitand ***S*-3** with bound (*S*)-**G10**. Representative snapshots displaying the buried volume as a mesh are shown on the left.

7 References

1. Dong, Y.; Li, R.; Lu, J.; Xu, X.; Wang, X.; Hu, Y., *J. Org. Chem.* **2005,** *70* (21), 8617-8620.
2. Betti, M., *Org. Synth.* **1929,** *9*, 60.
3. Lozano, D.; Álvarez-Yebra, R.; López-Coll, R.; Lledó, A., *Chem. Sci.* **2019,** *10* (44), 10351-10355.
4. Hill, R. K.; Chan, T. H.; Joule, J. A., *Tetrahedron* **1965,** *21* (1), 147-161.
5. Chen, H.; Zhang, Q.; Zheng, W.; Yang, H.; Zeng, Q., *Asian J. Org. Chem.* **2020,** *9* (5), 773-777.
6. Yamamoto, K.; Ikeda, K.; Yin, L. K., *J. Organomet. Chem.* **1989,** *370* (1), 319-332.
7. Alberico, E.; Braun, W.; Calmuschi-Cula, B.; Englert, U.; Salzer, A.; Totev, D., *Eur. J. Inorg. Chem.* **2007,** *2007* (31), 4923-4945.
8. Ollis, W. D.; Rey, M.; Sutherland, I. O., *J. Chem. Soc., Perkin Trans. 1* **1983,** (0), 1009-1027.
9. Weidmann, R.; Guette, J. P., *C. R. Acad. Sci., Paris, Ser. C* **1969,** *268* (25), 2225.
10. Chai, H.; Chen, Z.; Wang, S.-H.; Quan, M.; Yang, L.-P.; Ke, H.; Jiang, W., *CCS Chem* **2020,** *2* (6), 440-452.
11. Rodríguez-Escrich, S.; Solà, L.; Jimeno, C.; Rodríguez-Escrich, C.; Pericàs, M. A., *Adv. Synth. Catal.* **2008,** *350* (14-15), 2250-2260.
12. Zuo, W.; Huang, Z.; Zhao, Y.; Xu, W.; Liu, Z.; Yang, X.-J.; Jia, C.; Wu, B., *Chem. Commun.* **2018,** *54* (53), 7378-7381.
13. Zhang, G.-W.; Li, P.-F.; Meng, Z.; Wang, H.-X.; Han, Y.; Chen, C.-F., *Angew. Chem. Int. Ed.* **2016,** *55* (17), 5304-5308.
14. Brynn Hibbert, D.; Thordarson, P., *Chem. Commun.* **2016,** *52* (87), 12792-12805.
15. http://app.supramolecular.org/bindfit/
16. Frisch, M. J.; Trucks, G. W.; Schlegel, H. B.; Scuseria, G. E.; Robb, M. A.; Cheeseman, J. R.; Scalmani, G.; Barone, V.; Petersson, G. A.; Nakatsuji, H.; Li, X.; Caricato, M.; Marenich, A. V.; Bloino, J.; Janesko, B. G.; Gomperts, R.; Mennucci, B.; Hratchian, H. P.; Ortiz, J. V.; Izmaylov, A. F.; Sonnenberg, J. L.; Williams; Ding, F.; Lipparini, F.; Egidi, F.; Goings, J.; Peng, B.; Petrone, A.; Henderson, T.; Ranasinghe, D.; Zakrzewski, V. G.; Gao, J.; Rega, N.; Zheng, G.; Liang, W.; Hada, M.; Ehara, M.; Toyota, K.; Fukuda, R.; Hasegawa, J.; Ishida, M.; Nakajima, T.; Honda, Y.; Kitao, O.; Nakai, H.; Vreven, T.; Throssell, K.; Montgomery Jr., J. A.; Peralta, J. E.; Ogliaro, F.; Bearpark, M. J.; Heyd, J. J.; Brothers, E. N.; Kudin, K. N.; Staroverov, V. N.; Keith, T. A.; Kobayashi, R.; Normand, J.; Raghavachari, K.; Rendell, A. P.; Burant, J. C.; Iyengar, S. S.; Tomasi, J.; Cossi, M.; Millam, J. M.; Klene, M.; Adamo, C.; Cammi, R.; Ochterski, J. W.; Martin, R. L.; Morokuma, K.; Farkas, O.; Foresman, J. B.; Fox, D. J. *Gaussian 16*, Wallingford, CT, 2016.
17. Wang, J.; Wolf, R. M.; Caldwell, J. W.; Kollman, P. A.; Case, D. A., *J. Comput. Chem.* **2004,** *25* (9), 1157-1174.
18. Jakalian, A.; Bush, B. L.; Jack, D. B.; Bayly, C. I., *J. Comput. Chem.* **2000,** *21* (2), 132-146.
19. Li, P.; Song, L. F.; Merz, K. M., Jr., *J. Chem. Theory Comput.* **2015,** *11* (4), 1645-1657.
20. López-Coll, R.; Álvarez-Yebra, R.; Feixas, F.; Lledó, A., *Chem. Eur. J.* **2021,** *27* (39), 10099-10106.
21. Darden, T.; York, D.; Pedersen, L., *J. Chem. Phys.* **1993,** *98* (12), 10089-10092.
22. Voss, N. R.; Gerstein, M., *Nucleic Acids Res.* **2010,** *38* (suppl_2), W555-W562.

8 NMR Spectra of new compounds and known compounds without previously reported data.

^{1}H NMR (400 MHz, $CDCl_3$, 298K)

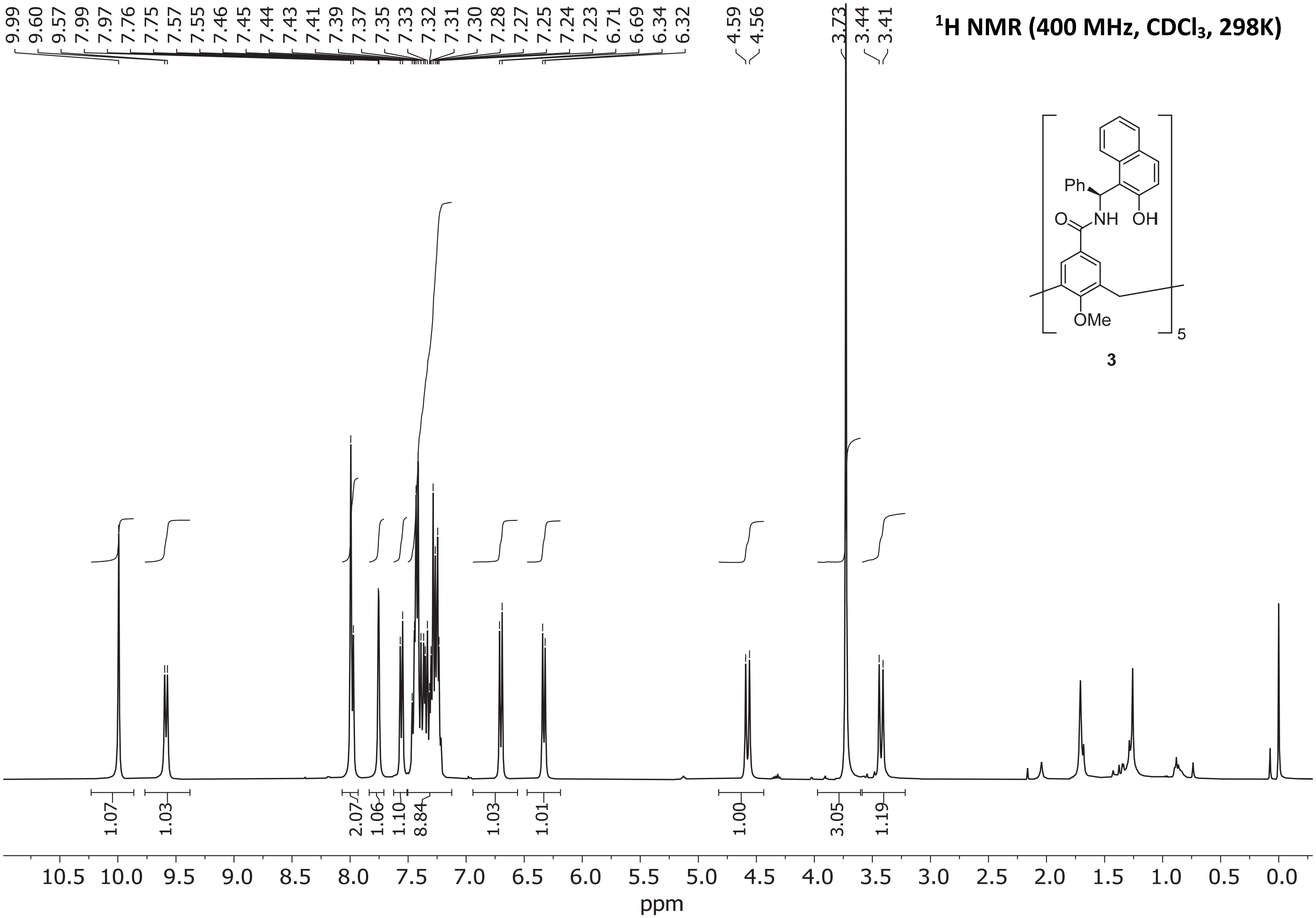

3

^{13}C NMR (101 MHz, $CDCl_3$, 298K)

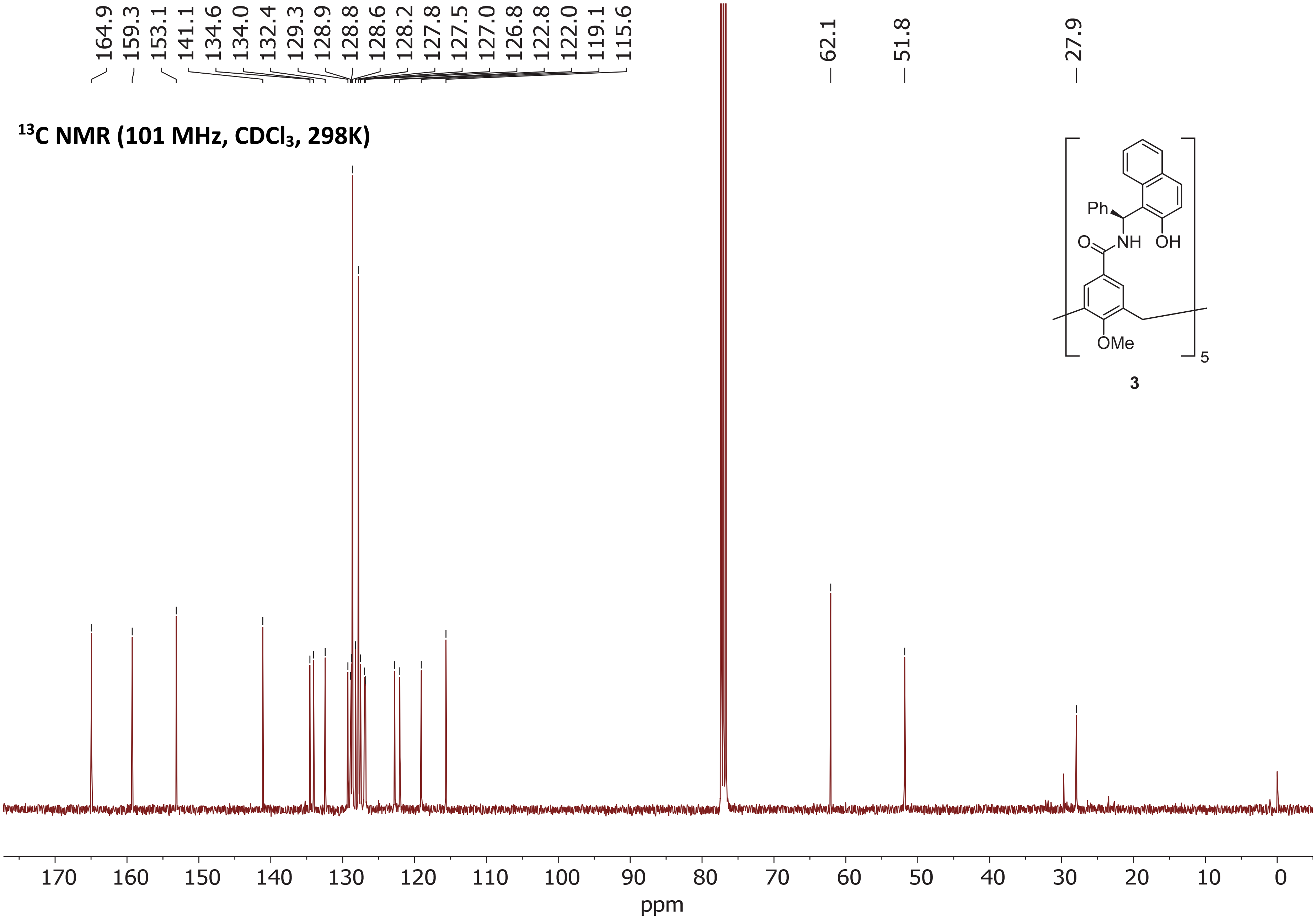

3

DETPQ (101 MHz, CDCl$_3$, 298K)

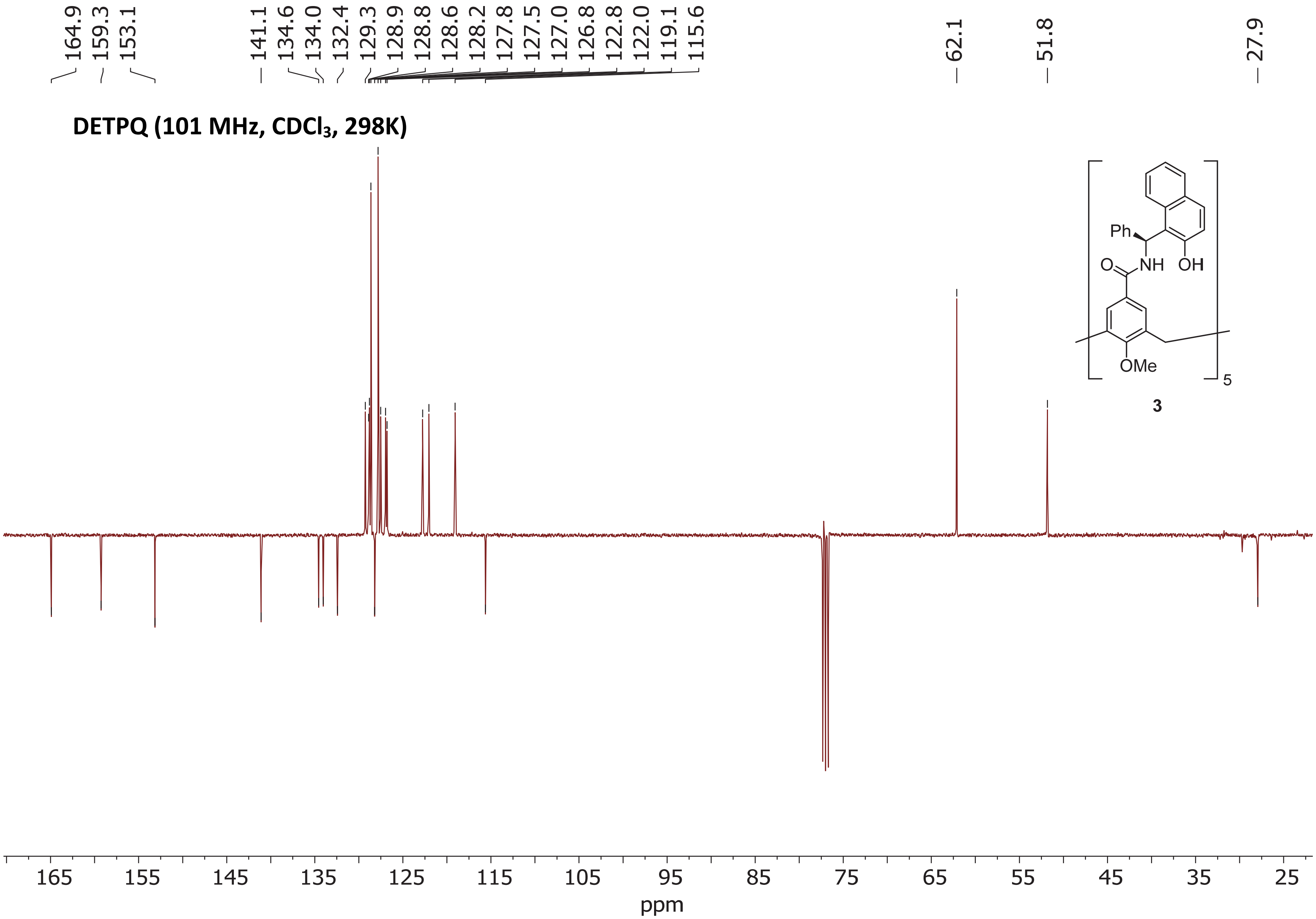

^{1}H-^{13}C HSQC (400 MHz, $CDCl_3$, 298K)

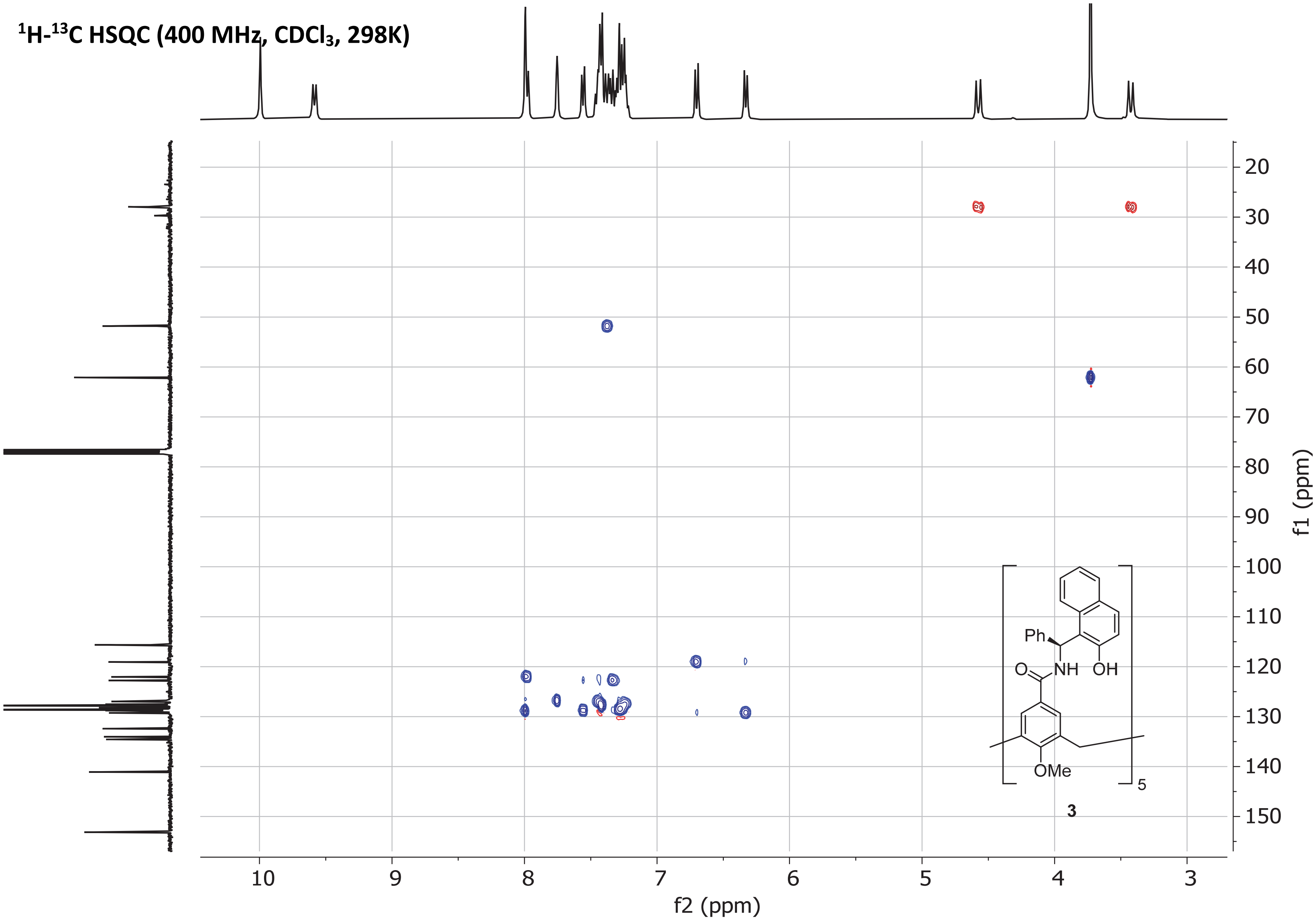

3

COSY (400 MHz, $CDCl_3$, 298K)

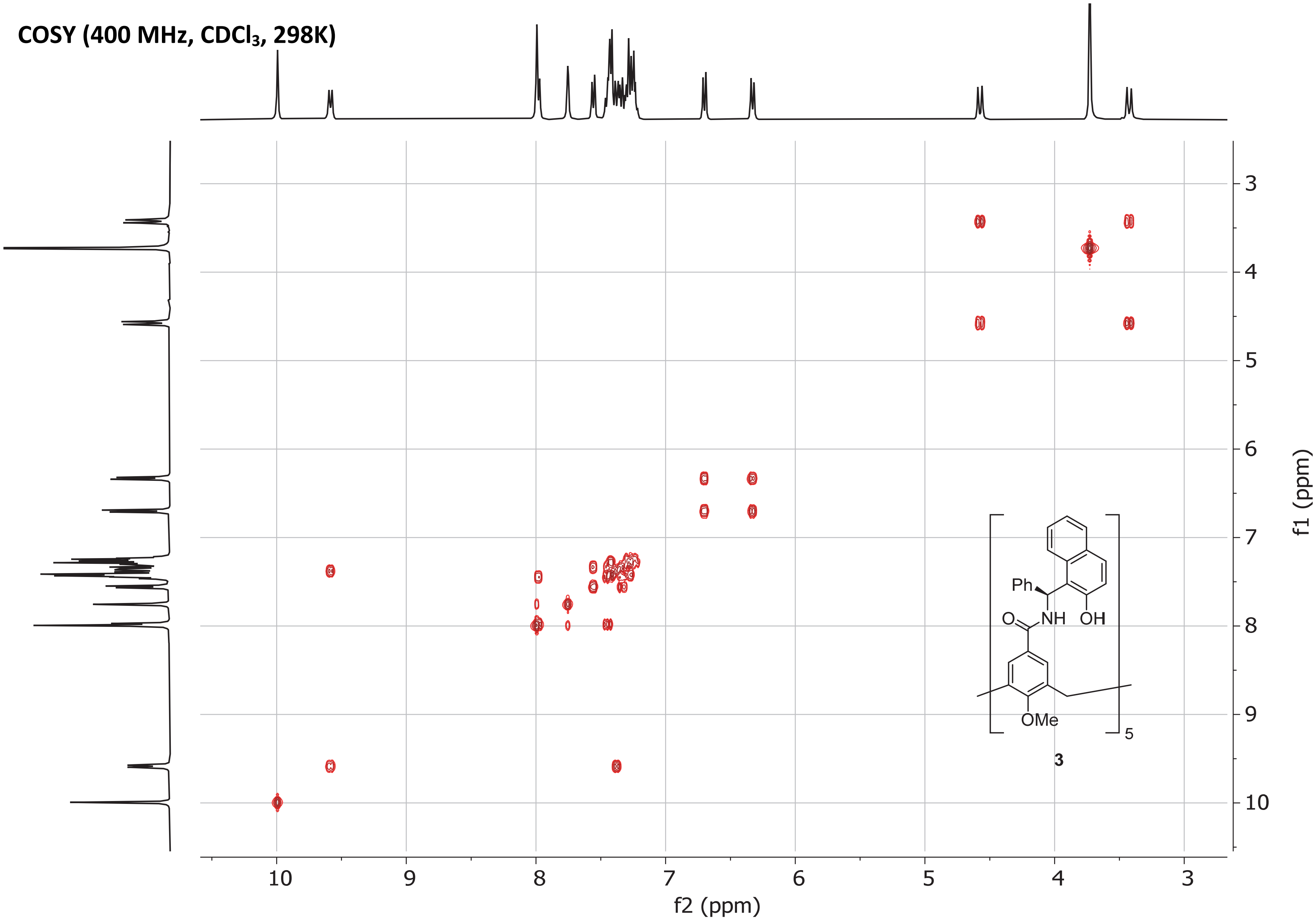

^{1}H-^{15}N HSQC (400 MHz, $CDCl_3$, 298K)

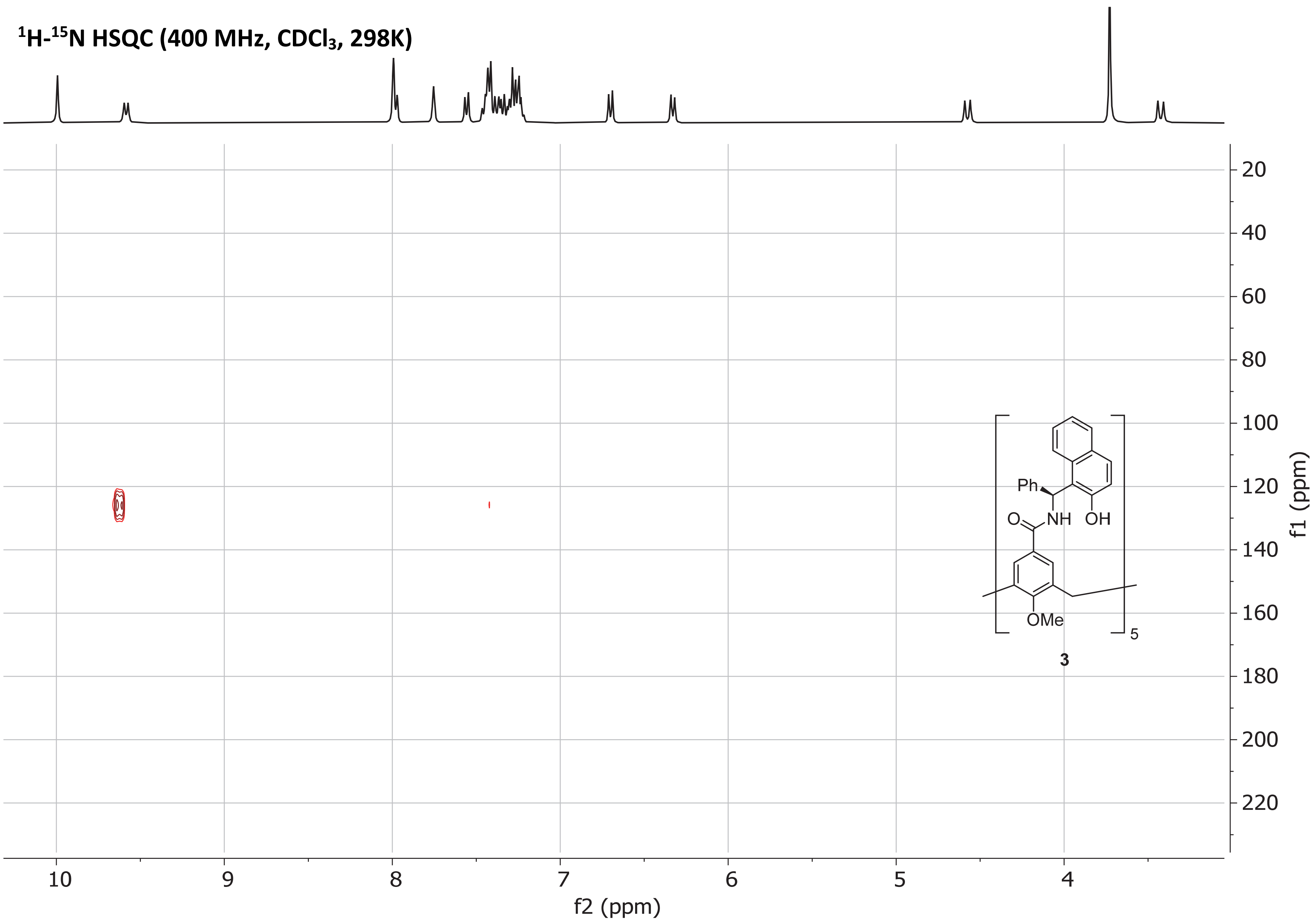

3

2D NOESY τ = 500 ms (400 MHz, $CDCl_3$, 298K)

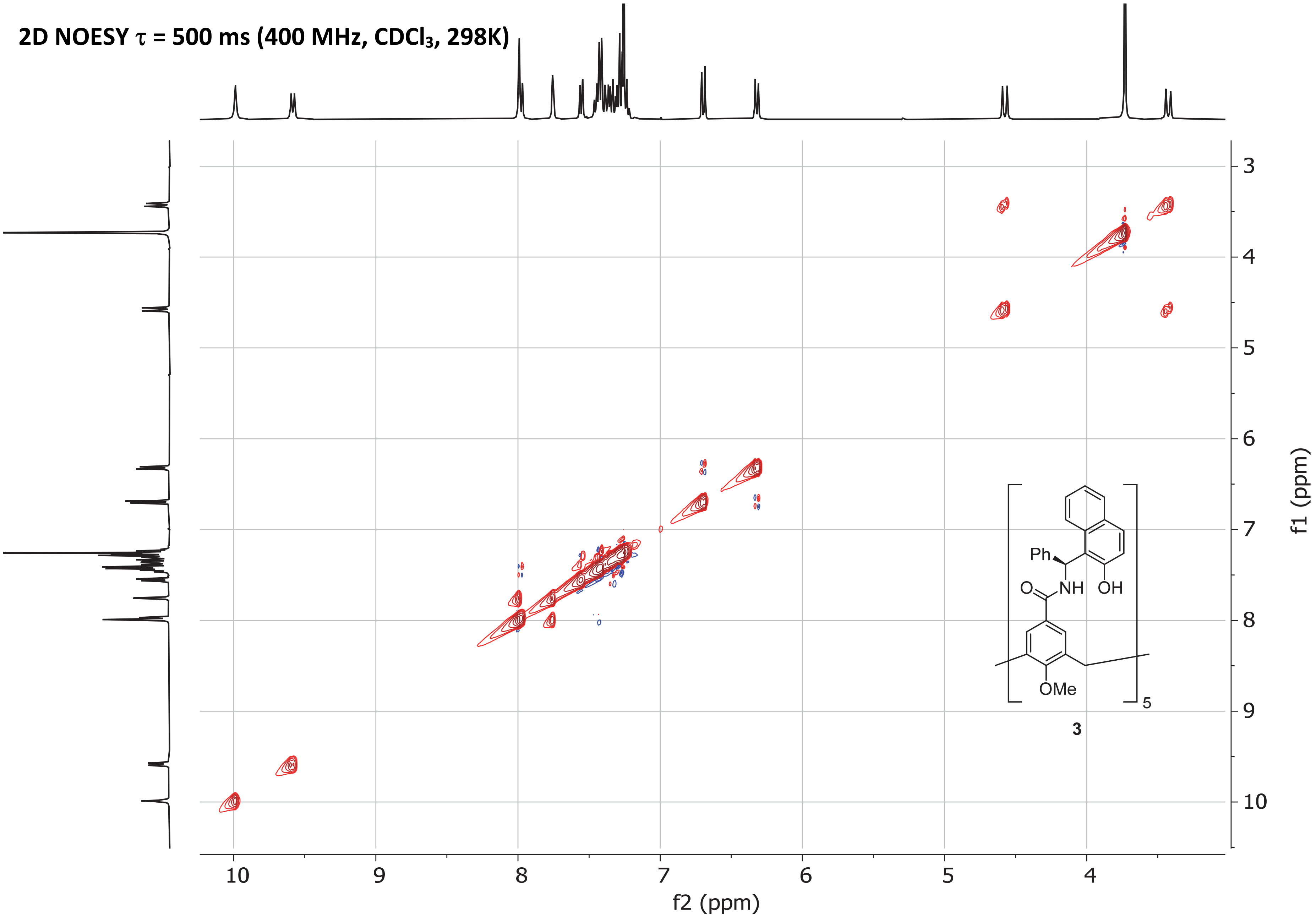

2D ROESY (400 MHz, $CDCl_3$, 298K)

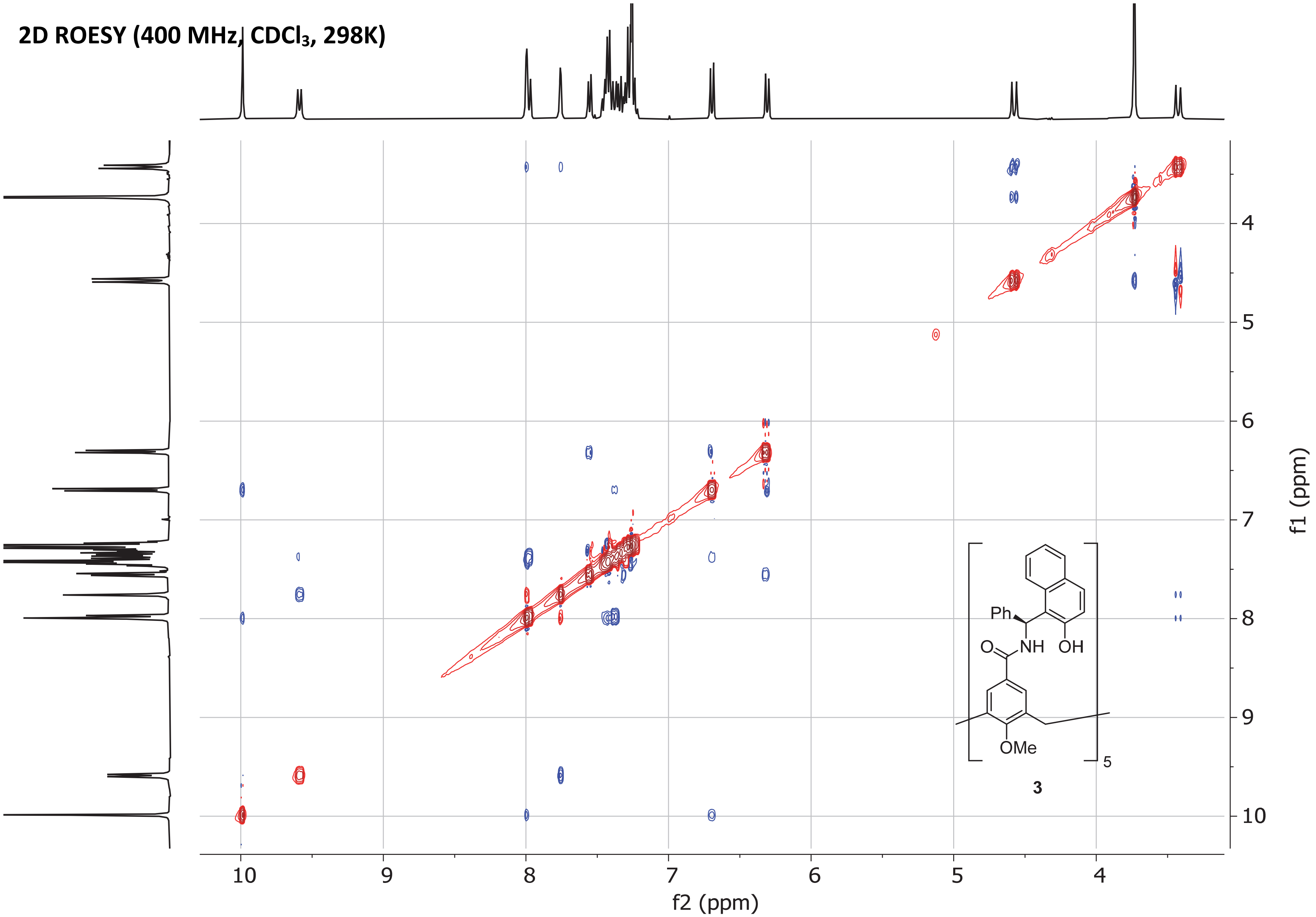

^{1}H NMR (400 MHz, $CDCl_3$, 298K)

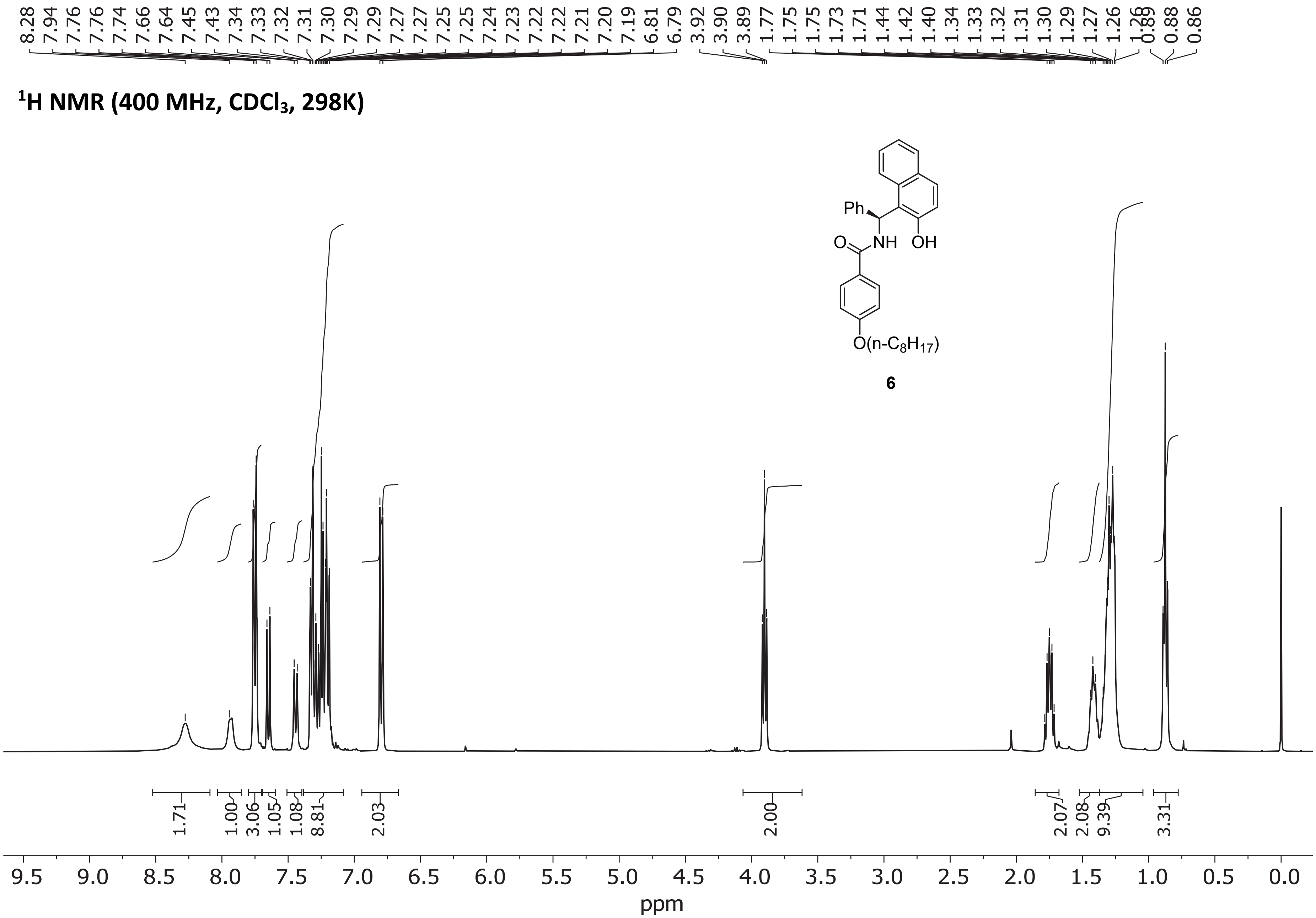

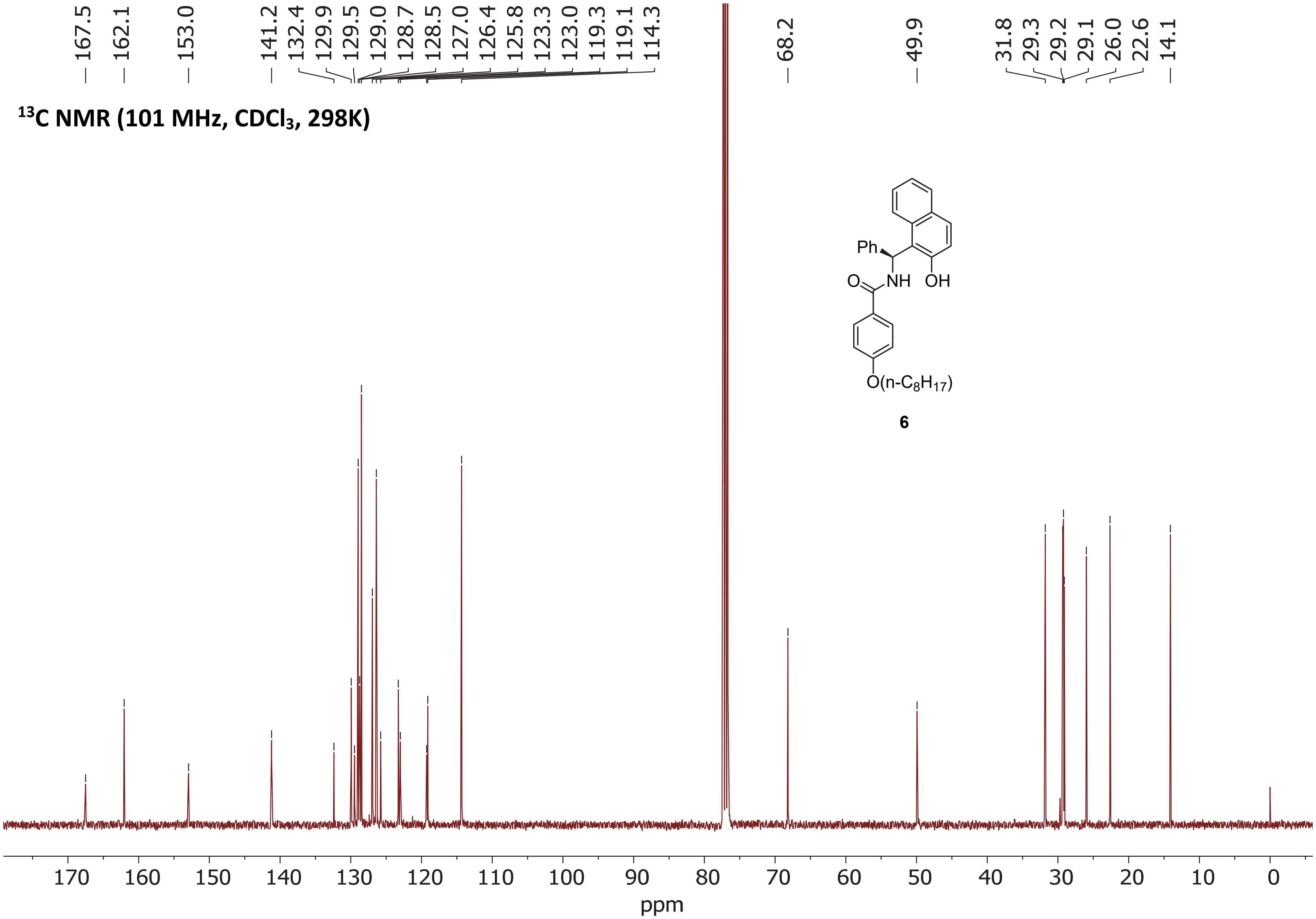
167.5
162.1
153.0
141.2
132.4
129.9
129.5
129.0
128.7
128.5
127.0
126.4
125.8
123.3
123.0
119.3
119.1
114.3
68.2
49.9
31.8
29.3
29.2
29.1
26.0
22.6
14.1
^{13}C NMR (101 MHz, $CDCl_3$, 298K)
Ph
O
NH
OH
O(n-C_8H_{17})
6
170
160
150
140
130
120
110
100
90
80
70
60
50
40
30
20
10
0
ppm

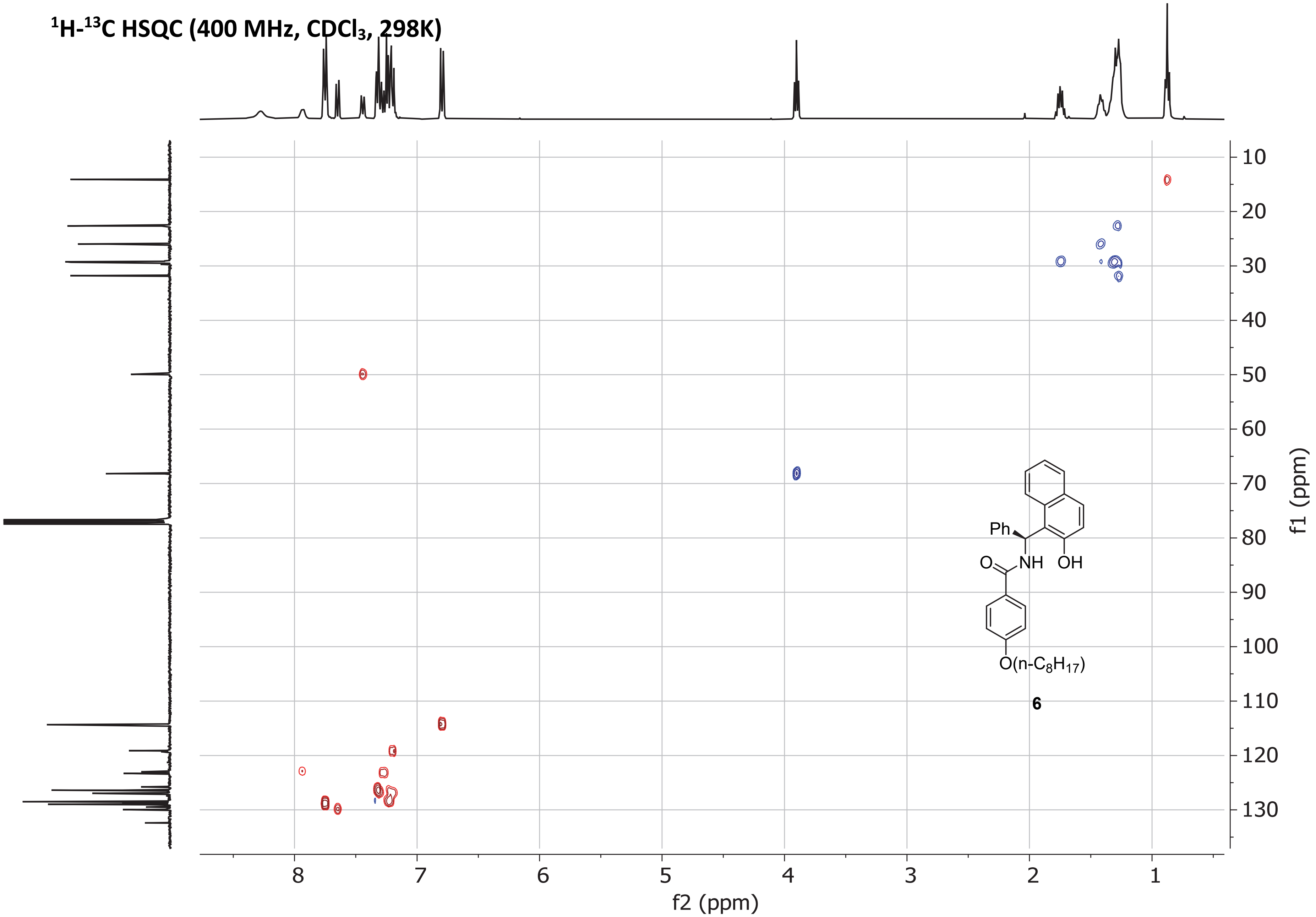

^{1}H-^{13}C HSQC (400 MHz, CDCl$_3$, 298K)
Ph
O
NH
OH
O(n-C$_8$H$_{17}$)
6
f2 (ppm)
f1 (ppm)

^{1}H-^{15}N HSQC (400 MHz, $CDCl_3$, 298K)

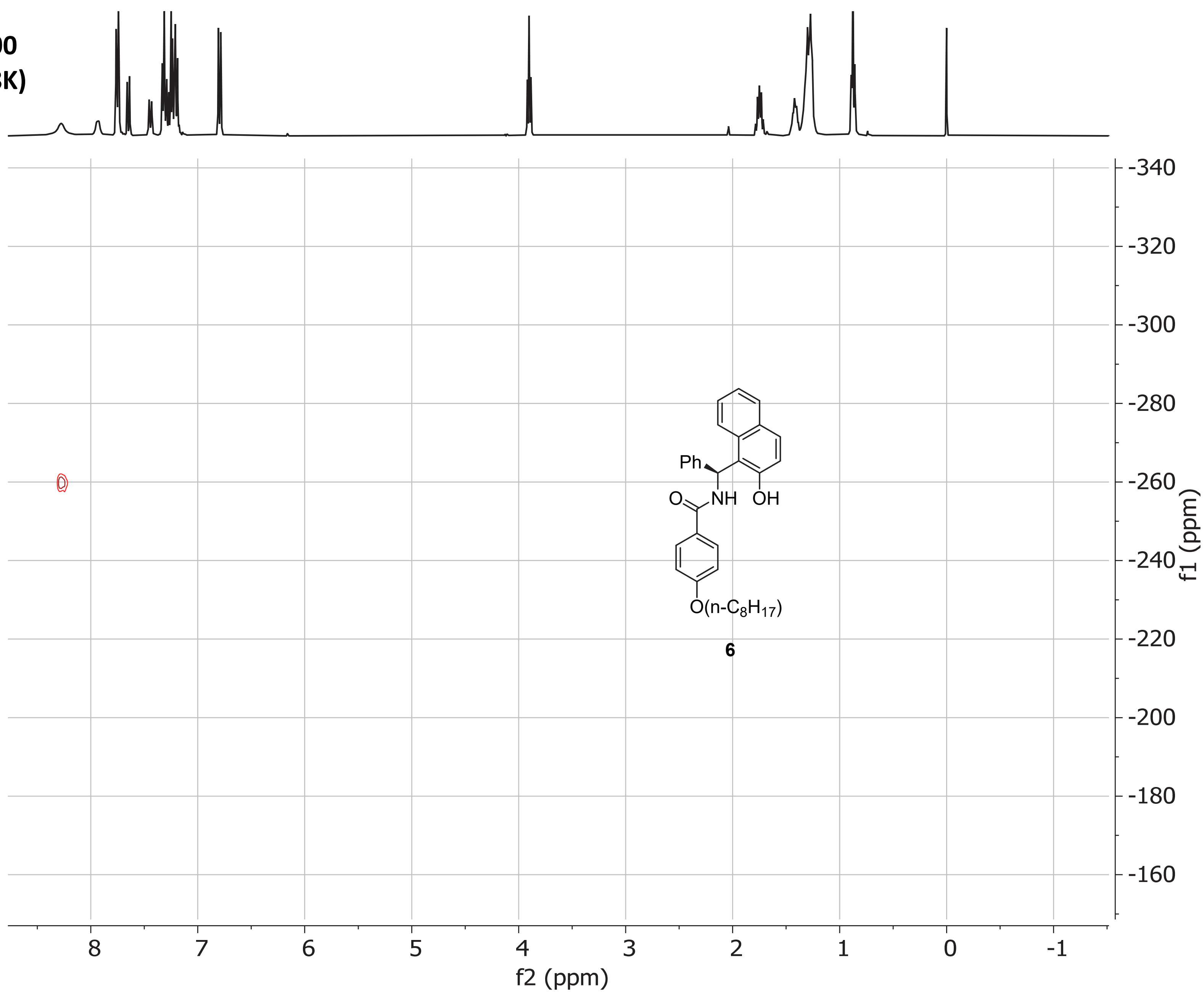

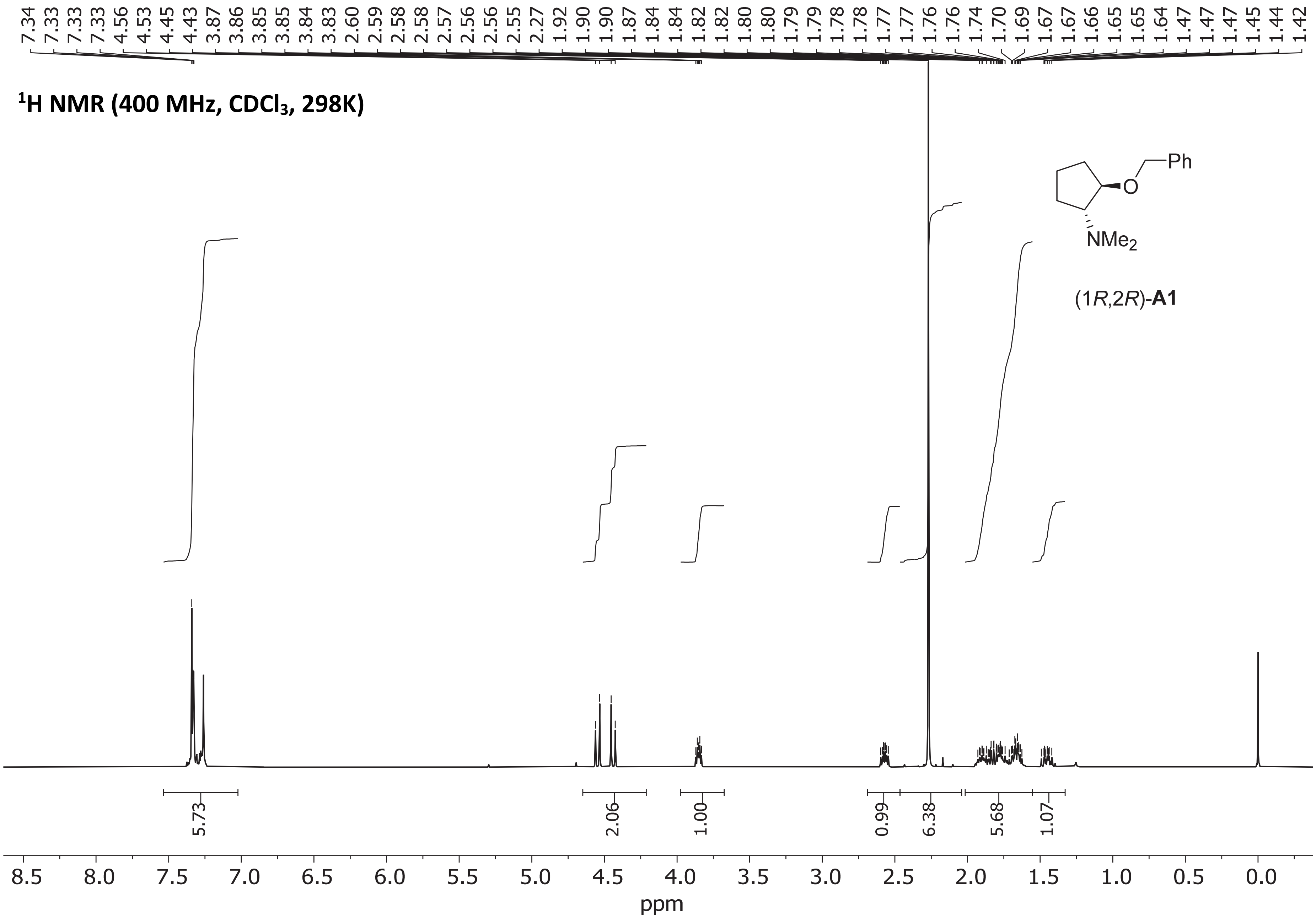
7.34 7.33 7.33 7.33 4.56 4.53 4.45 4.43 3.87 3.86 3.85 3.85 3.84 3.83 2.60 2.59 2.58 2.58 2.57 2.56 2.56 2.55 2.27 1.92 1.90 1.90 1.87 1.84 1.84 1.82 1.82 1.80 1.80 1.79 1.79 1.78 1.78 1.77 1.77 1.76 1.76 1.74 1.70 1.69 1.67 1.67 1.66 1.65 1.65 1.64 1.47 1.47 1.47 1.45 1.44 1.42
1H NMR (400 MHz, CDCl3, 298K)
Ph
O
NMe2
(1R,2R)-A1
5.73
2.06
1.00
0.99
6.38
5.68
1.07
8.5 8.0 7.5 7.0 6.5 6.0 5.5 5.0 4.5 4.0 3.5 3.0 2.5 2.0 1.5 1.0 0.5 0.0
ppm

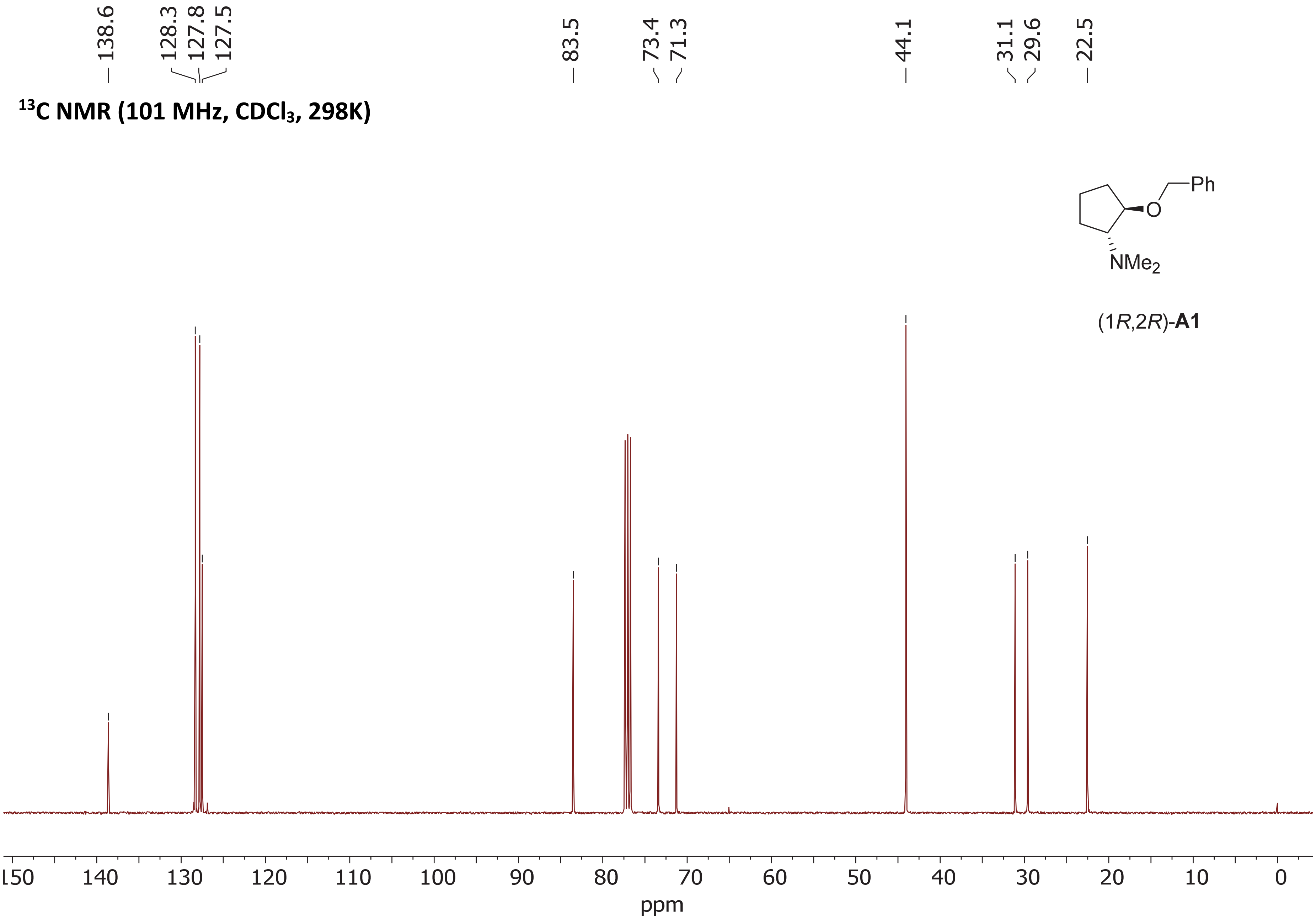
138.6
128.3
127.8
127.5
83.5
73.4
71.3
44.1
31.1
29.6
22.5
13C NMR (101 MHz, CDCl3, 298K)
Ph
O
NMe2
(1R,2R)-A1
150
140
130
120
110
100
90
80
70
60
50
40
30
20
10
0
ppm

^1H-^{13}C HSQC (400 MHz, $CDCl_3$, 298K)

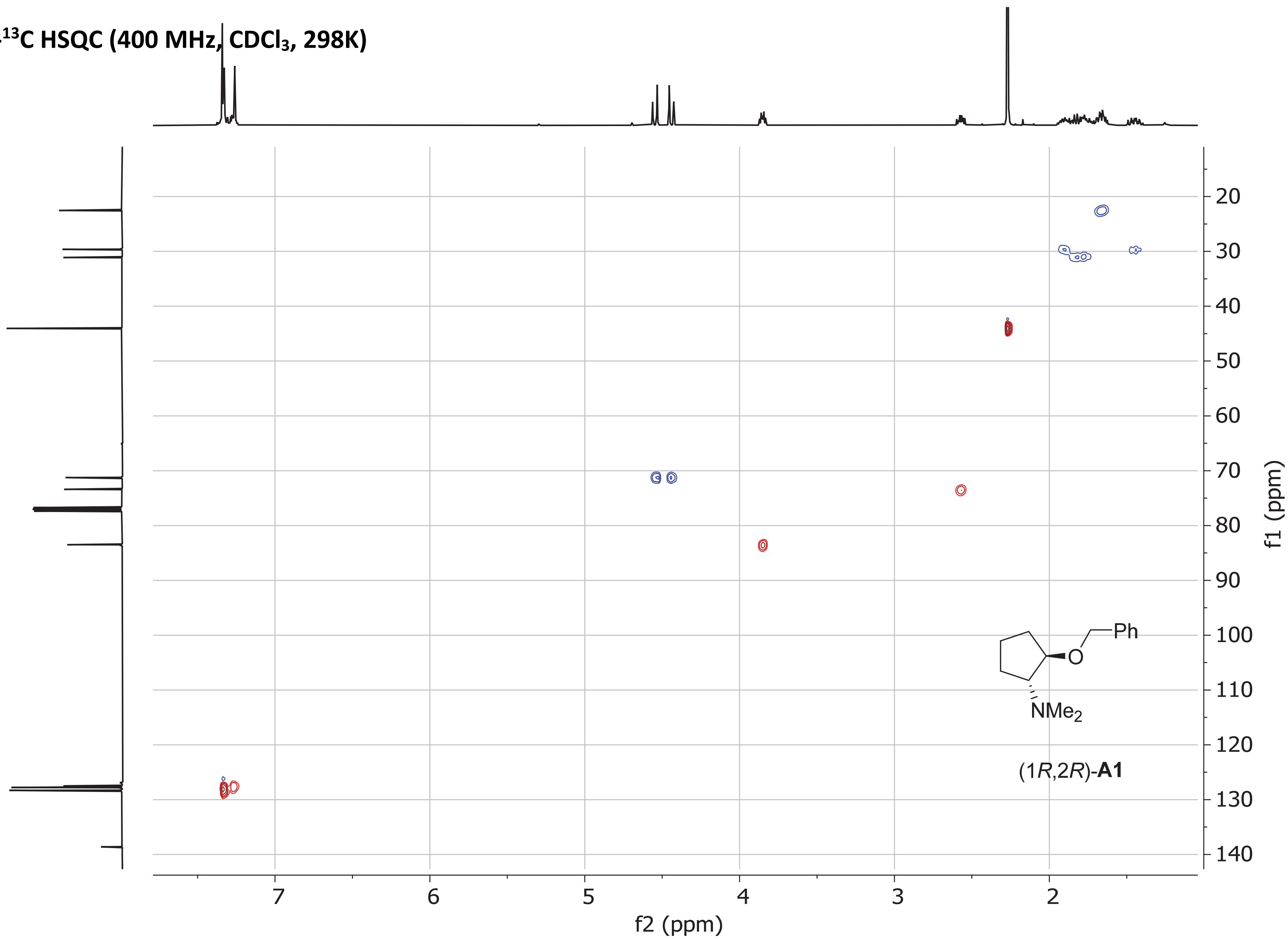

^{1}H NMR (400 MHz, $CDCl_3$, 298K)

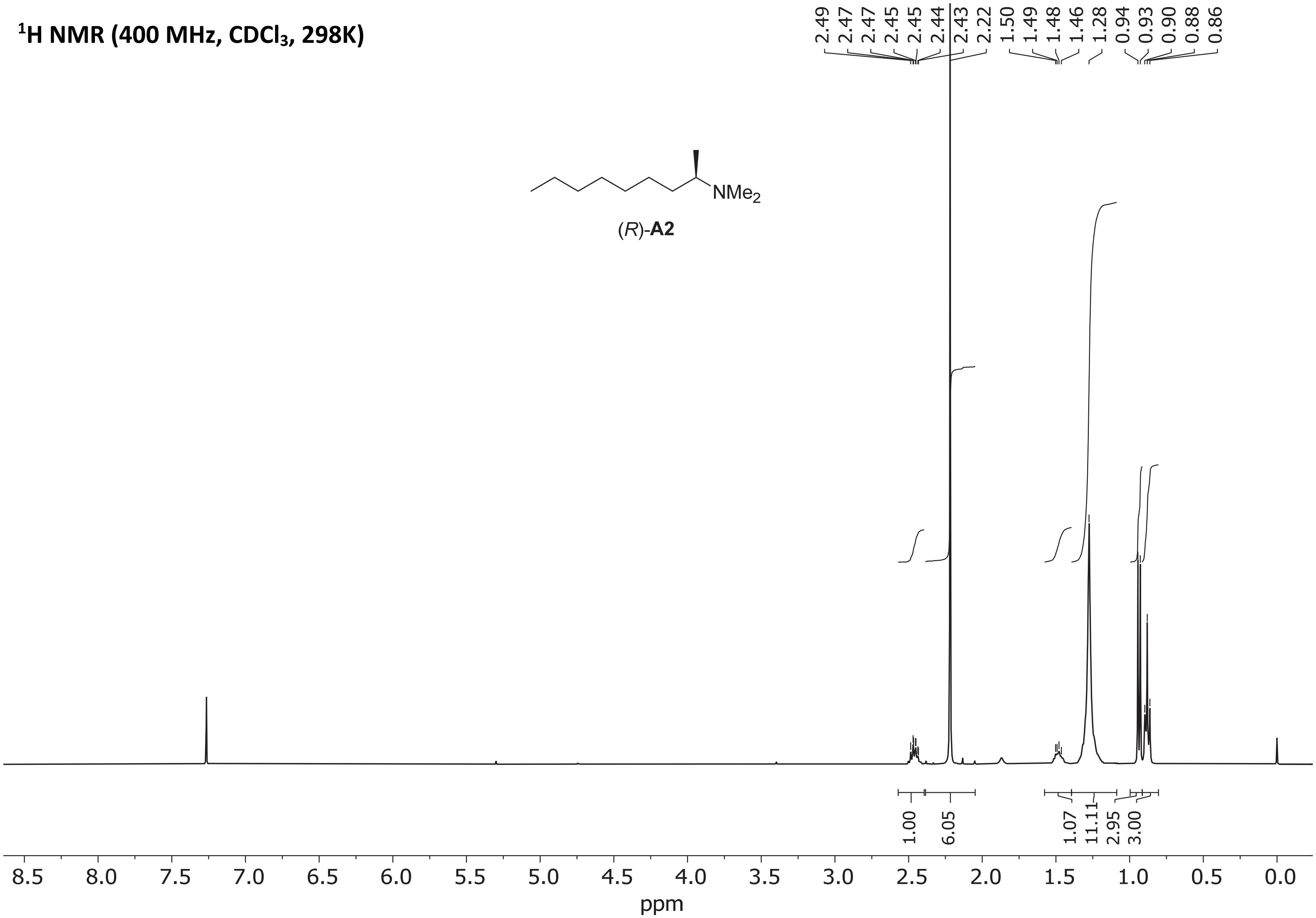

^{13}C NMR (101 MHz, $CDCl_3$, 298K)

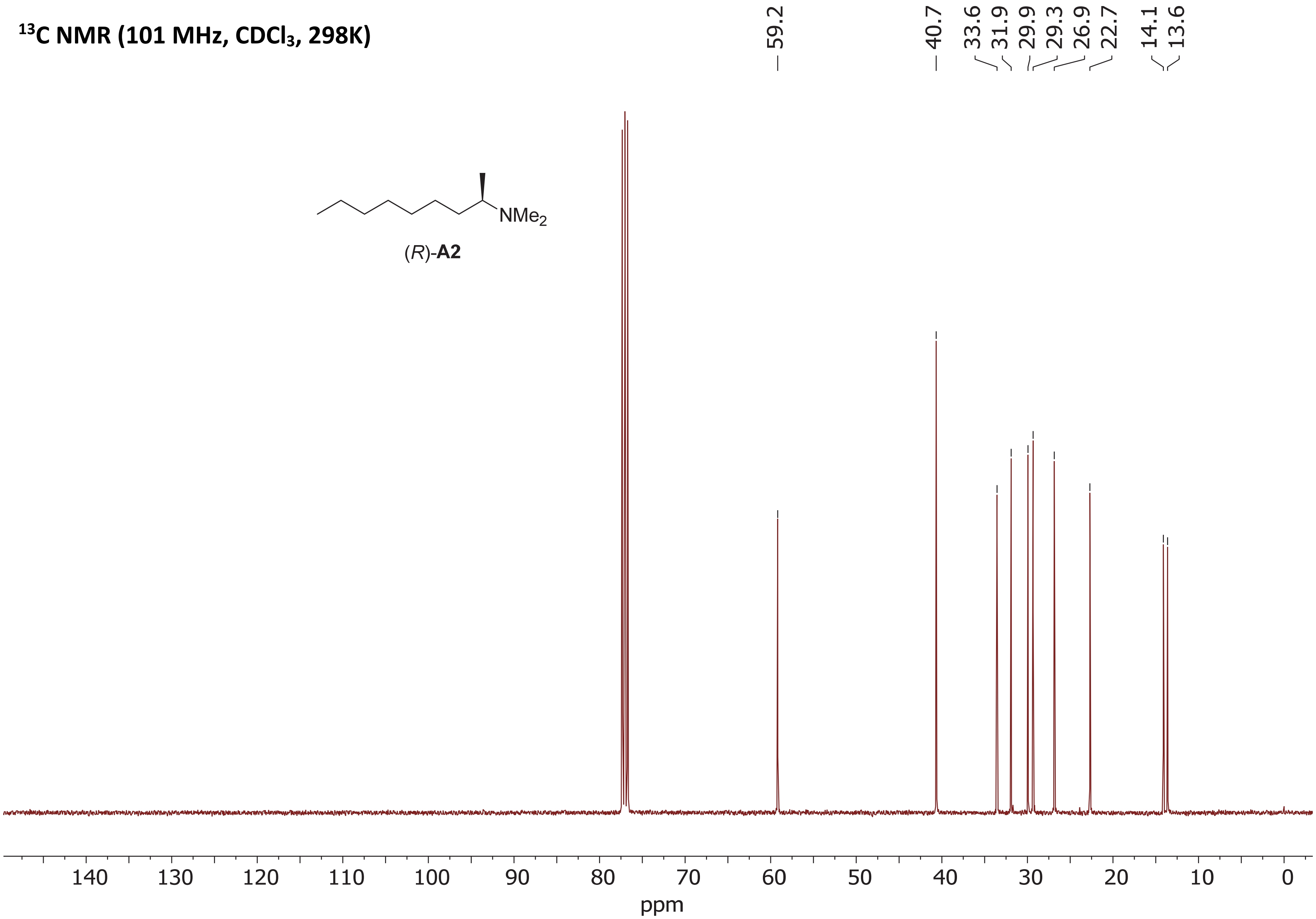

^{1}H-^{13}C HSQC (400 MHz, CDCl$_3$, 298K)

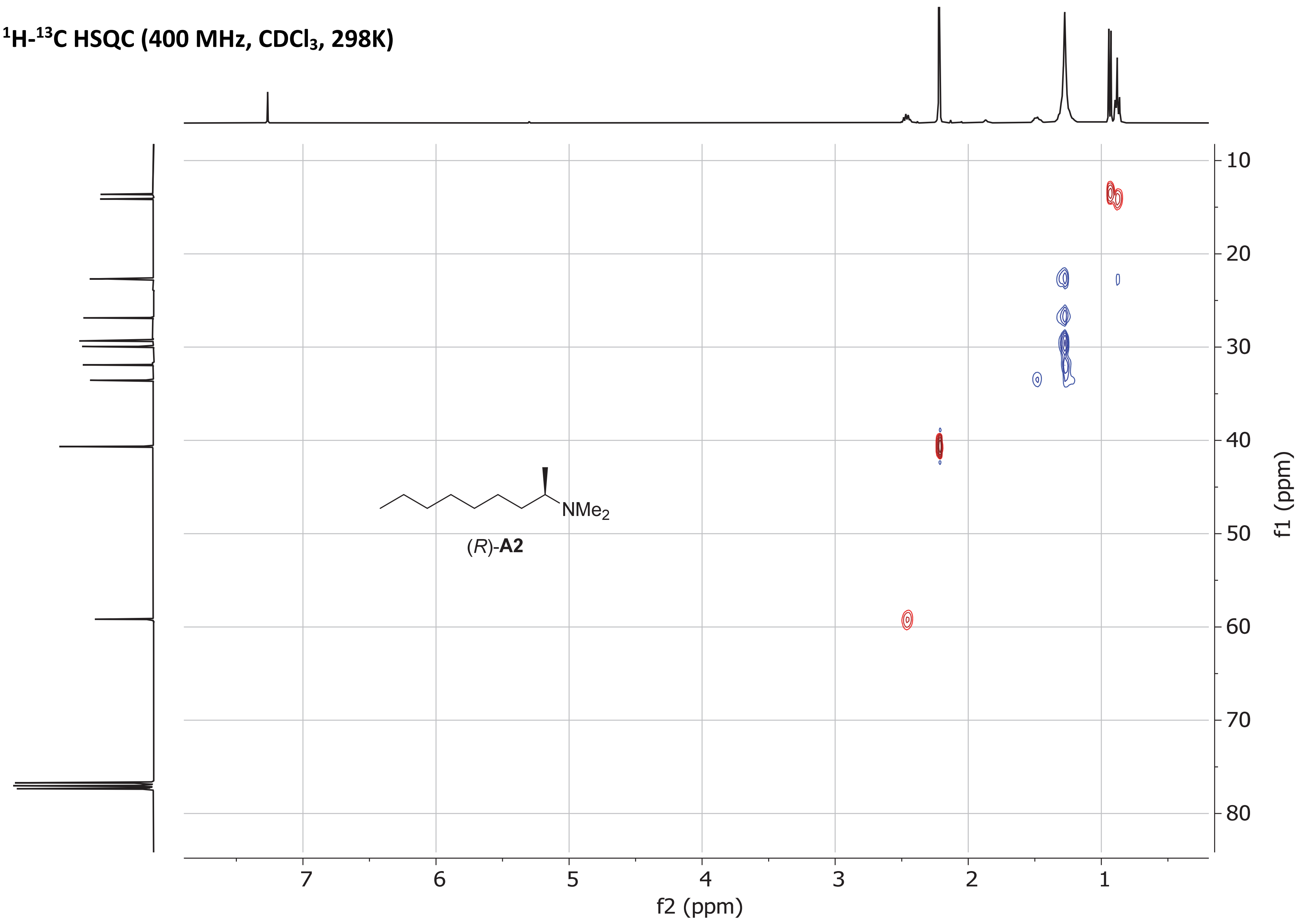

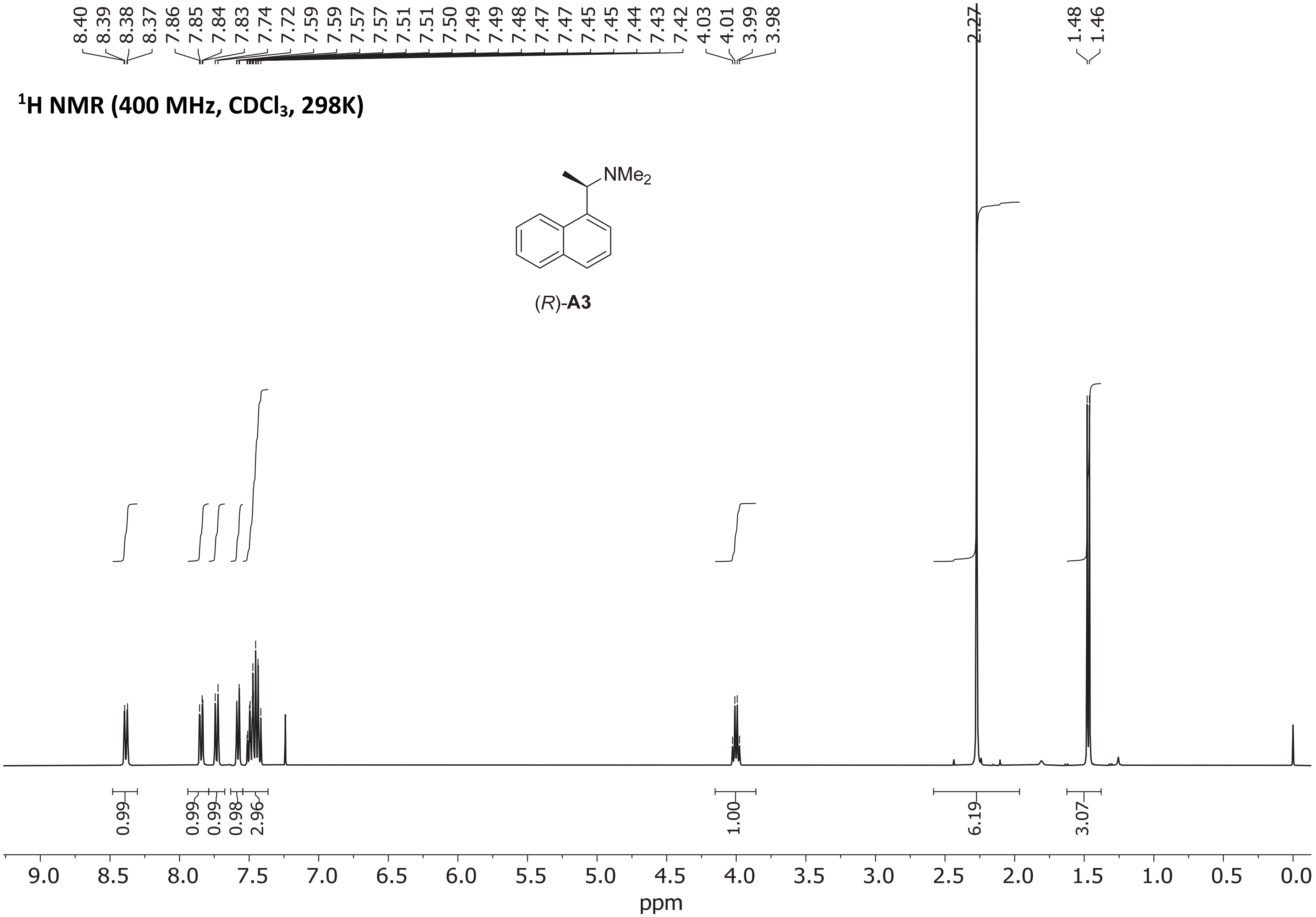
8.40
8.39
8.38
8.37
7.86
7.85
7.84
7.83
7.74
7.72
7.59
7.59
7.57
7.57
7.51
7.51
7.50
7.49
7.49
7.48
7.47
7.47
7.45
7.45
7.44
7.43
7.42
4.03
4.01
3.99
3.98
2.27
1.48
1.46
¹H NMR (400 MHz, CDCl₃, 298K)
NMe₂
(R)-A3
0.99
0.99
0.99
0.98
2.96
1.00
6.19
3.07
9.0
8.5
8.0
7.5
7.0
6.5
6.0
5.5
5.0
4.5
4.0
3.5
3.0
2.5
2.0
1.5
1.0
0.5
0.0
ppm

^{13}C NMR (101 MHz, $CDCl_3$, 298K)

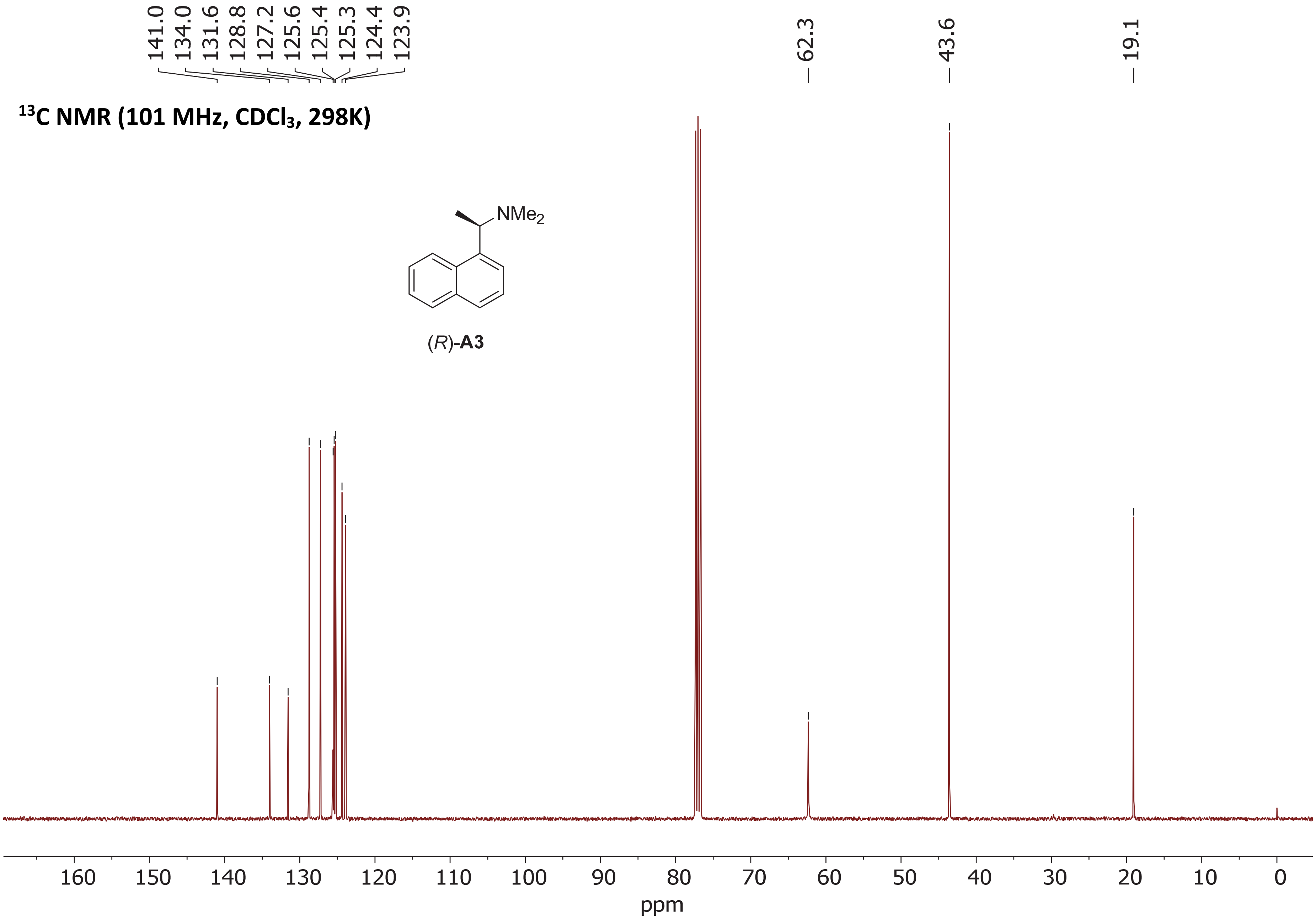

^{1}H-^{13}C HSQC (400 MHz, $CDCl_3$, 298K)

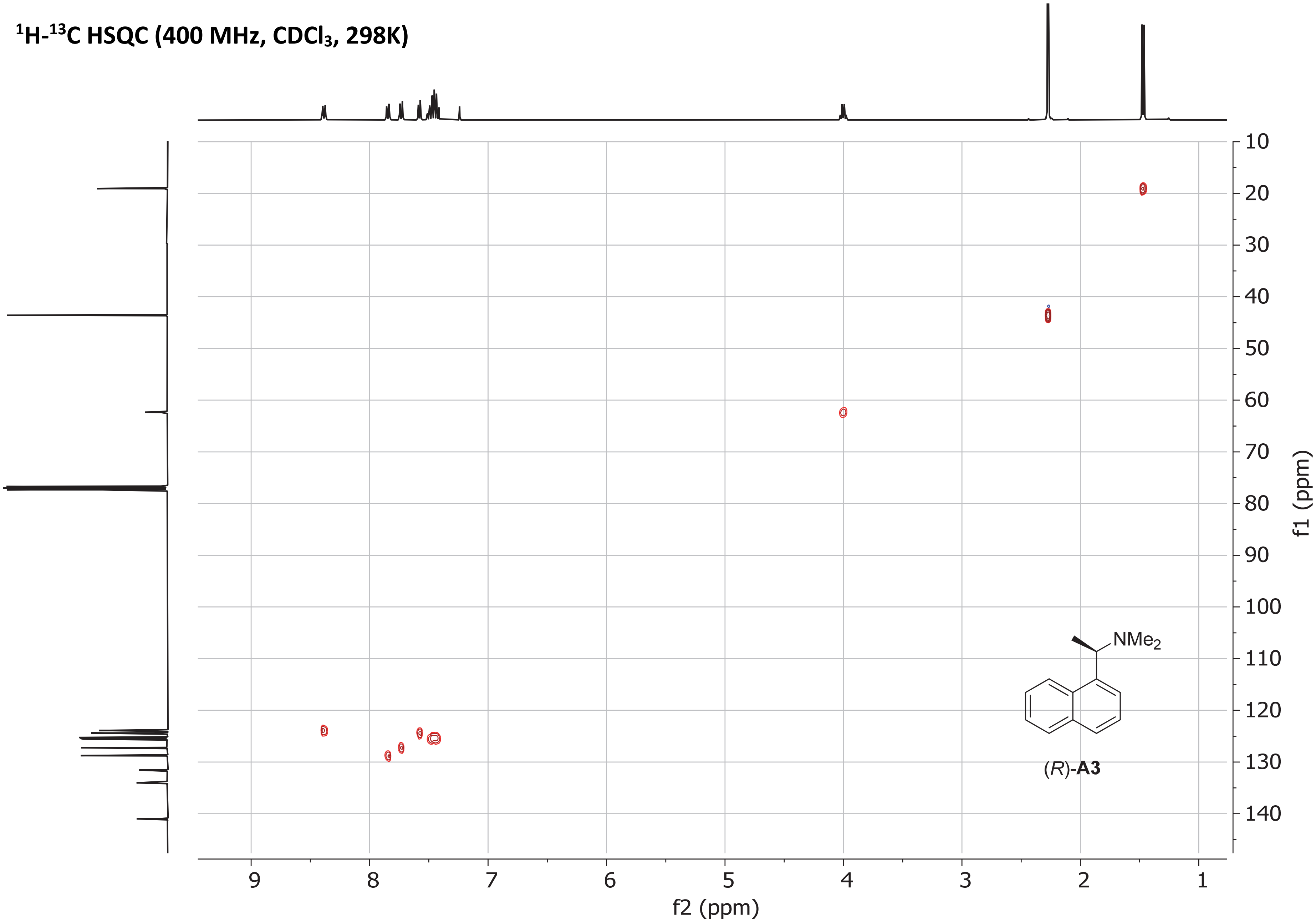

^{1}H NMR (400 MHz, $CDCl_3$, 298K)

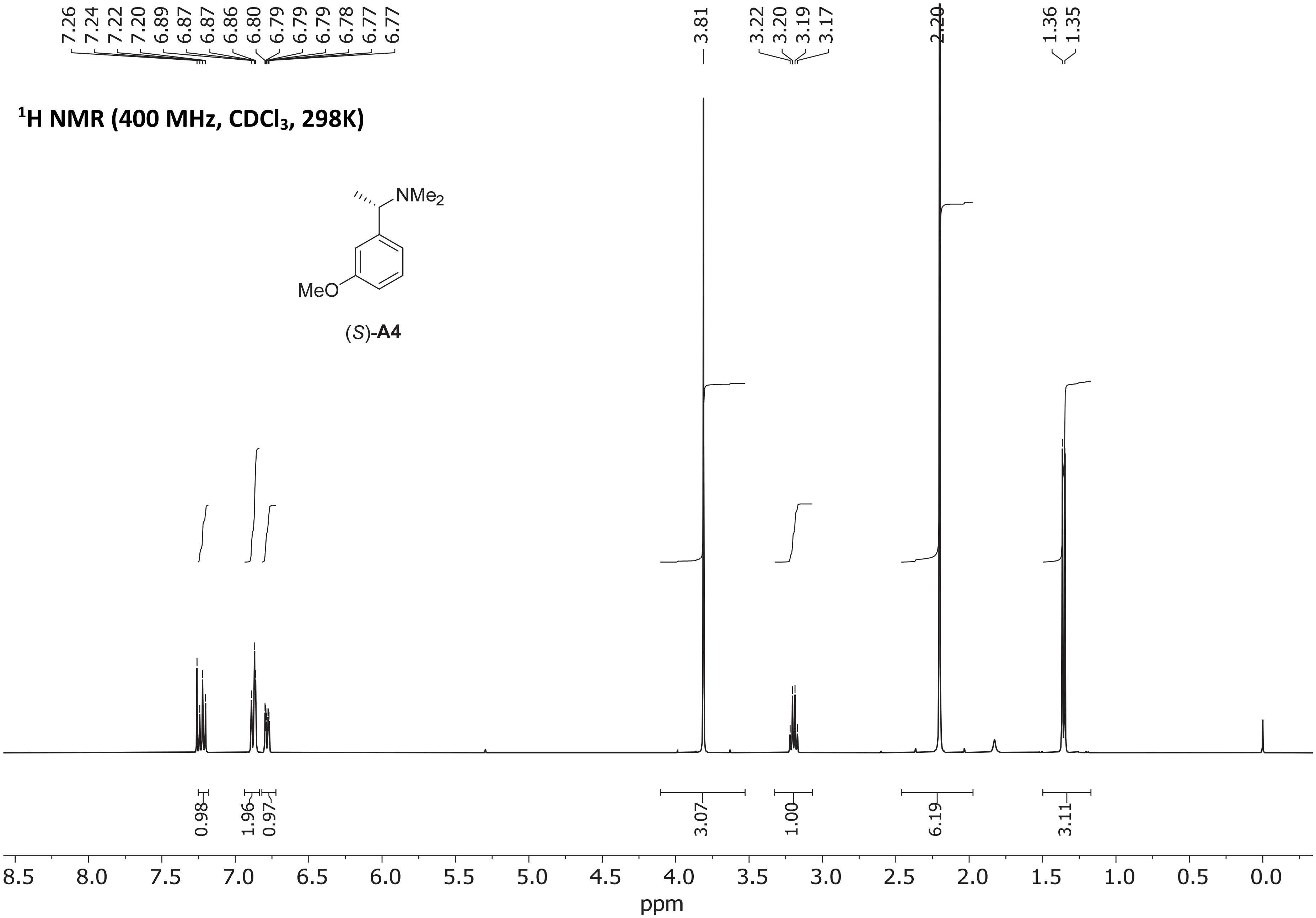

^{13}C NMR (101 MHz, $CDCl_3$, 298K)

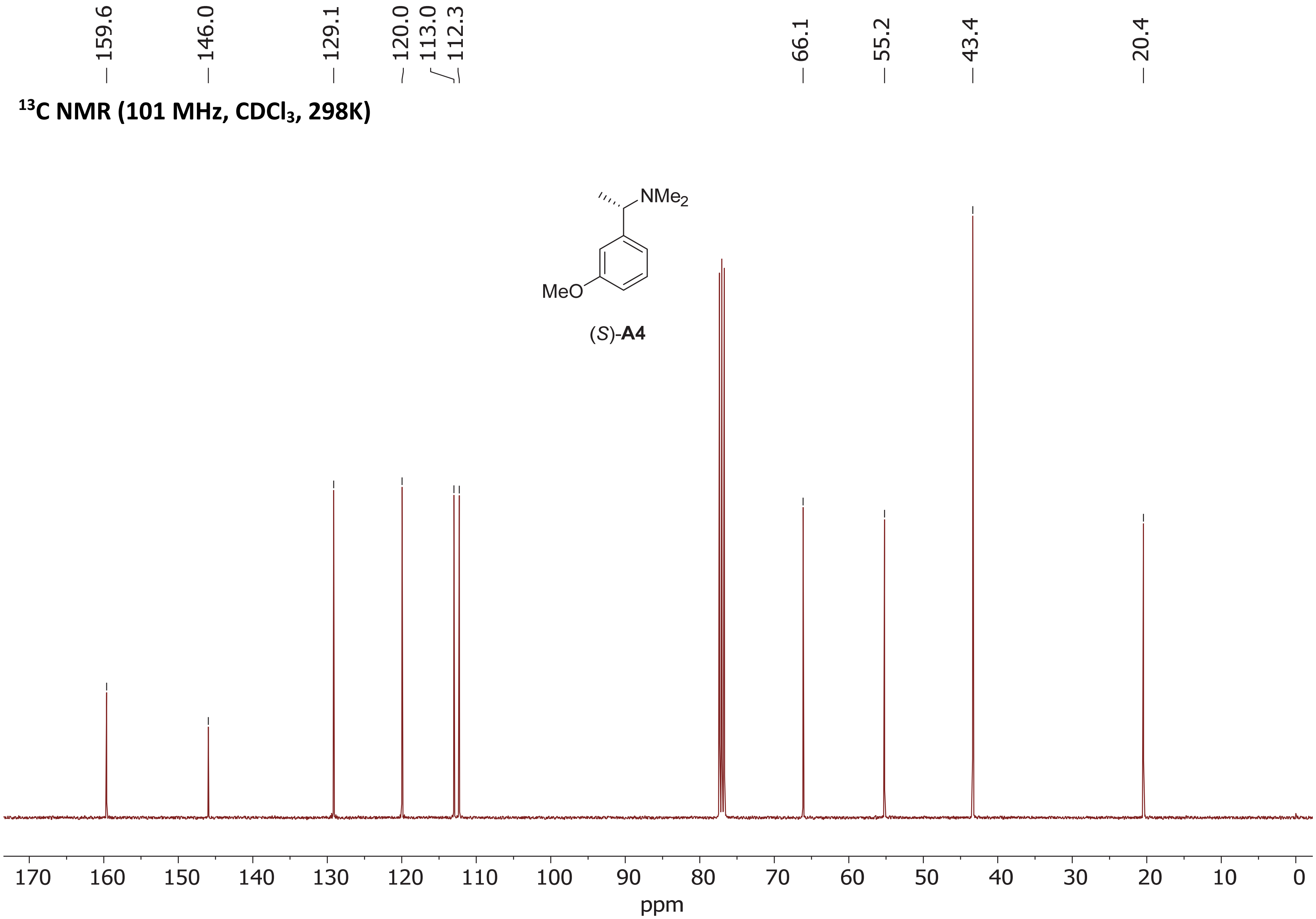

^1H-^{13}C HSQC (400 MHz, $CDCl_3$, 298K)

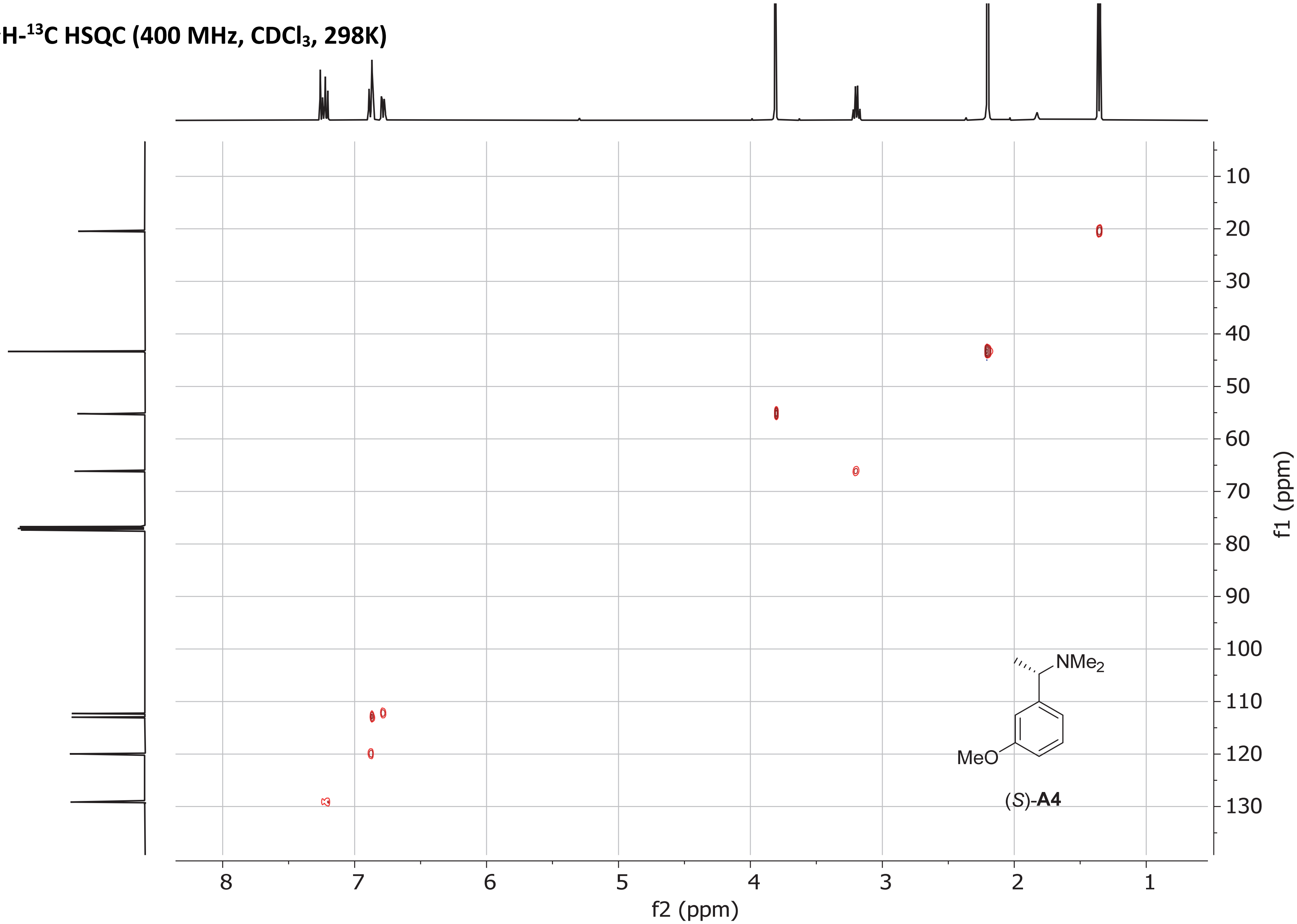

^{1}H NMR (400 MHz, $CDCl_3$, 298K)

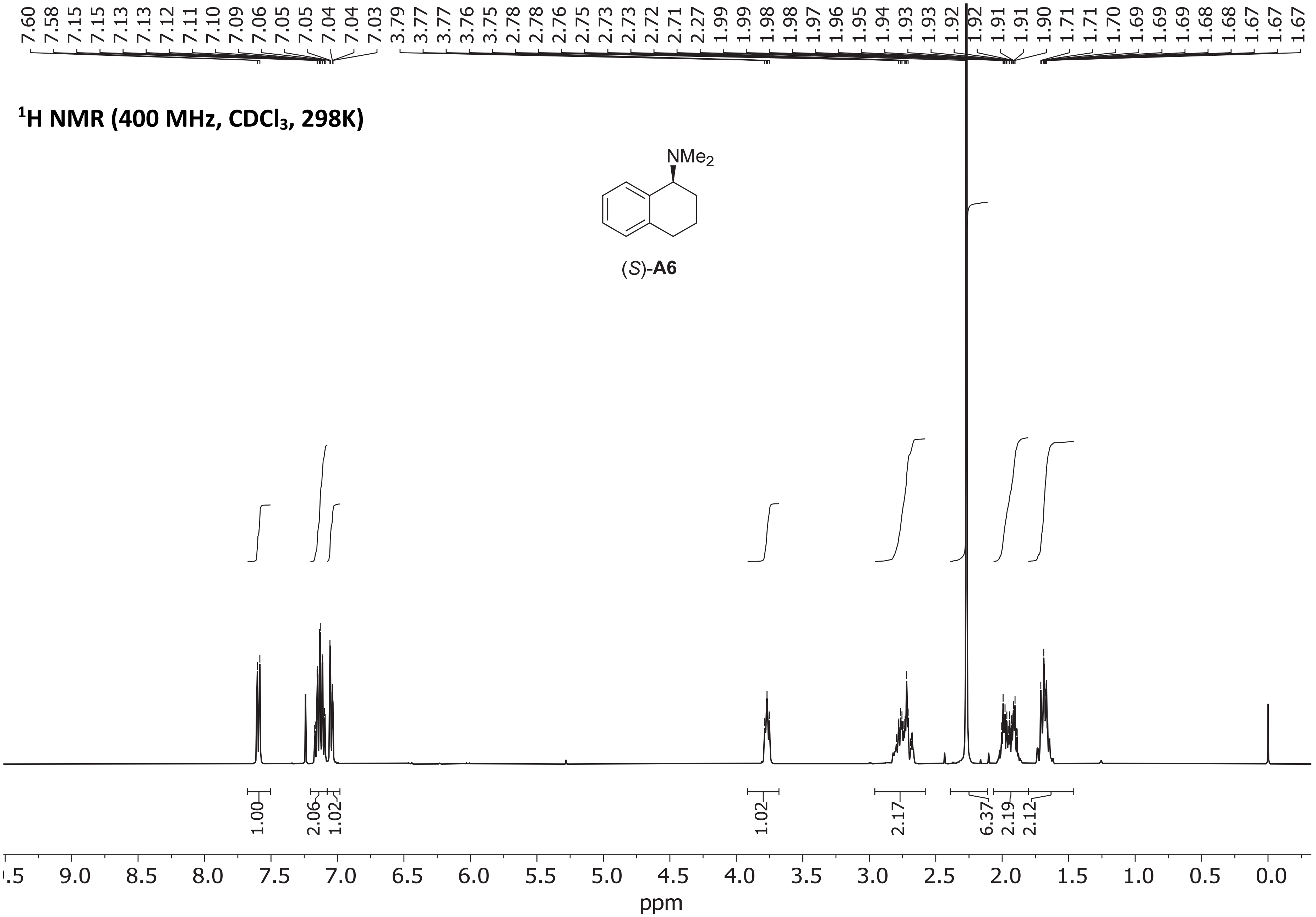

^{13}C NMR (101 MHz, $CDCl_3$, 298K)

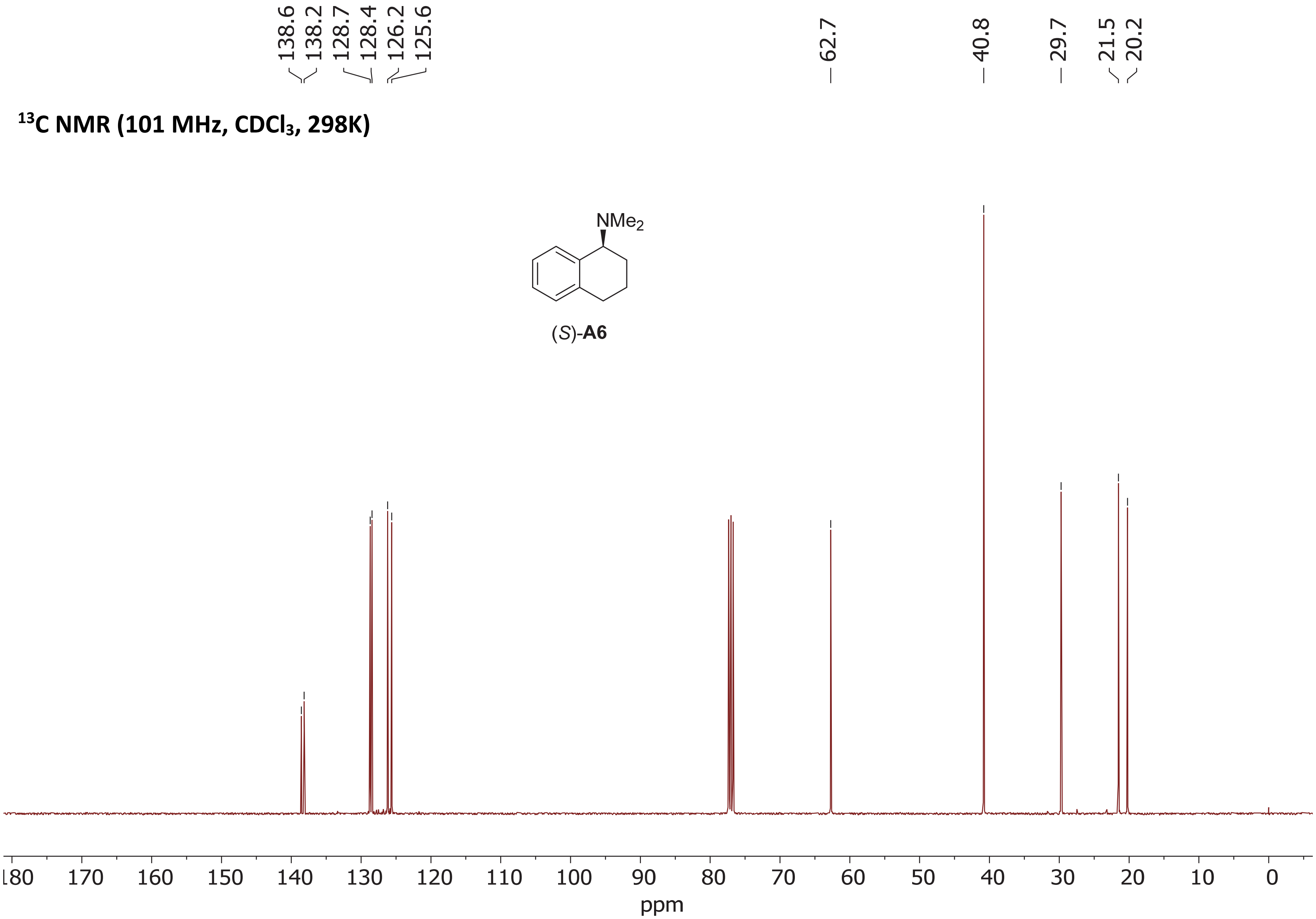

^1H-^{13}C HSQC (400 MHz, $CDCl_3$, 298K)

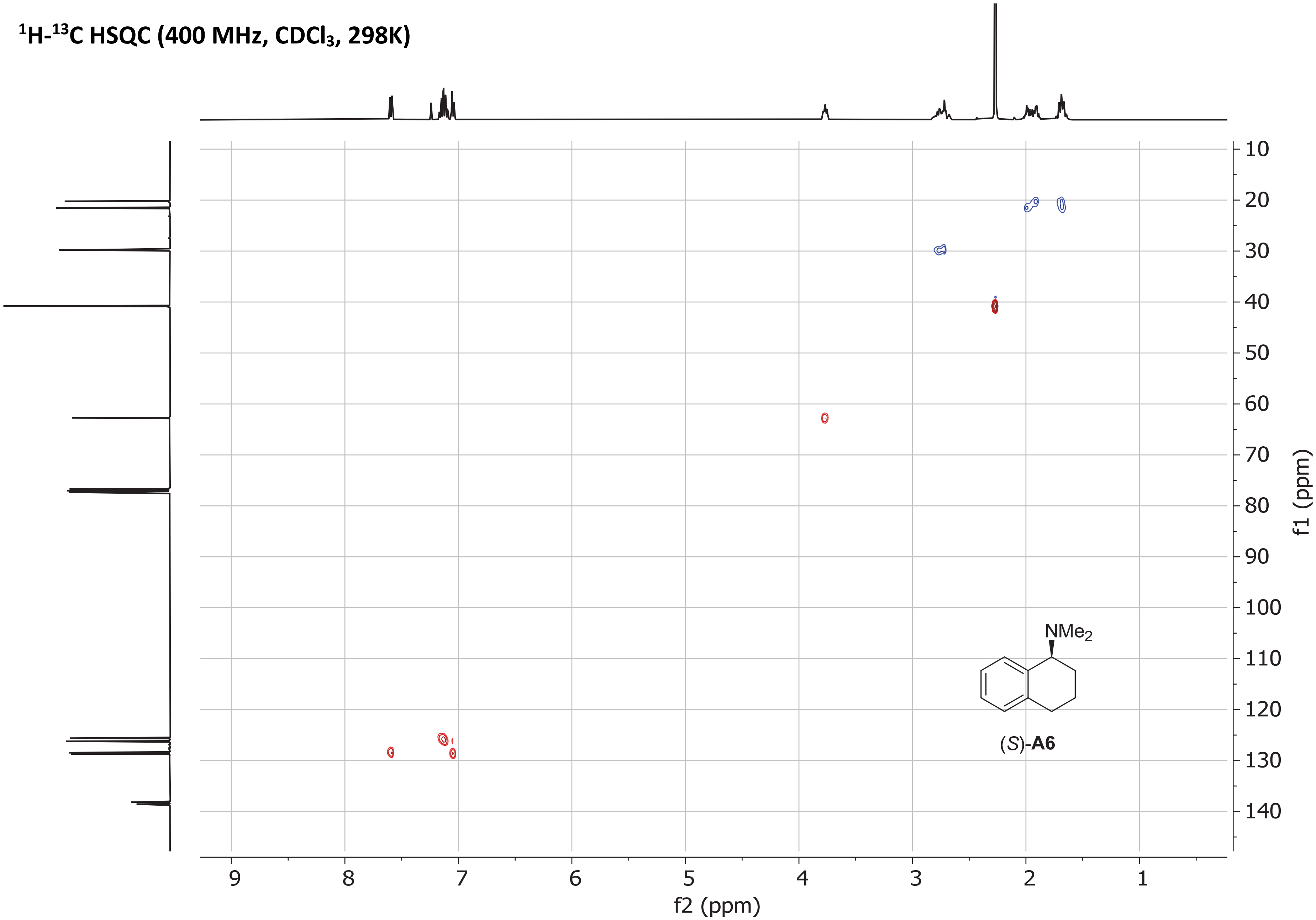

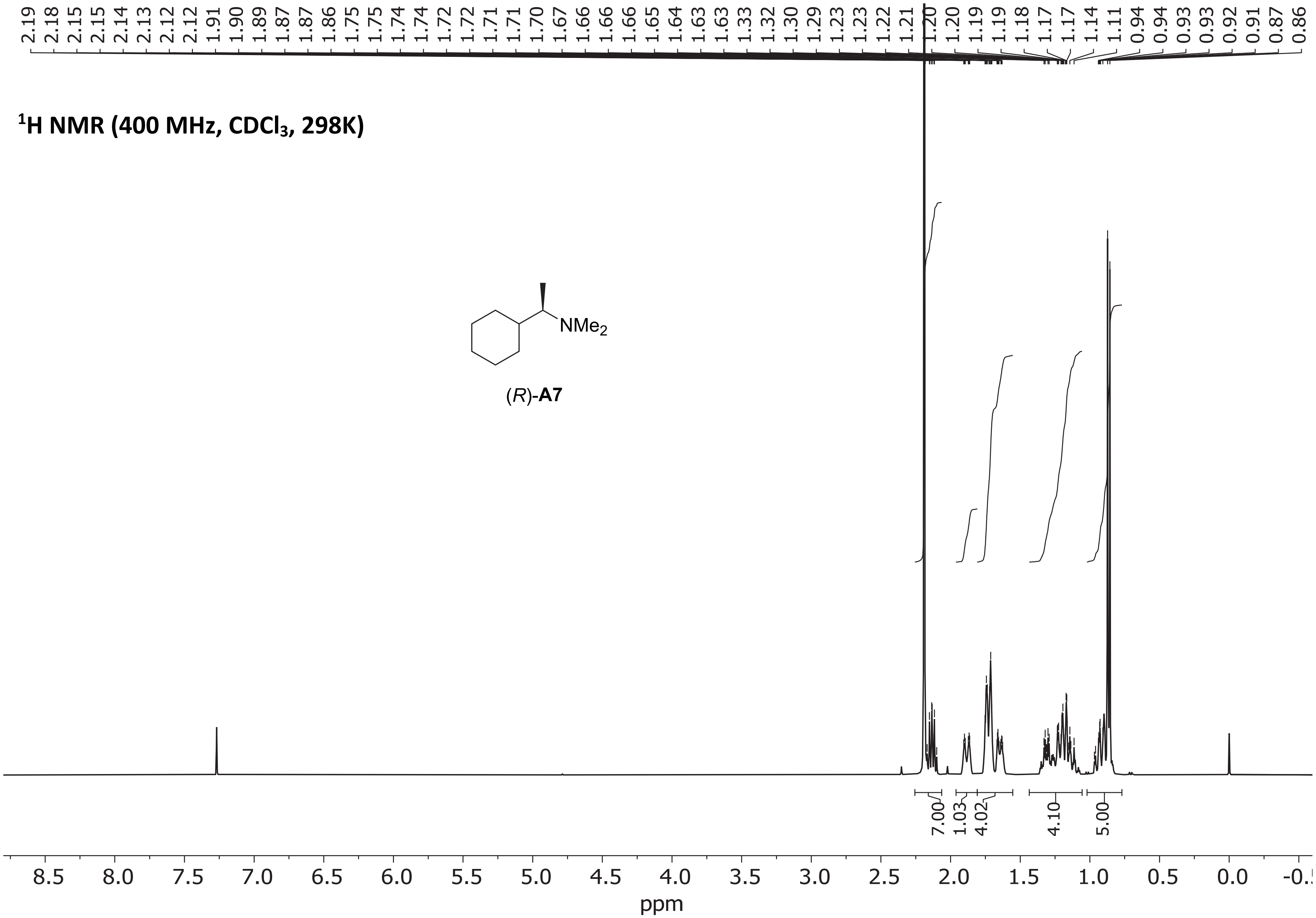

¹H NMR (400 MHz, CDCl₃, 298K)
NMe₂
(R)-A7
ppm

^{13}C NMR (101 MHz, $CDCl_3$, 298K)

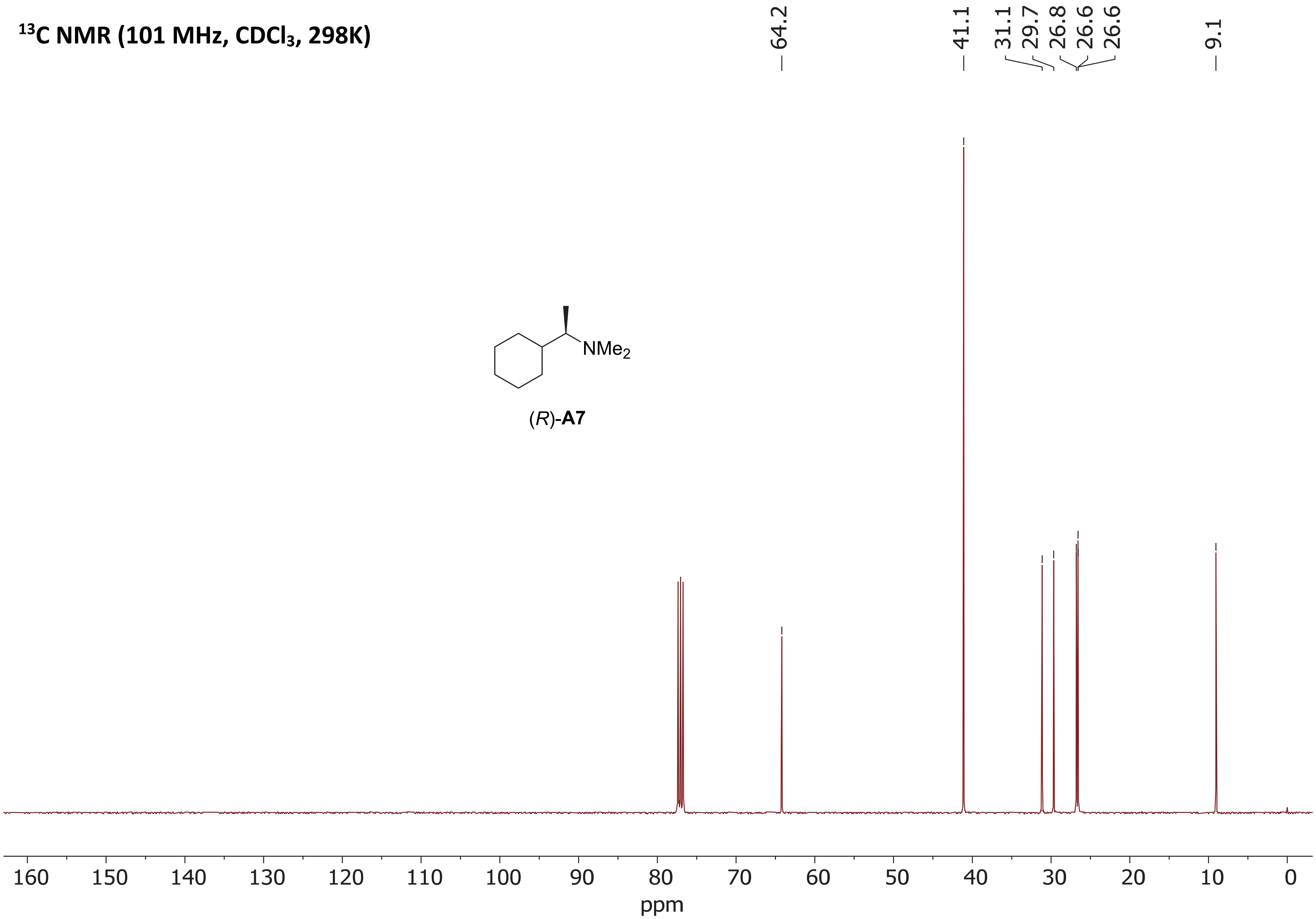

^{1}H-^{13}C HSQC (400 MHz, $CDCl_3$, 298K)

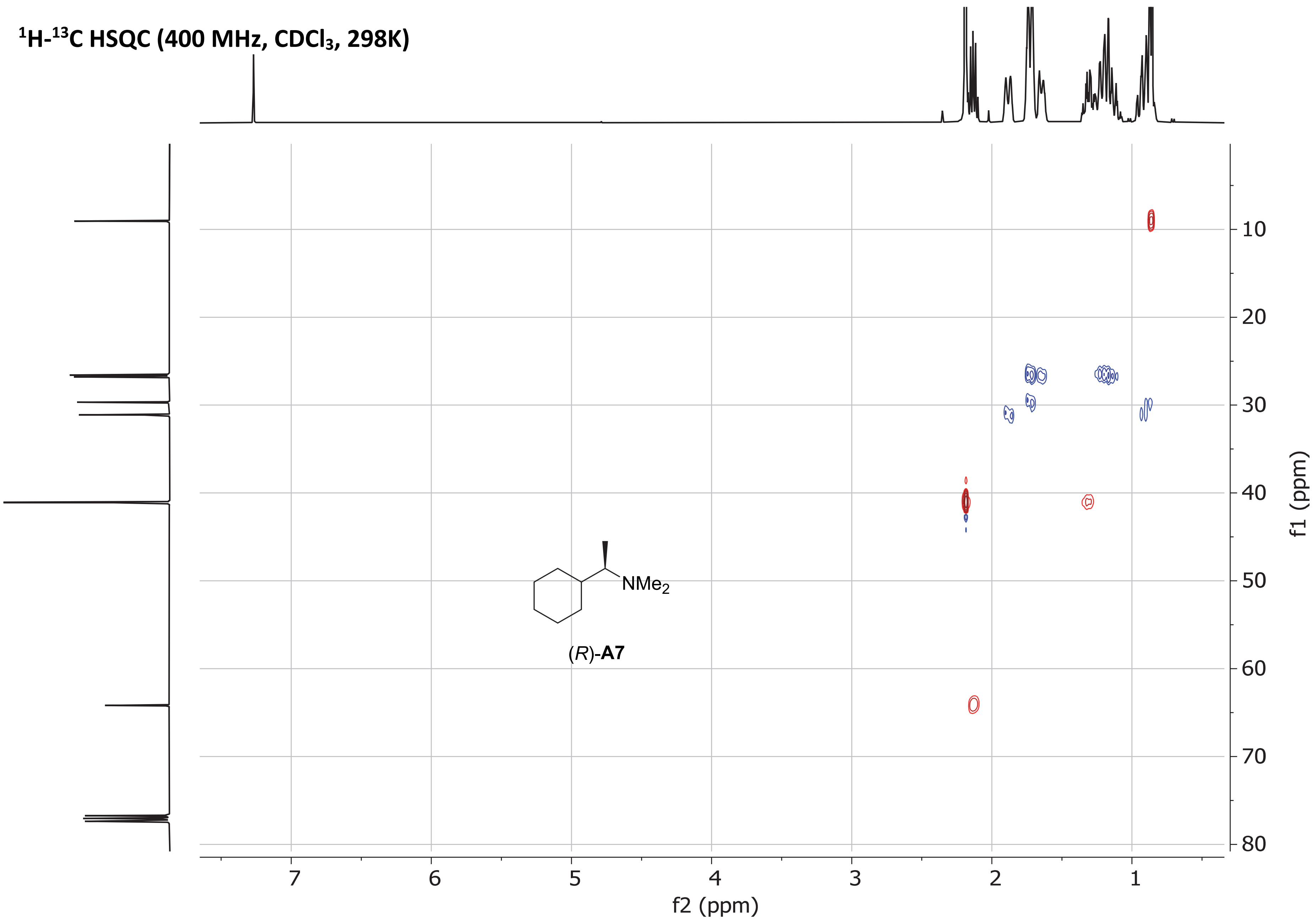

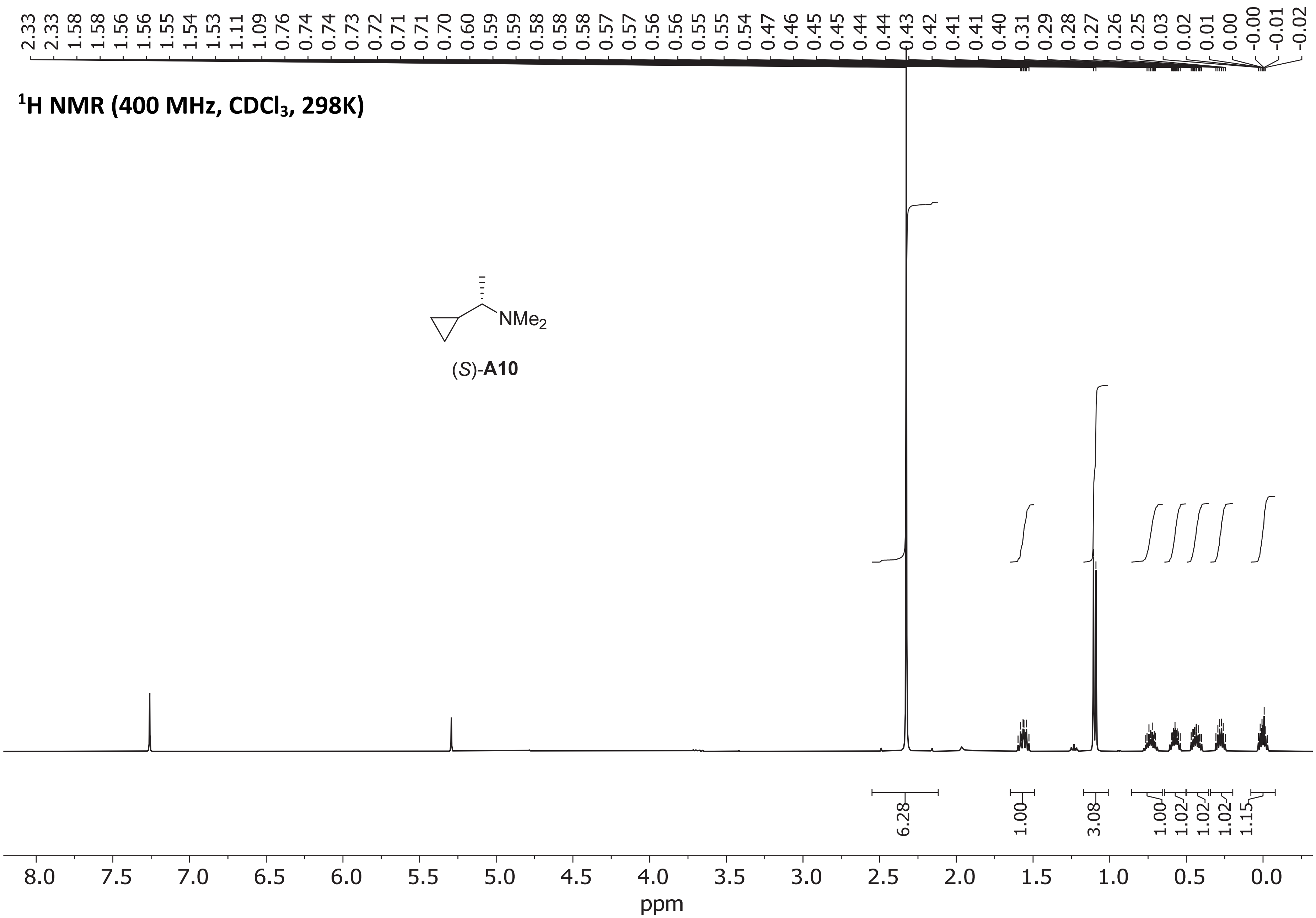
2.33
2.33
1.58
1.58
1.56
1.56
1.55
1.54
1.53
1.11
1.09
0.76
0.74
0.74
0.73
0.72
0.71
0.71
0.70
0.60
0.59
0.59
0.58
0.58
0.58
0.57
0.57
0.56
0.56
0.55
0.55
0.54
0.47
0.46
0.45
0.45
0.44
0.44
0.43
0.42
0.41
0.41
0.40
0.31
0.29
0.28
0.27
0.26
0.25
0.03
0.02
0.01
0.00
-0.00
-0.01
-0.02
¹H NMR (400 MHz, CDCl₃, 298K)
NMe₂
(S)-A10
6.28
1.00
3.08
1.00
1.02
1.02
1.02
1.15
8.0
7.5
7.0
6.5
6.0
5.5
5.0
4.5
4.0
3.5
3.0
2.5
2.0
1.5
1.0
0.5
0.0
ppm

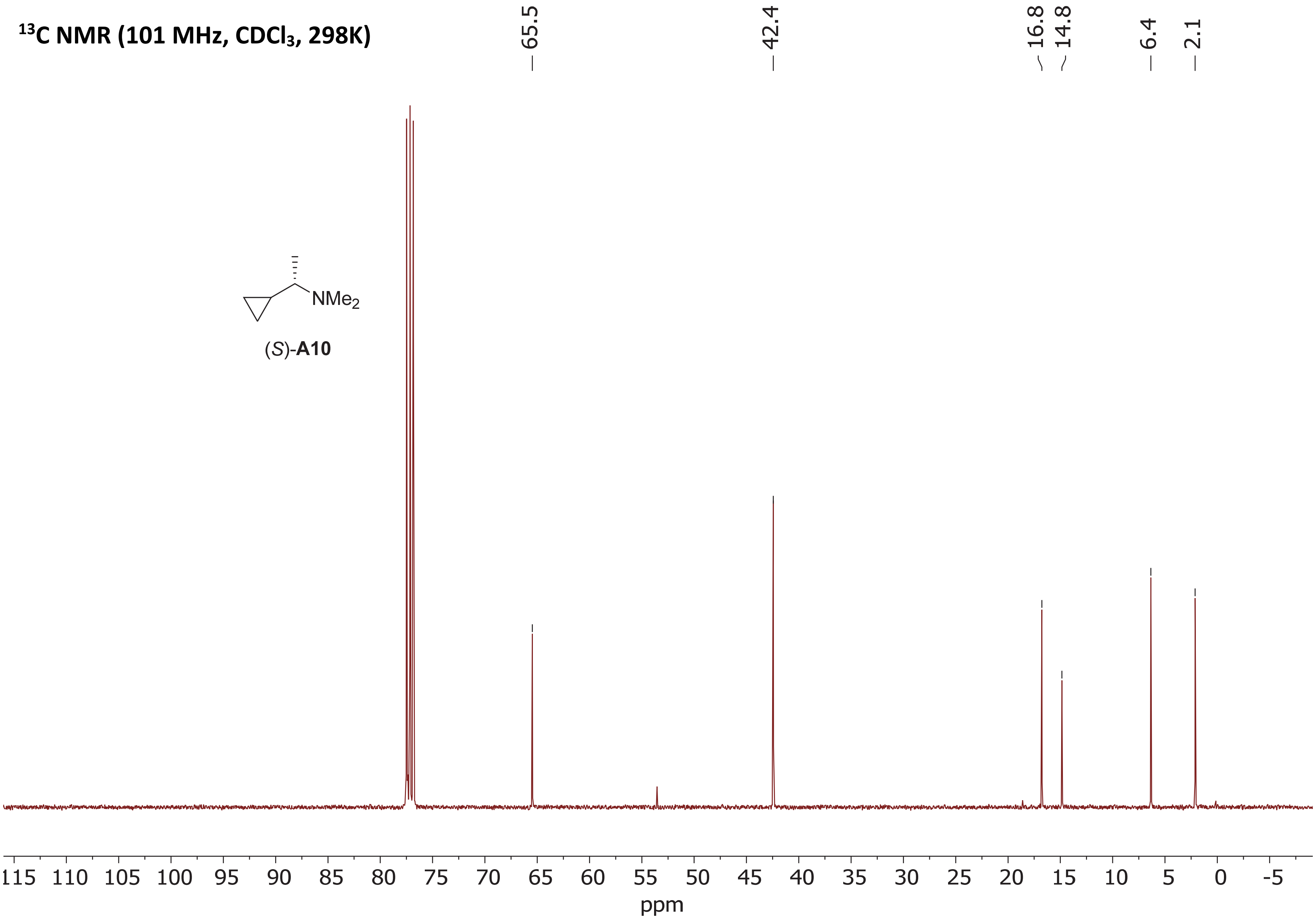

13C NMR (101 MHz, CDCl3, 298K)
65.5
42.4
16.8
14.8
6.4
2.1
NMe2
(S)-A10
115
110
105
100
95
90
85
80
75
70
65
60
55
50
45
40
35
30
25
20
15
10
5
0
-5
ppm

^{1}H-^{13}C HSQC (400 MHz, $CDCl_3$, 298K)

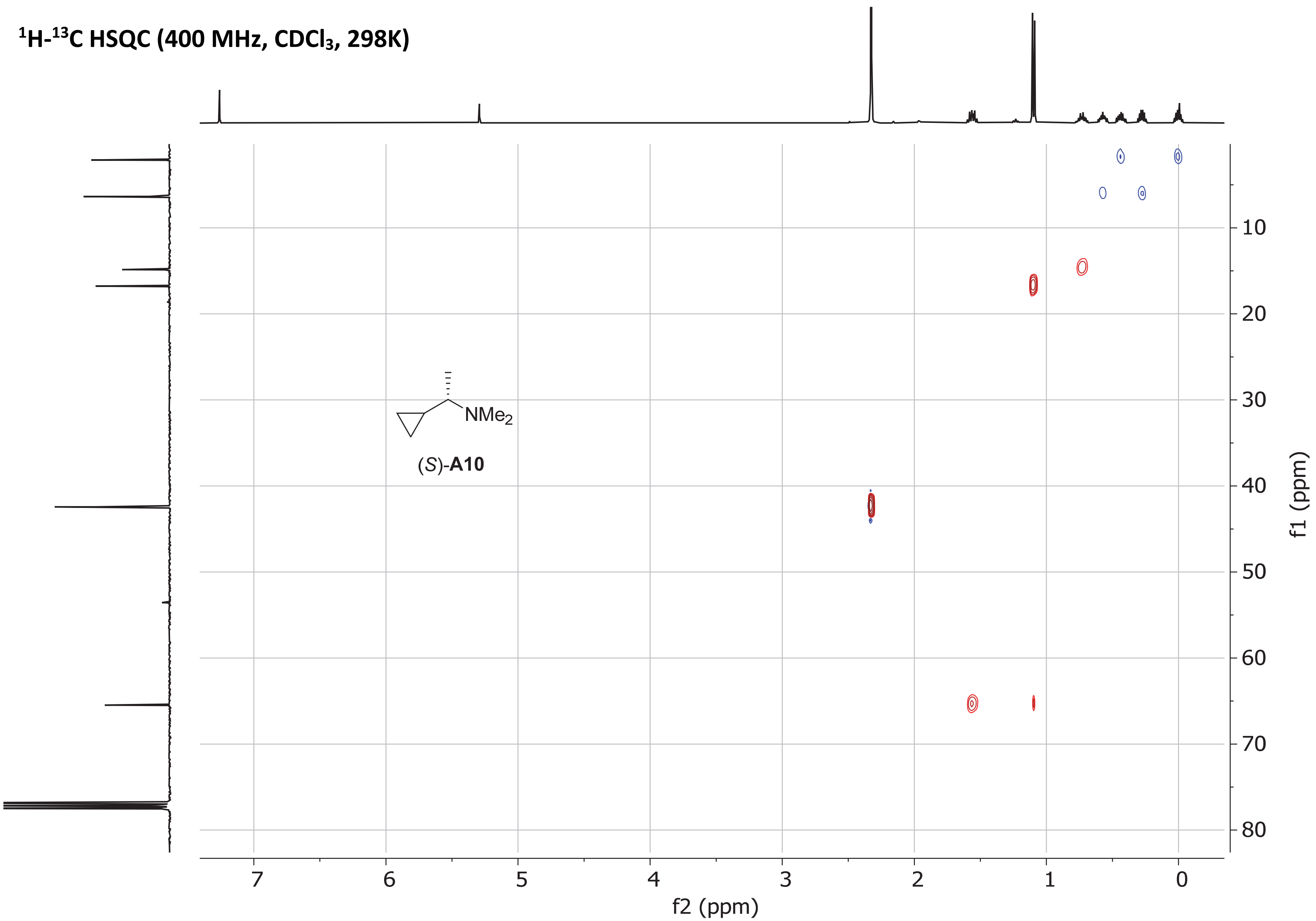

^{1}H NMR (400 MHz, $CDCl_3$, 298K)

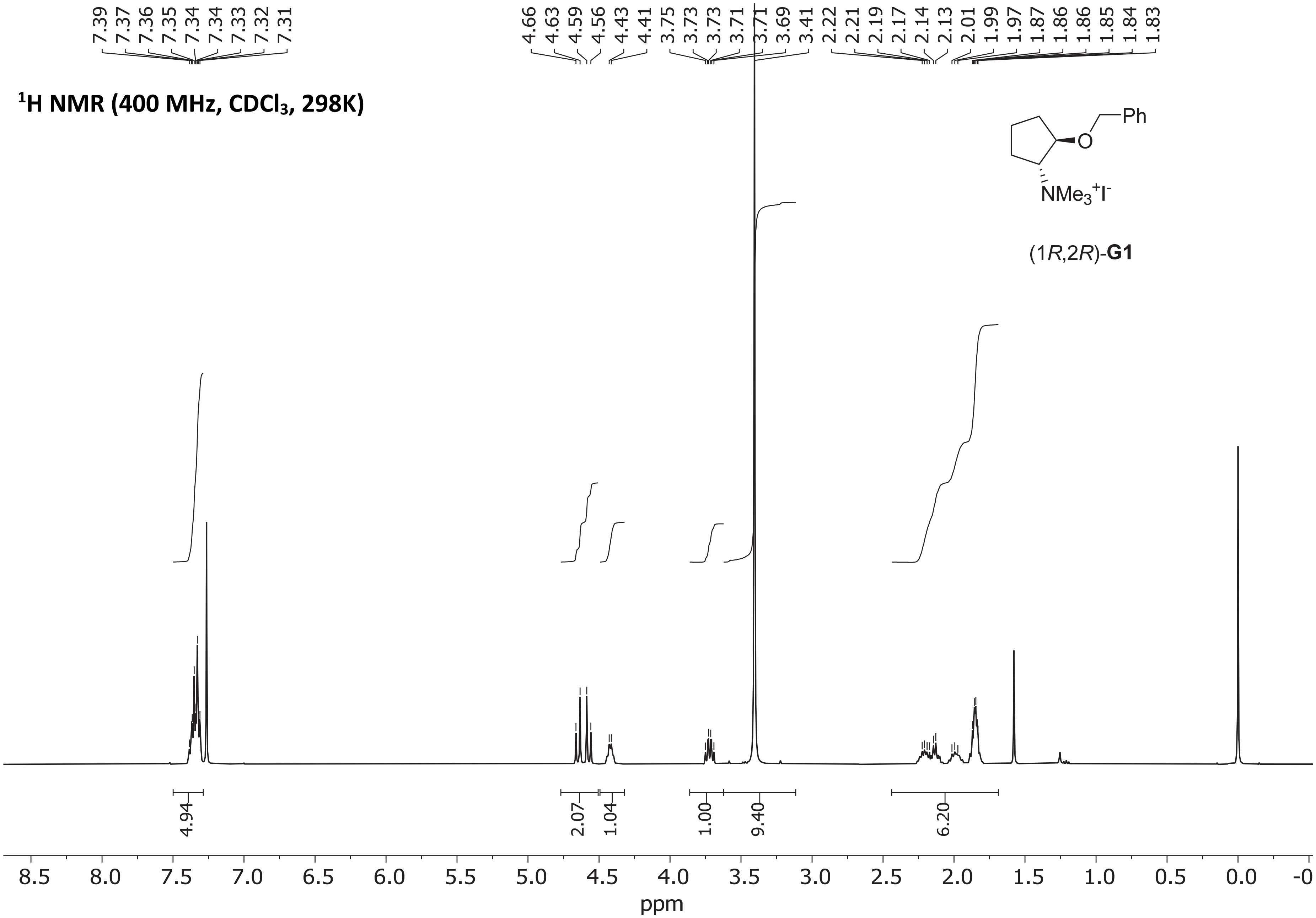

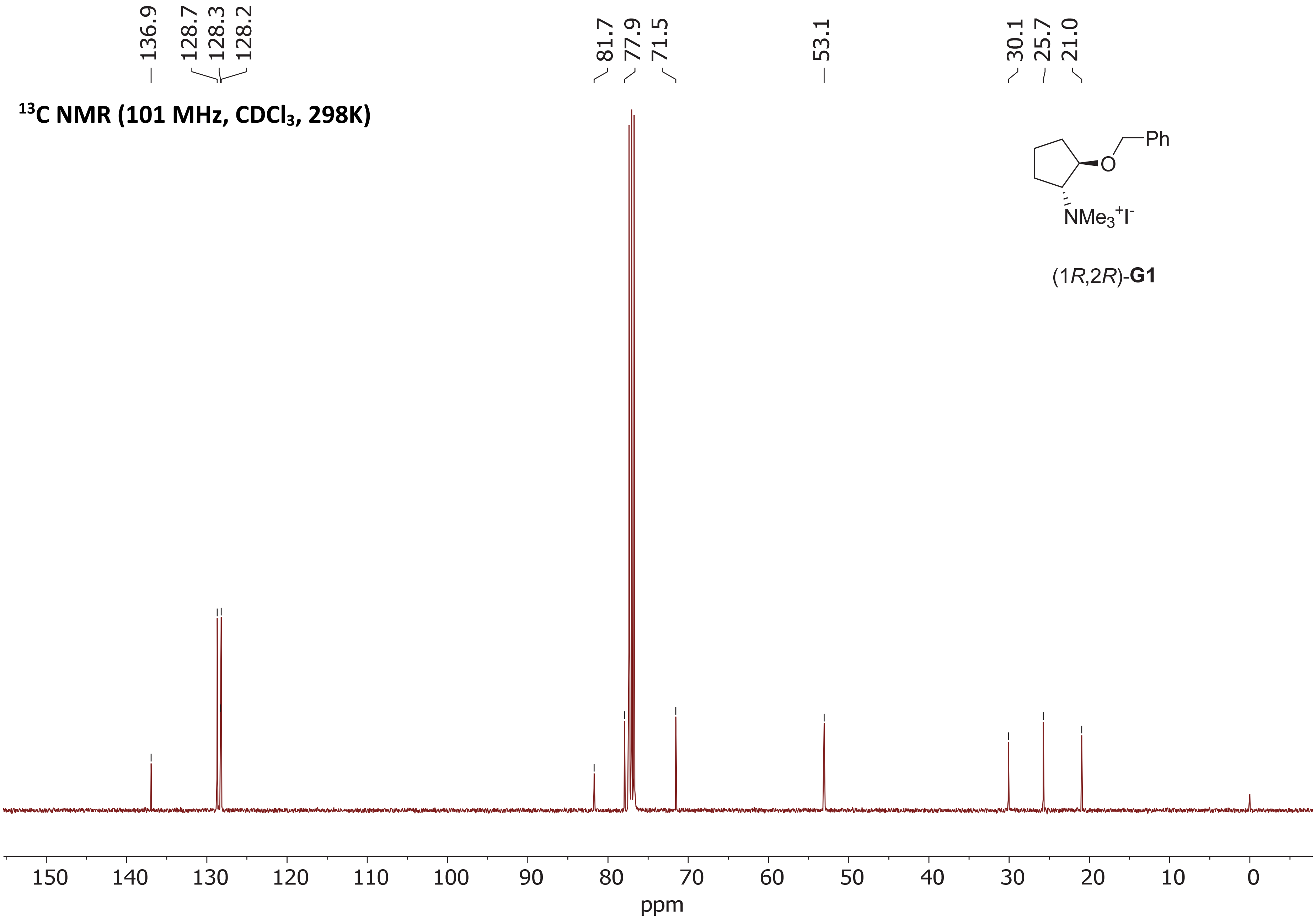

136.9
128.7
128.3
128.2
81.7
77.9
71.5
53.1
30.1
25.7
21.0
^{13}C NMR (101 MHz, $CDCl_3$, 298K)
Ph
O
$NMe_3^+I^-$
(1*R*,2*R*)-**G1**
150
140
130
120
110
100
90
80
70
60
50
40
30
20
10
0
ppm

^{1}H-^{13}C HSQC (400 MHz, $CDCl_3$, 298K)

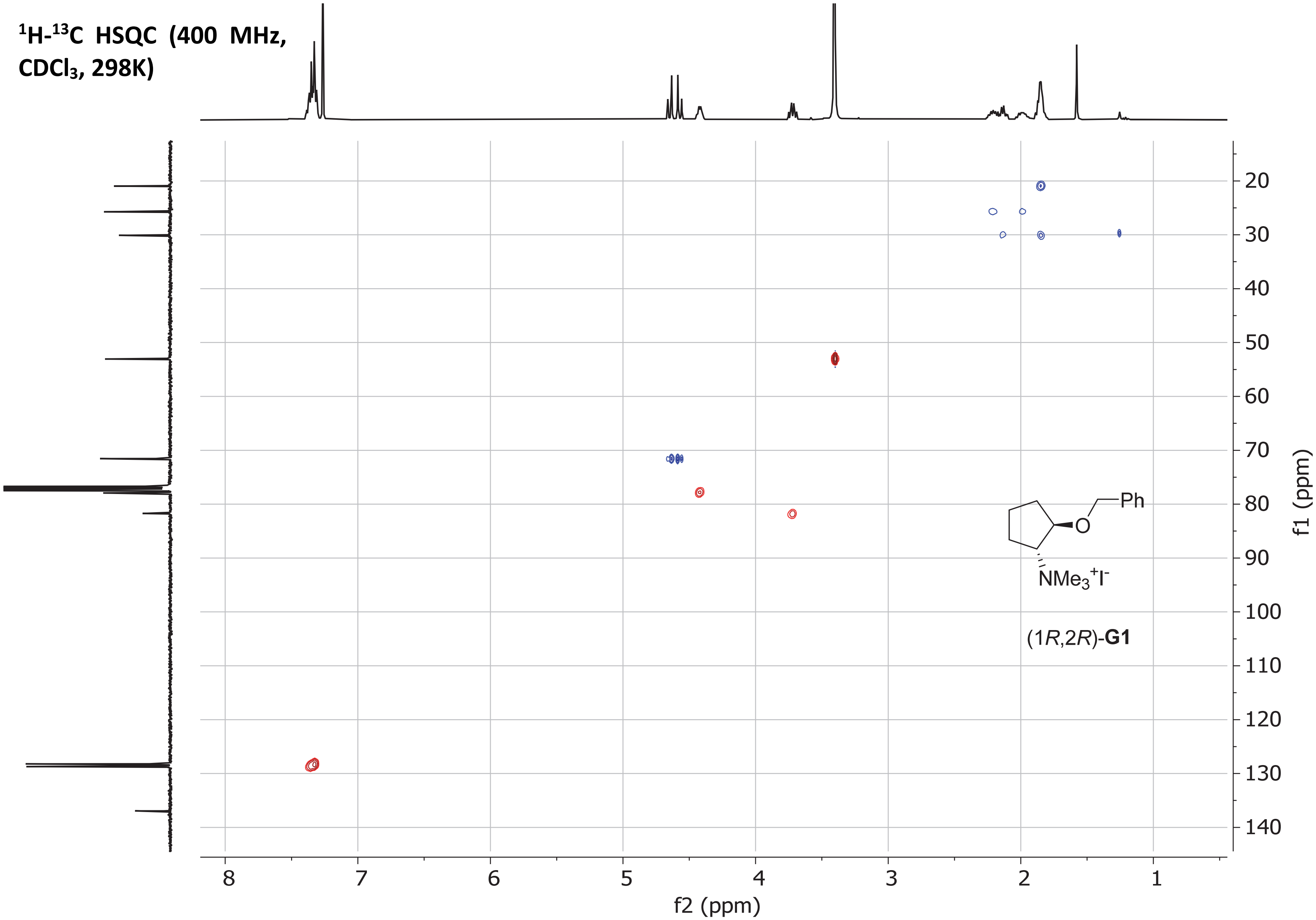

^{1}H NMR (400 MHz, $CDCl_3$, 298K)

(*R*)-**G2**

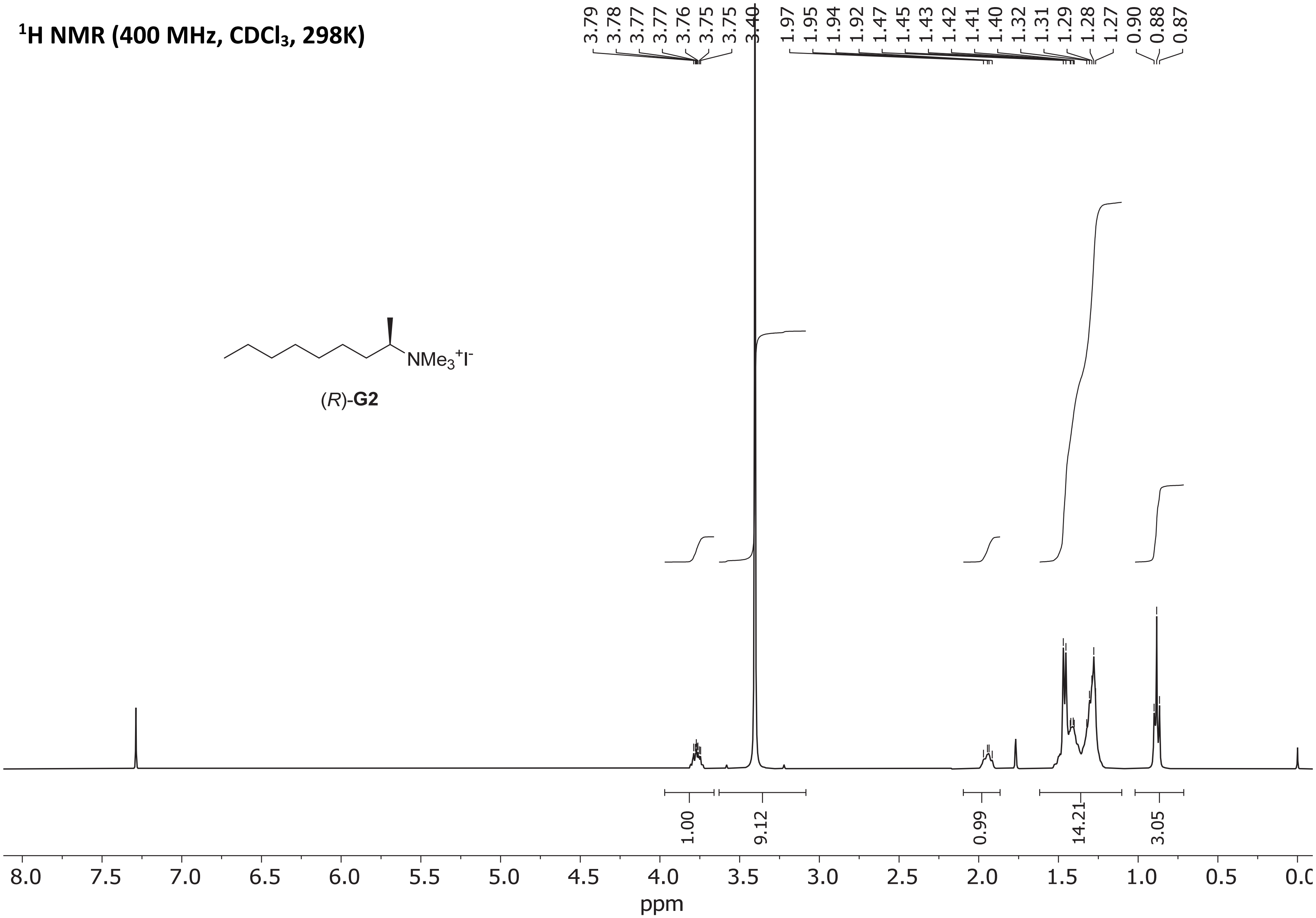

^{13}C NMR (101 MHz, $CDCl_3$, 298K)

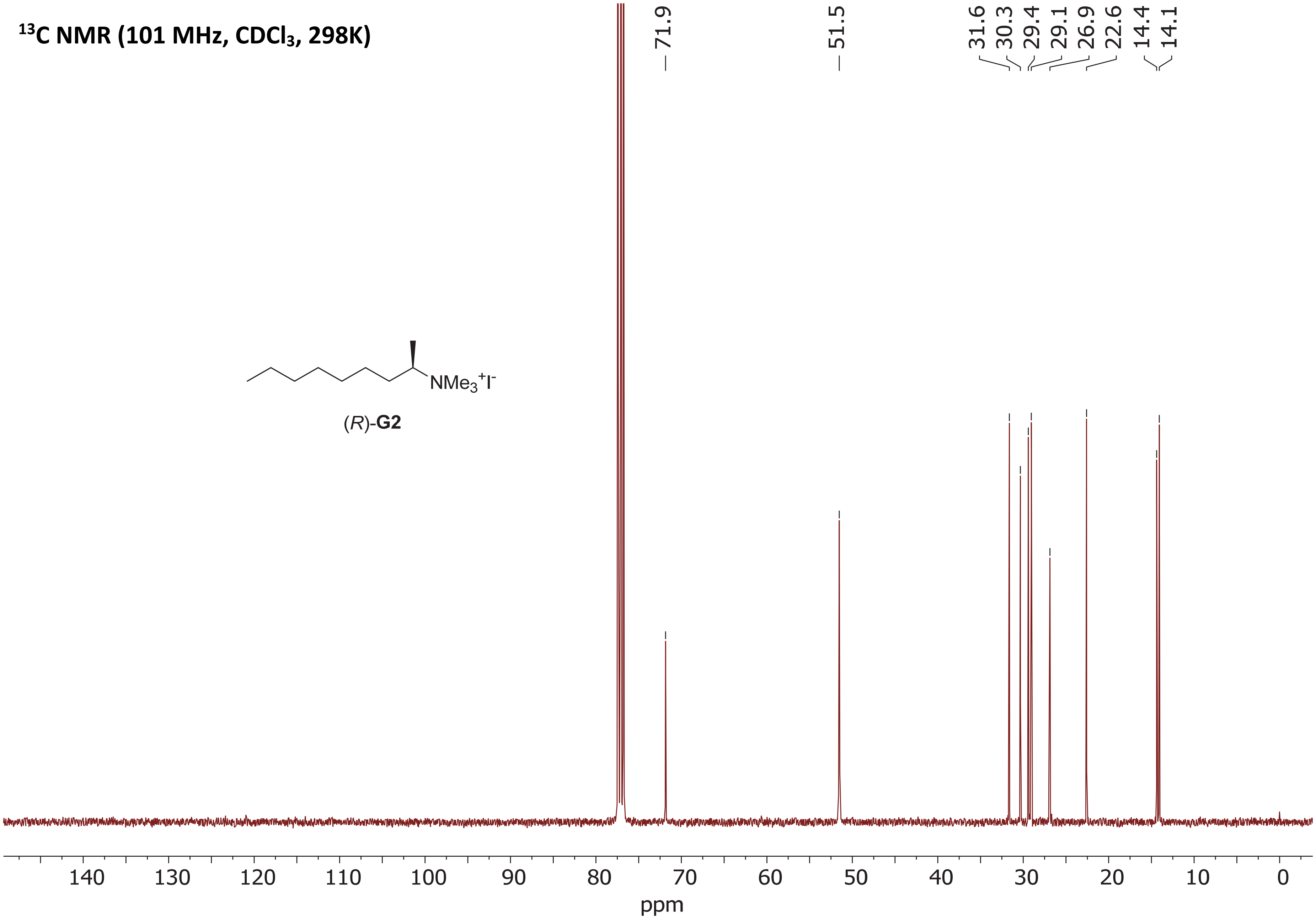

^{1}H-^{13}C HSQC (400 MHz, $CDCl_3$, 298K)

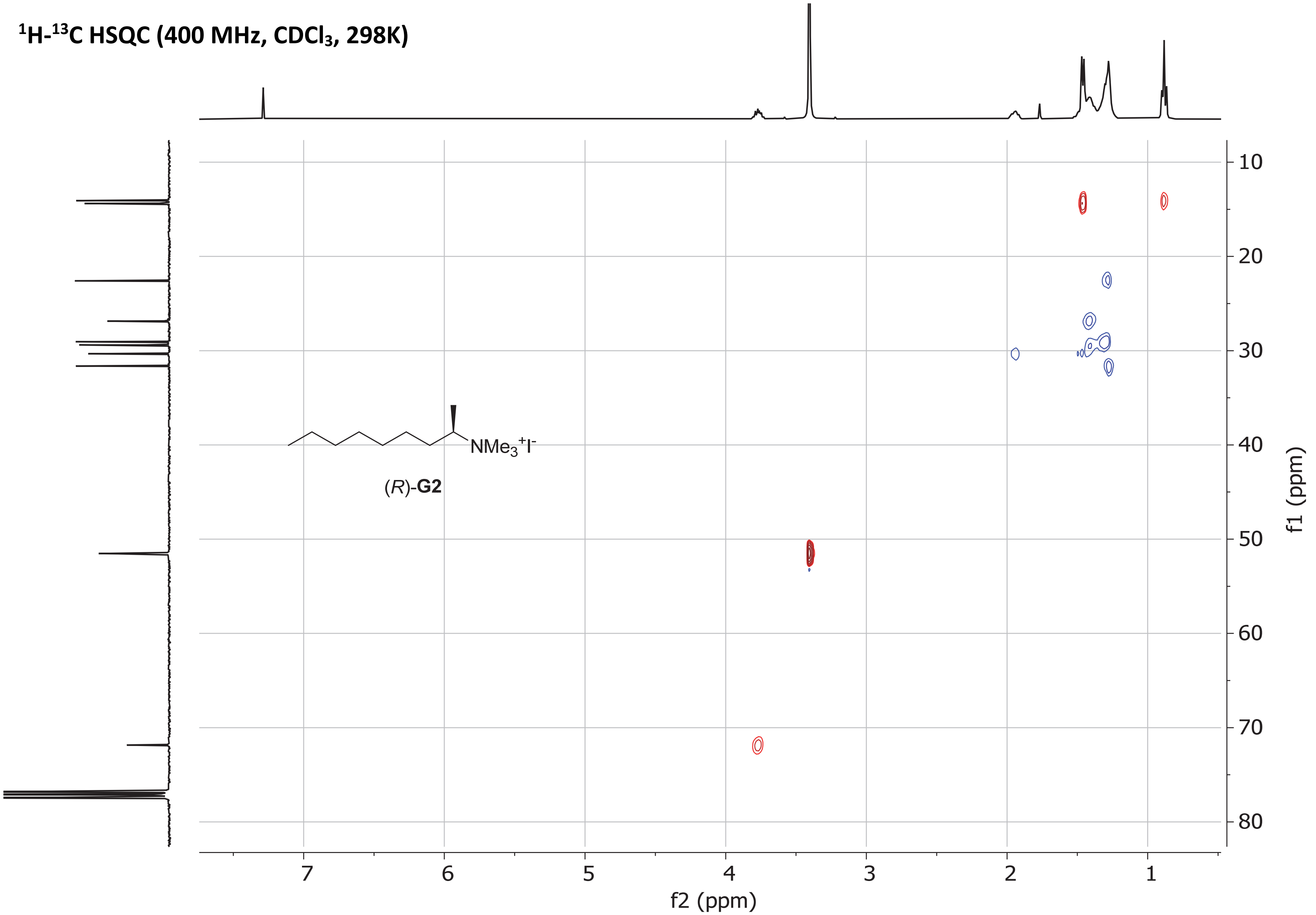

¹H NMR (400 MHz, $CDCl_3$, 298K)

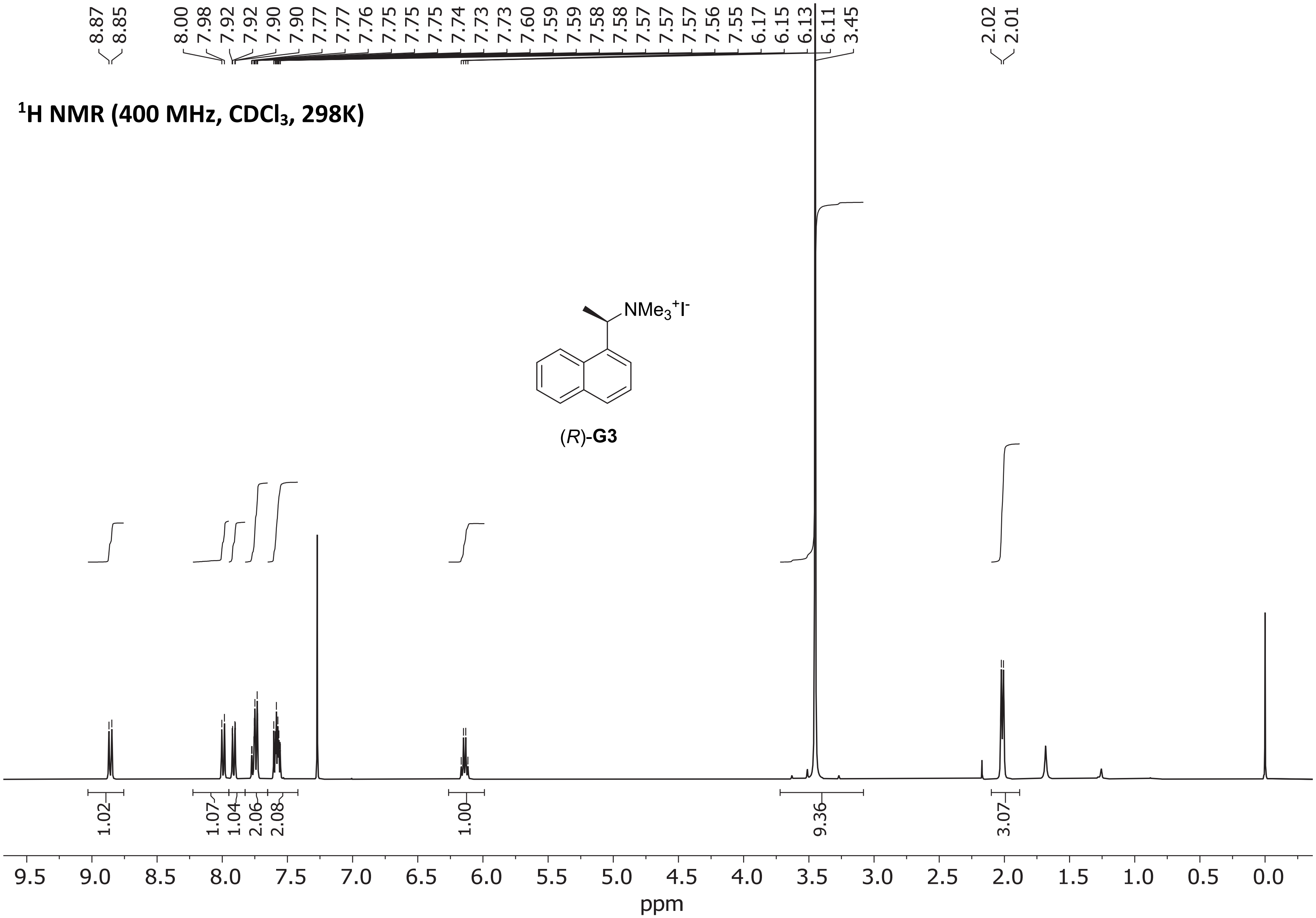

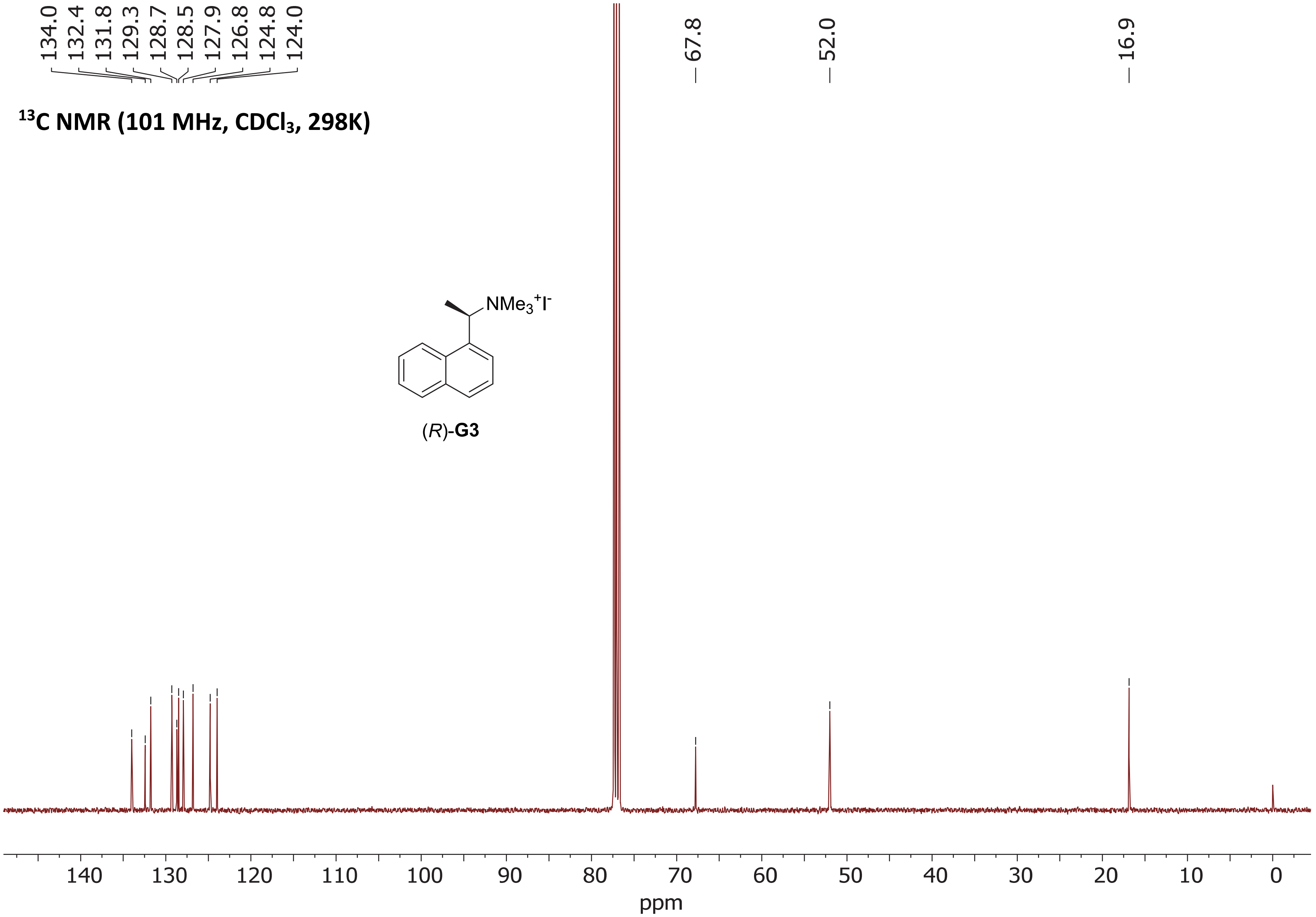

134.0
132.4
131.8
129.3
128.7
128.5
127.9
126.8
124.8
124.0
67.8
52.0
16.9
^{13}C NMR (101 MHz, CDCl$_3$, 298K)
NMe$_3$$^+I^-$
(R)-G3
140
130
120
110
100
90
80
70
60
50
40
30
20
10
0
ppm

DEPTQ (101 MHz, $CDCl_3$, 298K)

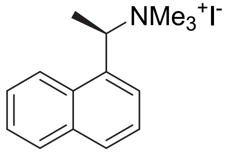

(*R*)-**G3**

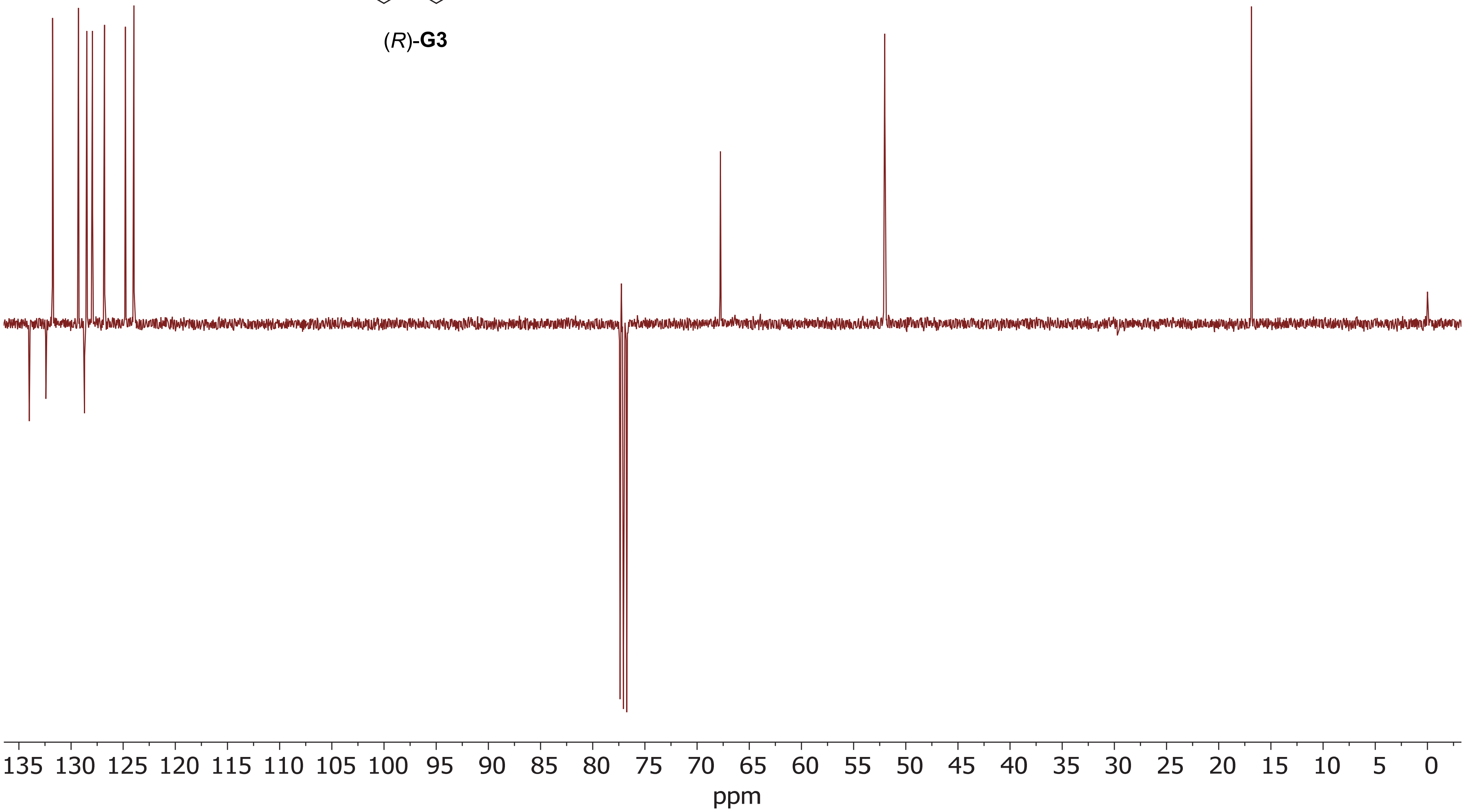

^{1}H-^{13}C HSQC (400 MHz, $CDCl_3$, 298K)

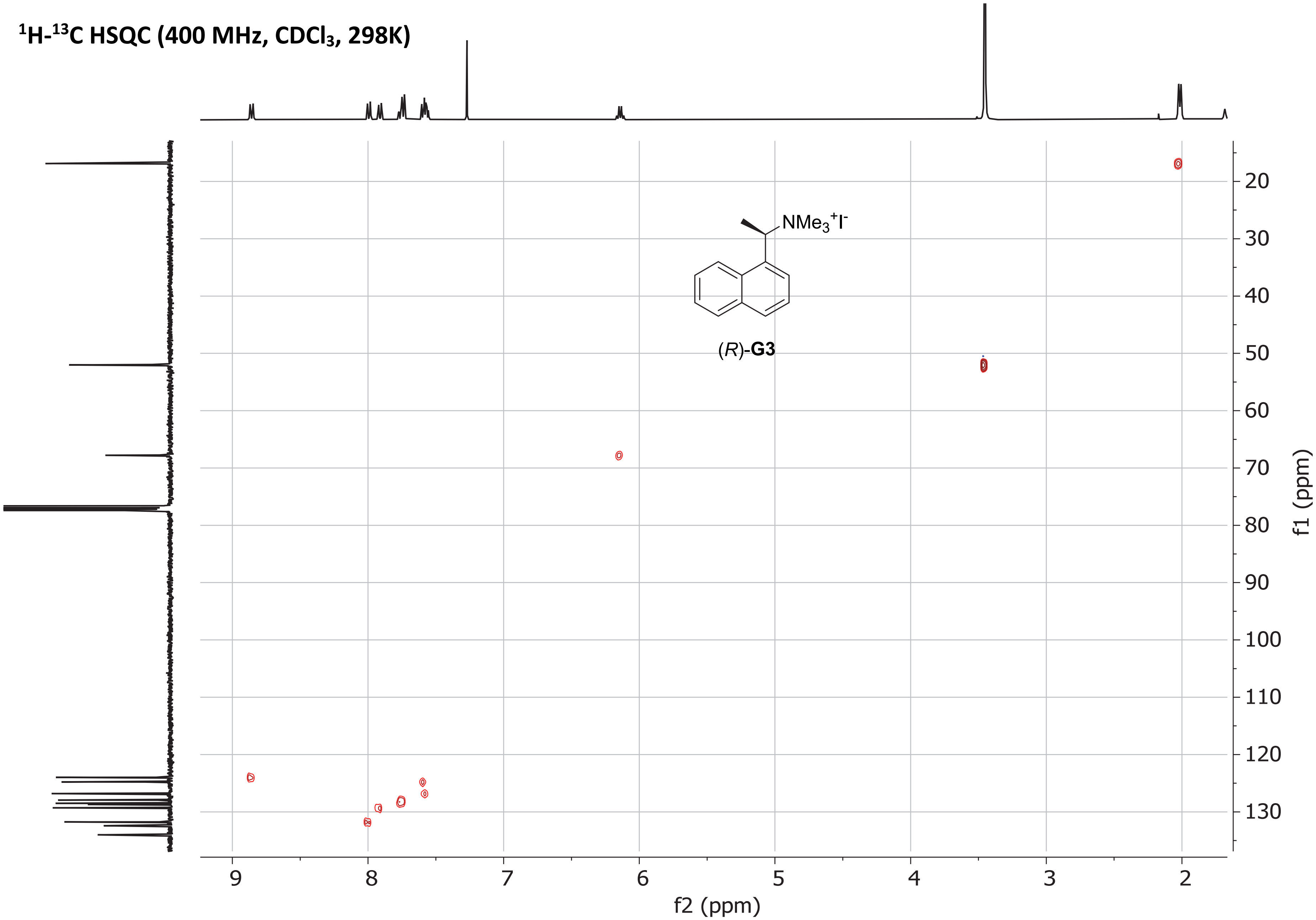

^{1}H NMR (400 MHz, $CDCl_3$, 298K)

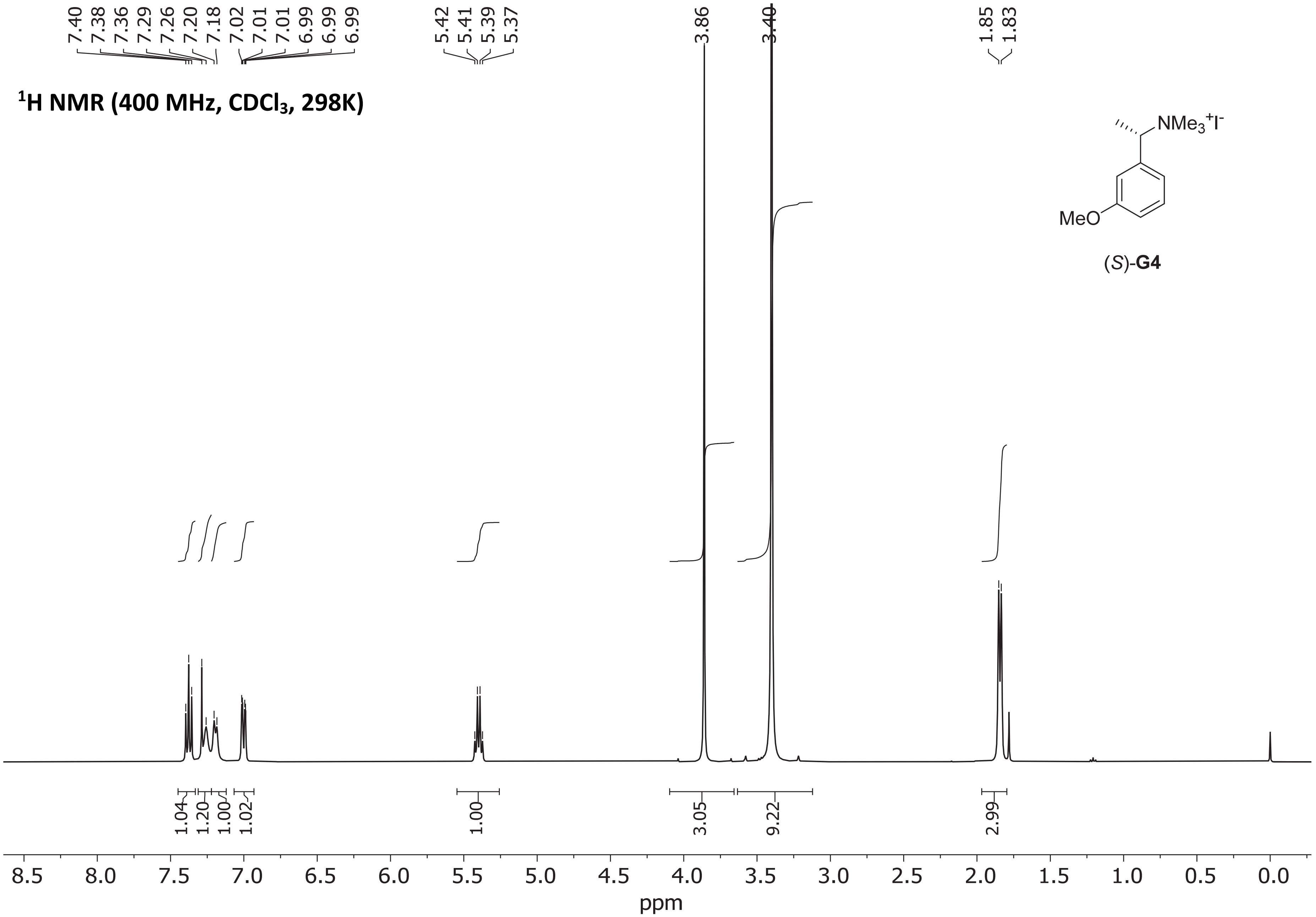

(*S*)-**G4**

^{13}C NMR (101 MHz, CDCl$_3$, 298K)

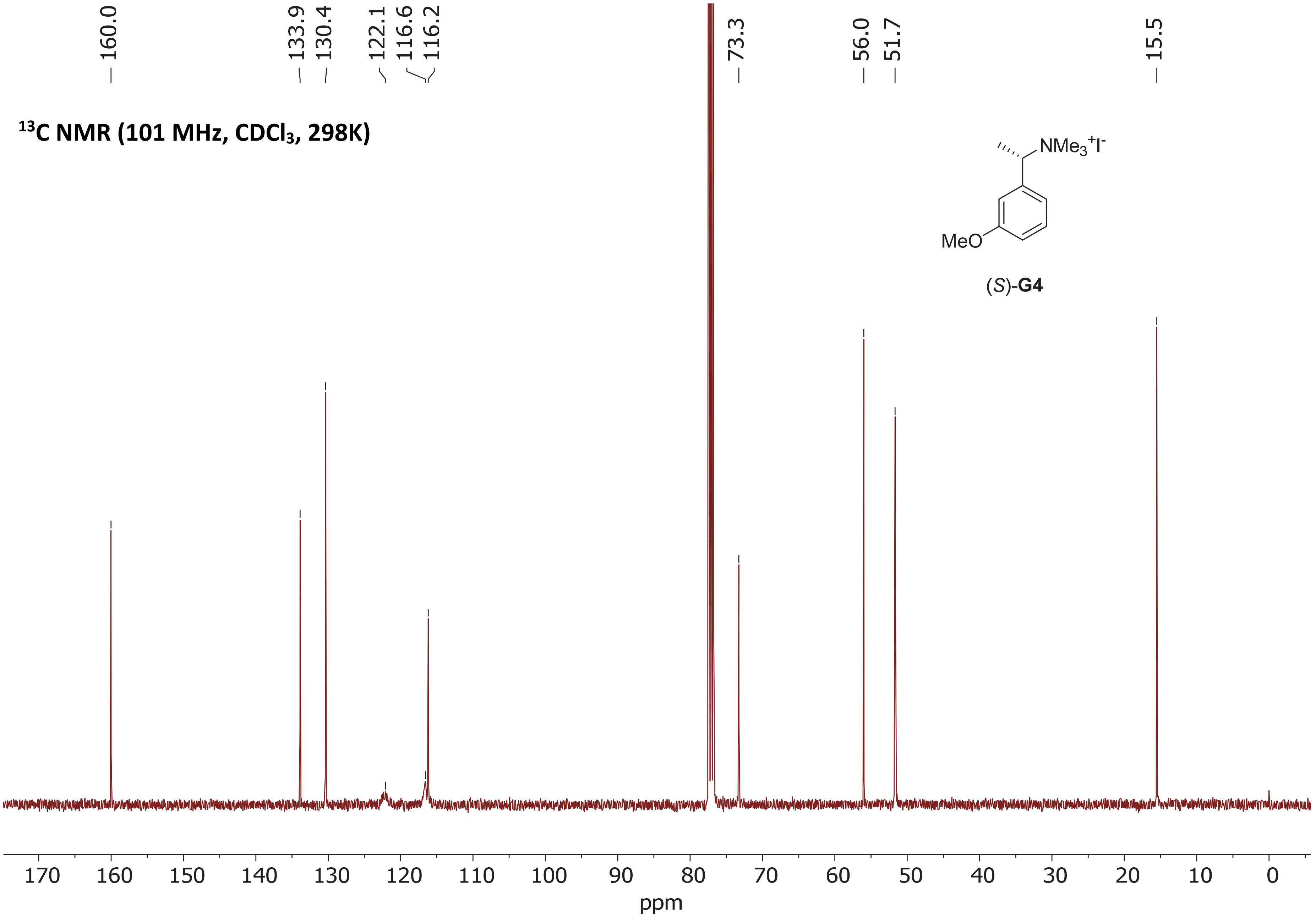

^{1}H-^{13}C HSQC (400 MHz, $CDCl_3$, 298K)

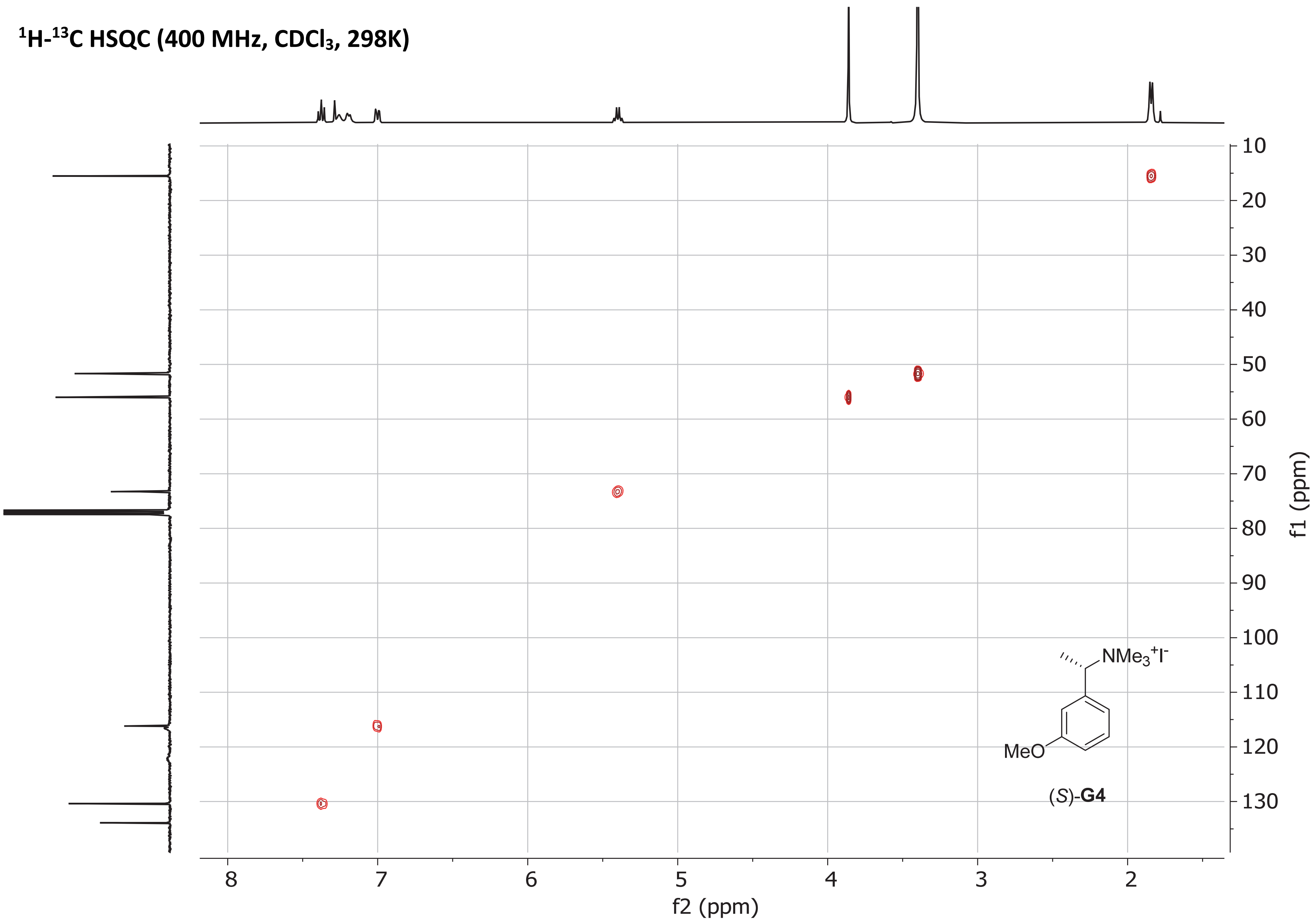

^{1}H NMR (400 MHz, CDCl$_3$, 298K)

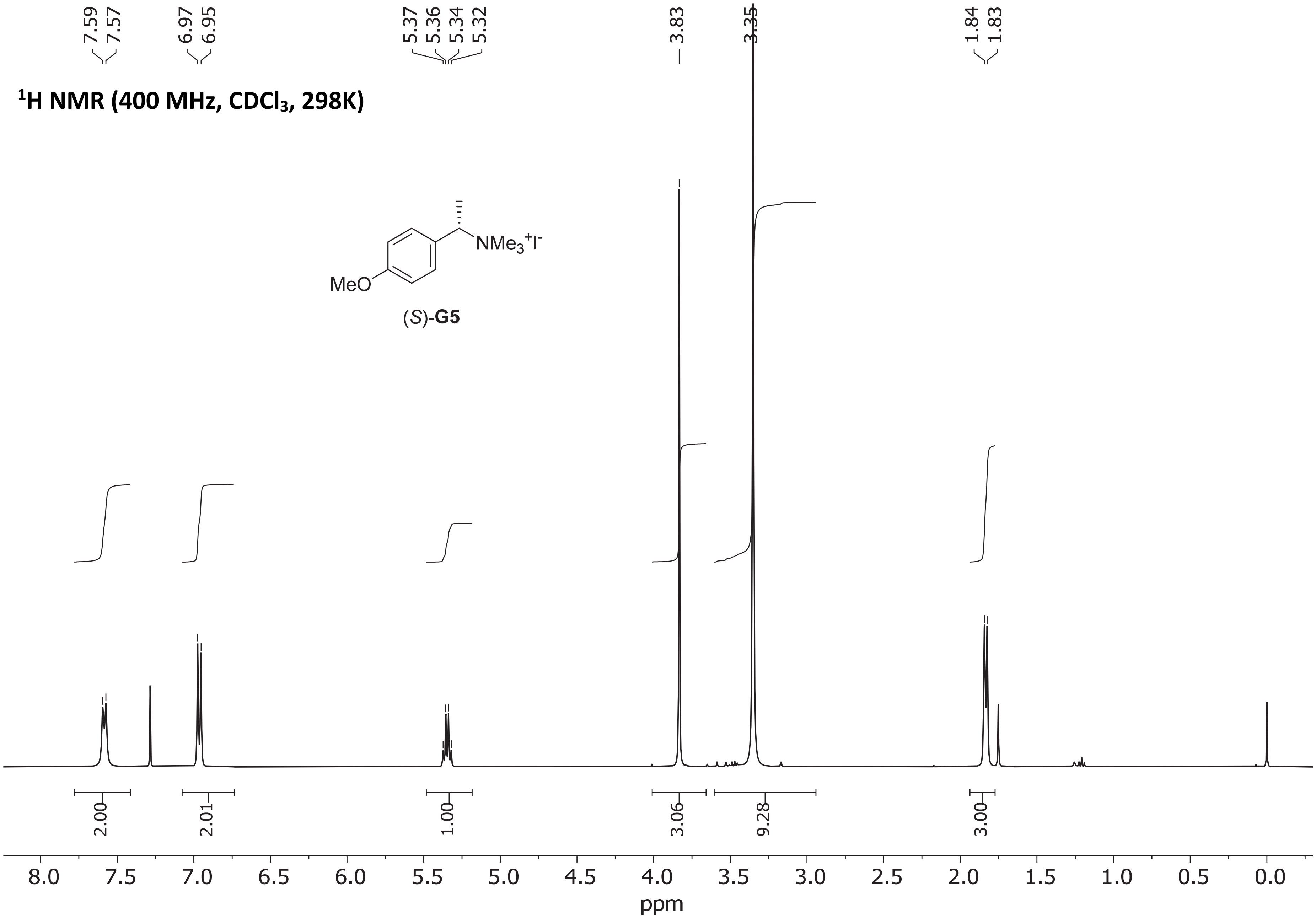

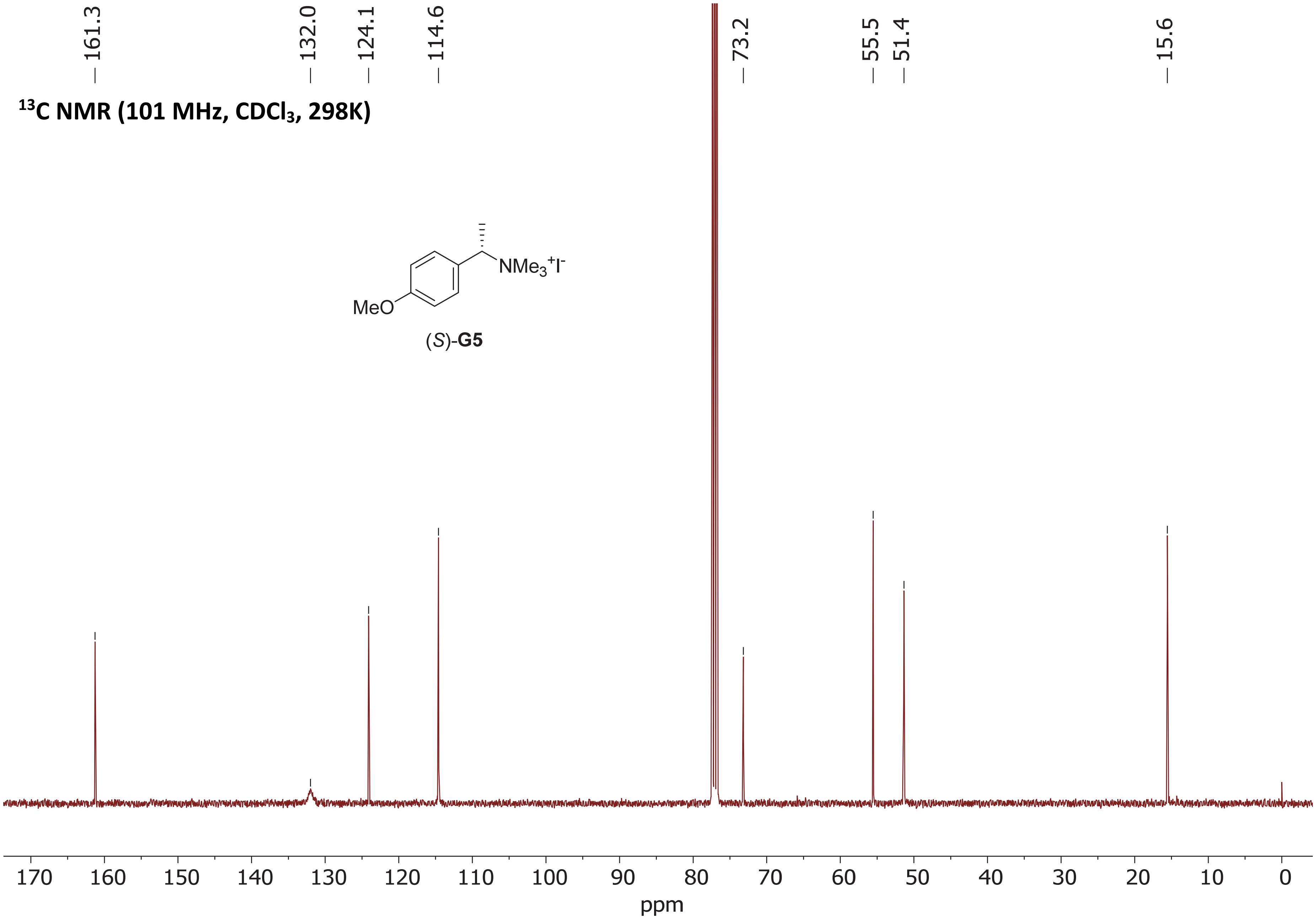

161.3
132.0
124.1
114.6
73.2
55.5
51.4
15.6
^{13}C NMR (101 MHz, $CDCl_3$, 298K)
MeO
$NMe_3^+I^-$
(*S*)-**G5**
170 160 150 140 130 120 110 100 90 80 70 60 50 40 30 20 10 0
ppm

^{1}H-^{13}C HSQC (400 MHz, $CDCl_3$, 298K)

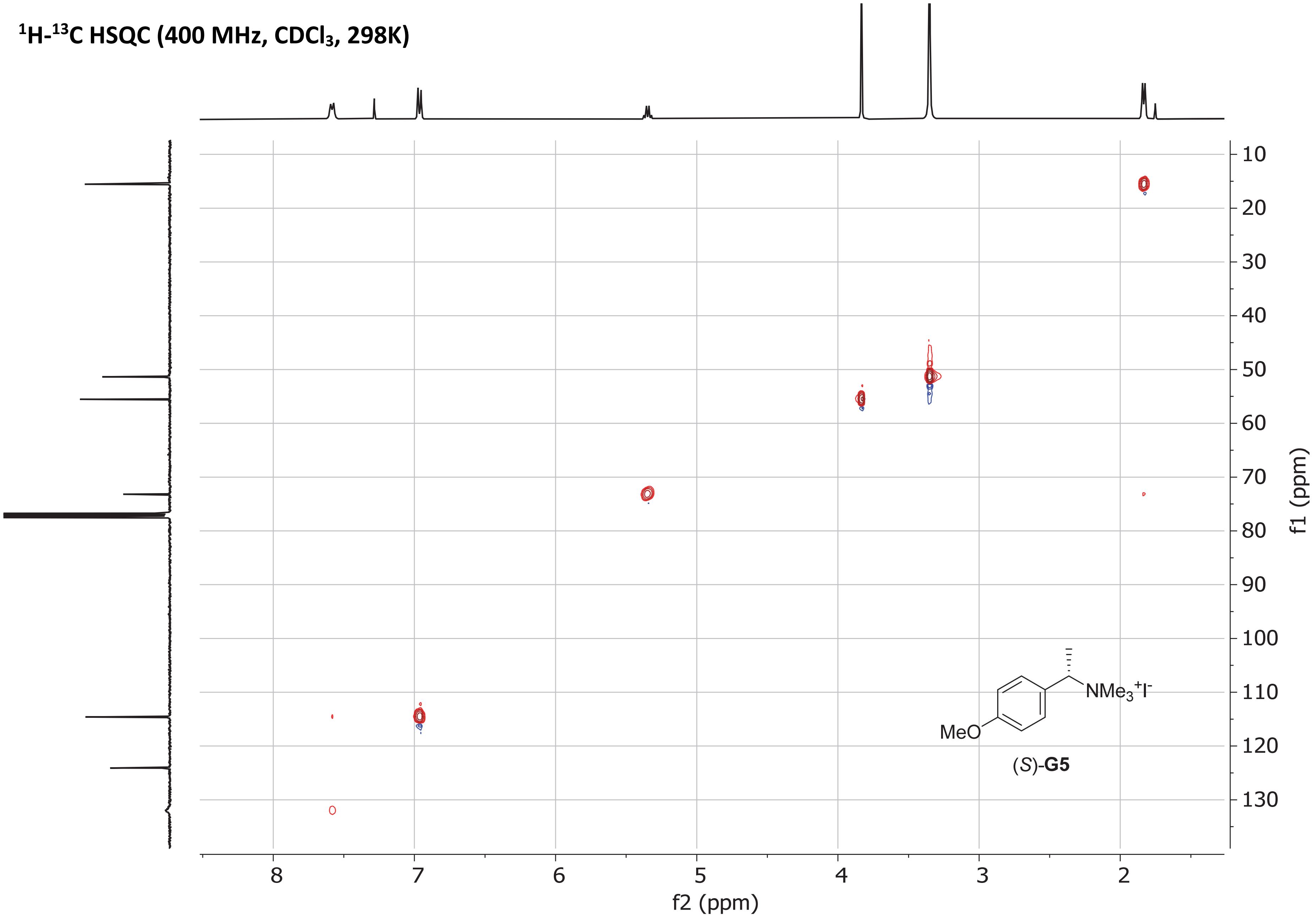

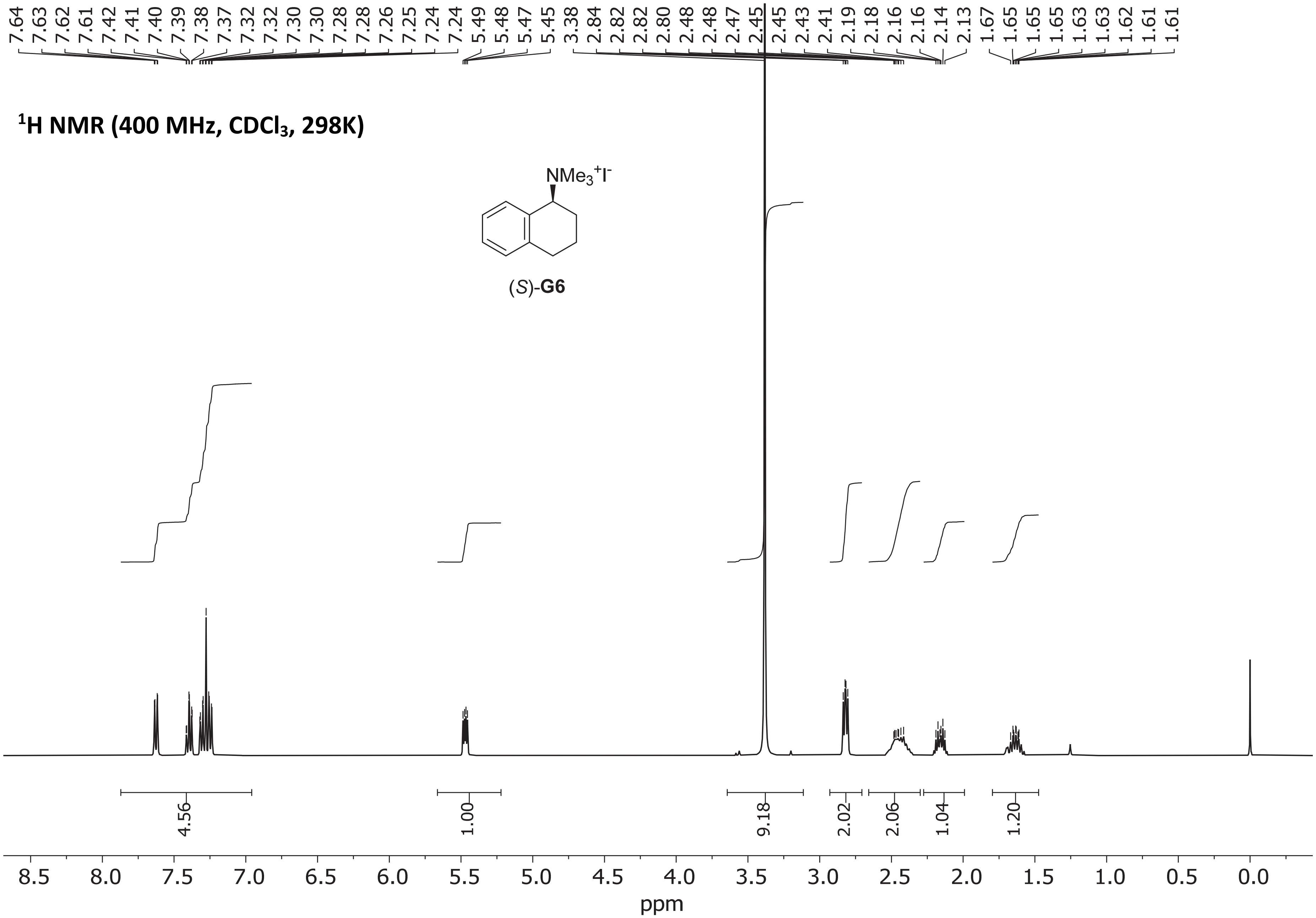
7.64
7.63
7.62
7.61
7.42
7.41
7.40
7.39
7.38
7.37
7.32
7.32
7.30
7.30
7.28
7.28
7.26
7.25
7.24
7.24
5.49
5.48
5.47
5.45
3.38
2.84
2.82
2.82
2.80
2.48
2.48
2.47
2.45
2.45
2.43
2.41
2.19
2.18
2.16
2.16
2.14
2.13
1.67
1.65
1.65
1.65
1.63
1.63
1.62
1.61
1.61
¹H NMR (400 MHz, CDCl₃, 298K)
NMe₃⁺I⁻
(S)-G6
4.56
1.00
9.18
2.02
2.06
1.04
1.20
8.5
8.0
7.5
7.0
6.5
6.0
5.5
5.0
4.5
4.0
3.5
3.0
2.5
2.0
1.5
1.0
0.5
0.0
ppm

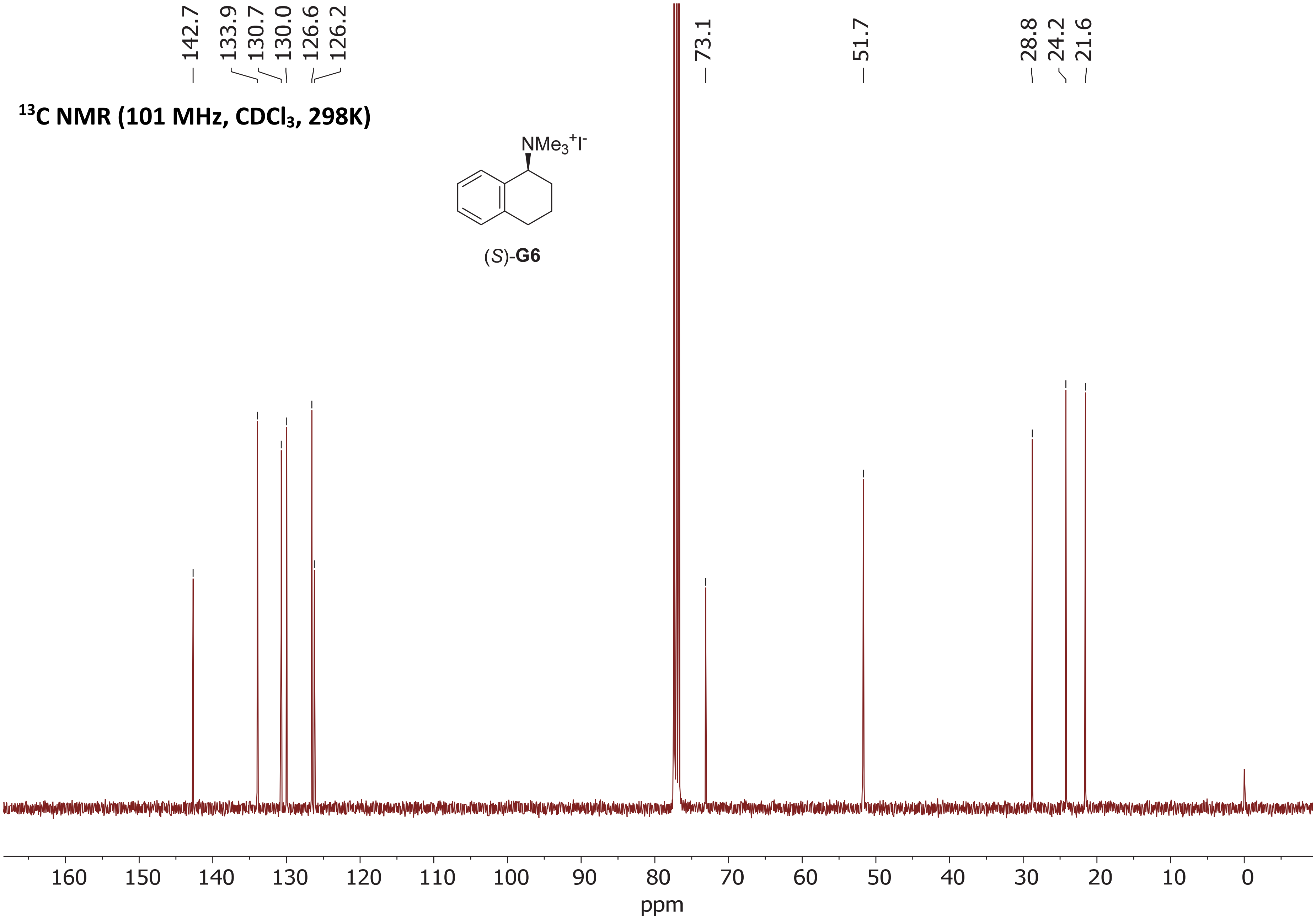

142.7
133.9
130.7
130.0
126.6
126.2
73.1
51.7
28.8
24.2
21.6
¹³C NMR (101 MHz, CDCl₃, 298K)
NMe₃⁺I⁻
(S)-G6
160
150
140
130
120
110
100
90
80
70
60
50
40
30
20
10
0
ppm

^{1}H-^{13}C HSQC (400 MHz, $CDCl_3$, 298K)

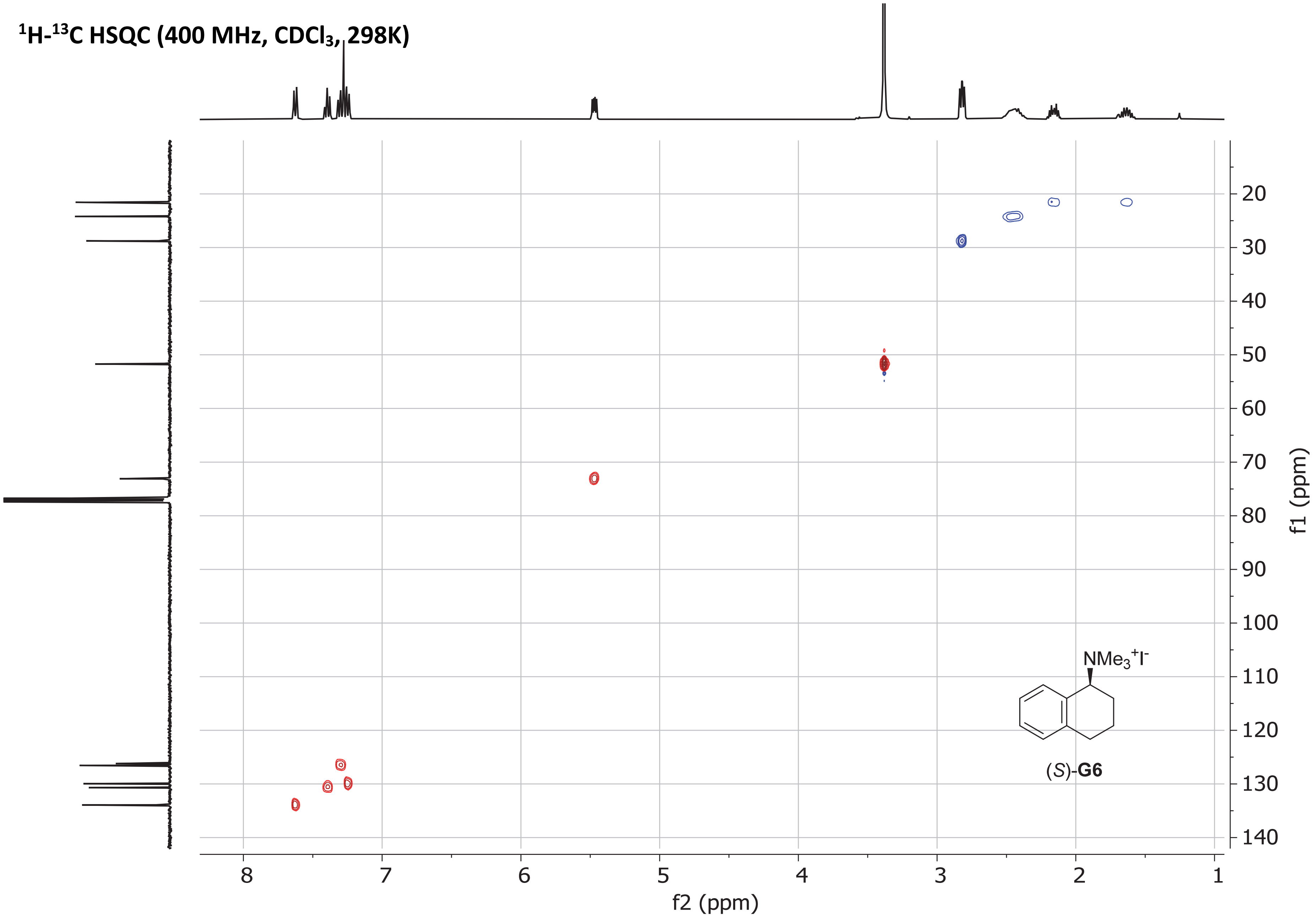

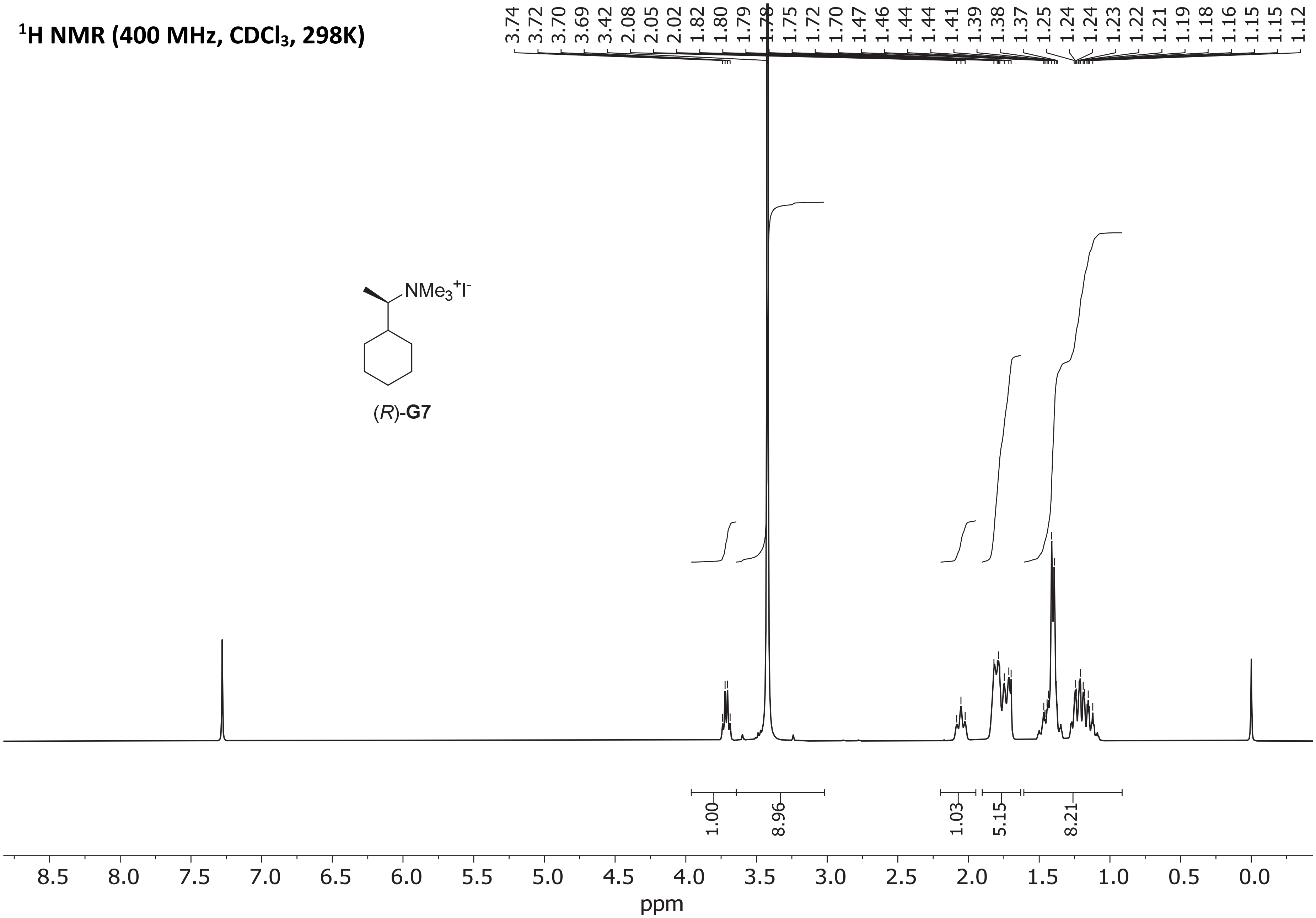
^{1}H NMR (400 MHz, $CDCl_3$, 298K)
3.74 3.72 3.70 3.69 3.42 2.08 2.05 2.02 1.82 1.80 1.79 1.78 1.75 1.72 1.70 1.47 1.46 1.44 1.44 1.41 1.39 1.38 1.37 1.25 1.24 1.24 1.23 1.22 1.21 1.19 1.18 1.16 1.15 1.15 1.12
NMe3+I-
(R)-G7
1.00
8.96
1.03
5.15
8.21
8.5 8.0 7.5 7.0 6.5 6.0 5.5 5.0 4.5 4.0 3.5 3.0 2.5 2.0 1.5 1.0 0.5 0.0
ppm

^{13}C NMR (101 MHz, CDCl$_3$, 298K)

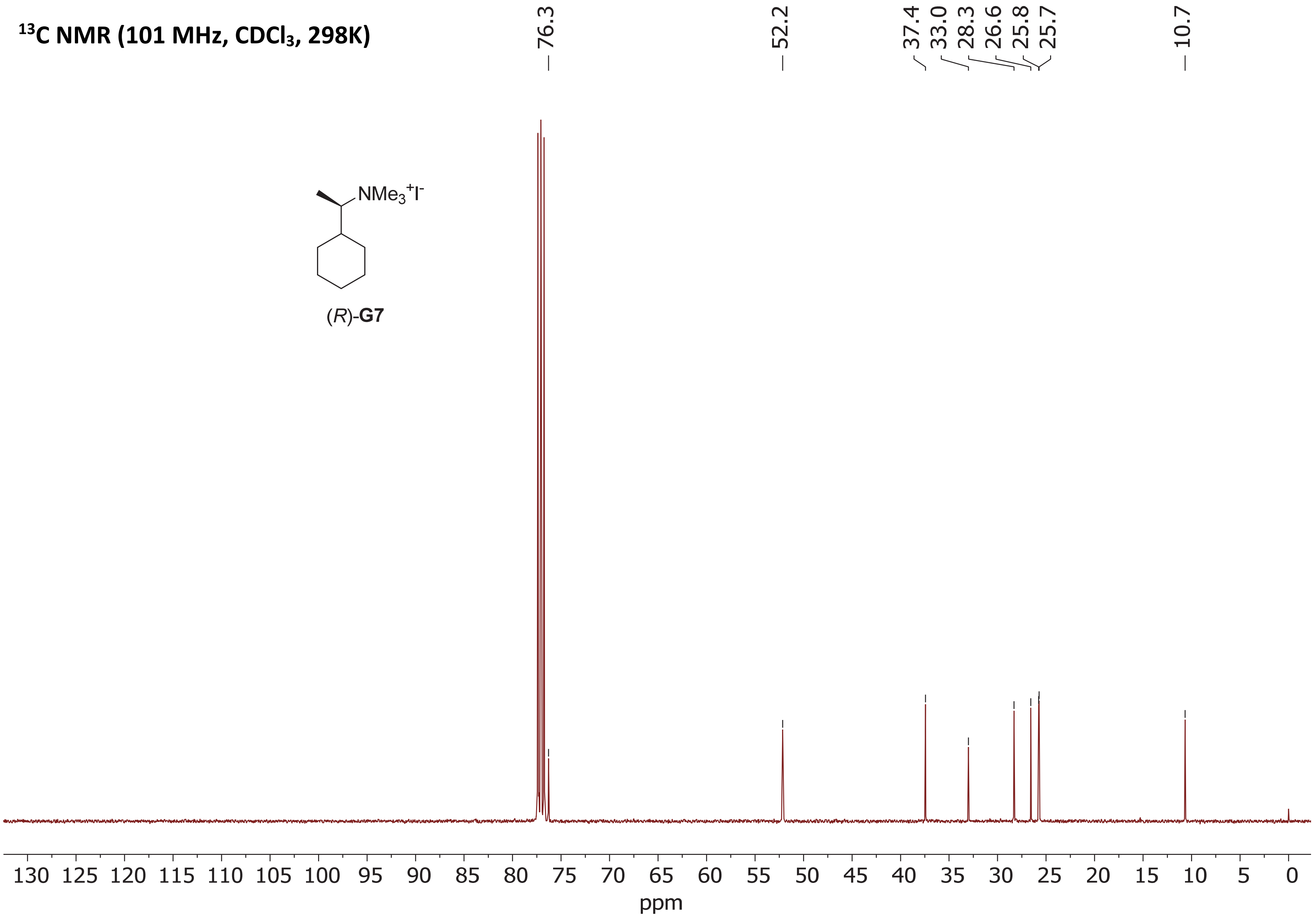

^{1}H-^{13}C HSQC (400 MHz, $CDCl_3$, 298K)

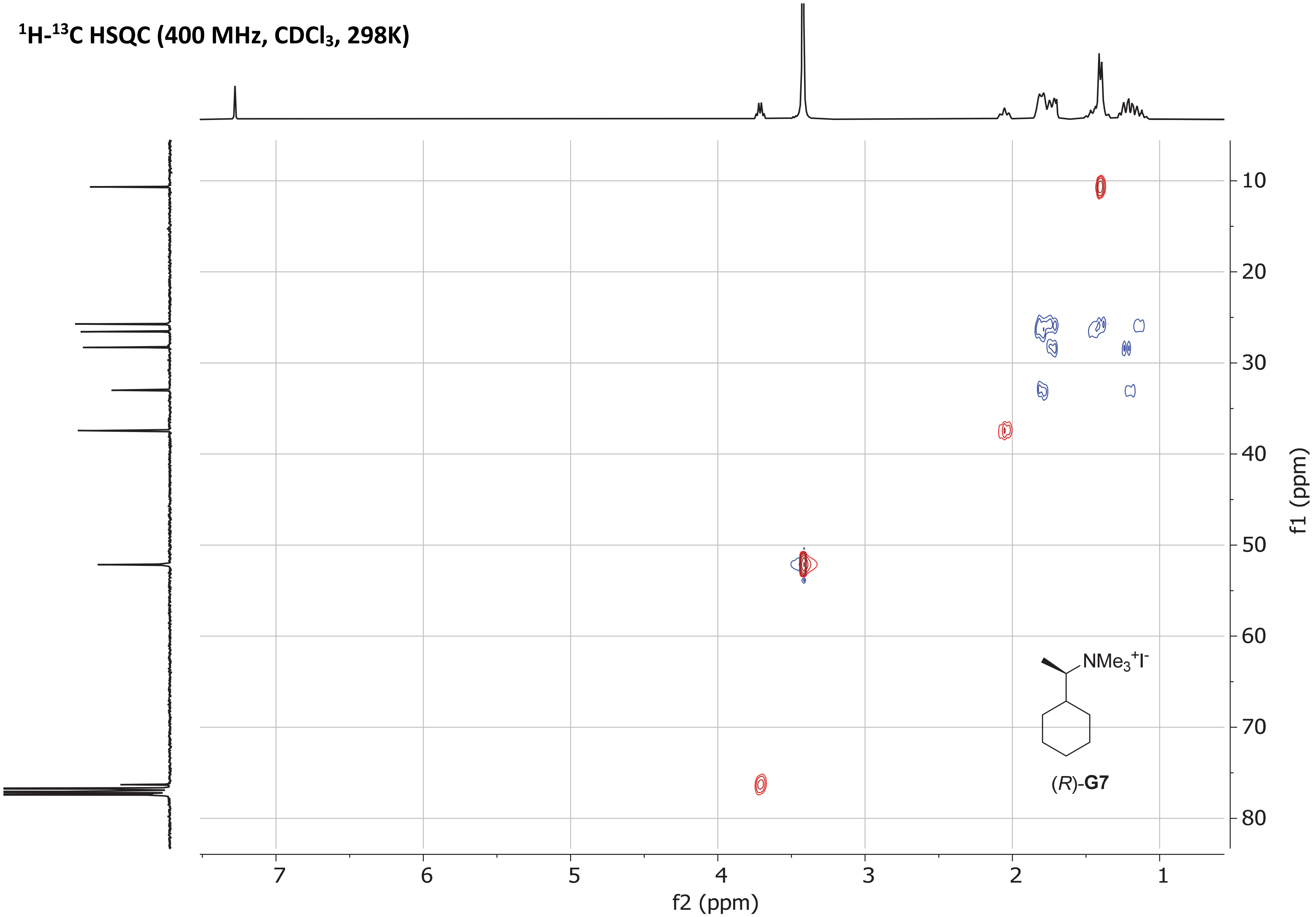

^{1}H NMR (400 MHz, $CDCl_3$, 298K)

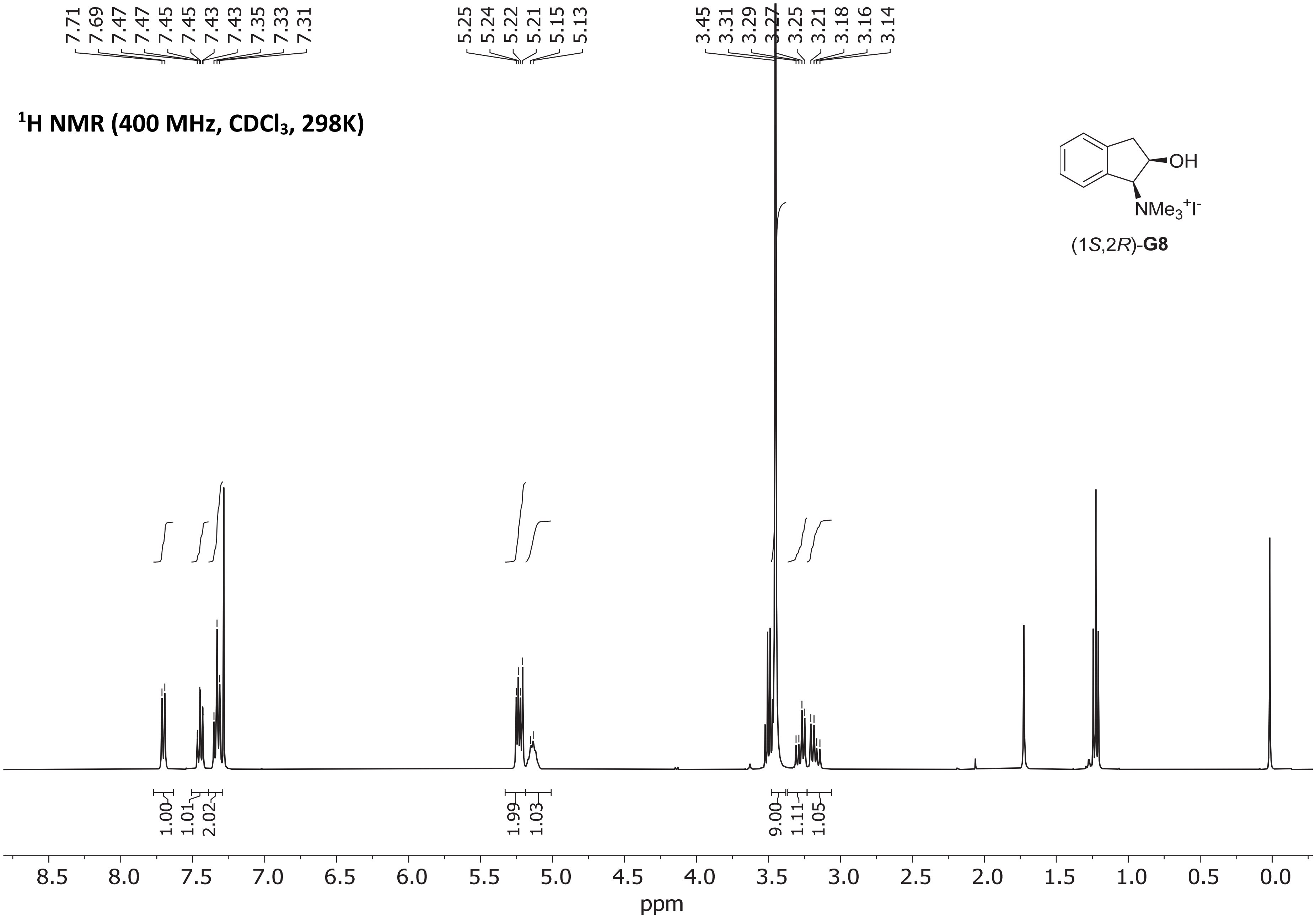

(1*S*,2*R*)-**G8**

^{1}H NMR (400 MHz, DMSO-d_6, 298K)

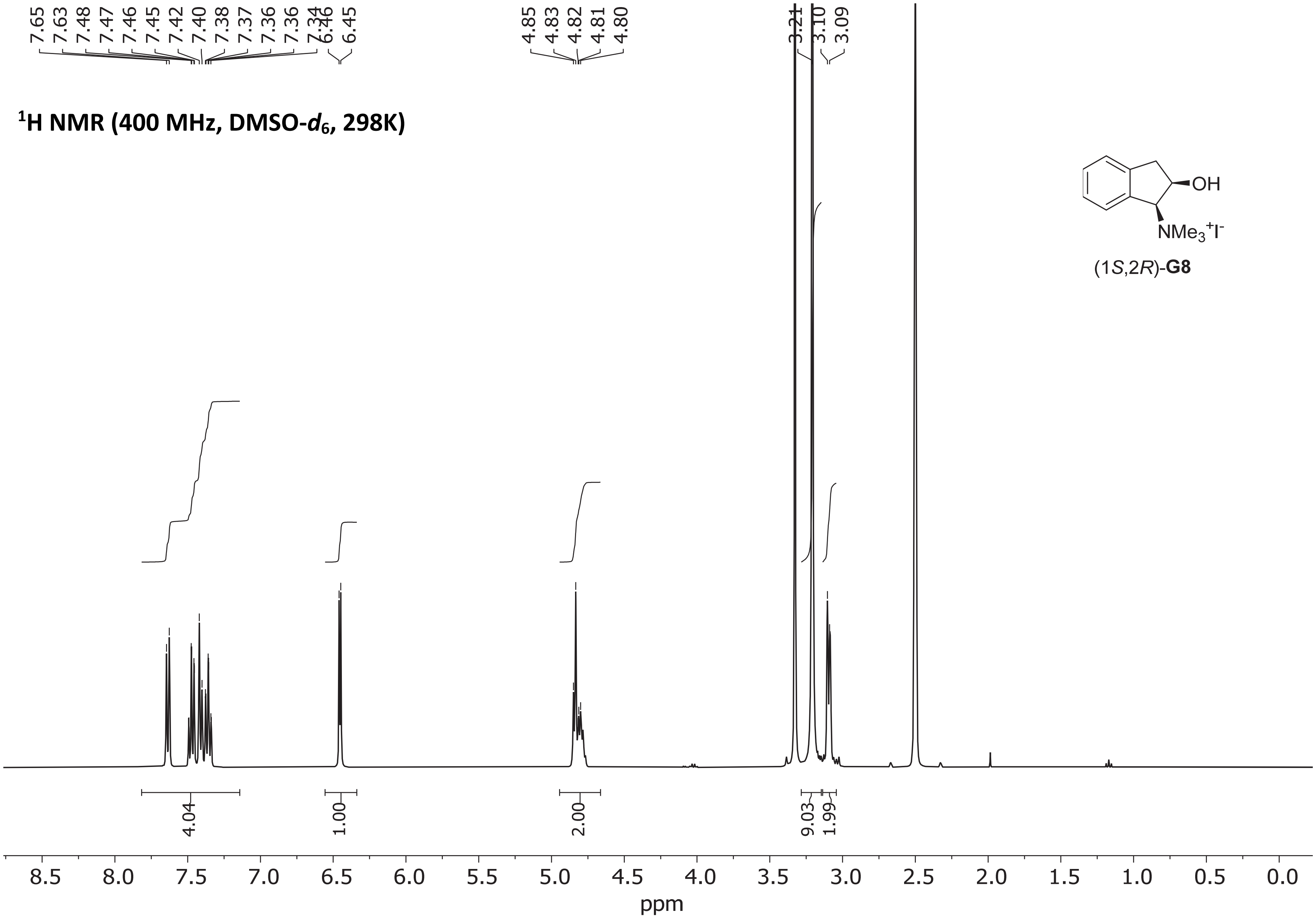

(1*S*,2*R*)-**G8**

^{13}C NMR (101 MHz, DMSO-d_6, 298K)

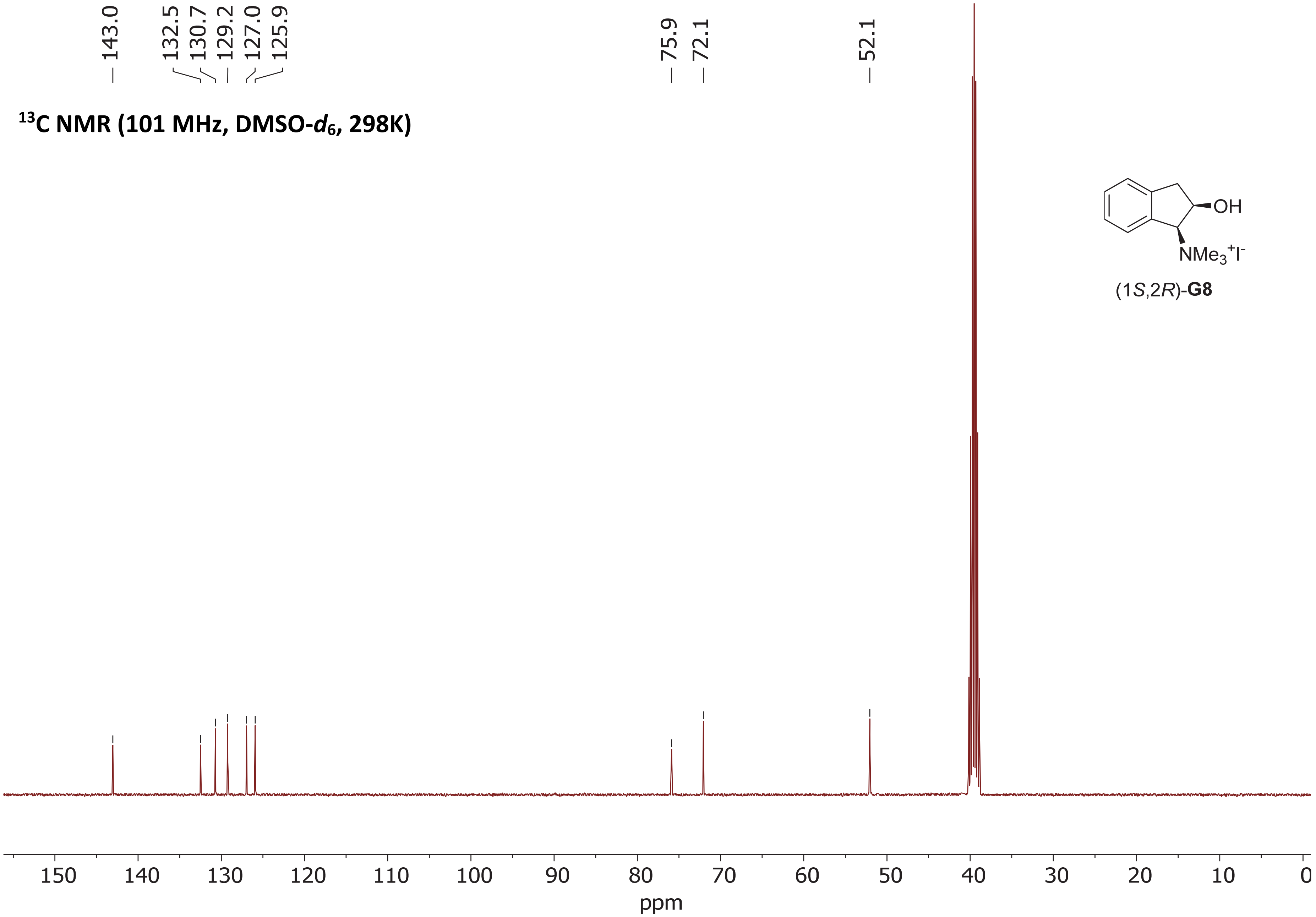

(1*S*,2*R*)-**G8**

^1H-^{13}C HSQC (400 MHz, DMSO-d_6, 298K)

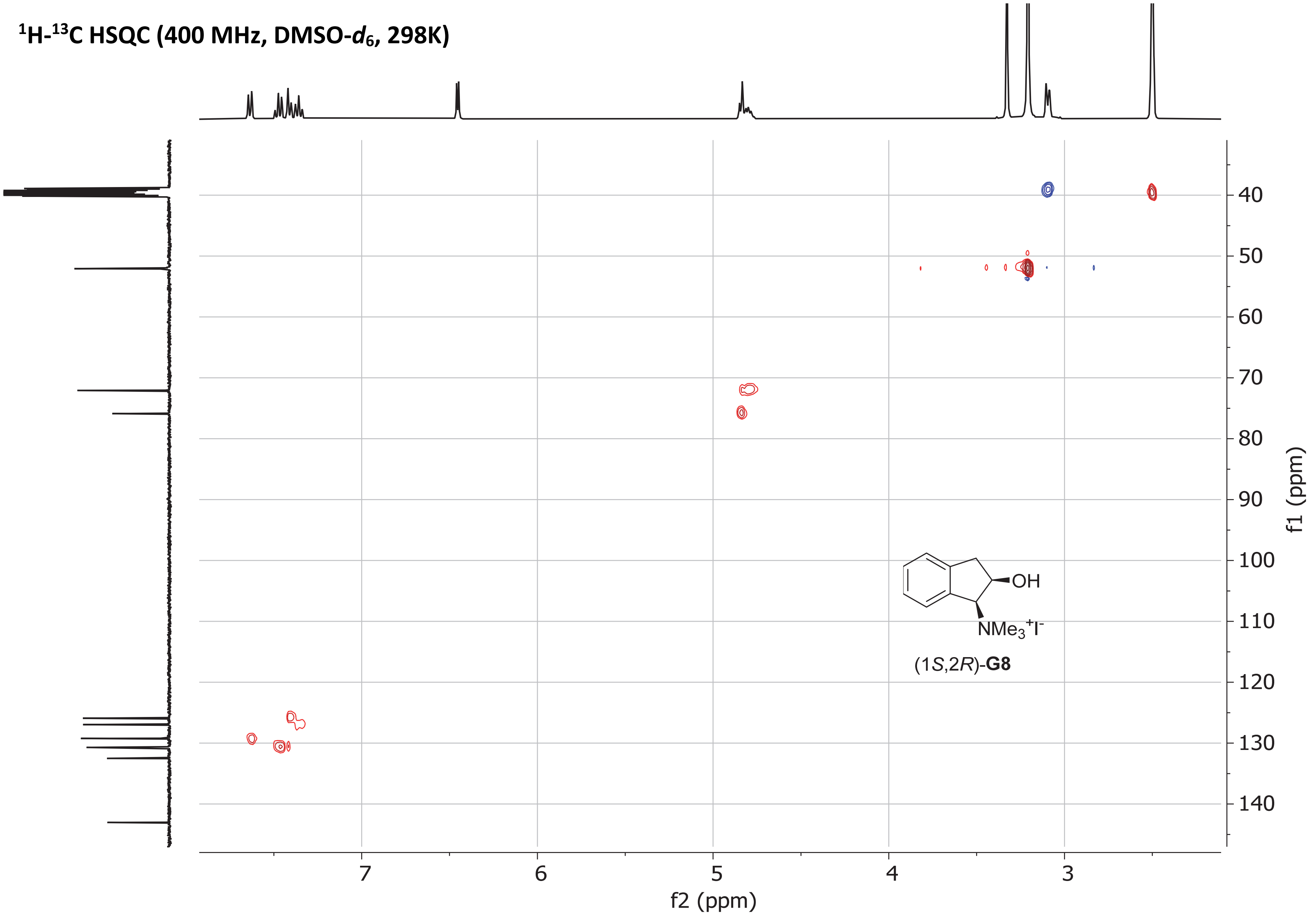

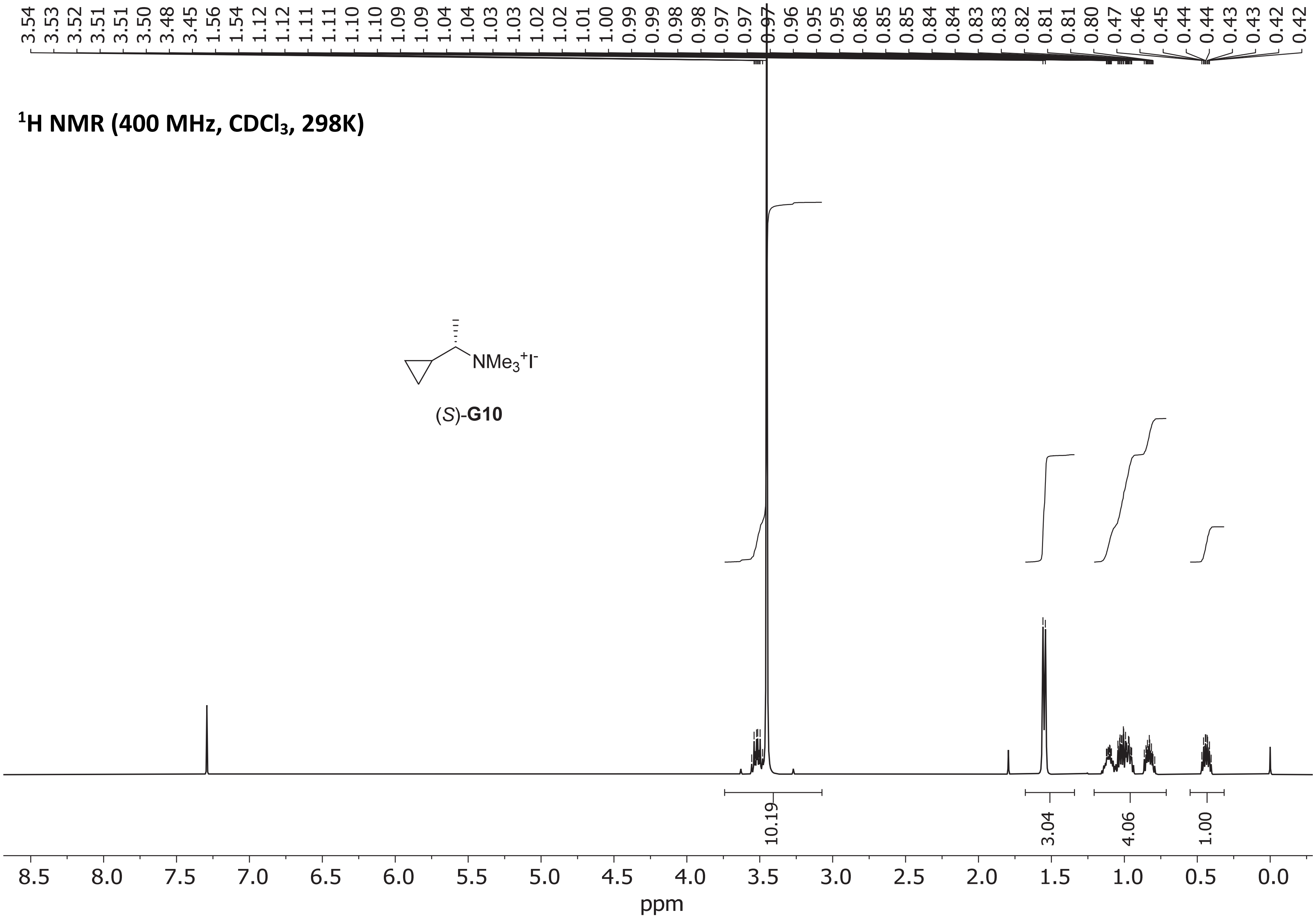

S99

^{13}C NMR (400 MHz, $CDCl_3$, 298K)

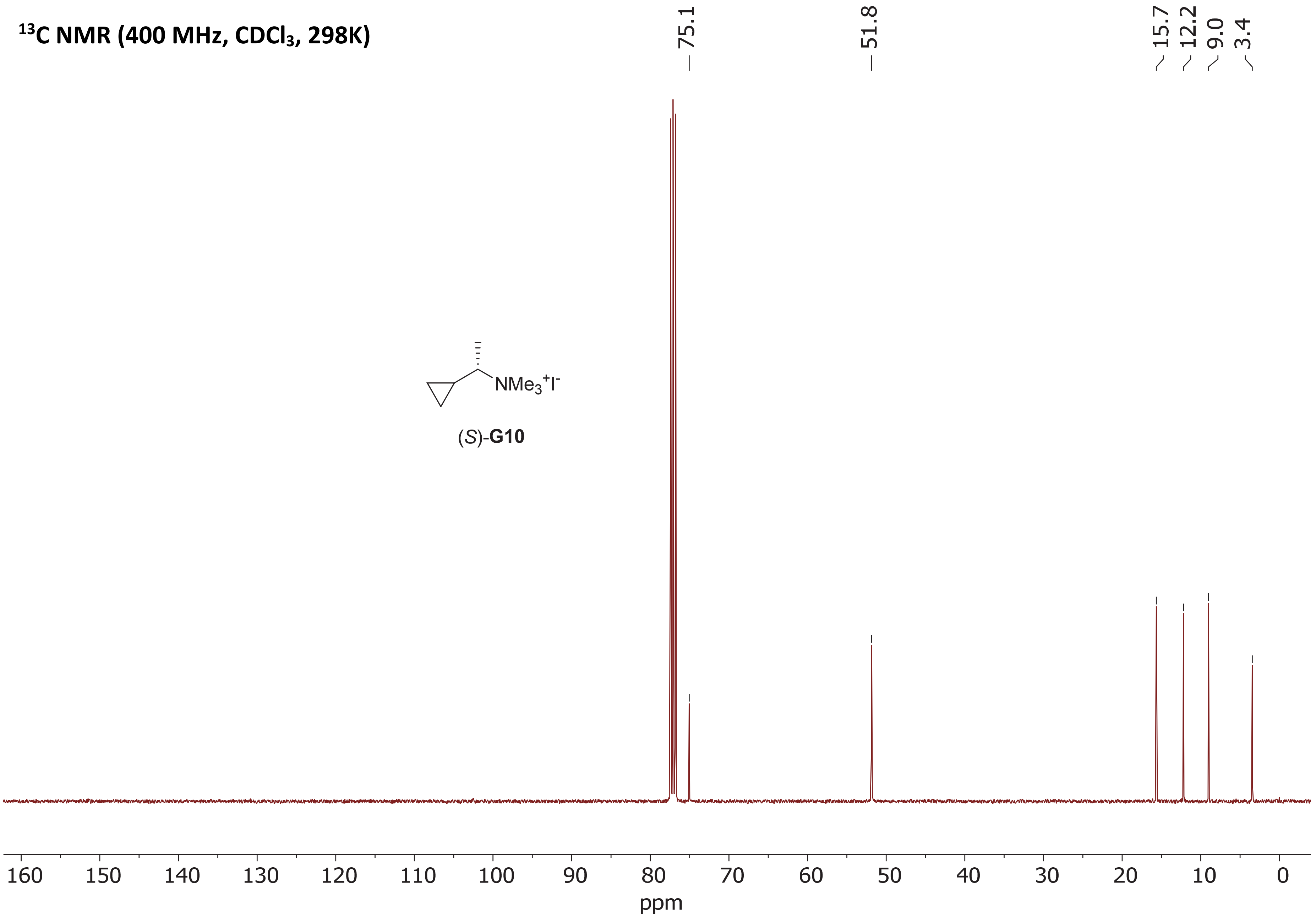

^1H-^{13}C HSQC (400 MHz, $CDCl_3$, 298K)

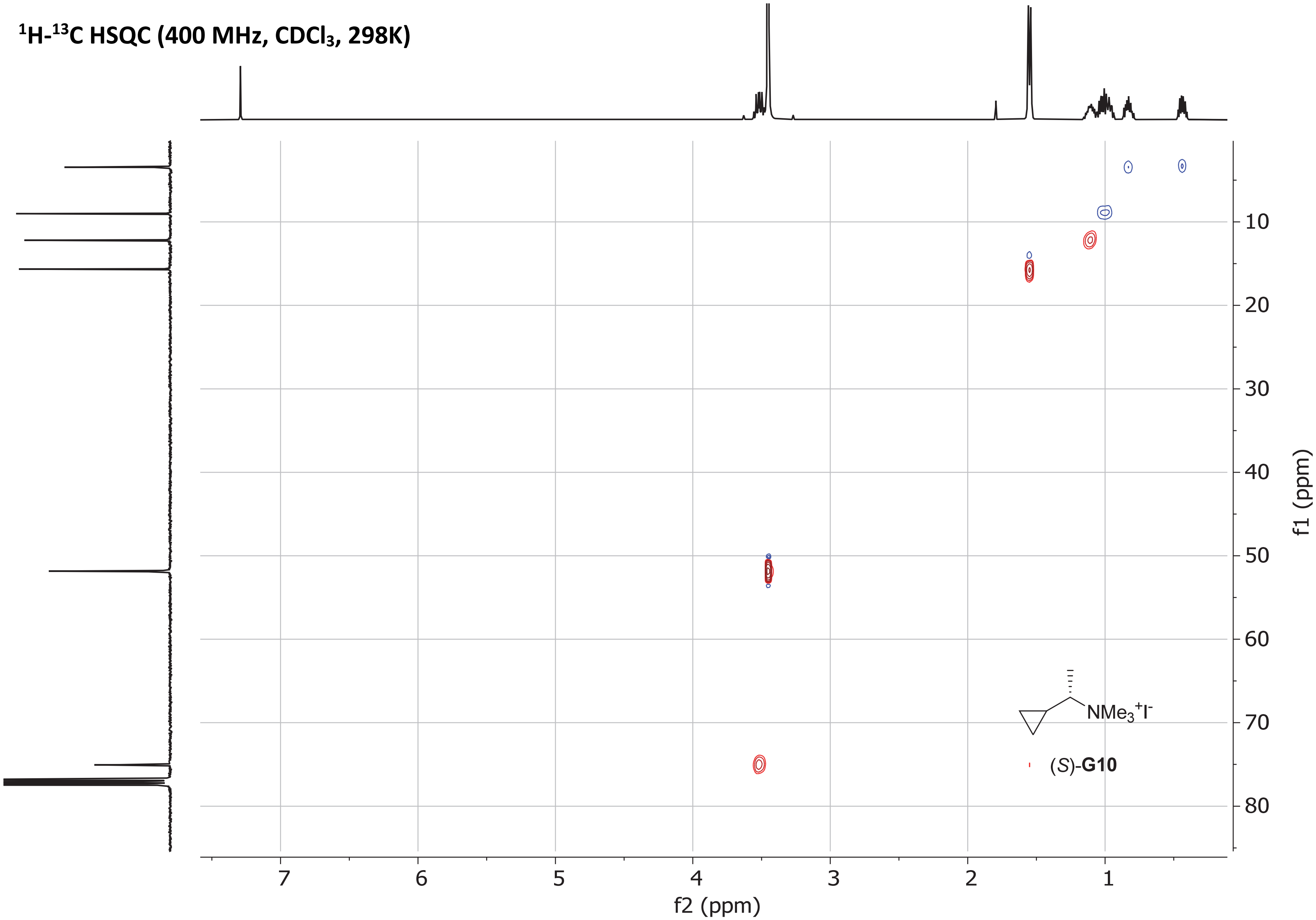

9 HRMS spectra of new compounds

ESI(-) HRMS

Generic Display Report

Analysis Info

Analysis Name	X:\QTOF files\Àngel Oliveras\Agustí Lledo\20220322\2022032200001.d	Acquisition Date	22/3/2022 10:03:20
Method	tune_high_negative.m	Operator	Univ. Girona
Sample Name	ALP1632	Instrument	micrOTOF-Q II
Comment			

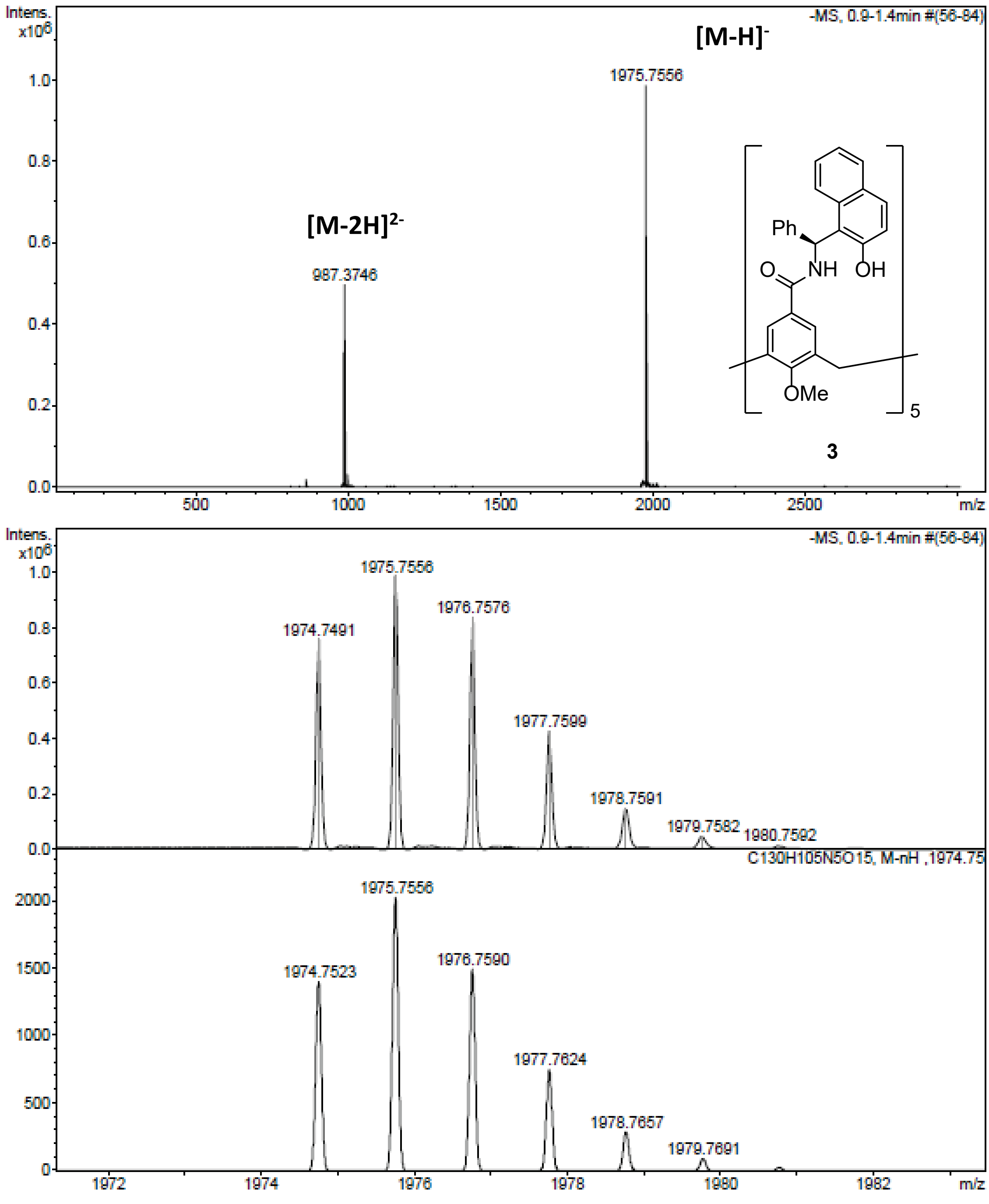

Bruker Compass DataAnalysis 4.0 printed: 22/3/2022 12:44:58 Page 1 of 1

ESI(-) HRMS

Generic Display Report

Analysis Info		Acquisition Date	22/9/2022 10:39:05
Analysis Name	X:\QTOF files\Àngel Oliveras\Agustí Lledo\20220922\20220922_000004.d		
Method	tune_low_negative.m	Operator	Univ. Girona
Sample Name	ALP1630	Instrument	micrOTOF-Q II
Comment			

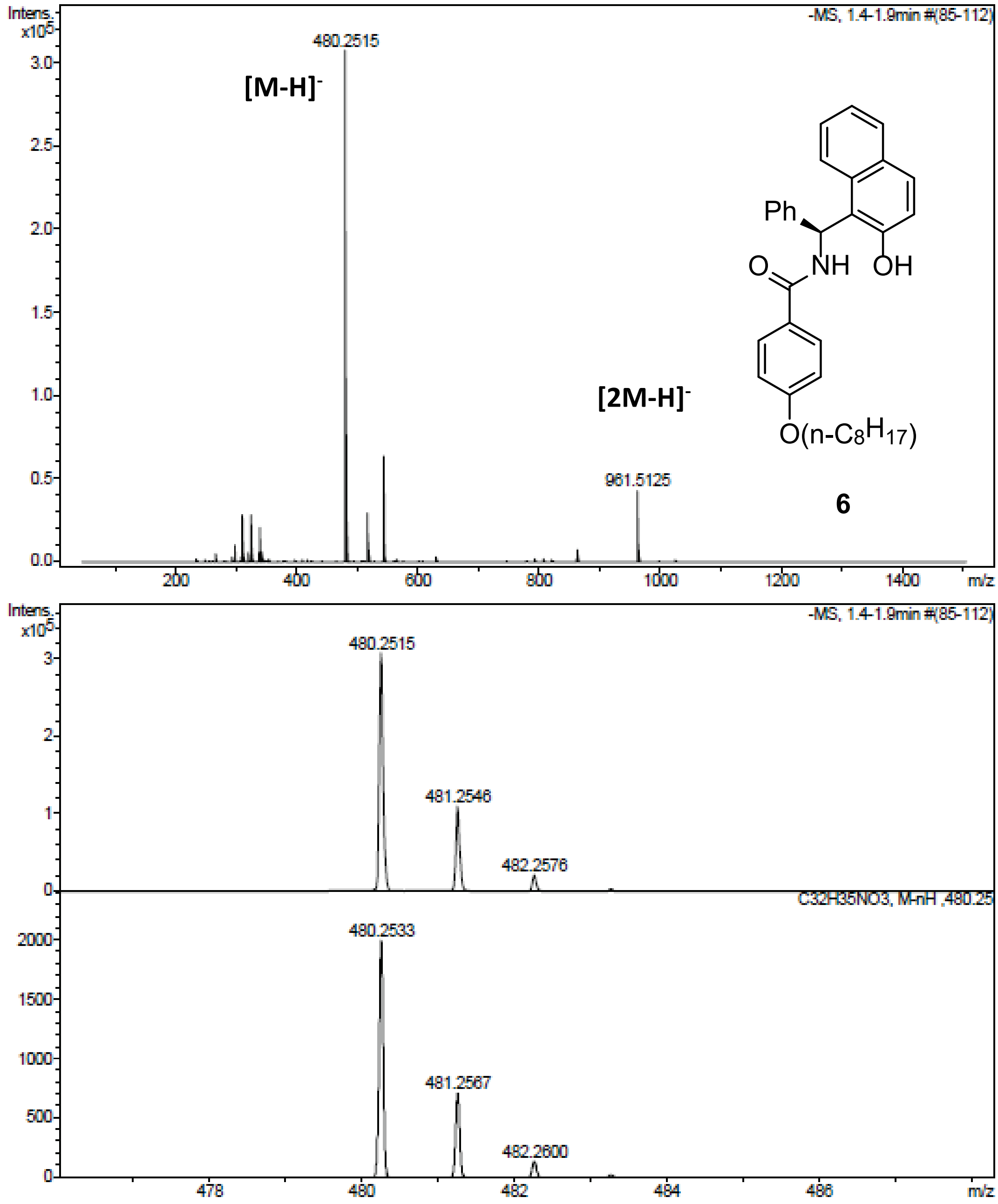

ESI(+) HRMS

Generic Display Report

**Analysis Info**		Acquisition Date	23/9/2022 8:10:52
Analysis Name	X:\QTOF files\Àngel Oliveras\Agustí Lledo\20220922\20220923_000001.d		
Method	tune_low.m	Operator	Univ. Girona
Sample Name	ALP1719	Instrument	micrOTOF-Q II
Comment			

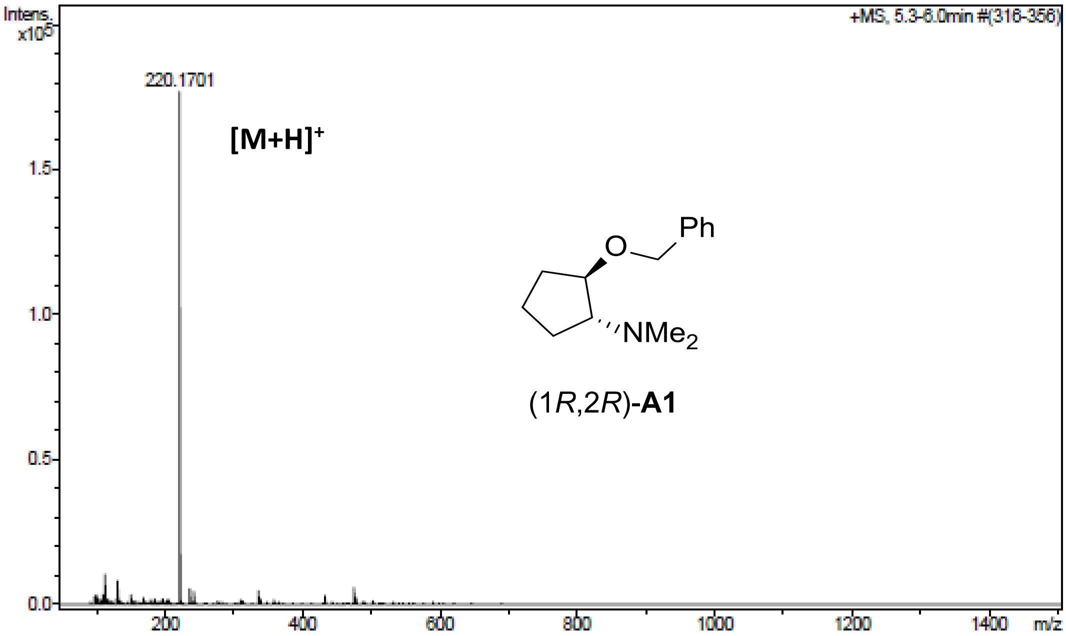

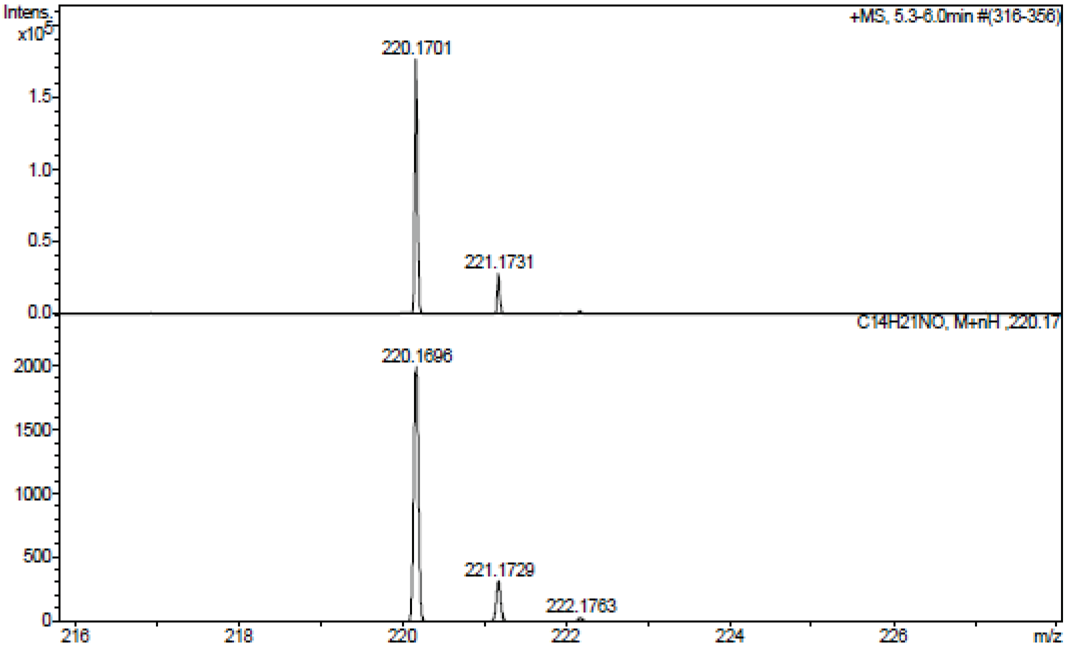

ESI(+) HRMS

Generic Display Report

**Analysis Info**		Acquisition Date	23/9/2022 8:45:14
Analysis Name	X:\QTOF files\Àngel Oliveras\Agustí Lledo\20220922\20220923_000004.d		
Method	tune_low.m	Operator	Univ. Girona
Sample Name	ALP1709	Instrument	micrOTOF-Q II
Comment			

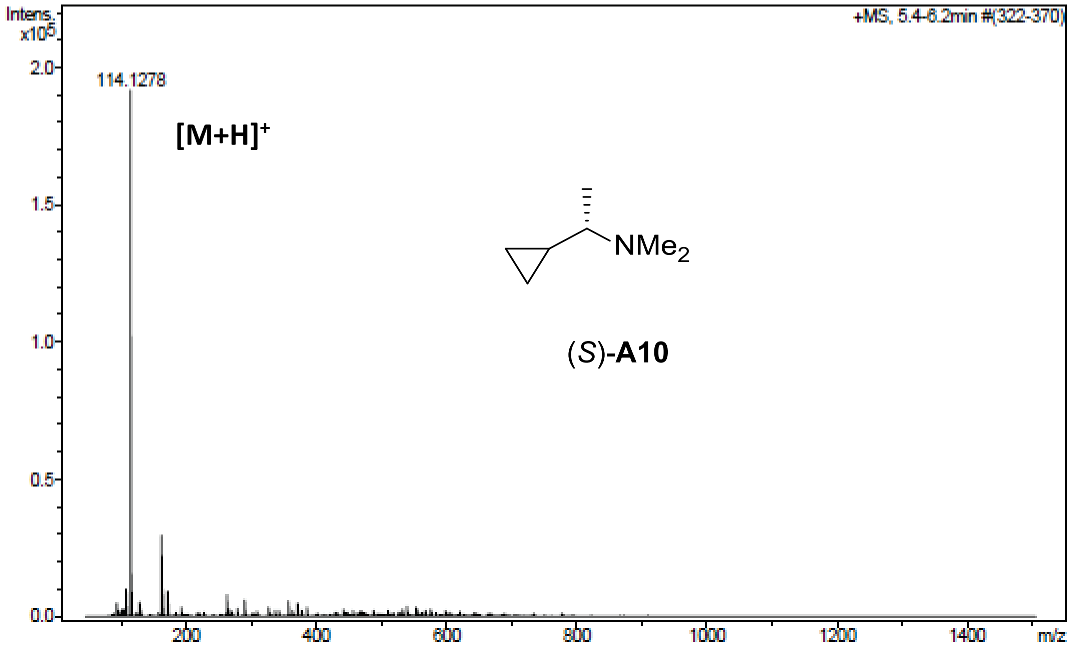

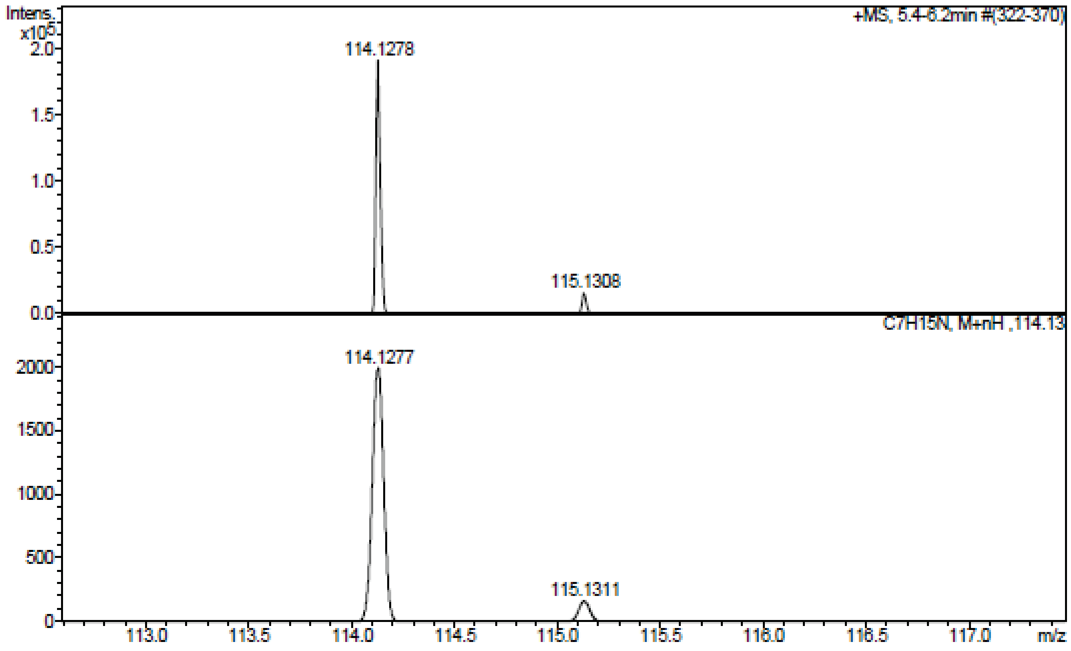

Bruker Compass DataAnalysis 4.0 printed: 23/9/2022 9:21:56 Page 1 of 1

ESI(+) HRMS

Generic Display Report

Analysis Info

Analysis Name	X:\QTOF files\Àngel Oliveras\Agustí Lledo\20220922\20220922_000003.d	Acquisition Date	22/9/2022 10:26:53
Method	tune_low.m	Operator	Univ. Girona
Sample Name	ALP1721	Instrument	micrOTOF-Q II
Comment			

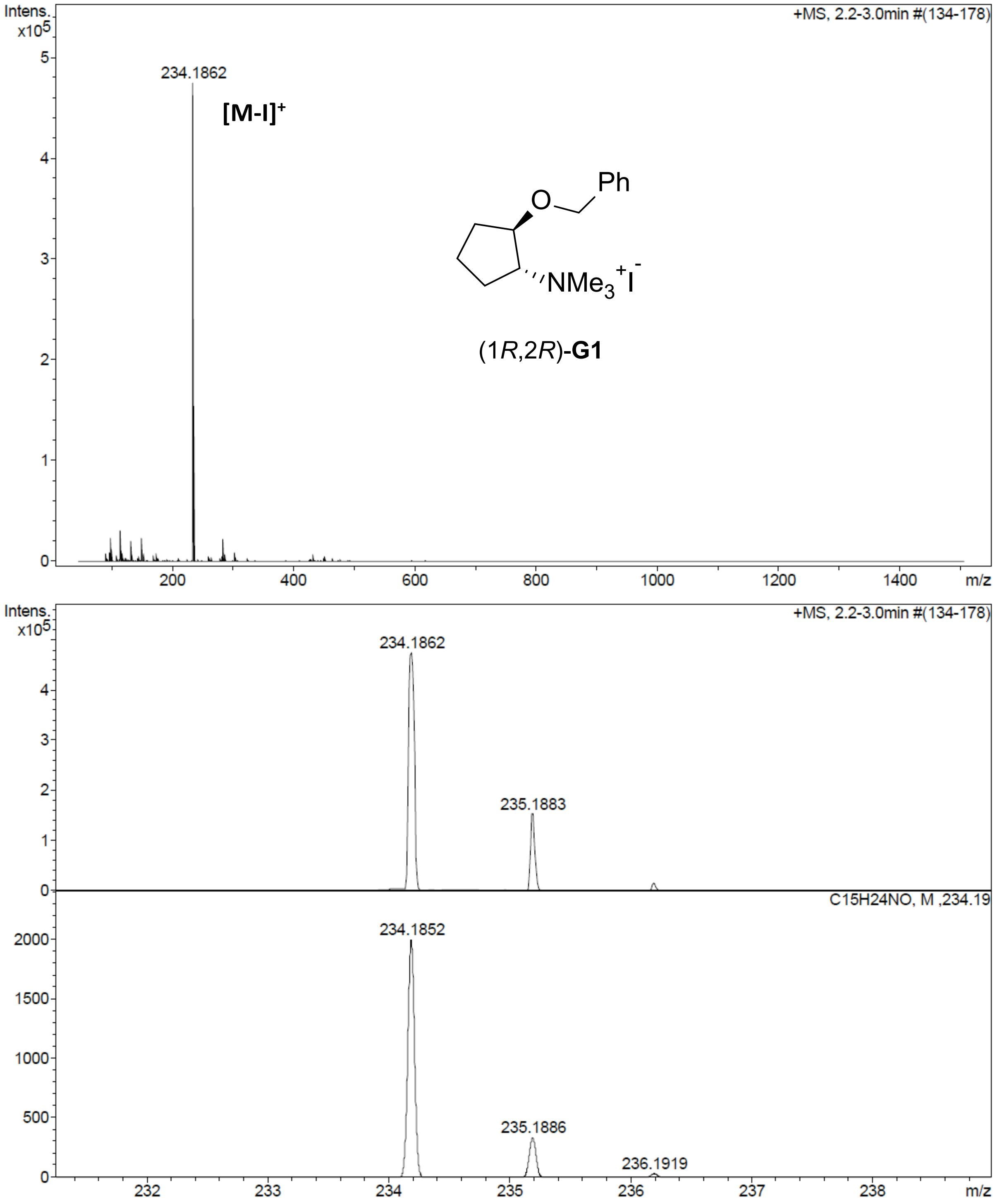

ESI(+) HRMS

Generic Display Report

Analysis Info

Analysis Name	X:\QTOF files\Àngel Oliveras\Agustí Lledo\20220922\20220922_000005.d	Acquisition Date	22/9/2022 10:48:44
Method	tune_low.m	Operator	Univ. Girona
Sample Name	ALP1716	Instrument	micrOTOF-Q II
Comment			

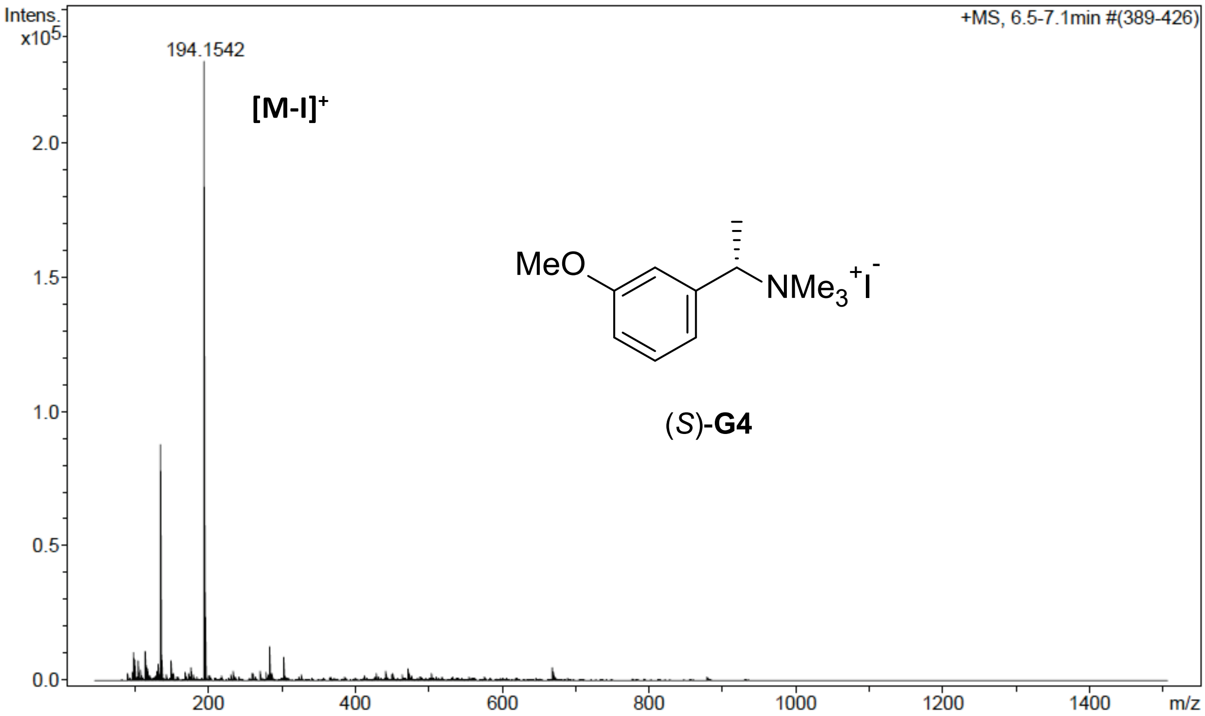

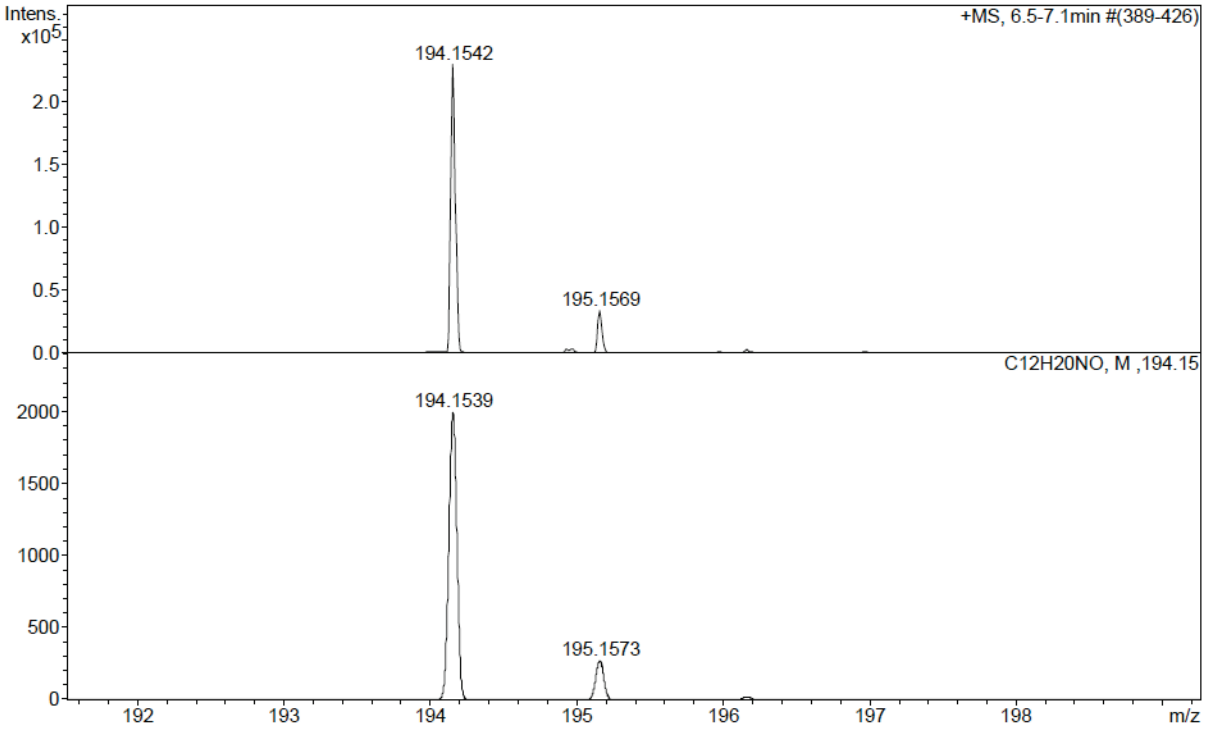

ESI(+) HRMS

Generic Display Report

Analysis Info		Acquisition Date	22/9/2022 12:25:30
Analysis Name	X:\QTOF files\Àngel Oliveras\Agustí Lledo\20220922\20220922_000007.d		
Method	tune_low.m	Operator	Univ. Girona
Sample Name	ALP1690	Instrument	micrOTOF-Q II
Comment			

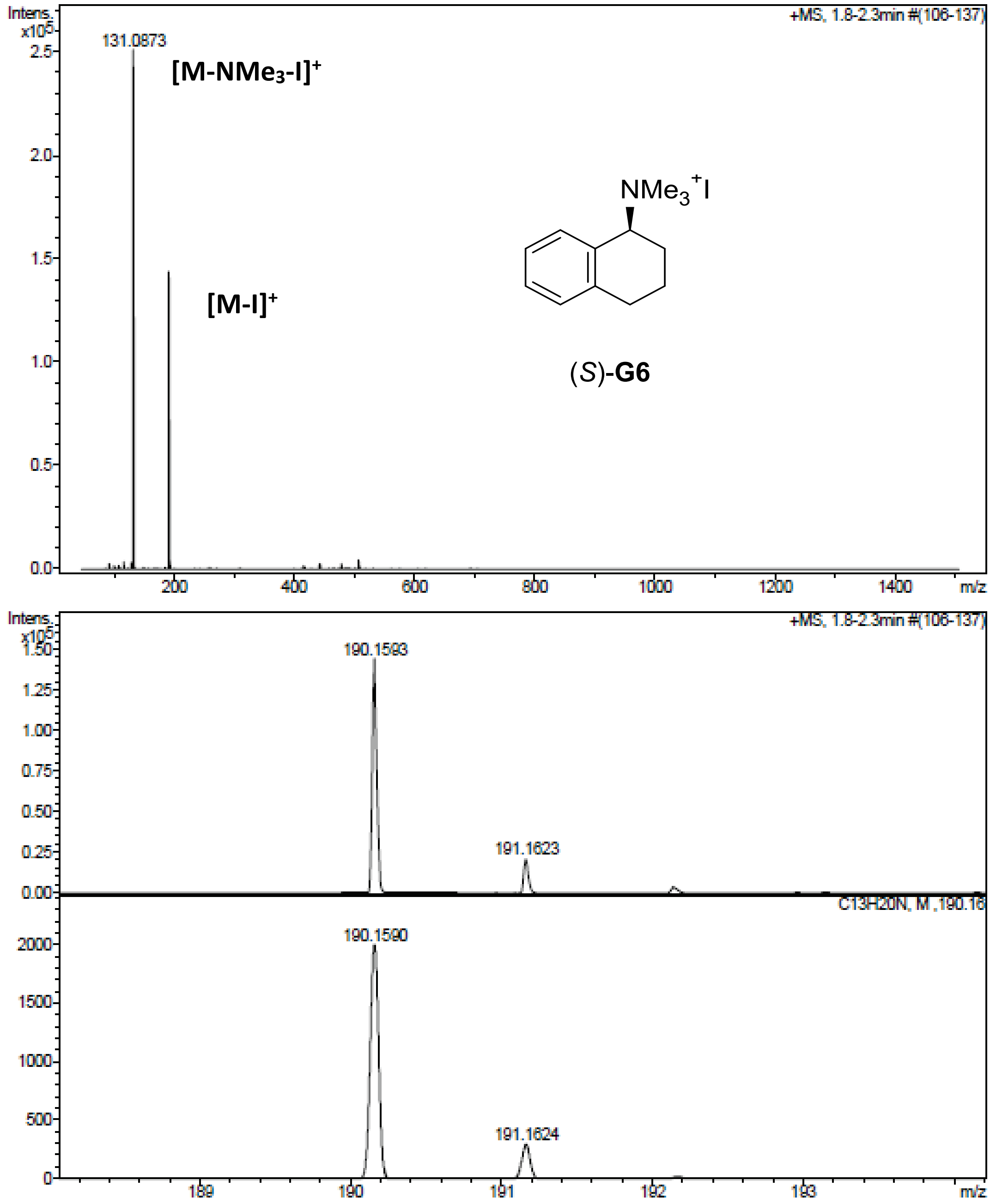

Bruker Compass DataAnalysis 4.0 printed: 23/9/2022 9:25:47 Page 1 of 1

ESI(+) HRMS

Generic Display Report

Analysis Info		Acquisition Date	22/9/2022 12:10:33
Analysis Name	X:\QTOF files\Àngel Oliveras\Agustí Lledo\20220922\20220922_000006.d		
Method	tune_low.m	Operator	Univ. Girona
Sample Name	ALP1678	Instrument	micrOTOF-Q II
Comment			

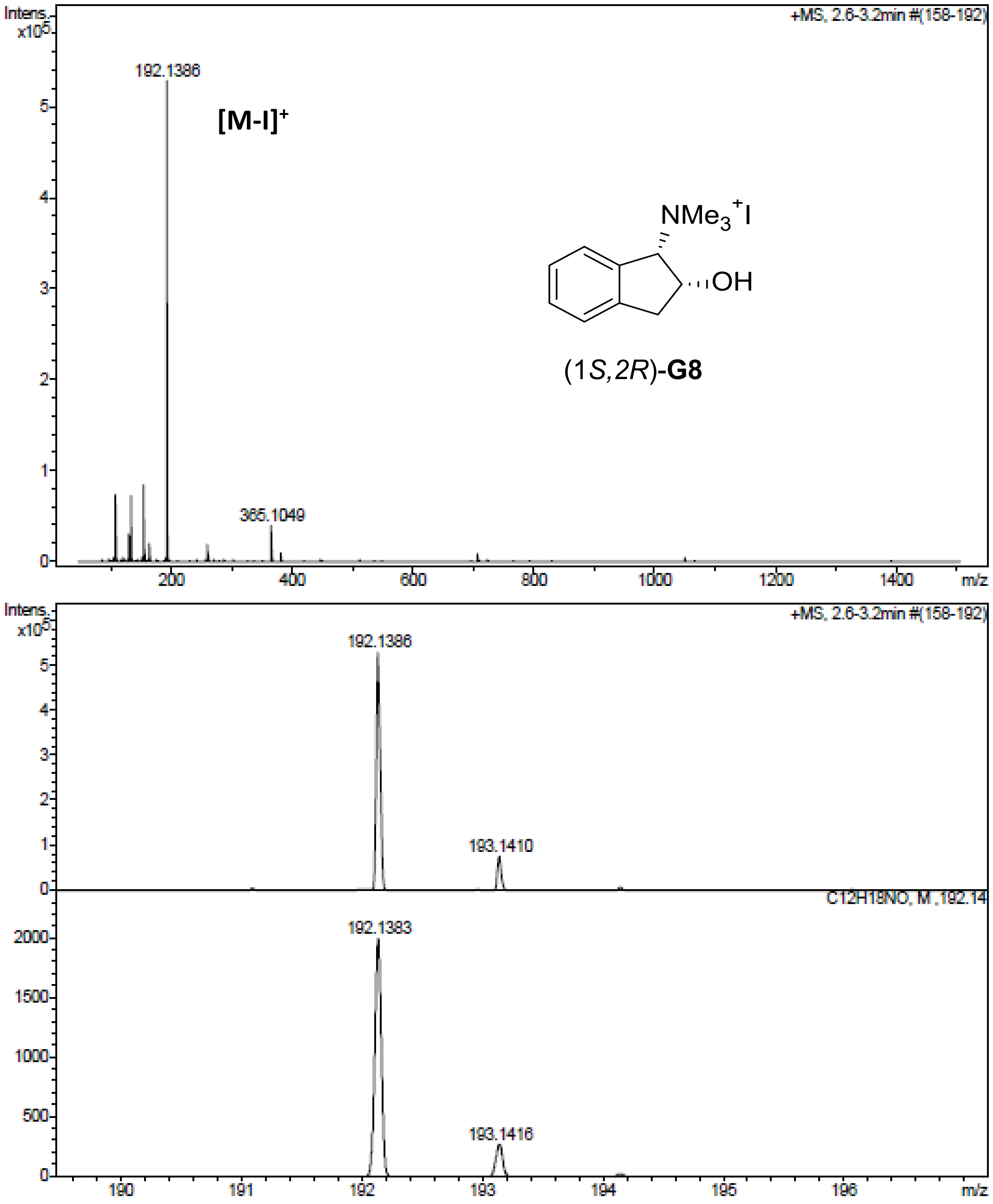

Bruker Compass DataAnalysis 4.0 printed: 23/9/2022 9:34:03 Page 1 of 1

ESI(+) HRMS

Generic Display Report

Analysis Info

Analysis Name	X:\QTOF files\Àngel Oliveras\Agustí Lledo\20220922\20220923_000003.d	Acquisition Date	23/9/2022 8:36:50
Method	tune_low.m	Operator	Univ. Girona
Sample Name	ALP1714	Instrument	micrOTOF-Q II
Comment			

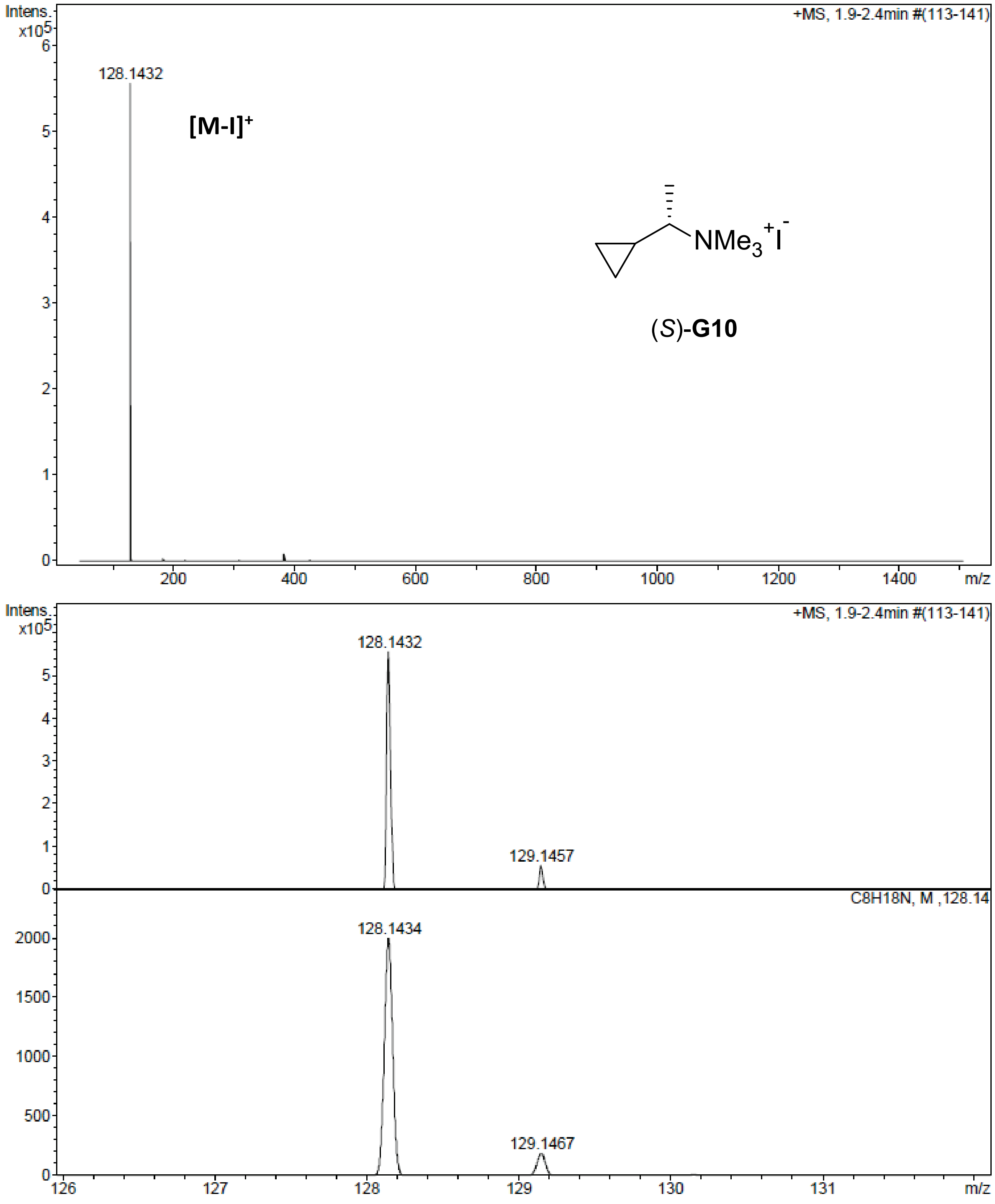

Bruker Compass DataAnalysis 4.0 printed: 23/9/2022 9:39:48 Page 1 of 1
